# An integrative bioinformatics approach reveals coding and non-coding gene variants associated with gene expression profiles and outcome in breast cancer molecular subtypes

**DOI:** 10.1038/s41416-018-0030-0

**Published:** 2018-03-21

**Authors:** Balázs Győrffy, Lőrinc Pongor, Giulia Bottai, Xiaotong Li, Jan Budczies, András Szabó, Christos Hatzis, Lajos Pusztai, Libero Santarpia

**Affiliations:** 1 MTA TTK Lendület Cancer Biomarker Research Group, Institute of Enzymology, Budapest, H-1117 Hungary; 2 2nd Department of Pediatrics, Semmelweis University, Budapest, H-1094 Hungary; 3 Oncology Experimental Therapeutics, Humanitas Clinical and Research Institute, Rozzano, Milan, 20089 Italy; 4 Breast Medical Oncology, Yale Cancer Center, Yale School of Medicine, New Haven, Connecticut 06520 USA; 5 Institute of Pathology, Charité University Hospital, 10117 Berlin, Germany

**Keywords:** Breast cancer, Genetic variation

## Abstract

**Background:**

Sequence variations in coding and non-coding regions of the genome can affect gene expression and signalling pathways, which in turn may influence disease outcome.

**Methods:**

In this study, we integrated somatic mutations, gene expression and clinical data from 930 breast cancer patients included in the TCGA database. Genes associated with single mutations in molecular breast cancer subtypes were identified by the Mann-Whitney *U*-test and their prognostic value was evaluated by Kaplan-Meier and Cox regression analyses. Results were confirmed using gene expression profiles from the Metabric data set (*n* = 1988) and whole-genome sequencing data from the TCGA cohort (*n* = 117).

**Results:**

The overall mutation rate in coding and non-coding regions were significantly higher in ER-negative/HER2-negative tumours (*P* = 2.8E–03 and *P* = 2.4E–07, respectively). Recurrent sequence variations were identified in non-coding regulatory regions of several cancer-associated genes, including *NBPF1*,* PIK3CA* and *TP53*. After multivariate regression analysis, gene signatures associated with three coding mutations (*CDH1*, *MAP3K1* and *TP53*) and two non-coding variants (*CRTC3* and *STAG2*) in cancer-related genes predicted prognosis in ER-positive/HER2-negative tumours.

**Conclusions:**

These findings demonstrate that sequence alterations influence gene expression and oncogenic pathways, possibly affecting the outcome of breast cancer patients. Our data provide potential opportunities to identify non-coding variations with functional and clinical relevance in breast cancer.

## Introduction

Results from DNA-sequencing studies revealed a great degree of genomic heterogeneity in breast cancer, which may partly explain the diversity of clinical behaviour of breast cancer subtypes.^1^ It is also apparent that breast cancer genome is characterised by only a few frequently mutated genes together with a long tail of rare mutations in a great variety of genes, whose potential prognostic impact is difficult to assess due to the small sample size of affected cases.^2^ Furthermore, although it has been suggested that these genetic aberrations can be useful drug targets or biomarkers for patient stratification, their utility in clinical practice has been limited so far.^3^

In addition to mutations in protein-coding regions, a new class of oncogenic events in non-coding areas of the genome has also been identified, suggesting that cancer can result from an array of genomic alterations affecting both coding and non-coding regions.^4^ Most pathogenic DNA sequence alterations directly or indirectly impact gene expression and protein functions, leaving an imprint on messenger RNA expression that can be captured by gene expression profiling.^5, 6^ It is noteworthy that mutation-associated gene expression signatures often have a greater prognostic value than single mutations.^7, 8^

In this study, we used the data from 930 breast cancer patients of The Cancer Genome Atlas (TCGA) database to identify genes with sequence variations in coding and non-coding regions that were captured by exome sequencing. We next identified genes whose expression was associated with a given mutation by comparing cases with and without the mutation. This analysis was done separately for each molecular breast cancer subtype. Once subtype-specific mutation-associated gene signatures were identified, we assessed their effects on survival. Results were also validated using whole-genome sequencing (WGS) data from the TCGA cohort (*n* = 117) and gene expression profiles from the Metabric data set (*n = *1988).

## Materials And Methods

### Data source and acquisition

Whole-exome sequencing (WES), RNA sequencing (RNA-seq) data and the related clinical information from the TCGA database (*n* = 930), as well as WGS data (*n* = 117) were obtained from http://cancergenome.nih.gov/. Gene expression analysis was performed on the pre-processed RNA-seq data (i.e., level 3 data) using MapSplice and RNA-Seq by Expectation-Maximisation. Individual patient files were merged into a single database using the plyr R package.^9^ Information for relapse-free survival was available only for 52 patients and therefore we used overall survival (OS) as outcome measure. Clinical and pathological features of the breast cancer cohort are described in Supplementary Table 1. The entire workflow of the study is summarised in Supplementary Figure 1.

### Processing of sequence variations

Aligned data were downloaded via The Cancer Genomics Hub (https://cghub.ucsc.edu/) for both tumour and matched normal samples. Somatic mutation calls were performed with the MuTect programme, as previously described.^8^ The identified variants were annotated with MuTect using SNP database (dbSNP, build 139) and Catalogue Of Somatic Mutations in Cancer (COSMIC, version 68) library.^10^ The recognised sequence variations were functionally annotated via SNPeff (version 3.5) and filtered to include the COSMIC-identified genes only.^8^ Known cancer-associated genes were defined by the Cancer Gene Census.^10^ We also analysed sequence variants occurring in introns, promoters (defined as − 2.5 kb from transcription starting sites) and other regulatory elements, including enhancers, untranslated regions (UTRs) and transcription factor (TF) binding sites, which were captured by WES. We used the LARVA software to identify significantly mutated non-coding regulatory elements, via modelling with β-binomial distribution and mutation rate calculation through DNA replication timing correction.^11^ To identify potential non-coding drivers, somatic mutations were annotated and prioritised by FunSeq2, combining inter- and intra-species conservation, loss- and gain-of-function events for TF binding, enhancer-gene linkages and network centrality, and per-element recurrence across samples.^12^

### Sample classification according to breast cancer molecular subtypes

Breast cancer molecular subtypes were defined by the oestrogen receptor (ER) and human epidermal growth factor receptor 2 (HER2) status determined by RNA-seq data. For ER the RNA-seq ID 2099 was used with a cut-off of 3,700, whereas for HER2 the RNA-seq ID 2064 was used with a cut-off of 27,000. Cut-off values were determined by a receiver operating curve (ROC) analysis comparing immunohistochemistry- and fluorescence *in situ* hybridisation-based classification with gene expression values. We independently analysed ER-positive/HER2-negative (*n* = 467), ER-negative/HER2-negative (*n* = 185) and HER2-positive (including ER-positive and ER-negative patients, *n* = 278) breast cancers.

### Setup of the validation data set

An independent validation was performed using gene expression data from breast cancer patients in the Metabric cohort.^13^ Illumina gene chip files were obtained from the European Genome-phenome Archive (EGA) (https://www.ebi.ac.uk/ega/). The entire data set contained 1988 samples, of which 1386 were ER-positive/HER2-negative, 271 were HER2-positive and 331 were ER-negative/HER2-negative. Instead of using the published pre-processed data set including two separate normalisations, all arrays were re-normalised in one setting. For this, the expression data was imported into R (https://www.r-project.org/) and summarised using the beadarray library.^14^ Probes not mapped to a given gene were deleted during summarisation (*n* = 319). Finally, quantile normalisation was performed using the preprocessCore package (https://github.com/bmbolstad/preprocessCore). In case of multiple probes targeting the same gene, the probe with the highest detection range was used. Statistical computations were performed as for the RNA-seq data.

### Gene expression signatures

Mann-Whitney *U*-test was performed to identify genes whose expression was significantly associated with a given genotype (i.e., somatic mutation) in each breast cancer subtype, separately. Samples were divided into two cohorts according to the mutation status and the cohorts were compared to each other. The analysis was performed for all coding and non-coding sequence variations without filtering for functional significance. The average expression of the significantly mutation-associated genes was designated as the gene expression signature of a given genotype. The expression of the downregulated genes was inverted before computing the mean expression of the signature. Significant associations from the Mann-Whitney *U*-test (*P* ≤ 0.01) were ranked based on their achieved *P*-values. Finally, a maximum of 100 significant genes for each signature was included to reduce noise in large gene sets.

### Statistical analyses

To examine the association of mutation status and mutation-associated signatures with OS, we performed Kaplan-Meier and Cox proportional hazard regression analyses using the median expression of the signature to dichotomise the population. Multivariate analysis was performed including tumour size (T stage), lymph node status (N stage), the presence of distant metastases (M stage) and *MKI67* gene expression as a measure of proliferation. The level of statistical significance was set at *P < *0.05.

## Results

### Database characteristics

Nine hundred and thirty patients with invasive breast cancer were analysed, including 50.2% ER-positive/HER2-negative, 19.9% ER-negative/HER2-negative and 29.9% HER2-positive cancers. The mean age of patients was 58.3 years. Nodal status was available for 905 patients, of which 45.3% were lymph node positive. The median follow-up was 31.5 months. The detailed characteristics of patients included in the analysis are presented in Supplementary Table 1.

### Genetic variants in coding and non-coding regions in breast cancer

We found 208 and 3562 genes with sequence variations in coding and non-coding areas in at least 2% of total samples, respectively. Recurrent sequence variants occurring in > 5% of cases in coding and non-coding regions were found in 29 and 675 genes, respectively. The complete list of all genes with genetic variants in coding sequences is showed in Supplementary Table 2, whereas variants in non-coding areas are listed in Supplementary Table 3.

**Fig. 1:**
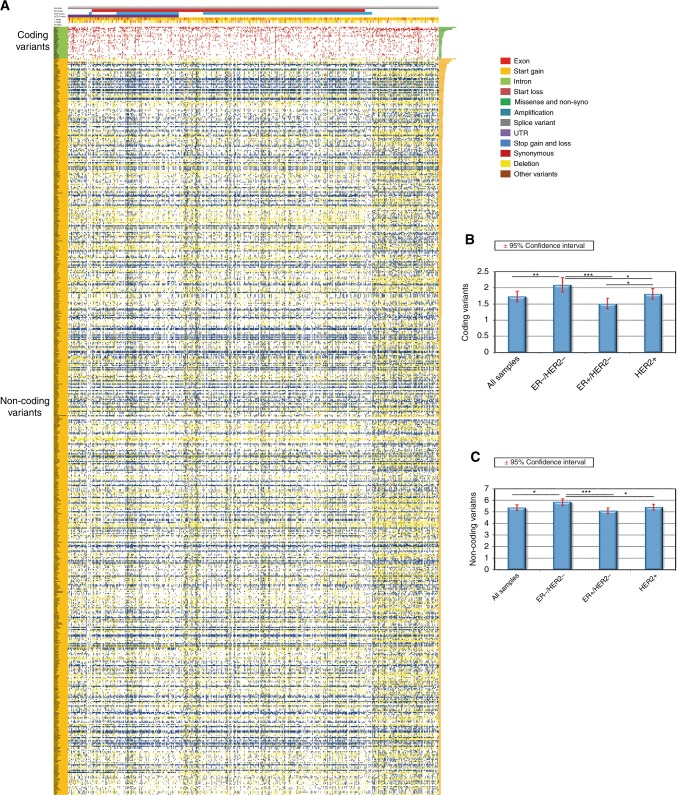
Genes characterised by genetic variations in coding and non-coding regions in breast cancer. **a** A heatmap for recurrent variations in coding (green) and non-coding (orange) regions was generated for 930 tumours of the TCGA cohort. Clinicopathological features for each sample (i.e., ER, PGR, HER2 status and TNM stage) are shown. Each column represents an individual tumour and each row represents a gene. Different types of variations are highlighted with different colours. The right histogram displays the percentage of breast cancer harbouring sequence variants in the corresponding gene. Histograms showing the number of variations in **b** coding and **c** non-coding regions across all genes among all patients and each BC subgroup. Error bars represent 95% confidence intervals. *P < 0.05; **P < 0.01; *** *P < *0.001

The rate and pattern of genetic alterations differed between breast cancer subtypes, highlighting the genomic heterogeneity of breast tumours (Fig. 1). An overview of sequence variants in coding and non-coding regions and major associated clinical features, including receptors status and TNM stage, is provided in Fig. 1a. The overall mutation rate in both coding and non-coding regions was significantly higher in ER-negative/HER2-negative compared with HER2-positive and ER-positive/HER2-negative cancers (*P* = 2.8E–03 and *P* = 2.4E–07, respectively), which is consistent with the extensive genomic instability characterizing triple-negative breast tumours (TNBC) (Fig. 1b, c). We also confirmed that mutations in *PIK3CA* (32.0%) and *TP53* (24.4%) were the most frequent genetic aberrations in cancer-related genes in breast tumours, followed by *CDH1* (4%), *MAP3K1* (4%), *PTEN* (3%) and *PTPRD* (3%) (Fig. 1a and Supplementary Table 2). An illustrative overview of the type and distribution of mutations in *TP53* and *MAP3K1* is provided in Supplementary Figure 2. Recurrent sequence variations were also found in the non-coding regulatory regions of several cancer-associated genes, including *NBPF1* (44.2%), *TP53* (26.2%), *PIK3CA* (18.8%) and *OBSCN* (11.6%) (Fig. 1a and Supplementary Table 3).

As these findings derived from the analysis of WES data, which are not able to comprehensively describe the landscape of non-coding changes, we also validated our results by analysing WGS data from 117 breast cancers enclosed in the TCGA cohort. Among the 3562 genes that showed sequence variations in non-coding areas from the WES analysis (Supplementary Table 3), we confirmed that 3442 genes had the same mutation profile in WGS analysis, demonstrating that the non-coding areas of these genes were truly affected by sequence variations (Supplementary Table 4).

**Fig. 2:**
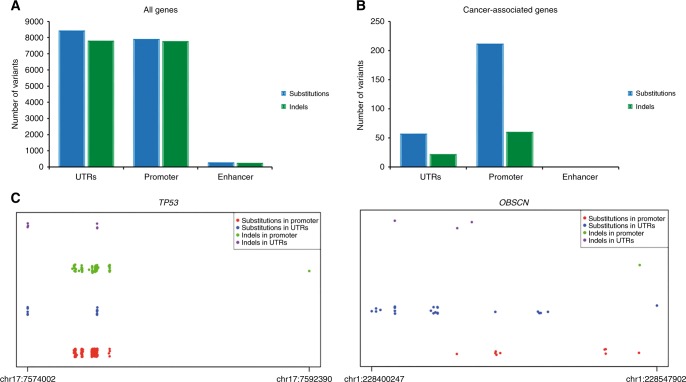
Distribution of non-coding variants in functional genomic regions in breast cancer. **a** Distribution of substitutions and insertions/deletions (indels) in significantly mutated non-coding regulatory regions of all genes. **b** Distribution of substitutions and indels in non-coding regulatory areas of cancer-related genes. **c** Distributions of substitution and indels in the promoter and untraslated regions (UTRs) of two cancer-associated genes

To dissect the functional value of genetic alterations in non-coding regions and identify potentially novel non-coding drivers, we employed the computational framework LARVA and the FunSeq2 tool. We identified several recurrent non-coding variants in fundamental regulatory sites such as the promoter and UTR regions. Overall, we found that substitutions and insertions/deletions (indels) were present with the same frequency in non-coding elements of all genes (Fig. 2a). Interestingly, substitutions were the most common variations in non-coding regulatory areas, especially promoters, of cancer-related genes, including *OBSCN* and *TP53* (Fig. 2b,c). It is worth noting that variants in non-coding regions can have a functional impact by altering gene transcription and translation through the modification of promoters and regulatory elements. Accordingly, we found several genetic alterations in promoters, introns, and other non-coding regulatory regions, which can possibly alter the phosphorylation, protein–protein interaction, and regulatory networks involving different cancer-related genes, including *ATM/ATR*, *FGFR1*, *FOXA1*, *IGF1R*, *NF1*, *NOTCH2* and *TOP2A* (Supplementary Table 5). In addition, we found a sequence variation in the UTR of the mediator of DNA damage response *ATR* that can lead to a defective function of the RNA decay factor XRN1 (Supplementary Table 5). Finally, we reported different variants that can be responsible of motif-breaking events or gain of new binding sites for TFs that can potentially constitute functionally relevant driver events, especially when occurring in cancer-associated genes, such as *AKT2*, *CDKN1B*, *ERBB2*, *FBXO11*, *NF1*, *PTCH1* and *TP53* (Table 1 and Supplementary Table 5).

**Table 1:** Major functional variations in non-coding regions of cancer-associated genes in breast cancer patients from the TCGA database

Chromosome	Location	Reference	Alteration	Target gene	Network	Motif analysis	Functions and annotation	Score
chr19	41354417	G	C	*AKT2* (Distal)	Protein–protein interaction, regulatory	Motif-gaining: CHD2	Cancer, actionable	1.92
chr12	12870230	G	A	*CDKN1B* (Promoter and UTR)	Protein–protein interaction, regulatory	Motif-breaking: JUND/AP-1	DNA repair	4.11
chr17	37871614	T	G	*ERBB2* (Intron and Promoter)	Protein–protein interaction, regulatory	Motif-gaining: ZEB1	Cancer, actionable	3.71
chr2	48061657	A	T	*FBXO11* (Intron and Promoter)	Protein–protein interaction	Motif-gaining: GATA	Cancer	1.92
chr17	29684678	C	A	*NF1* (Intron and Promoter)	Protein–protein interaction, regulatory	Motif-breaking: MAFF	Cancer, actionable	1.46
chr9	98244061	T	C	*PTCH1* (Intron and Promoter)	Protein–protein interaction	Motif-gaining: FOXA	Cancer, actionable	2.45
chr17	7578564	G	A	*TP53* (Intron and Promoter)	Protein–protein interaction, regulatory, phosphorylation	Motif-gaining: PU.1	DNA repair, cancer, actionable	3.15

### Effect of genetic variants on patients’ survival

**Fig. 3:**
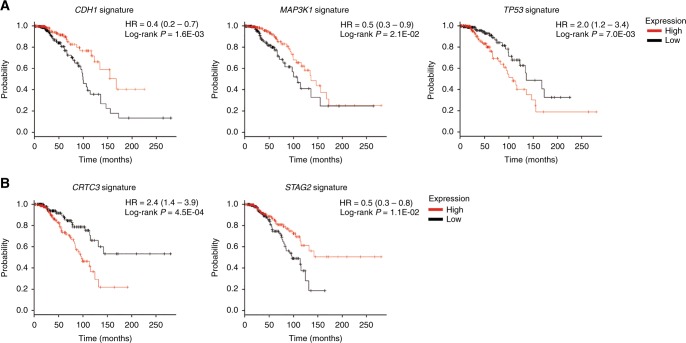
Prognostic value of expression signatures correlated with genetic variants in cancer-associated genes. **a** Kaplan-Meier analysis of overall survival for the signatures associated with mutations in the coding areas of *CDH1*, *MAP3K1* and *TP53* in ER-positive/HER2-negative breast cancer. **b** Kaplan-Meier analysis of overall survival for the signatures associated with sequence variations in the non-coding regions of *CRTC3* and *STAG2* in ER-positive/HER2-negative breast cancer

We then investigated the prognostic value of genomic variants in both coding and non-coding areas in breast cancer subtypes. We did not find any direct association between genetic alterations in coding or non-coding regions and breast cancer patients’ outcome in univariate analysis (Supplementary Table 6). As cancer-related DNA sequence alterations can have a substantial impact on gene expression and consequently influence important signalling pathways, to identify gene networks correlated with genetic alterations and understand their effects on clinical outcome we generated gene expression signatures associated with each variant in coding and non-coding regions separately and performed univariate and multivariate survival analysis according to breast cancer subtype. After multivariate analysis, seven signatures related to coding mutations (*ABCA13*, *CDH1*, *MAP3K1*, *MUC16*, *NEB*, *TAB3* and *TP53*) were associated with OS in ER-positive/HER2-negative tumours. In particular, the *TP53*-related signature enclosing genes predominantly involved in cell cycle, DNA repair, signal transduction and apoptosis was predictive of poor prognosis in ER-positive/HER2-negative breast cancer (Table 2). Only two signatures (*MUC12* and *RYR2*) predicted prognosis in ER-negative/HER2-negative cancers (Table 2). Furthermore, we found 15 gene signatures associated with non-coding variants that were predictive of OS in ER-positive/HER2-negative breast cancer, including *CRTC3-* and *STAG2*-related signatures (Table 2). Conversely, we demonstrated that only the *CROCC*-associated signature, which included the DNA homologous recombination factor *RAD52*, the tumour suppressor gene *HIC1* and the repressor of the sonic hedgehog pathway *TULP3*, had a prognostic value in ER-negative/HER2-negative cancers (Table 2). No gene signatures remained significant after multivariate analysis in HER2-positive tumours. Kaplan-Meier analyses for known cancer-associated genes are shown in Fig. 3. All the genes included in each significant coding- and non-coding-related signature, whose expression and/or function may be directly or indirectly affected by the presence of the given genetic alteration, are listed in Supplementary Tables 7 and 8, respectively.

**Table 2:** Variants-related transcriptional signatures associated with overall survival in breast cancer patients from the TCGA database

	Univariate analysis		Multivariate analysis				
Gene	*P*-value	HR	*P*-value	HR	Variable	*P*-value	HR
**ER-positive/HER2-negative breast cancers (** ***n*** ** = 467)**							
Variants in coding regions							
*ABCA13*	7.8E–03	0.5	4.5E–04	0.4	T stage	4.0E–02	1.4
*CDH1*^a^	1.6E–03	0.4	1.2E–03	0.4	-		
*MAP3K1*^a^	2.1E–02	0.5	4.0E–02	0.6	-		
*MUC16*	4.4E–02	1.7	3.0E–02	1.8	-		
*NEB*	2.4E–03	0.4	2.6E–04	0.3	M stage	3.0E–02	3.4
*TAB3*	1.7E–03	2.3	4.4E–04	3.1	T stage	3.0E–02	1.5
*TP53*^a^	7.0E–03	2.0	5.9E–03	2.4	-		
Variants in non-coding regions							
*AAK1*	1.10E–02	1.9	6.60E–04	2.9	-		
*CA5A*	8.50E–03	0.5	2.51E–05	0.3	-		
*CRTC3*^a^	4.50E–04	2.4	2.00E–03	2.5	M stage	4.0E–02	2.9
*CTNNA2*	1.80E–02	0.6	1.50E–02	0.5	-		
*DOCK2*	4.20E–05	2.8	1.10E–02	2.2	-		
*FAM118A*	2.80E–02	1.7	7.60E–03	2.3	M stage	2.0E–02	3.7
*FASTKD1*	5.20E–03	2.0	2.50E–02	2.0	-		
*HDLBP*	3.30E–03	2.1	3.00E–02	2.0	M stage	3.0E–02	3.8
*HUS1*	2.40E–02	1.7	4.30E–02	1.8	-		
*PDZD7*	1.20E–02	1.8	2.60E–02	2.0	-		
*PPP1R12A*	3.20E–07	3.5	1.40E–04	3.4	-		
*RYR3*	3.60E–02	0.6	1.40E–02	0.5	-		
*STAG2*^a^	1.10E–02	0.5	7.70E–03	0.4	-		
*TMEM50A*	4.30E–03	2.0	9.20E–04	2.7	-		
*TTC27*	2.70E–03	2.0	4.80E–03	2.3	-		
**ER-negative/HER2-negative breast cancers (** ***n*** ** = 185)**							
Variants in coding regions							
*MUC12*	8.3E–03	0.3	6.0E–03	0.3	N stage	2.9E–05	3.2
*RYR2*	5.7E–03	0.3	8.9E–03	0.3	N stage	3.4E–06	3.6
Variants in non-coding regions							
*CROCC*	3.10E–03	3.1	3.0E–02	2.9	N stage	1.0E–05	3.3

*ER* oestrogen receptor; *HER2* human epidermal growth factor receptor 2; *HR* hazard ratio.

^a^Known cancer-associated genes defined according to the Cancer Gene Census.

To independently validate the prognostic value of the identified signatures, we used gene expression data from the Metabric cohort. We confirmed that the signatures associated with genes mutated in coding areas, including *CDH1, MAP3K1, NEB, TAB3* and *TP53*, as well as the signatures related to genes with sequence variations in non-coding regions, such as *AAK1*, *CA5A*, *FASTKD1*, *HUS1* and *PDZD7*, were predictive of OS in ER-positive/HER2-negative breast cancer (Supplementary Table 9). We were not able to validate the association of *STAG2*- and *CRTC3-*related signatures with survival in ER-positive/HER2-negative cancers due to the lack of the probe sets specific for these signatures in the Illumina gene chips. Furthermore, *MUC12* and *RYR2* signatures demonstrated a consistent prognostic value in ER-negative/HER2-negative breast cancer (Supplementary Table 9). Illustrative Kaplan-Meier plots generated from the analysis of the validation cohort are shown in Supplementary Figure 3.

## Discussion

Human cancers are characterised by changes in DNA sequence, which confer oncogenic traits and influence the transcriptomic and proteomic landscape of tumours, potentially affecting the clinical course of the disease. In particular, breast cancer is associated with a few frequently mutated genes together with many rare mutations.^2, 15^ Among breast cancer subtypes, we confirmed that ER-negative/HER2-negative breast cancers have the highest overall mutation rate.^1^ This increased mutation frequency is likely due to the intrinsic genomic instability and the high rate of sequence alterations in genes involved in DNA damage response (e.g., *BRCA1/2* and *TP53*), which characterise TNBC.^15–17^ Moreover, we demonstrated different genetic variations in additional potential candidate cancer genes.

Several studies have attempted to investigate the prognostic relevance of somatic mutations within single genes in breast cancer, mainly *TP53* and *PIK3CA*.^18–20^ However, a clinically useful association between gene mutations and outcome has not been clearly demonstrated so far.^2^ Accordingly, we demonstrated that single genetic alterations in coding and non-coding regions have no prognostic value in breast cancer. Otherwise, DNA sequence variations may affect the expression of functionally related genes, leading to the alteration of key signalling pathways, and changes in gene expression related to genetic variations can directly reflect the influence of the genotype over the phenotype.^2, 6, 18, 21^ Consistently, apart from single genes, specific mutational signatures have been recently related to transcriptomic pathways, including cell cycle and immune response, in breast cancer molecular subtypes.^21, 22^ Thus, the identification of these “transcriptomic fingerprints” may improve the recognition of clinically significant oncogenic pathways that could be used to develop novel pathway-directed drugs in molecular breast cancer subtypes.

In a previous study, we have demonstrated a link between tumour genotype and gene expression in breast cancer.^8^ However, this study had three major limitations. First, all breast cancer patients were evaluated together regardless of the molecular subtypes. Second, the ROC analysis performed to identify genes to be included in the signatures did not allow a direct transfer of results to the validation phase due to the need of cut-off selection. Finally, non-coding mutations were omitted during the analysis. In the current study we overcome these limitations using a different approach to identify gene networks correlated with sequence variations in coding and non-coding regions and to correlate the identified signatures with clinical outcome in molecular breast cancer subtypes. Our analysis identified few but relevant prognostic signatures associated with known cancer-related genes mutated in coding-regions (e.g., *CDH1, MAP3K1*, *TAB3* and *TP53*) in ER-positive/HER2-negative breast cancer. In agreement with previous data, which associated *CDH1* and *MAP3K1* mutations with indolent ER-positive/luminal A phenotypes, we showed that the unbalance of gene networks caused by genetic alterations in the coding regions of these genes strongly correlated with good clinical outcome in both the TCGA and the Metabric cohorts.^23, 24^ We also confirmed the prognostic value of *TP53* mutations-derived signature in ER-positive breast tumours using an independent set of samples.^23, 25–27^ Importantly, the finding of genes included in the *TP53*-related signature are predominantly involved in cell cycle, DNA repair, signal transduction, and apoptosis substantiates the robustness of our approach, confirming that genetic alterations can directly or indirectly affect the expression and functions of other cancer-associated genes, ultimately impairing key signalling pathways and patients’ prognosis.

In addition, data derived from comprehensive next-generation sequencing studies have tried to enable the identification of potential driver events in breast cancer.^15, 18, 28–30^ However, several of these genes with sequence variations were not previously classified as canonical cancer genes.^31^ In line with a recent report we found the presence of several coding, potentially driver, mutations occurring outside of known cancer genes.^32^ It is conceivable that the majority of the genetic alterations of these genes lead to biological effects that converge on key regulatory pathways, indirectly modulating oncogenic signalling. For instance, we found variations in the genomic sequence of the adaptor protein TAB3, which may impair its ability to bind the TAK1 kinase, ultimately affecting nuclear factor-κB and mitogen-activated protein kinase pathways.^33^

Even though most genomic analyses have focused on protein-coding areas, new classes of oncogenic events are being discovered in the non-coding regions of the genome.^4^ Although our results were derived from the analysis of WES data, which only partially cover non-coding elements, we demonstrated that the non-coding regions of these genes were truly affected by sequence variations by analysing WGS data from breast cancers enclosed in the TCGA cohort. Importantly, we found that variants in non-coding regions are more abundant than coding mutations, as most non-coding mutations correspond to passenger events or minor driver events. Furthermore, we demonstrated that different types of DNA alterations occur with distinct frequencies in non-coding areas of the genome, especially in the promoters and UTRs of cancer-associated genes, as well as cancer unrelated genes. Noteworthy, recent data suggest that different mutational processes imprint specific patterns of genomic changes and that these mutational signatures show distinct associations with transcription, DNA replication time, gene density and physical chromatin organisation.^15, 22^ Furthermore, it has been demonstrated that mutations of specific regulatory sites are under selective pressure and frequently occur in proximity to known cancer genes.^34^ Even though these mechanisms warrant further studies, they may partially explain the pattern of mutation profile we found in the promoters and UTRs of cancer-related genes.

However, distinguishing driver from passenger mutations in non-coding regions is challenging for the larger number of mutations in non-coding elements than coding regions. In addition, non-coding regions are incompletely annotated and generally function within complex regulatory networks and, therefore, current methods developed on coding sequence properties may be less robust for non-coding drivers identification. Although, it is likely that a subset of non-coding mutations may work as ‘mini drivers’ in cancer. In order to identify the ‘mini drivers’ in non-coding regions, we need to understand their functional impact. In this study, we demonstrated the presence of several genetic variations in regulatory elements, including promoters, enhancers, UTRs and introns, which might influence gene expression, as well as the functions, interactions and regulatory networks involving important genes, such as *ATM/ATR*, *FGFR1*, *FOXA1*, *IGF1R*, *NF1*, *NOTCH2* and *TOP2A*, thus functionally impairing specific oncogenic signalling in breast cancer. For instance, we found a repetitive sequence variation in the UTR of *ATR*, which is a well-known mediator of DNA damage response. This specific variation may alter the function of the RNA decay factor XRN1 that has been involved in the initiation of DNA double-strand breaks processing, control of checkpoint activation and regulation of telomere metabolism, thus suggesting a functional role for this non-coding variant to control genome stability.^35, 36^ Furthermore, consistent with our results, recent findings indicated that a mutational hotspot in the regulatory region of *FOXA1* leads to protein overexpression through the increased binding of the TF E2F in breast cancer.^37^ Alterations of non-coding sequences can also cause motif-breaking or -gaining events that affect gene expression through the modification of binding sites for TFs (e.g., AP-1, FOXA, GATA, MAFF, PU.1 and ZEB1) or chromatin organisation modifier (e.g., CHD2). For instance, the presence of a genetic variation in the non-coding sequence of *ERBB2* generating a binding motif for the major inducer of epithelial-to-mesenchymal transition ZEB1 can potentially increase the aggressiveness and tumourigenic potential of HER2-positive breast cancer cells.

To understand the functional relevance of such variations in non-coding regions we assessed their association with gene expression and patients’ outcome and demonstrated that the transcriptomic signature of *STAG2* gene was associated with good prognosis in ER-positive/HER2-negative breast cancer. Studies on the biological and clinical relevance of *STAG2* mutations have generated conflicting results in different type of malignancies, and the functional consequence of variations in the non-coding sequence of *STAG2* in breast cancer remains to be determined.^38^ Furthermore, the prognostic value of the *CROCC*-associated signature in ER-negative/HER2-negative breast cancer, including *RAD52, HIC1* and *TULP3* genes, which have been all associated with TNBC, warrants further investigations.^39–42^

Overall, we identified very few gene mutations associated with breast cancer outcome, highlighting the needs to further explore the non-coding portion of the genome. However, some limitations might apply to this study. First, we used only OS data, the TCGA cohort is characterised by a relatively median short-term follow-up and few associated death events. Second, the sample size for the different breast cancer molecular subtypes is diverse, thus reducing the statistical power to identify gene mutations significantly associated with gene expression and survival. Finally, incomplete coverage of non-coding elements limits the identification of consistent variants in these regions. Thus, further specific studies are required to determine the real significance of sequence changes in the non-coding areas of the breast cancer genome. Another inherent limitation of all mutation-based analysis is the infrequency of recurrent mutations in breast cancer besides *TP53* and *PIK3CA*. Owing to limited sample number we collapsed mutations at a single gene level, thus excluding the possibility to assess the impact of different mutations at the single codon level. Furthermore, our results might likely benefit of an additional layer of data integration complexity, including protein expression.

In conclusion, our results identify novel sequence variations in coding and non-coding elements suggesting that a deeper understanding of the mutational landscape of these regions may help to identify clinically relevant and potentially druggable gene targets in molecular breast cancer subtypes.

## Electronic supplementary material

Supplementary Figures

Supplementary Table 1

Supplementary Table 2

Supplementary Table 3

Supplementary Table 4

Supplementary Table 5

Supplementary Table 6

Supplementary Table 7

Supplementary Table 8

Supplementary Table 9

## Availability of data and materials

The original data used in this study are available from TCGA (http://cancergenome.nih.gov/) and the EGA (https://www.ebi.ac.uk/ega/).
