# Supplementary material for: An integrative bioinformatics approach reveals coding and non-coding gene variants associated with gene expression profiles and outcome in breast cancer molecular subtypes
Source: Br J Cancer. 2018 Mar 21;118(8):1107–14. doi: 10.1038/s41416-018-0030-0 (PMC5931099; doi:10.1038/s41416-018-0030-0)
Supplement: Supplementary file 1 — Supplementary Figures [file 41416_2018_30_MOESM1_ESM.pdf]

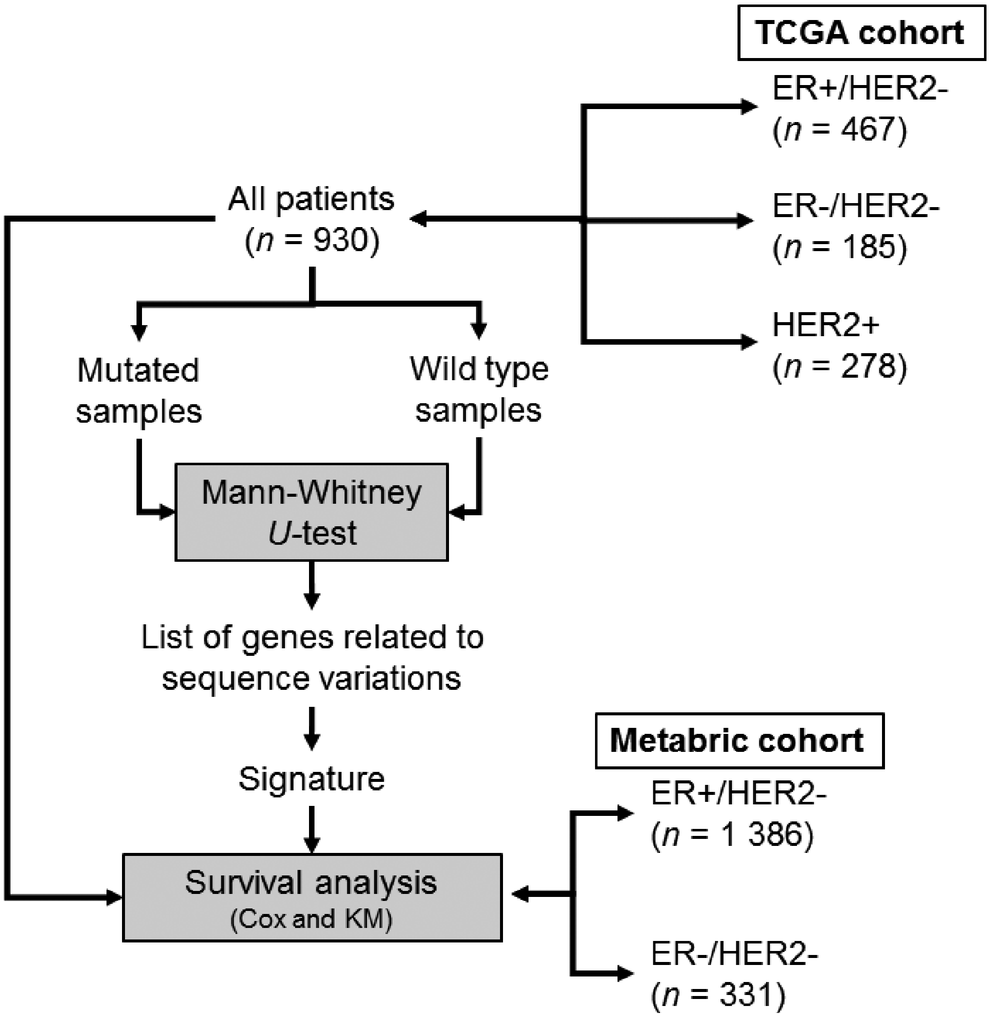

Supplementary Figure 1. Workflow of the study.

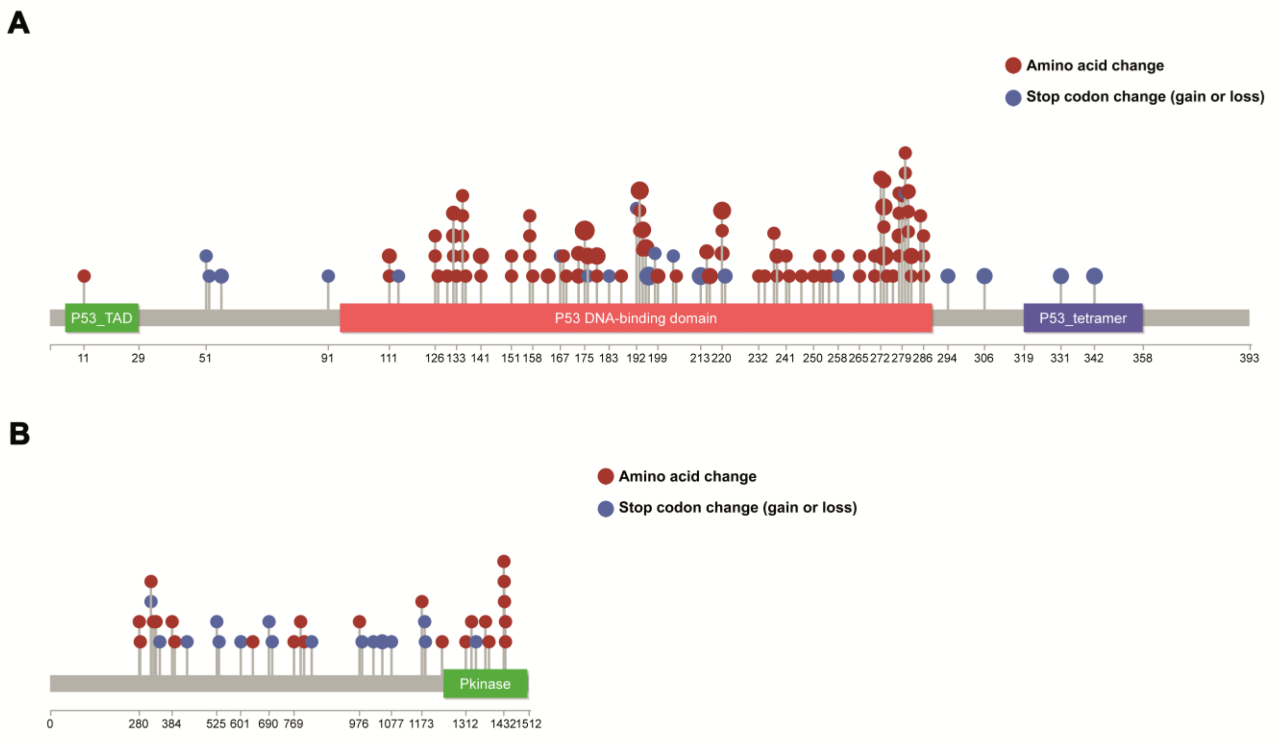

Supplementary Figure 2. Coding mutations identified in TP53 and MAP3K1 genes. Needle plots depicting the type and distribution of mutations in *TP53* (**A**) and *MAP3K1* (**B**) genes. The length of each bar represents the number of mutations identified at each position. The horizontal axis represents the amino-acid sequence of genes.

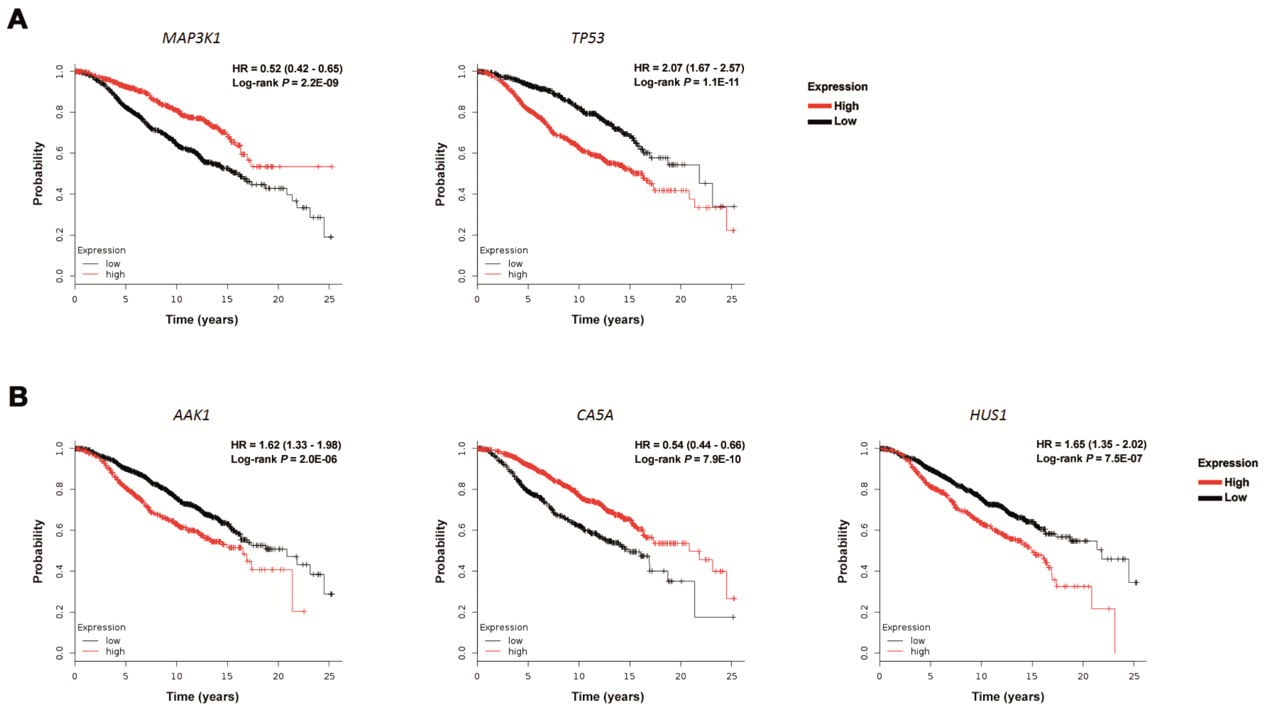

Supplementary Figure 3. Prognostic value of expression signatures correlated with genetic variants in the validation cohort. (**A**) Kaplan-Meier analysis of overall survival for the signatures associated with mutations in the coding areas of *MAP3K1* and *TP53* genes in ER-positive/HER2-negative breast cancer. (**B**) Kaplan-Meier analysis of overall survival for the signatures associated with sequence variations in the non-coding regions of *AAK1*, *CA5A*, and *HUS1* genes in ER-positive/HER2-negative breast cancer.
