# Supplementary material for: An integrative bioinformatics approach reveals coding and non-coding gene variants associated with gene expression profiles and outcome in breast cancer molecular subtypes
Source: Br J Cancer. 2018 Mar 21;118(8):1107–14. doi: 10.1038/s41416-018-0030-0 (PMC5931099; doi:10.1038/s41416-018-0030-0)
Supplement: Supplementary file 2 — Supplementary Table 1 [file 41416_2018_30_MOESM2_ESM.pdf]

**Supplementary Table 1. Clinical and pathological characteristics of breast cancer patients included in the study**	
**Clinical and pathological information**	**TCGA Dataset**
Patients (*n*)	930
Mean age (min-max)	58.3 (26 - 90)
Median follow-up (months)	31.56
**Lymph node status**	
Positive	421 (45.3%)
Negative	484 (52.0%)
Unknown	25 (2.7%)
**ER status**	
Positive	674 (72.5%)
Negative	256 (27.5%)
Unknown	-
**HER2 status**	
Positive	143 (15.4%)
Negative	787 (84.6%)
Unknown	-
**T stage**	
1	244 (26.2%)
2	539 (58.0%)
3	111 (11.9%)
4	33 (3.5%)
Unknown	3 (0.3%)
**N stage**	
0	439 (47.2%)
1	309 (33.2%)
2	103 (11.1%)
3	64 (6.9%)
Unknown	15 (1.6%)
**M stage**	
0	805 (86.6%)
1	16 (1.7%)
Unknown	109 (11.7%)
**Vital status**	
Alive	823 (88.5%)
Death	107 (11.5%)
Unknown	-

Abbreviations: ER = estrogen receptor; HER2 = human epidermal growth factor receptor 2; TCGA = The Cancer Genome Atlas.
