# Supplementary material for: An integrative bioinformatics approach reveals coding and non-coding gene variants associated with gene expression profiles and outcome in breast cancer molecular subtypes
Source: Br J Cancer. 2018 Mar 21;118(8):1107–14. doi: 10.1038/s41416-018-0030-0 (PMC5931099; doi:10.1038/s41416-018-0030-0)
Supplement: Supplementary file 3 — Supplementary Table 2 [file 41416_2018_30_MOESM3_ESM.pdf]

Supplementary Table 2. Genes with sequence variations in coding regions in at least 20 breast cancer patients				
**Gene**	**All samples ($n = 930$)**	**ER-positive/HER2-negative ($n = 467$)**	**ER-negative/HER2-negative ($n = 185$)**	**HER2-positive ($n = 278$)**
PIK3CA	32%	38%	11%	35%
TTN	25%	23%	32%	22%
TP53	24%	15%	56%	18%
MUC16	15%	12%	19%	16%
MUC4	11%	10%	11%	12%
CDC27	10%	10%	11%	8%
FLG	9%	7%	10%	11%
KMT2C	9%	8%	9%	9%
NBPF1	8%	8%	11%	6%
USH2A	8%	6%	13%	6%
HMCN1	6%	7%	6%	6%
AHNAK2	6%	5%	7%	8%
RYR2	6%	5%	7%	7%
SYNE1	6%	4%	8%	7%
NEB	6%	6%	4%	7%
MUC17	6%	6%	8%	4%
MUC12	6%	5%	8%	6%
OBSCN	5%	6%	9%	2%
GPR98	5%	6%	3%	5%
LRP2	5%	3%	8%	5%
SPTA1	5%	5%	8%	4%
DMD	5%	4%	7%	5%
DST	5%	4%	8%	5%
TPP2	5%	5%	7%	3%
TAB3	5%	6%	4%	4%
FRG1B	5%	4%	6%	5%
ABCA13	5%	5%	4%	3%
DNAH11	5%	4%	5%	6%
PKHD1L1	5%	3%	6%	5%
MUC5B	4%	4%	5%	4%
SYNE2	4%	3%	6%	5%
ZFHX4	4%	5%	5%	3%
CDH1	4%	5%	1%	5%
ASPM	4%	3%	6%	4%
CSMD3	4%	4%	7%	3%
CACNA1E	4%	3%	6%	4%
FAT3	4%	3%	9%	3%
MAP3K1	4%	5%	2%	4%
FAT1	4%	4%	3%	4%
MARK1	4%	5%	3%	3%
MKI67	4%	3%	5%	4%
APOB	4%	4%	4%	4%
CEP290	4%	3%	5%	4%
RHPN2	4%	4%	3%	4%
RYR3	4%	3%	3%	6%
USP34	4%	4%	5%	4%
DNAH5	4%	3%	5%	4%
TG	4%	4%	5%	3%
PCLO	4%	4%	2%	4%
FAM186A	4%	4%	3%	3%
FLG2	4%	3%	5%	4%
NCOR1	4%	3%	3%	6%

PRUNE2	4%	3%	6%	3%
XIRP2	4%	3%	4%	4%
DNAH17	3%	2%	8%	3%
MDN1	3%	2%	5%	5%
DNAH8	3%	2%	4%	5%
HRNR	3%	3%	6%	3%
LRP1B	3%	3%	5%	2%
MUC2	3%	3%	5%	2%
VIT	3%	4%	4%	1%
CSMD1	3%	4%	4%	2%
EYS	3%	3%	4%	3%
MACF1	3%	3%	3%	3%
MYCBP2	3%	3%	4%	3%
CENPE	3%	2%	4%	4%
DNAH14	3%	2%	5%	4%
FAM47A	3%	3%	2%	3%
HYDIN	3%	3%	3%	4%
IGFN1	3%	2%	7%	2%
TOMM70A	3%	3%	2%	4%
UBR4	3%	3%	3%	3%
ANK2	3%	3%	3%	3%
ANK3	3%	2%	3%	4%
BIRC6	3%	2%	5%	3%
DCHS2	3%	3%	4%	3%
DNAH3	3%	3%	4%	3%
DNAH9	3%	3%	4%	3%
FANCD2	3%	3%	3%	3%
HARS	3%	3%	3%	3%
KIAA1109	3%	3%	2%	4%
MXRA5	3%	2%	3%	4%
PTEN	3%	2%	5%	3%
WDR87	3%	2%	5%	2%
AHNAK	3%	3%	4%	3%
BRCA1	3%	3%	3%	2%
CSMD2	3%	3%	5%	1%
FCGBP	3%	2%	5%	3%
GPR112	3%	3%	3%	3%
LAMA1	3%	2%	4%	3%
RP1L1	3%	3%	3%	3%
SVEP1	3%	3%	3%	3%
TCHH	3%	2%	4%	4%
TENM1	3%	3%	3%	3%
VPS13C	3%	3%	3%	2%
WDR52	3%	2%	6%	3%
ADAMTS7	3%	2%	2%	4%
AHCTF1	3%	2%	3%	3%
AKAP9	3%	3%	3%	3%
ARID1A	3%	2%	2%	5%
CFH	3%	3%	3%	1%
DNAH7	3%	2%	4%	3%
F5	3%	1%	4%	4%
RELN	3%	2%	5%	3%
RGPD3	3%	2%	4%	3%
SPHKAP	3%	2%	2%	4%
VPS13B	3%	2%	3%	4%
AGGF1	3%	3%	3%	1%

BTNL8	3%	3%	3%	3%
CMYA5	3%	2%	2%	4%
CR1	3%	2%	3%	3%
CRIPAK	3%	3%	2%	3%
DNAH6	3%	2%	3%	3%
EMR2	3%	2%	3%	3%
ERBB2	3%	1%	2%	6%
FMN2	3%	2%	3%	3%
MROH2B	3%	2%	3%	3%
NOTCH2	3%	2%	2%	4%
PCNXL2	3%	2%	3%	3%
PRKDC	3%	1%	4%	4%
PTPRD	3%	2%	2%	4%
TSG101	3%	4%	2%	1%
UTRN	3%	2%	5%	3%
ALMS1	3%	1%	4%	4%
BAI3	3%	2%	3%	3%
CUBN	3%	2%	3%	3%
DYNC1H1	3%	2%	3%	4%
DYNC2H1	3%	2%	4%	2%
FRAS1	3%	1%	5%	2%
GOLGB1	3%	2%	5%	2%
HEG1	3%	1%	3%	4%
PAPPA2	3%	2%	3%	4%
PCMTD1	3%	2%	3%	3%
RPGR	3%	3%	2%	3%
TEX15	3%	2%	3%	4%
TNRC6B	3%	2%	4%	3%
ZAN	3%	3%	3%	2%
AK9	2%	3%	3%	2%
COL12A1	2%	2%	4%	2%
KMT2D	2%	2%	3%	3%
LAMA2	2%	1%	4%	3%
PKHD1	2%	1%	3%	3%
REV3L	2%	2%	3%	2%
RYR1	2%	2%	3%	3%
TRPC5	2%	2%	3%	3%
ATM	2%	2%	2%	3%
BAZ2B	2%	1%	3%	3%
C5orf42	2%	2%	3%	3%
CHD4	2%	2%	3%	2%
ERBB3	2%	2%	3%	3%
FAT2	2%	3%	2%	2%
FBN1	2%	2%	2%	3%
FREM2	2%	3%	2%	3%
KIAA1210	2%	1%	3%	3%
LRP1	2%	1%	3%	4%
MGA	2%	2%	1%	3%
MYH4	2%	2%	4%	2%
PCDH15	2%	2%	4%	2%
PREX2	2%	2%	3%	2%
RNF213	2%	1%	6%	2%
SCN10A	2%	1%	3%	4%
TBC1D32	2%	3%	1%	3%
TRIM49	2%	1%	4%	4%
UBR5	2%	2%	3%	3%

UNC13C	2%	2%	3%	2%
USP6	2%	2%	3%	2%
VPS13D	2%	2%	3%	2%
ADAMTSL3	2%	2%	2%	2%
CACNA1C	2%	1%	5%	3%
CEP350	2%	2%	2%	4%
CHD6	2%	2%	3%	2%
COL6A5	2%	2%	4%	1%
COL6A6	2%	2%	3%	1%
DYX1C1	2%	2%	3%	2%
NBPF3	2%	2%	4%	2%
OR2T34	2%	1%	3%	3%
PCNT	2%	1%	6%	1%
RAPGEF6	2%	2%	2%	3%
SACS	2%	2%	3%	2%
SEPT10	2%	2%	2%	3%
SIRPA	2%	2%	2%	3%
SRCAP	2%	2%	2%	4%
ABCA4	2%	1%	3%	3%
AP4B1	2%	2%	3%	2%
ASH1L	2%	1%	3%	3%
ATP10B	2%	2%	3%	2%
BRCA2	2%	1%	3%	3%
COL14A1	2%	2%	1%	3%
CS	2%	2%	2%	3%
DDX11	2%	2%	3%	2%
DNAH2	2%	1%	5%	1%
DNAJC13	2%	2%	2%	3%
DNHD1	2%	2%	2%	3%
FAT4	2%	1%	2%	4%
FREM1	2%	2%	3%	3%
GON4L	2%	2%	2%	3%
IGSF10	2%	1%	6%	2%
KIF4A	2%	2%	2%	3%
MAP1A	2%	2%	3%	2%
MYH6	2%	1%	3%	3%
MYH7	2%	2%	2%	3%
NBEA	2%	2%	4%	2%
NCOA6	2%	1%	2%	3%
OTOGL	2%	1%	4%	1%
PEG3	2%	1%	3%	3%
PKD1L1	2%	2%	3%	2%
SCN11A	2%	2%	4%	1%
TAF1L	2%	2%	2%	3%
