# Supplementary material for: An integrative bioinformatics approach reveals coding and non-coding gene variants associated with gene expression profiles and outcome in breast cancer molecular subtypes
Source: Br J Cancer. 2018 Mar 21;118(8):1107–14. doi: 10.1038/s41416-018-0030-0 (PMC5931099; doi:10.1038/s41416-018-0030-0)
Supplement: Supplementary file 4 — Supplementary Table 3 [file 41416_2018_30_MOESM4_ESM.pdf]

Supplementary Table 3. Genes with sequence variations in non-coding regions in at least 20 breast cancer patients

Gene	All samples ($n = 930$)	ER-positive/HER2-negative ($n = 467$)	ER-negative/HER2-negative ($n = 185$)	HER2-positive ($n = 278$)
NBPF1	44%	42%	49%	45%
TTN-AS1	32%	31%	38%	31%
BAGE2	32%	32%	32%	30%
TP53	26%	16%	58%	22%
TTN	26%	24%	29%	27%
RYR2	21%	21%	21%	20%
FRG1B	20%	19%	22%	21%
KMT2C	20%	19%	20%	22%
TPTE	19%	20%	18%	19%
PIK3CA	19%	23%	6%	19%
FLG-AS1	18%	14%	24%	20%
CROCCP2	17%	17%	22%	12%
PCDHGA1	17%	16%	15%	19%
PCDHGA2	16%	15%	15%	18%
SYNE1	16%	12%	22%	19%
EIF2B5	15%	14%	24%	12%
PCDHGA3	15%	14%	15%	18%
MUC16	15%	17%	11%	15%
GON4L	15%	15%	15%	15%
HMCN1	15%	17%	12%	13%
PCDHGB1	14%	13%	14%	16%
COL22A1	14%	14%	15%	13%
DMD	14%	13%	15%	14%
USH2A	14%	13%	12%	15%
MUC4	13%	12%	15%	14%
PCDHGA4	13%	12%	14%	15%
PKHD1L1	13%	12%	19%	12%
OR2L13	13%	13%	12%	13%
CR1	13%	11%	16%	13%
SPTA1	13%	11%	14%	14%
CROCC	12%	12%	16%	11%
FCGR3A	12%	10%	15%	15%
PCDHGB2	12%	11%	14%	14%
DNAH8	12%	10%	16%	12%
HYDIN	12%	12%	11%	13%
DMBT1	12%	11%	15%	10%
OBSCN	12%	10%	15%	11%
FCGR2B	11%	9%	14%	14%
UGT1A8	11%	10%	18%	10%
PCDHGA5	11%	10%	10%	14%
PI4KA	11%	11%	15%	9%
RYR3	11%	12%	9%	12%
SPDYE3	11%	12%	14%	9%
NEB	11%	10%	14%	12%
LRP1B	11%	11%	15%	8%
CSF2RA	11%	12%	9%	11%
CACNA1C	11%	10%	10%	12%
DNAH11	11%	10%	11%	12%
PCDHGB3	11%	9%	11%	14%
ZNF512	11%	11%	12%	9%
DDX11	11%	12%	10%	9%
HUS1	11%	10%	15%	9%
DST	10%	10%	12%	10%
NBPF14	10%	8%	15%	12%
CDC27	10%	10%	12%	9%
NIPBL	10%	9%	18%	8%
SYCP2L	10%	9%	11%	11%
ABI3BP	10%	9%	14%	10%

CACNA1E	10%	9%	14%	10%
FRAS1	10%	8%	9%	14%
PCDHGA6	10%	9%	10%	13%
UGT1A10	10%	8%	16%	9%
CEP170	10%	9%	11%	10%
DZIP3	10%	10%	9%	10%
MYO3B	10%	9%	11%	10%
SDK1	10%	8%	11%	13%
SMG1	10%	9%	12%	9%
GPR98	10%	10%	10%	9%
HBG2	10%	9%	12%	10%
SYNE2	10%	10%	9%	9%
TG	10%	10%	11%	8%
CSMD1	10%	9%	9%	12%
PLEKHB2	10%	8%	11%	11%
PKHD1	9%	9%	12%	9%
MACF1	9%	8%	10%	11%
ABCA13	9%	9%	9%	10%
PCDHGA7	9%	7%	9%	12%
LPA	9%	10%	8%	9%
PCDHGB4	9%	7%	9%	13%
PRKCA	9%	8%	10%	11%
ACSM3	9%	11%	7%	7%
ANAPC1	9%	8%	12%	8%
FANCD2	9%	7%	9%	12%
GPN1	9%	9%	11%	8%
DNAH17	9%	7%	11%	10%
GOLGB1	9%	10%	10%	6%
MUC17	9%	8%	12%	9%
PKD1L1	9%	8%	12%	8%
PREX2	9%	6%	11%	12%
UGT1A6	9%	8%	15%	7%
C1orf112	9%	8%	14%	7%
DOCK2	9%	10%	6%	9%
HDLBP	9%	10%	8%	7%
KDM2A	9%	9%	8%	9%
UGT1A7	9%	7%	15%	7%
VPS13B	9%	7%	10%	10%
VWF	9%	8%	11%	9%
DNAH9	9%	7%	11%	9%
UBR4	9%	9%	8%	9%
ASAP1	8%	7%	10%	10%
CRTC3	8%	9%	10%	7%
CSMD3	8%	9%	11%	7%
ITPR1	8%	8%	8%	10%
KIAA1109	8%	9%	6%	9%
MST1P2	8%	8%	14%	5%
MTHFD1L	8%	8%	11%	7%
MYO3A	8%	7%	11%	8%
PCDHGA8	8%	7%	9%	11%
AHNAK2	8%	7%	9%	10%
CACNA1B	8%	7%	13%	8%
LRP2	8%	8%	9%	8%
LRRC16A	8%	7%	9%	10%
DNAH5	8%	7%	12%	8%
PTPRB	8%	9%	8%	8%
DPP4	8%	9%	6%	8%
RYR1	8%	7%	10%	9%
UGT1A4	8%	7%	14%	6%
UPK3B	8%	7%	11%	8%
C16orf45	8%	9%	6%	8%
FREM1	8%	7%	10%	8%

SBF2	8%	9%	8%	7%
STAG2	8%	7%	8%	10%
SULF1	8%	9%	10%	5%
ANKRD20A5P	8%	7%	8%	9%
AVL9	8%	9%	6%	8%
CFH	8%	9%	8%	5%
DNAH3	8%	8%	7%	9%
DNAH7	8%	8%	8%	8%
ESR2	8%	9%	7%	7%
LAMA1	8%	6%	11%	9%
VWA3A	8%	8%	8%	8%
C14orf37	8%	10%	8%	5%
COL6A6	8%	8%	11%	6%
DIP2B	8%	9%	7%	6%
DNAH14	8%	5%	10%	10%
DOCK4	8%	6%	12%	8%
MYH3	8%	8%	10%	6%
TMEM50A	8%	9%	8%	6%
UGGT1	8%	8%	8%	8%
USP34	8%	7%	11%	8%
DNAH10	8%	6%	12%	8%
FIP1L1	8%	7%	5%	11%
LAMA2	8%	6%	14%	7%
LIPE-AS1	8%	6%	10%	9%
MYOM1	8%	6%	9%	9%
PLB1	8%	7%	11%	7%
PTPRD	8%	9%	5%	8%
TPTE2	8%	6%	8%	10%
TPTE2P6	8%	8%	8%	8%
ANKRD36	8%	6%	11%	8%
CDH1	8%	8%	7%	7%
CDKL5	8%	7%	6%	9%
DNAH6	8%	7%	9%	7%
MSTO2P	8%	5%	11%	10%
PDS5B	8%	7%	9%	7%
PDXDC1	8%	5%	12%	10%
RPH3A	8%	8%	9%	6%
SOD2	8%	7%	9%	7%
BAGE5	8%	7%	9%	8%
BCAS1	8%	9%	6%	5%
CUBN	8%	7%	8%	9%
MED12L	8%	6%	10%	8%
PDXDC2P	8%	7%	8%	8%
RPTOR	8%	7%	9%	8%
STAG3L2	8%	7%	7%	9%
SVIL	8%	7%	8%	8%
KALRN	7%	6%	8%	10%
MROH7-TTC4	7%	9%	4%	8%
NFASC	7%	7%	9%	8%
SACS	7%	8%	9%	6%
AAK1	7%	9%	8%	5%
ASXL3	7%	7%	9%	6%
ATP8A2	7%	6%	9%	8%
COL11A1	7%	7%	8%	8%
CSMD2	7%	7%	12%	5%
DYNC2H1	7%	7%	10%	6%
FER1L6	7%	7%	6%	8%
VIT	7%	8%	9%	5%
WDR52	7%	6%	10%	8%
COL4A5	7%	7%	7%	7%
CYP3A4	7%	6%	6%	9%
DDC	7%	9%	7%	5%

KRT26	7%	8%	9%	5%
PTCH1	7%	7%	8%	7%
RBX1	7%	8%	7%	6%
SLC4A10	7%	8%	6%	7%
TPR	7%	5%	9%	10%
AKAP13	7%	6%	10%	7%
CACNA2D1	7%	8%	10%	4%
KDM5B	7%	7%	8%	7%
PKD2L2	7%	9%	5%	5%
PRKDC	7%	7%	8%	8%
SP140	7%	7%	4%	9%
DPP10	7%	6%	10%	6%
GOPC	7%	7%	8%	7%
TPP2	7%	7%	9%	6%
CEP112	7%	8%	6%	5%
COL3A1	7%	7%	8%	6%
CPNE4	7%	6%	8%	8%
CTNNA2	7%	7%	9%	6%
DPY19L2	7%	8%	8%	5%
EPB41L4A	7%	8%	5%	6%
LAMA3	7%	5%	12%	6%
MST1L	7%	7%	8%	7%
NF1	7%	6%	7%	8%
PCDHGA9	7%	6%	6%	9%
SYCP2	7%	7%	5%	8%
UBR5	7%	4%	9%	10%
ULK4	7%	7%	8%	6%
USP9X	7%	7%	6%	7%
UTRN	7%	7%	8%	6%
ANK2	7%	6%	8%	6%
CACNA1A	7%	7%	7%	6%
CACNA1D	7%	7%	8%	6%
LYST	7%	5%	11%	7%
MYCBP2	7%	6%	9%	6%
NAA38	7%	7%	8%	6%
SLC13A1	7%	6%	7%	7%
SLC26A5	7%	7%	9%	5%
ANXA6	7%	6%	8%	7%
CBWD1	7%	6%	6%	8%
CEP128	7%	6%	7%	7%
FASTKD1	7%	6%	9%	6%
MDN1	7%	4%	11%	8%
MROH2B	7%	6%	8%	7%
PCDH15	7%	5%	11%	6%
PLXNC1	7%	7%	9%	4%
POTEH	7%	7%	6%	6%
PRKAG2	7%	5%	10%	8%
RNF213	7%	6%	11%	5%
FMN2	7%	7%	5%	6%
PPP1R12A	7%	6%	8%	6%
RANBP2	7%	5%	7%	8%
SNAP25-AS1	7%	7%	4%	7%
WDR63	7%	6%	7%	6%
ABCA9	6%	5%	9%	8%
ANKRD30A	6%	6%	9%	5%
ASPM	6%	6%	8%	7%
C20orf26	6%	5%	8%	8%
CCDC121	6%	7%	8%	5%
COL4A6	6%	6%	7%	7%
FN1	6%	7%	8%	5%
FRA10AC1	6%	7%	7%	5%
GREB1	6%	6%	8%	6%

HOOK2	6%	6%	8%	7%
MROH8	6%	6%	8%	6%
PCDHGB6	6%	5%	6%	9%
RAB3GAP2	6%	6%	6%	6%
RGL1	6%	6%	8%	7%
RGPD3	6%	6%	6%	7%
TPO	6%	6%	9%	6%
ZSCAN5A	6%	6%	6%	8%
AGAP5	6%	6%	7%	6%
AXDND1	6%	5%	8%	8%
COL27A1	6%	6%	9%	5%
CR1L	6%	5%	8%	8%
DGKI	6%	6%	8%	6%
KIF21B	6%	5%	8%	7%
NBPF3	6%	5%	8%	8%
PCDHGA10	6%	5%	6%	9%
PLCB1	6%	6%	8%	6%
TBC1D5	6%	6%	5%	8%
TRIM5	6%	6%	5%	7%
TTC27	6%	6%	5%	7%
USP32	6%	6%	6%	7%
A2ML1	6%	6%	7%	6%
COL7A1	6%	5%	11%	6%
DCDC1	6%	4%	9%	8%
FCGR2C	6%	4%	8%	8%
FLG	6%	6%	4%	9%
GARNL3	6%	7%	5%	5%
SNX29P2	6%	7%	4%	6%
VPS8	6%	6%	8%	6%
ADAM32	6%	6%	7%	6%
ANK1	6%	5%	8%	7%
ANO3	6%	6%	4%	9%
CCDC144A	6%	5%	8%	7%
CCDC39	6%	5%	5%	9%
FAM160B1	6%	6%	9%	5%
GML	6%	6%	5%	7%
IQGAP1	6%	4%	8%	8%
KCNU1	6%	6%	8%	5%
LEF1	6%	6%	6%	6%
METTL13	6%	6%	6%	6%
MMP26	6%	5%	5%	9%
MSTO1	6%	3%	10%	8%
PDCD11	6%	6%	6%	6%
STAB2	6%	5%	6%	8%
UGT1A3	6%	6%	9%	5%
WDFY4	6%	5%	8%	7%
ZAN	6%	6%	8%	6%
ACACA	6%	4%	5%	10%
AIM1	6%	6%	7%	6%
AKAP9	6%	5%	8%	6%
ANK3	6%	4%	6%	9%
ATP8B2	6%	4%	8%	8%
DNAH2	6%	6%	6%	6%
DYSF	6%	6%	8%	5%
HNRNPU-AS1	6%	6%	7%	6%
MERTK	6%	6%	6%	6%
NBPF12	6%	6%	6%	6%
PRUNE	6%	6%	7%	5%
SORCS1	6%	5%	6%	8%
TTC34	6%	7%	6%	5%
ZFAT	6%	7%	7%	4%
BAZ2B	6%	6%	9%	4%

CEP290	6%	6%	6%	5%
CNGB3	6%	6%	5%	7%
HERC2P4	6%	6%	5%	7%
IGF2R	6%	6%	6%	6%
RRN3P2	6%	6%	7%	5%
SCFD1	6%	6%	6%	6%
TBCD	6%	5%	7%	7%
TRIO	6%	5%	4%	8%
AHCTF1	6%	5%	6%	7%
AK9	6%	6%	5%	6%
CHD6	6%	6%	9%	4%
COL5A1	6%	5%	6%	7%
DDX12P	6%	5%	7%	6%
FCGR2A	6%	4%	7%	7%
HBE1	6%	4%	9%	6%
MUC5B	6%	5%	6%	7%
MYH4	6%	6%	6%	5%
RGSL1	6%	5%	6%	6%
RHPN2	6%	6%	4%	6%
SAE1	6%	6%	5%	5%
SMEK1	6%	6%	5%	6%
TBC1D3P1-DHX40P1	6%	6%	4%	6%
UNC13C	6%	5%	6%	6%
ZDHHC11	6%	6%	9%	4%
ANKRD20A9P	6%	5%	6%	6%
ARHGEF11	6%	5%	6%	6%
ATAD2B	6%	6%	7%	5%
C5orf42	6%	4%	9%	6%
CDH23	6%	6%	6%	4%
COL19A1	6%	5%	6%	7%
DENND4A	6%	6%	4%	5%
DEPDC5	6%	5%	6%	6%
EMR2	6%	4%	8%	6%
FBN1	6%	5%	4%	8%
FEZ2	6%	6%	4%	6%
FOLH1	6%	5%	6%	6%
GPATCH1	6%	6%	8%	4%
HNRNPU	6%	5%	8%	6%
ITPR3	6%	4%	6%	8%
LY75	6%	6%	7%	4%
MAGI1	6%	5%	8%	5%
MED12	6%	5%	6%	6%
MYH1	6%	6%	7%	4%
NCOR1	6%	4%	6%	8%
NPR2	6%	7%	4%	5%
NXF5	6%	5%	6%	7%
PIEZO2	6%	5%	7%	6%
RIMS2	6%	5%	8%	5%
SRGAP3	6%	6%	5%	5%
STAG3	6%	4%	8%	6%
THOC2	6%	5%	4%	8%
UBR3	6%	6%	6%	5%
UNC13A	6%	6%	8%	4%
ABCA8	6%	7%	4%	4%
ADAMTS7	6%	5%	5%	7%
AMPH	6%	6%	8%	3%
ASPH	6%	4%	6%	7%
ATM	6%	5%	6%	6%
CENPF	6%	6%	5%	5%
CLTC	6%	5%	8%	5%
COG3	6%	7%	6%	4%
COL12A1	6%	4%	9%	6%

COL13A1	6%	6%	8%	4%
COL14A1	6%	4%	6%	8%
DMGDH	6%	5%	5%	6%
DNM3	6%	5%	7%	5%
HTT	6%	5%	5%	8%
IARS	6%	6%	6%	5%
ITPR2	6%	6%	5%	5%
KIAA1217	6%	4%	8%	6%
LRRK1	6%	5%	7%	5%
NTRK1	6%	4%	9%	6%
PNPT1	6%	5%	8%	5%
PTPRQ	6%	5%	7%	6%
TAB3	6%	6%	4%	5%
TENM1	6%	5%	6%	6%
TYW1B	6%	4%	5%	8%
VPS13D	6%	5%	7%	5%
ZNF142	6%	5%	6%	6%
ZNF277	6%	6%	8%	4%
AR	5%	5%	4%	7%
ASH1L	5%	5%	6%	6%
ATP8A1	5%	5%	8%	4%
DOCK11	5%	6%	5%	6%
FCGBP	5%	4%	7%	6%
MIA3	5%	4%	6%	7%
MMP21	5%	6%	6%	4%
MYH15	5%	5%	8%	5%
MYLK	5%	5%	8%	5%
NOTCH2	5%	4%	6%	8%
OPA1	5%	5%	7%	5%
PKD1L2	5%	4%	9%	6%
PSG8	5%	5%	4%	8%
PTPRN2	5%	5%	6%	6%
RAB11A	5%	6%	4%	5%
RELN	5%	4%	8%	5%
SMG7	5%	4%	6%	7%
UBE2D3	5%	6%	4%	5%
ANKRA2	5%	6%	4%	4%
ANKRD30B	5%	5%	8%	5%
ART3	5%	5%	4%	6%
ASTN1	5%	5%	5%	6%
CACNA2D3	5%	5%	5%	6%
CBX5	5%	6%	6%	4%
CNOT1	5%	6%	4%	5%
DCC	5%	5%	5%	6%
DNAJC13	5%	6%	5%	5%
FBN2	5%	5%	5%	6%
GRIA3	5%	5%	5%	7%
IARS2	5%	5%	8%	4%
IQGAP2	5%	6%	5%	5%
LY75-CD302	5%	6%	6%	3%
MYH11	5%	4%	7%	6%
MYO5A	5%	5%	4%	7%
MYO5B	5%	4%	6%	7%
NEBL	5%	6%	5%	5%
NOMO1	5%	5%	5%	6%
OTOA	5%	4%	7%	6%
PACRGL	5%	6%	6%	4%
PHF12	5%	6%	4%	5%
SLC12A6	5%	5%	5%	6%
TTC40	5%	5%	8%	5%
USP6	5%	4%	6%	6%
WDR11	5%	5%	6%	5%

ABL2	5%	5%	5%	5%
AKNAD1	5%	3%	8%	6%
ANKEF1	5%	6%	3%	5%
ANO1	5%	4%	6%	7%
BANK1	5%	6%	4%	4%
CDS2	5%	4%	6%	7%
CHD5	5%	4%	7%	6%
CLEC16A	5%	5%	5%	6%
COL1A2	5%	4%	5%	7%
DLEC1	5%	6%	5%	4%
DOCK1	5%	5%	6%	5%
ERMN	5%	6%	5%	4%
FANCA	5%	5%	6%	5%
FER1L6-AS2	5%	5%	3%	7%
FLNB	5%	5%	7%	5%
GALNT8	5%	6%	4%	6%
KCNN3	5%	4%	6%	7%
KIAA1429	5%	6%	3%	6%
LRP1	5%	3%	6%	8%
NAV2	5%	4%	6%	6%
PCNT	5%	3%	10%	6%
RC3H1	5%	4%	5%	7%
SMARCA4	5%	6%	5%	4%
TAF1	5%	5%	5%	6%
TEP1	5%	6%	6%	4%
TTLL5	5%	6%	3%	6%
WDFY3	5%	4%	5%	6%
ACSM1	5%	6%	4%	4%
ADAMTSL3	5%	4%	6%	6%
ANTXR1	5%	5%	8%	4%
ATAD2	5%	4%	7%	5%
BAI3	5%	5%	4%	6%
CACNA1S	5%	4%	6%	5%
COL4A2	5%	4%	8%	5%
DYNC1H1	5%	4%	4%	7%
EP400	5%	4%	7%	6%
EPHB1	5%	5%	7%	4%
GTF3C3	5%	6%	5%	3%
HECW1	5%	4%	6%	5%
ITGA1	5%	5%	4%	6%
IWS1	5%	5%	6%	4%
KIAA1257	5%	6%	5%	4%
KIF21A	5%	6%	5%	4%
KIF2A	5%	6%	4%	5%
MTR	5%	3%	6%	8%
NBEA	5%	4%	9%	4%
NPHP3	5%	4%	8%	6%
NRXN3	5%	4%	6%	6%
SI	5%	5%	7%	4%
SMARCA2	5%	6%	5%	5%
SYK	5%	5%	4%	6%
TLE4	5%	6%	3%	6%
TRPA1	5%	5%	5%	6%
TRPC4AP	5%	7%	3%	4%
ZC3HAV1	5%	5%	5%	5%
ADAMTSL1	5%	6%	3%	5%
ADCY2	5%	4%	5%	6%
AGFG1	5%	7%	5%	2%
ANKRD20A11P	5%	4%	8%	6%
ANKRD20A8P	5%	4%	6%	6%
ATR	5%	3%	9%	6%
BIRC6	5%	4%	6%	6%

CASK	5%	4%	6%	5%
CIT	5%	4%	3%	8%
COL24A1	5%	4%	5%	6%
COL5A3	5%	5%	5%	5%
EFCAB12	5%	4%	5%	6%
ENPEP	5%	5%	4%	5%
EPDR1	5%	5%	7%	4%
FAM118A	5%	6%	2%	5%
GRK4	5%	6%	4%	5%
IFT122	5%	3%	9%	5%
INADL	5%	4%	7%	6%
IQCK	5%	7%	3%	3%
KIF9	5%	4%	6%	6%
LCP2	5%	5%	3%	6%
LGR5	5%	5%	7%	4%
LYRM1	5%	5%	6%	5%
MCF2L2	5%	3%	8%	6%
MKI67	5%	4%	5%	7%
PRUNE2	5%	5%	8%	4%
SDHAP1	5%	5%	4%	5%
SEC16B	5%	5%	5%	5%
SLC15A2	5%	4%	6%	6%
UNC5D	5%	4%	6%	7%
VWA8	5%	5%	7%	4%
ACSM5	5%	4%	5%	6%
ANKRD30BL	5%	4%	6%	6%
ANKRD36BP2	5%	4%	5%	6%
C7orf63	5%	4%	5%	6%
CADPS	5%	6%	2%	5%
CDC42BPA	5%	4%	4%	7%
COL4A3	5%	5%	5%	4%
F5	5%	4%	5%	7%
FAT3	5%	5%	5%	5%
HEG1	5%	4%	5%	6%
HJURP	5%	5%	5%	5%
HNRNPA1	5%	6%	6%	3%
IGFN1	5%	4%	7%	5%
IQGAP3	5%	4%	8%	5%
KRT86	5%	4%	7%	5%
MLLT4	5%	5%	4%	5%
NBAS	5%	4%	5%	7%
NBPF22P	5%	5%	5%	5%
NRAP	5%	4%	5%	5%
OVCH1-AS1	5%	6%	3%	4%
PCNXL2	5%	3%	5%	7%
PLG	5%	4%	4%	6%
PLXNA2	5%	3%	8%	6%
PMFBP1	5%	4%	6%	5%
RARA	5%	5%	5%	5%
SUPT6H	5%	4%	6%	6%
SZT2	5%	4%	10%	4%
THSD7B	5%	4%	5%	6%
TRPM6	5%	4%	6%	6%
TTC3	5%	4%	8%	4%
U2SURP	5%	4%	5%	5%
UNC80	5%	4%	6%	5%
VPS13C	5%	5%	6%	4%
ZNF551	5%	4%	4%	6%
ZZEF1	5%	6%	4%	4%
ARHGAP6	5%	5%	3%	5%
BPIFB2	5%	4%	6%	6%
CARD11	5%	5%	5%	4%

CCDC180	5%	4%	5%	6%
CCDC7	5%	5%	5%	5%
COPA	5%	4%	7%	5%
CYP2A6	5%	5%	5%	4%
DGKB	5%	5%	2%	6%
DLG2	5%	3%	8%	5%
EDEM2	5%	3%	5%	7%
EGFR	5%	4%	4%	6%
EML5	5%	4%	4%	6%
ERI2	5%	6%	4%	4%
FRY	5%	4%	8%	4%
GABRA2	5%	4%	5%	5%
GIGYF2	5%	5%	3%	5%
GTF3C1	5%	5%	5%	5%
KIF1B	5%	4%	6%	5%
MAP2	5%	4%	5%	6%
MUC2	5%	5%	5%	5%
MYO10	5%	3%	7%	6%
MYOF	5%	4%	5%	6%
MYT1L	5%	4%	5%	6%
NSD1	5%	4%	7%	4%
PFKFB2	5%	3%	6%	6%
PLAA	5%	4%	6%	5%
PSME4	5%	5%	5%	5%
PTPRM	5%	4%	5%	6%
RCHY1	5%	5%	6%	4%
RIF1	5%	4%	6%	5%
SLC17A5	5%	5%	6%	4%
SLIT2	5%	5%	3%	6%
SPEF2	5%	4%	7%	5%
SRCAP	5%	4%	5%	6%
TRAPPC9	5%	4%	10%	3%
ZNF98	5%	4%	4%	7%
A2M	5%	3%	7%	5%
AK7	5%	4%	6%	5%
ARID4B	5%	5%	5%	4%
BCL2L14	5%	4%	6%	5%
BMS1P20	5%	4%	5%	6%
BRWD3	5%	5%	3%	5%
CPNE3	5%	5%	5%	4%
EFCAB6	5%	4%	10%	3%
GPR112	5%	3%	5%	7%
GRM7	5%	4%	6%	4%
IL1RAP	5%	4%	6%	5%
ITK	5%	3%	4%	8%
KCNT2	5%	4%	4%	6%
KIAA0430	5%	5%	3%	5%
LRRK2	5%	3%	6%	6%
MORC4	5%	6%	3%	4%
MYH9	5%	4%	6%	5%
MYO18A	5%	4%	6%	5%
PDZD7	5%	5%	6%	3%
POLA1	5%	4%	5%	6%
RASSF2	5%	5%	4%	5%
RBFOX1	5%	5%	5%	4%
SDHA	5%	4%	6%	5%
STAT3	5%	4%	6%	6%
TNC	5%	5%	3%	6%
WDR64	5%	4%	4%	6%
ZNF492	5%	4%	7%	4%
ACTR2	5%	4%	6%	4%
ACTR3B	5%	4%	3%	6%

ADCY10	5%	3%	7%	5%
ATP1A4	5%	4%	6%	5%
BRCA1	5%	4%	6%	5%
CCDC93	5%	4%	5%	5%
CDK5RAP2	5%	4%	5%	5%
CENPE	5%	3%	6%	6%
DENND1B	5%	4%	3%	6%
DNAJC18	5%	6%	5%	3%
ERBB3	5%	4%	4%	5%
ESRP1	5%	6%	2%	4%
FANCI	5%	4%	4%	6%
IPO9-AS1	5%	4%	4%	6%
ITGAL	5%	5%	4%	4%
MAGI3	5%	4%	7%	4%
PARP1	5%	3%	6%	5%
PCDHGA11	5%	3%	5%	6%
PCDHGB7	5%	3%	4%	7%
PDHA1	5%	4%	4%	6%
POLR1A	5%	4%	6%	4%
RALGAPA2	5%	5%	6%	4%
ST3GAL3	5%	4%	5%	6%
TLN2	5%	4%	7%	4%
TMPRSS15	5%	3%	7%	6%
UNC79	5%	5%	5%	4%
UTP20	5%	5%	4%	4%
VAPA	5%	4%	5%	5%
XRN1	5%	3%	6%	5%
ADAMTS20	5%	4%	6%	4%
ALMS1	5%	3%	6%	6%
C2CD3	5%	3%	6%	6%
C3	5%	4%	5%	5%
CFHR2	5%	4%	5%	5%
COL6A5	5%	4%	7%	4%
CPED1	5%	3%	5%	6%
DOCK5	5%	3%	5%	6%
DOCK8	5%	4%	5%	4%
EDEM3	5%	6%	3%	4%
ENPP1	5%	4%	5%	5%
ERBB2	5%	3%	2%	10%
EXOSC10	5%	4%	6%	4%
FER1L5	5%	3%	10%	4%
GSTM5	5%	3%	8%	5%
H2AFV	5%	4%	6%	4%
INTS4	5%	4%	4%	6%
KIAA0368	5%	3%	5%	6%
KIAA0922	5%	4%	6%	4%
KLC4	5%	4%	5%	5%
LOXHD1	5%	4%	8%	4%
OVCH1	5%	6%	3%	3%
PCSK5	5%	4%	4%	5%
POLE	5%	4%	5%	4%
PRB1	5%	4%	4%	6%
SLC4A4	5%	4%	3%	5%
SLC6A1	5%	4%	3%	7%
SNRNP200	5%	3%	6%	5%
STIL	5%	5%	6%	3%
TBX20	5%	4%	6%	5%
TTC4	5%	6%	2%	4%
TXK	5%	4%	3%	6%
ACAT2	4%	3%	6%	5%
ACIN1	4%	4%	4%	6%
ANKRD19P	4%	4%	6%	4%

ANKRD36B	4%	3%	6%	5%
AP1B1	4%	3%	8%	4%
BMS1	4%	4%	4%	6%
CASP1	4%	3%	9%	5%
CDK11A	4%	3%	5%	6%
CEACAM1	4%	3%	6%	5%
CEP120	4%	4%	5%	5%
CHD4	4%	3%	8%	4%
CKAP5	4%	3%	6%	5%
CNTN4	4%	4%	4%	5%
CNTNAP2	4%	4%	6%	4%
COG7	4%	5%	4%	4%
COL15A1	4%	4%	3%	6%
COL6A3	4%	3%	7%	5%
CPS1	4%	4%	5%	5%
CRAT	4%	4%	4%	5%
CRLF3	4%	5%	3%	5%
CTTNBP2	4%	3%	4%	7%
CYP4F2	4%	4%	3%	6%
DCHS2	4%	4%	6%	3%
DHRSX	4%	4%	4%	6%
DNAH1	4%	4%	4%	5%
DOCK9	4%	4%	8%	3%
DPP6	4%	4%	3%	6%
DPY19L2P1	4%	3%	6%	5%
DYX1C1-CCPG1	4%	5%	5%	3%
EPG5	4%	5%	3%	5%
EPRS	4%	5%	4%	4%
EYS	4%	3%	8%	5%
GCC2	4%	4%	7%	4%
IL16	4%	4%	5%	4%
KMT2D	4%	3%	3%	7%
LPAL2	4%	3%	5%	6%
MARCH1	4%	3%	5%	6%
MTSS1	4%	4%	4%	5%
MYH13	4%	5%	5%	4%
NAP1L4	4%	5%	3%	5%
NCOA3	4%	4%	5%	4%
NCOR2	4%	4%	5%	4%
NEK10	4%	4%	3%	6%
NTRK3	4%	5%	3%	4%
NUP210L	4%	3%	8%	5%
NVL	4%	3%	5%	7%
PIWIL1	4%	3%	5%	6%
POTEG	4%	4%	6%	4%
PPHLN1	4%	4%	4%	5%
PRSS37	4%	4%	5%	4%
RNF123	4%	4%	4%	4%
SCN5A	4%	4%	5%	4%
SETD2	4%	3%	5%	5%
SIPA1L2	4%	4%	5%	4%
SLC16A10	4%	6%	4%	3%
SLC44A5	4%	3%	8%	4%
SORT1	4%	5%	3%	4%
SPAG5	4%	4%	3%	6%
STK31	4%	4%	4%	5%
SV2C	4%	5%	4%	4%
TRIM37	4%	5%	5%	4%
UQCRC2	4%	4%	4%	5%
ABCA4	4%	4%	5%	4%
ABCC1	4%	4%	4%	4%
B4GALT5	4%	5%	3%	4%

CCDC146	4%	4%	3%	5%
CLSPN	4%	4%	5%	3%
COG5	4%	3%	6%	5%
DGKH	4%	4%	3%	5%
DHX29	4%	5%	1%	5%
DIAPH2	4%	4%	4%	5%
FAM186A	4%	5%	3%	4%
FARP1	4%	3%	9%	3%
FBXO11	4%	3%	5%	5%
FHOD3	4%	4%	7%	3%
FRYL	4%	4%	5%	5%
GBP2	4%	3%	8%	4%
GGA3	4%	5%	5%	3%
GRIP1	4%	4%	3%	5%
HEPHL1	4%	4%	5%	4%
IL1R2	4%	5%	3%	4%
ITIH5	4%	3%	6%	5%
KCNAB1	4%	4%	5%	4%
LRPPRC	4%	3%	5%	5%
LRRC7	4%	3%	6%	6%
MAOB	4%	5%	4%	4%
MROH7	4%	4%	3%	5%
MYBPC1	4%	4%	6%	4%
MYO16	4%	4%	5%	4%
MYO18B	4%	3%	8%	4%
NCAPH	4%	4%	4%	4%
OR4N2	4%	3%	3%	7%
OTOGL	4%	3%	5%	5%
PLD5	4%	5%	3%	3%
PLEKHH2	4%	4%	3%	5%
PLXNA4	4%	6%	3%	3%
POLR2J4	4%	4%	3%	6%
PPFIBP1	4%	5%	4%	4%
RALGPS2	4%	4%	4%	5%
RGS7	4%	4%	4%	5%
ROCK1	4%	4%	4%	5%
RPS6KA2	4%	4%	5%	4%
SDAD1	4%	4%	6%	4%
SLC9C2	4%	5%	2%	5%
SORBS3	4%	4%	5%	3%
SP100	4%	3%	6%	5%
SPINK5	4%	4%	5%	5%
STON1-GTF2A1L	4%	3%	6%	5%
STRADB	4%	4%	4%	5%
SVEP1	4%	4%	4%	4%
TENM2	4%	5%	4%	4%
TIAM1	4%	3%	4%	8%
TLK2	4%	3%	3%	6%
TNNI3K	4%	2%	6%	6%
UBR2	4%	3%	5%	5%
USP43	4%	4%	4%	5%
VPS53	4%	5%	3%	4%
WASH2P	4%	4%	3%	6%
ABCA12	4%	3%	6%	5%
ACTN2	4%	3%	5%	6%
AGBL1	4%	3%	6%	5%
ATP13A3	4%	3%	8%	4%
CREBBP	4%	5%	4%	4%
ENPP2	4%	4%	5%	4%
FAM135B	4%	3%	5%	5%
FPGT-TNNI3K	4%	3%	6%	6%
FRMPD2	4%	5%	4%	3%

GAD2	4%	4%	5%	4%
GCNT2	4%	3%	6%	5%
GPR75-ASB3	4%	3%	8%	4%
HECTD4	4%	3%	5%	6%
IFI44L	4%	4%	5%	5%
KIF13A	4%	3%	5%	6%
KIF26B	4%	3%	4%	5%
KNTC1	4%	3%	5%	6%
KSR2	4%	4%	4%	5%
LRCH3	4%	3%	4%	6%
MAP3K1	4%	6%	1%	3%
MECOM	4%	3%	6%	5%
MLLT10P1	4%	4%	4%	5%
MYO5C	4%	4%	4%	5%
NALCN	4%	4%	3%	4%
NAT10	4%	4%	5%	4%
NPC1	4%	4%	6%	4%
NUP188	4%	4%	2%	6%
PDE1C	4%	4%	3%	4%
PDZD2	4%	3%	3%	7%
PGLYRP4	4%	4%	5%	3%
PHKA2	4%	5%	4%	4%
PIK3C2B	4%	3%	6%	6%
PLD1	4%	4%	4%	5%
PPFIA4	4%	2%	7%	6%
PPP1R12B	4%	4%	3%	5%
PTPN14	4%	3%	7%	5%
PTPRZ1	4%	3%	4%	6%
ROS1	4%	5%	3%	4%
SDK2	4%	3%	5%	6%
SGSM1	4%	4%	5%	5%
SLC12A5	4%	3%	5%	5%
SLC28A2	4%	3%	4%	7%
SLC44A3	4%	4%	5%	4%
SLCO1B7	4%	3%	5%	5%
SMCHD1	4%	4%	4%	5%
TLN1	4%	4%	5%	4%
TMEM14B	4%	3%	4%	5%
TNS4	4%	4%	3%	5%
TRAF3IP3	4%	4%	4%	4%
UBA6-AS1	4%	4%	4%	5%
UBAP2	4%	2%	6%	6%
USP8	4%	4%	3%	5%
VPS13A	4%	3%	5%	5%
WNK1	4%	3%	6%	4%
ALK	4%	4%	2%	5%
AQP7	4%	4%	4%	4%
ARFGEF1	4%	3%	5%	5%
ARHGAP29	4%	4%	4%	4%
ATP2A2	4%	4%	4%	4%
CASC1	4%	4%	4%	4%
CCDC144CP	4%	3%	5%	5%
CDH12	4%	3%	5%	4%
CDH2	4%	5%	4%	3%
CENPP	4%	3%	5%	6%
CRB1	4%	4%	4%	5%
CSPP1	4%	3%	5%	5%
DGKG	4%	3%	3%	7%
DSCAM	4%	3%	5%	5%
ERAP1	4%	4%	5%	3%
FBN3	4%	3%	4%	6%
HCLS1	4%	4%	6%	3%

HERC1	4%	4%	5%	4%
JAKMIP3	4%	3%	5%	5%
KRT6B	4%	3%	4%	6%
LAMB1	4%	4%	5%	4%
LIMCH1	4%	3%	5%	4%
LRP6	4%	3%	6%	4%
MAP3K15	4%	4%	3%	5%
MROH5	4%	3%	6%	4%
MRPS17	4%	4%	5%	3%
MYH6	4%	2%	5%	6%
MYH7	4%	3%	4%	5%
MYO1B	4%	3%	6%	4%
MYO7B	4%	4%	5%	4%
NCAPD2	4%	4%	3%	4%
NFKB1	4%	3%	5%	4%
NIN	4%	3%	4%	6%
ODF2L	4%	4%	5%	4%
PCLO	4%	3%	5%	5%
PPFIA1	4%	4%	5%	4%
PPFIBP2	4%	4%	4%	4%
PSG9	4%	4%	4%	4%
RABGAP1L	4%	3%	5%	5%
REV3L	4%	4%	5%	4%
RIMS1	4%	4%	6%	3%
ROBO1	4%	3%	6%	4%
RPGR	4%	4%	4%	5%
SENP6	4%	4%	7%	3%
SLC5A1	4%	5%	3%	3%
SLCO1B3	4%	3%	5%	5%
SUN5	4%	3%	5%	5%
TEX14	4%	3%	5%	5%
TMTC1	4%	4%	4%	5%
TNRC6B	4%	3%	5%	5%
TOPBP1	4%	4%	6%	3%
TTC7A	4%	4%	6%	3%
UBAP2L	4%	3%	3%	7%
XIRP2	4%	3%	6%	5%
XPO5	4%	3%	6%	5%
ZBTB8OS	4%	5%	2%	4%
ZRANB3	4%	4%	4%	3%
ABCB1	4%	2%	6%	5%
ABCC3	4%	4%	4%	5%
ACACB	4%	3%	3%	6%
ADAM19	4%	4%	3%	4%
AGAP11	4%	4%	4%	4%
ANKRD42	4%	4%	4%	4%
AP4B1-AS1	4%	3%	6%	4%
ARFGEF2	4%	3%	5%	5%
ASB3	4%	3%	7%	4%
ATP13A4	4%	4%	3%	5%
AUTS2	4%	4%	3%	5%
BTNL8	4%	4%	4%	4%
BZRAP1	4%	4%	5%	4%
CACNA1G	4%	3%	4%	6%
CD163L1	4%	3%	6%	4%
CEP192	4%	4%	4%	4%
COL4A1	4%	3%	4%	5%
CYP2C19	4%	4%	2%	6%
DNHD1	4%	3%	5%	5%
ERCC6	4%	4%	5%	4%
FNDC1	4%	3%	5%	5%
FOCAD	4%	2%	8%	4%

GABRB3	4%	4%	1%	5%
GOLGA6A	4%	3%	6%	4%
HARS	4%	4%	4%	5%
HDAC1	4%	5%	3%	4%
HSD17B7	4%	2%	5%	6%
HSPG2	4%	3%	5%	5%
IL9R	4%	3%	6%	4%
KIAA1432	4%	4%	4%	3%
LIN9	4%	3%	5%	4%
LPHN2	4%	3%	4%	5%
LRBA	4%	4%	3%	4%
MARS	4%	3%	6%	4%
MCF2	4%	3%	5%	6%
METTL5	4%	4%	5%	4%
MORC1	4%	4%	5%	4%
MPP6	4%	5%	5%	2%
NBPF9	4%	3%	6%	5%
NPHP1	4%	4%	4%	4%
NRP2	4%	3%	4%	5%
NUP98	4%	3%	4%	5%
PAPPA2	4%	2%	10%	3%
PDK1	4%	4%	5%	3%
PFKFB1	4%	5%	4%	3%
PIK3C2G	4%	4%	4%	4%
PLCB4	4%	4%	4%	4%
PPEF1	4%	4%	4%	4%
PPM1H	4%	5%	3%	3%
PRRC2C	4%	3%	4%	5%
PSG7	4%	3%	4%	6%
PXDNL	4%	2%	8%	4%
RNF17	4%	4%	3%	4%
SLC22A10	4%	5%	4%	3%
SLC2A5	4%	4%	5%	3%
SLC35G1	4%	5%	2%	4%
ST18	4%	5%	4%	3%
SYMPK	4%	3%	4%	5%
TANC2	4%	3%	5%	5%
TDRD10	4%	3%	5%	4%
THSD7A	4%	3%	4%	5%
TMEM38B	4%	4%	3%	5%
TPCN2	4%	3%	4%	5%
TSC2	4%	5%	4%	3%
TSG101	4%	4%	3%	4%
TTC21A	4%	4%	4%	4%
TTC39A	4%	4%	3%	4%
WDR96	4%	3%	4%	6%
XPNPEP2	4%	3%	5%	5%
XPR1	4%	4%	4%	4%
ABCC9	4%	3%	4%	5%
ADAMTS12	4%	3%	4%	5%
AHNAK	4%	3%	5%	4%
ALDH1L1	4%	3%	6%	4%
APOB	4%	3%	6%	4%
ATP2C1	4%	3%	2%	6%
BPIFB4	4%	4%	3%	5%
C1orf101	4%	3%	5%	5%
C9	4%	4%	5%	3%
CAPN9	4%	3%	6%	4%
CATSPERB	4%	3%	4%	5%
CCT6P3	4%	3%	4%	5%
CELF2	4%	3%	5%	4%
CEP350	4%	3%	4%	5%

CHEK2P2	4%	3%	3%	5%
CNTRL	4%	3%	3%	5%
CRMP1	4%	3%	4%	4%
DAAM2	4%	4%	3%	4%
DAB1	4%	3%	5%	5%
DMXL1	4%	3%	5%	4%
DOCK10	4%	4%	4%	4%
DROSHA	4%	3%	4%	5%
DTX2P1-UPK3BP1-PMS2P11	4%	3%	5%	4%
DZANK1	4%	3%	5%	5%
FCRL2	4%	2%	5%	6%
FREM2	4%	3%	4%	5%
FUBP3	4%	5%	4%	3%
GADL1	4%	4%	4%	3%
GNPAT	4%	3%	4%	6%
GPR116	4%	4%	5%	4%
GRIPAP1	4%	3%	4%	5%
GTF2A1L	4%	3%	5%	5%
GUCY2C	4%	4%	3%	4%
HSD17B4	4%	3%	4%	6%
HSPD1	4%	5%	3%	3%
IFT172	4%	3%	3%	5%
INO80	4%	5%	3%	3%
INPP5D	4%	3%	6%	3%
ITGAX	4%	4%	5%	3%
ITGB1	4%	4%	5%	3%
KANSL1L	4%	4%	5%	4%
KCNMA1	4%	3%	6%	4%
KCNQ3	4%	3%	5%	4%
LAMB4	4%	4%	3%	4%
LCT	4%	3%	6%	4%
LIG1	4%	3%	5%	5%
LMAN1L	4%	4%	4%	4%
MASP1	4%	4%	3%	5%
MTOR	4%	4%	3%	4%
MYOM3	4%	3%	5%	5%
NINL	4%	4%	3%	5%
NLK	4%	4%	3%	3%
NPEPPS	4%	3%	4%	5%
NSMAF	4%	3%	4%	5%
NSRP1	4%	5%	2%	4%
NUP205	4%	3%	4%	5%
PAM	4%	3%	4%	5%
PCDH11X	4%	4%	7%	2%
PLCH1	4%	3%	3%	5%
POTEC	4%	3%	5%	4%
PPP3CC	4%	4%	4%	4%
PRIM2	4%	4%	6%	2%
PRKCG	4%	3%	4%	5%
PSMA5	4%	4%	3%	3%
PTEN	4%	3%	5%	4%
PTPRF	4%	2%	5%	6%
PZP	4%	4%	5%	4%
RIMBP2	4%	4%	5%	3%
RRN3P1	4%	5%	3%	3%
SCN8A	4%	4%	3%	5%
SCN9A	4%	3%	4%	4%
SCYL2	4%	4%	4%	4%
SLC9C1	4%	4%	4%	3%
SMARCC1	4%	4%	3%	4%
SMC6	4%	3%	6%	4%
SORBS2	4%	3%	3%	5%

SPAG17	4%	2%	6%	5%
SPTBN1	4%	3%	3%	5%
STRC	4%	3%	4%	5%
TBC1D19	4%	3%	3%	6%
TENM4	4%	3%	4%	5%
TMC5	4%	5%	1%	4%
TNIK	4%	3%	5%	5%
TRPM4	4%	3%	3%	6%
TSPAN8	4%	5%	4%	3%
TVP23C	4%	3%	5%	5%
USH1C	4%	3%	3%	6%
WDR70	4%	4%	4%	4%
ZFHX4	4%	3%	5%	4%
ZNF28	4%	3%	5%	5%
ZNF713	4%	4%	5%	3%
ADAM28	4%	4%	2%	4%
ADH7	4%	4%	3%	3%
ANKRD28	4%	4%	5%	3%
AOX1	4%	3%	4%	6%
AQP7P1	4%	4%	5%	3%
ARNT	4%	3%	5%	4%
ATAD3B	4%	4%	5%	3%
ATG7	4%	3%	5%	5%
ATG9A	4%	3%	6%	3%
ATP10B	4%	3%	3%	5%
AWAT1	4%	3%	4%	4%
CAPN13	4%	3%	6%	4%
CCDC155	4%	4%	4%	3%
CENPN	4%	4%	3%	4%
CHD8	4%	3%	4%	5%
CLIP1	4%	4%	3%	4%
CNTN6	4%	4%	5%	3%
DIP2C	4%	4%	4%	3%
DRG1	4%	5%	2%	3%
FAT1	4%	4%	3%	3%
HDX	4%	4%	4%	3%
ITGA7	4%	4%	3%	4%
KCNH1	4%	2%	5%	5%
KDM3A	4%	3%	5%	5%
KIAA0196	4%	4%	2%	4%
KIAA0319	4%	4%	3%	3%
LMO7	4%	3%	4%	5%
LRRC69	4%	5%	3%	1%
MATN2	4%	2%	4%	6%
MCTP2	4%	4%	5%	3%
MED24	4%	3%	2%	6%
MYH8	4%	3%	2%	5%
NAA60	4%	2%	5%	5%
NAV1	4%	4%	3%	4%
NAV3	4%	3%	5%	4%
NBEAP1	4%	3%	5%	4%
NEDD4L	4%	4%	3%	4%
NEO1	4%	3%	2%	6%
NKAIN3	4%	3%	5%	4%
NUP214	4%	3%	3%	6%
NUP85	4%	3%	4%	5%
OC90	4%	4%	3%	3%
OR4F15	4%	4%	4%	4%
PASD1	4%	2%	6%	5%
PIKFYVE	4%	3%	4%	5%
PRKCQ	4%	4%	3%	4%
PTPRG	4%	4%	4%	3%

RANBP17	4%	3%	3%	5%
REPS2	4%	4%	5%	3%
RFX4	4%	4%	2%	5%
SCN2A	4%	3%	5%	5%
SH3KBP1	4%	4%	3%	3%
SLC12A8	4%	3%	4%	4%
SLC26A9	4%	3%	5%	4%
SLCO1B1	4%	3%	4%	4%
SMC1B	4%	3%	4%	5%
SORCS2	4%	4%	3%	4%
SORL1	4%	3%	5%	4%
STAT4	4%	3%	7%	4%
SULF2	4%	3%	5%	4%
TAOK1	4%	3%	4%	4%
TDRD9	4%	4%	4%	4%
TEK	4%	2%	5%	5%
THADA	4%	3%	4%	4%
TM6SF2	4%	3%	5%	3%
TMEM106B	4%	4%	3%	4%
TMEM8B	4%	4%	4%	2%
TRDN	4%	3%	3%	5%
TVP23C-CDRT4	4%	3%	4%	5%
VAV3	4%	4%	4%	4%
VWA3B	4%	4%	3%	4%
WWP1	4%	4%	3%	4%
ZCCHC10	4%	5%	1%	3%
ZNFX1	4%	4%	3%	4%
ACSM2B	4%	4%	3%	4%
ADAM23	4%	4%	4%	2%
APBB2	4%	4%	4%	3%
ATP13A5	4%	2%	7%	4%
BBS9	4%	3%	3%	5%
BPI	4%	3%	3%	5%
C1orf56	4%	4%	3%	4%
C5	4%	3%	3%	4%
CACNA2D4	4%	2%	8%	4%
COL28A1	4%	3%	4%	5%
COL4A4	4%	4%	4%	3%
CYP2A7	4%	3%	4%	5%
DDX60L	4%	3%	4%	5%
DPY19L1	4%	3%	5%	4%
DYNC1I1	4%	2%	7%	4%
ECM1	4%	2%	6%	5%
ELMO1	4%	2%	5%	5%
EML1	4%	4%	3%	3%
ESPNP	4%	4%	4%	3%
GBAP1	4%	3%	4%	5%
GPA33	4%	3%	4%	5%
GUCY2F	4%	4%	2%	5%
HEPH	4%	3%	3%	5%
HERC3	4%	3%	5%	5%
HOOK3	4%	4%	4%	3%
KIF4A	4%	4%	4%	3%
KRT6C	4%	3%	2%	6%
LARS	4%	3%	4%	4%
LRRC37A3	4%	3%	3%	5%
MADD	4%	3%	4%	4%
MAGEC3	4%	3%	4%	5%
MAGI2	4%	3%	3%	4%
MAP2K4	4%	5%	3%	3%
MARK4	4%	4%	3%	4%
MBTPS2	4%	4%	3%	4%

MCM3AP	4%	4%	4%	3%
MYH10	4%	4%	4%	3%
NLRC5	4%	2%	7%	4%
NUP155	4%	3%	3%	4%
NXPE2	4%	5%	4%	2%
OSBPL3	4%	4%	3%	4%
P2RX7	4%	4%	4%	3%
P4HA3	4%	2%	6%	4%
PHEX	4%	3%	5%	5%
PIP5K1A	4%	3%	4%	4%
PRG4	4%	3%	4%	4%
PRKAR2A	4%	3%	7%	2%
PSMA2	4%	5%	3%	2%
PTK2	4%	3%	4%	4%
RASGRF1	4%	3%	5%	4%
RPS6KB1	4%	3%	4%	5%
SETBP1	4%	3%	3%	5%
SLC24A4	4%	2%	5%	5%
SLC26A7	4%	3%	3%	4%
SPRN	4%	3%	4%	4%
SRGAP1	4%	3%	4%	5%
STAMBPL1	4%	3%	5%	4%
STARD9	4%	4%	4%	4%
TMEM67	4%	4%	4%	3%
TRPM1	4%	3%	3%	5%
TRPM8	4%	4%	5%	3%
TXLNG	4%	4%	1%	5%
USP42	4%	3%	4%	5%
WDR43	4%	3%	5%	4%
WRN	4%	3%	4%	5%
YEATS2	4%	3%	5%	3%
ZFPM2	4%	3%	5%	4%
ZFYVE26	4%	4%	1%	4%
ZIM2	4%	2%	4%	6%
ZKSCAN7	4%	3%	4%	4%
ZNF285	4%	4%	3%	3%
ABCA1	4%	3%	4%	4%
ABCA10	4%	3%	3%	5%
ABCB5	4%	2%	6%	4%
ABLIM3	4%	4%	3%	3%
ACPP	4%	4%	3%	3%
ANLN	4%	4%	2%	4%
ARHGEF10	4%	2%	3%	7%
ATP11A	4%	3%	4%	4%
ATP6V1C1	4%	4%	4%	3%
ATP9A	4%	2%	3%	6%
AWAT2	4%	4%	3%	3%
BRD9	4%	2%	6%	4%
BRWD1	4%	3%	3%	5%
BZRAP1-AS1	4%	2%	4%	6%
CA5A	4%	4%	1%	4%
CALN1	4%	4%	1%	5%
CHD2	4%	3%	5%	3%
CHD7	4%	2%	6%	4%
CLSTN3	4%	3%	3%	5%
COL5A2	4%	3%	5%	3%
COX10	4%	3%	3%	4%
CUL9	4%	2%	5%	4%
CWC22	4%	3%	4%	4%
DHRS2	4%	4%	4%	3%
DNMT1	4%	2%	6%	5%
DZIP1L	4%	3%	5%	4%

EHMT1	4%	3%	4%	4%
EP300	4%	3%	4%	4%
EPB42	4%	4%	2%	3%
FAM13A	4%	4%	1%	4%
FAM86C2P	4%	3%	4%	4%
FSTL1	4%	4%	4%	3%
G6PC	4%	3%	4%	5%
GLB1L3	4%	2%	4%	6%
GRHL3	4%	3%	3%	4%
HCAR1	4%	3%	5%	3%
HECTD1	4%	4%	4%	3%
HELZ	4%	3%	4%	4%
HSPE1-MOB4	4%	5%	2%	3%
INMT-FAM188B	4%	3%	3%	4%
ITCH	4%	3%	5%	4%
KIAA1549L	4%	2%	3%	6%
KLF7	4%	4%	3%	4%
KTN1	4%	3%	3%	4%
L3MBTL3	4%	4%	3%	4%
LAMC1	4%	3%	5%	4%
LGR6	4%	3%	5%	3%
LIAS	4%	3%	5%	3%
LRRC37B	4%	3%	3%	5%
MAPKAPK3	4%	4%	4%	3%
MCM7	4%	3%	5%	4%
MLLT3	4%	3%	4%	3%
MRPL3	4%	4%	4%	3%
NAA25	4%	4%	2%	3%
NBEAL1	4%	3%	3%	5%
NF2	4%	4%	3%	3%
NPHS1	4%	2%	5%	5%
NRXN1	4%	4%	3%	3%
P2RY12	4%	2%	4%	6%
PALLD	4%	4%	2%	4%
POLN	4%	3%	5%	3%
POTEKP	4%	3%	6%	3%
PTPRE	4%	3%	3%	4%
PTPRS	4%	3%	2%	5%
RAPGEF6	4%	3%	3%	5%
RB1CC1	4%	3%	4%	4%
RFX7	4%	5%	3%	2%
RGS22	4%	2%	5%	5%
RNF212	4%	4%	3%	3%
SCN3A	4%	2%	6%	4%
SGIP1	4%	3%	5%	4%
SLC17A8	4%	4%	4%	2%
SLC2A3	4%	3%	5%	4%
SLC4A7	4%	3%	4%	5%
SMYD3	4%	4%	2%	4%
SPAG8	4%	4%	4%	3%
TBC1D31	4%	4%	3%	4%
TBC1D3P2	4%	3%	3%	5%
TMEM254	4%	3%	3%	4%
TMEM87B	4%	4%	3%	4%
TNPO2	4%	3%	8%	3%
TOMM70A	4%	4%	2%	4%
TRIM51	4%	3%	5%	3%
TRPM2	4%	3%	4%	4%
TRPM3	4%	3%	3%	5%
TSEN2	4%	4%	3%	3%
TYW1	4%	3%	4%	4%
USP7	4%	3%	2%	6%

WHSC1L1	4%	3%	4%	4%
XDH	4%	3%	4%	4%
YWHAE	4%	4%	6%	1%
ZCWPW1	4%	4%	4%	3%
ZFC3H1	4%	4%	3%	2%
ZMYM4	4%	3%	5%	4%
ZNF432	4%	3%	5%	4%
ZNF512B	4%	4%	4%	3%
ZNF577	4%	3%	5%	3%
ZNF814	4%	3%	4%	4%
ABCB6	3%	3%	5%	3%
ABR	3%	3%	4%	4%
ADCY8	3%	4%	4%	3%
ADPGK	3%	3%	4%	4%
ALG1L2	3%	3%	6%	3%
ANKAR	3%	3%	5%	3%
ANKHD1	3%	4%	3%	3%
AOAH	3%	3%	5%	3%
AP4B1	3%	3%	4%	4%
ARPP21	3%	3%	3%	4%
ATP5J2-PTCD1	3%	4%	4%	2%
ATP6V1D	3%	4%	2%	3%
BBS5	3%	3%	3%	4%
BET1L	3%	2%	4%	5%
CAD	3%	3%	4%	4%
CADPS2	3%	3%	4%	5%
CALR3	3%	4%	2%	3%
CAMK2A	3%	3%	4%	3%
CATSPERG	3%	3%	4%	3%
CDH18	3%	2%	6%	4%
CEP170P1	3%	3%	4%	4%
CGA	3%	3%	4%	4%
COL16A1	3%	2%	3%	5%
COL9A1	3%	3%	4%	4%
CPAMD8	3%	3%	2%	4%
CTNND1	3%	4%	4%	2%
DHX9	3%	3%	4%	4%
DRGX	3%	4%	4%	3%
EIF3I	3%	1%	6%	5%
EMR1	3%	3%	4%	4%
ERBB4	3%	2%	4%	5%
FAM91A1	3%	4%	3%	3%
FER1L6-AS1	3%	5%	3%	1%
GRIN2A	3%	3%	4%	3%
HGSNAT	3%	4%	5%	2%
IL1RL2	3%	4%	4%	3%
IPMK	3%	4%	4%	3%
ITGA2	3%	4%	1%	4%
KLHL2	3%	3%	2%	5%
MACROD2	3%	2%	4%	5%
METTL2B	3%	4%	2%	4%
MPDZ	3%	3%	5%	3%
MUC12	3%	2%	5%	4%
MYH2	3%	4%	4%	3%
MYO15A	3%	3%	5%	4%
NLRP3	3%	3%	4%	4%
OTOF	3%	3%	5%	4%
PARP11	3%	3%	7%	1%
PIGB	3%	4%	3%	3%
PLEKHA3	3%	3%	4%	4%
PM20D1	3%	3%	3%	5%
PMS2P5	3%	3%	3%	5%

PNLDC1	3%	3%	2%	6%
PPL	3%	3%	5%	3%
PPOX	3%	2%	4%	5%
PRRC2B	3%	3%	3%	4%
PTCD1	3%	4%	4%	2%
PTPRJ	3%	3%	4%	3%
RAD51B	3%	3%	2%	4%
RBM6	3%	4%	4%	3%
RFC1	3%	3%	3%	5%
RMDN2-AS1	3%	3%	2%	5%
SIGLEC10	3%	4%	2%	3%
SIRPB1	3%	4%	3%	4%
SMARCA1	3%	3%	5%	4%
SNX29P1	3%	3%	3%	4%
SORBS1	3%	3%	3%	5%
SSBP3	3%	2%	5%	4%
STAG1	3%	2%	5%	4%
STXBP1	3%	3%	5%	3%
STXBP3	3%	4%	5%	2%
STYK1	3%	4%	3%	2%
TBC1D23	3%	4%	4%	3%
TMEM180	3%	4%	2%	4%
TMEM234	3%	1%	6%	5%
TRAK2	3%	3%	4%	4%
TRIM14	3%	3%	2%	5%
TXNL4B	3%	3%	4%	4%
VDAC3	3%	4%	3%	3%
WDPCP	3%	4%	3%	3%
WDR17	3%	3%	3%	4%
WDR52-AS1	3%	3%	4%	4%
XKR4	3%	2%	7%	4%
ZNF540	3%	4%	2%	3%
ZNF83	3%	4%	3%	3%
ADA	3%	4%	2%	4%
ADAMTS9	3%	3%	3%	4%
AIF1L	3%	3%	4%	3%
ANKRD17	3%	3%	3%	5%
AP4M1	3%	3%	4%	3%
ARHGEF12	3%	3%	2%	5%
ARHGEF28	3%	3%	3%	4%
ARMC4	3%	2%	5%	4%
ATG2B	3%	4%	3%	3%
ATP2B2	3%	3%	3%	4%
ATP2B4	3%	4%	4%	3%
BAZ1A	3%	2%	5%	5%
C11orf65	3%	3%	4%	4%
C16orf62	3%	2%	5%	4%
C3orf83	3%	4%	3%	3%
CAPS2	3%	4%	2%	4%
CD53	3%	4%	2%	3%
CFTR	3%	2%	6%	4%
CGB1	3%	3%	3%	4%
CLCN1	3%	3%	4%	4%
CLCNKA	3%	2%	4%	5%
CLEC18B	3%	2%	9%	2%
CMYA5	3%	4%	2%	4%
COL17A1	3%	3%	4%	4%
COL21A1	3%	3%	6%	3%
CRLS1	3%	5%	2%	2%
DOPEY1	3%	3%	4%	3%
EI24	3%	4%	4%	1%
ELAVL4	3%	5%	2%	2%

EPT1	3%	2%	5%	5%
ERO1LB	3%	3%	5%	4%
FAN1	3%	4%	2%	3%
FAT2	3%	3%	5%	3%
FLNC	3%	2%	5%	5%
GAREM	3%	3%	4%	3%
GCN1L1	3%	3%	4%	3%
GCOM1	3%	3%	1%	5%
GLE1	3%	3%	3%	4%
GLG1	3%	4%	4%	2%
GPR133	3%	3%	3%	4%
GREB1L	3%	4%	3%	3%
GRIK2	3%	3%	2%	4%
HECW2	3%	3%	3%	4%
HSF2	3%	3%	3%	4%
IFI16	3%	3%	5%	4%
IFT140	3%	4%	3%	3%
IKBKAP	3%	3%	4%	3%
INPP4A	3%	2%	5%	4%
KIAA0586	3%	3%	3%	4%
KIF14	3%	3%	4%	3%
KIF5B	3%	3%	5%	4%
KPNB1	3%	4%	3%	3%
MAP4K4	3%	3%	3%	5%
MARCO	3%	3%	4%	4%
MAST4	3%	3%	4%	4%
MEI1	3%	3%	3%	4%
NCL	3%	3%	3%	4%
NLRP5	3%	3%	4%	3%
NRCAM	3%	3%	3%	4%
NRXN2	3%	3%	4%	3%
PARP4	3%	3%	3%	4%
PDE8A	3%	3%	4%	3%
POLR2B	3%	2%	3%	6%
POLR2M	3%	3%	1%	5%
PRMT8	3%	3%	4%	3%
PTPRT	3%	3%	3%	4%
RB1	3%	3%	4%	4%
ROBO2	3%	3%	6%	3%
SAMSN1	3%	3%	7%	2%
SCEL	3%	2%	5%	4%
SENP2	3%	3%	3%	4%
SEPT10	3%	3%	3%	4%
SERPINB11	3%	3%	2%	5%
SIGLEC6	3%	3%	3%	4%
SLC26A4	3%	3%	5%	3%
SLC37A3	3%	3%	4%	4%
SLC9A9	3%	2%	3%	6%
SLK	3%	3%	4%	3%
SNORA63	3%	3%	2%	4%
SPG11	3%	3%	4%	3%
SSPO	3%	2%	6%	4%
SUCO	3%	2%	3%	6%
SVILP1	3%	3%	5%	3%
TACC1	3%	4%	4%	3%
TARS2	3%	3%	5%	4%
TBCK	3%	2%	3%	5%
TGFBI	3%	4%	1%	4%
TMEM131	3%	3%	4%	4%
TNKS1BP1	3%	2%	6%	3%
TNN	3%	3%	3%	4%
TOP2A	3%	2%	4%	5%

TP53BP2	3%	2%	3%	6%
TRAPPC10	3%	3%	4%	4%
UBN1	3%	3%	3%	4%
UGGT2	3%	3%	3%	5%
USP13	3%	3%	2%	5%
UST	3%	4%	3%	3%
VWDE	3%	4%	3%	3%
WDR41	3%	4%	4%	2%
WDR44	3%	3%	2%	5%
WWP2	3%	2%	7%	3%
ZDHHC9	3%	4%	2%	3%
ABCC4	3%	2%	5%	4%
ABCC5	3%	3%	4%	3%
ACOT11	3%	3%	5%	2%
ACTR3C	3%	4%	2%	3%
ADAMTS16	3%	3%	3%	4%
ADCY5	3%	3%	5%	3%
ALG1	3%	3%	3%	4%
ALG13	3%	2%	4%	5%
ANXA13	3%	3%	5%	3%
ARHGEF33	3%	2%	4%	4%
ASCC3	3%	3%	4%	3%
ATP1A2	3%	3%	4%	4%
C1orf168	3%	4%	2%	3%
CACNA1H	3%	3%	4%	3%
CATSPERD	3%	4%	2%	3%
CCZ1B	3%	3%	2%	5%
CDH17	3%	3%	4%	4%
CEP89	3%	3%	2%	4%
CHD3	3%	3%	4%	3%
CLN3	3%	2%	5%	4%
CTAGE5	3%	2%	5%	4%
CUL3	3%	3%	4%	4%
CYFIP2	3%	3%	2%	4%
DENND4B	3%	2%	6%	4%
DHX57	3%	3%	5%	3%
DMXL2	3%	3%	5%	3%
DOPEY2	3%	3%	4%	3%
EGFLAM	3%	3%	3%	4%
ERMP1	3%	3%	3%	4%
EVA1C	3%	3%	4%	3%
EXOSC3	3%	3%	4%	4%
FAM153B	3%	3%	3%	3%
FNDC3B	3%	2%	4%	4%
FOLH1B	3%	2%	4%	4%
FRMPD4	3%	3%	2%	4%
GOLGA2	3%	3%	3%	4%
GOLGA8A	3%	2%	4%	4%
GSDMC	3%	3%	4%	3%
HAVCR1	3%	3%	3%	5%
HEATR5A	3%	2%	2%	6%
HSPE1	3%	4%	2%	2%
IL18R1	3%	3%	3%	5%
INTS8	3%	3%	3%	4%
ITGA8	3%	3%	5%	3%
ITGA9	3%	2%	5%	4%
ITSN1	3%	3%	2%	4%
KDR	3%	3%	4%	4%
LAMA5	3%	3%	3%	3%
LIPA	3%	3%	3%	4%
LRRC16B	3%	4%	3%	3%
LRRIQ1	3%	3%	3%	4%

LTBP1	3%	2%	4%	4%
MAP3K4	3%	3%	2%	5%
MED23	3%	2%	2%	6%
MEP1A	3%	3%	4%	3%
MTBP	3%	3%	4%	3%
MYH14	3%	1%	5%	5%
MYO9A	3%	3%	3%	4%
NDUFB9	3%	4%	2%	3%
NOS1	3%	2%	5%	5%
NRG2	3%	3%	3%	3%
OSBPL9	3%	3%	2%	4%
PDE4D	3%	3%	2%	4%
PHF20L1	3%	3%	4%	2%
PREX1	3%	2%	5%	4%
PTK7	3%	3%	4%	3%
PYGB	3%	3%	4%	4%
RAB31	3%	4%	3%	3%
RAD18	3%	4%	2%	2%
RALGPS1	3%	2%	4%	4%
RBBP4	3%	4%	3%	3%
RGS9	3%	3%	2%	4%
RMDN2	3%	3%	2%	5%
RSF1	3%	3%	4%	4%
SCN11A	3%	2%	5%	4%
SEMA3D	3%	4%	3%	3%
SENP7	3%	3%	6%	2%
SF3B1	3%	4%	3%	2%
SKIV2L2	3%	4%	2%	3%
SLC12A2	3%	2%	3%	5%
SLC4A2	3%	3%	4%	4%
SND1	3%	3%	4%	3%
SORCS3	3%	3%	2%	5%
SRRM2	3%	3%	4%	3%
SYN3	3%	3%	5%	3%
TACC2	3%	3%	4%	3%
TANC1	3%	2%	5%	4%
TBC1D8	3%	3%	3%	4%
TCF4	3%	2%	4%	5%
THOC1	3%	3%	5%	3%
TNR	3%	3%	2%	5%
TRIP12	3%	2%	5%	4%
TTF2	3%	3%	6%	1%
TUFT1	3%	3%	4%	3%
UBE3C	3%	2%	4%	4%
UMODL1	3%	2%	5%	4%
VAV1	3%	4%	2%	3%
VAV2	3%	4%	2%	3%
ZBTB41	3%	3%	4%	4%
ZNF638	3%	3%	3%	4%
ZNF670	3%	3%	3%	4%
ZNF813	3%	2%	4%	4%
ACAD11	3%	3%	4%	4%
ACSL6	3%	3%	4%	3%
ADAM15	3%	2%	6%	4%
ADAM17	3%	3%	3%	4%
ADCK3	3%	3%	4%	2%
ADGB	3%	3%	6%	2%
AGBL5	3%	3%	3%	4%
AMOT	3%	4%	2%	3%
ANKRD44	3%	2%	3%	4%
ANPEP	3%	3%	2%	5%
APPL2	3%	4%	2%	3%

ARAP1	3%	3%	4%	3%
ARHGEF39	3%	2%	3%	5%
ARHGEF7	3%	3%	4%	3%
ASTN2	3%	2%	3%	5%
ATP11C	3%	3%	4%	4%
ATP2A1	3%	3%	2%	4%
ATP8B4	3%	3%	3%	3%
ATRN	3%	2%	3%	4%
ATXN2	3%	3%	3%	3%
BLM	3%	3%	2%	4%
C10orf76	3%	3%	3%	3%
C15orf38-AP3S2	3%	3%	4%	2%
CACHD1	3%	3%	3%	4%
CD86	3%	3%	3%	3%
CDKL4	3%	3%	3%	4%
CHD9	3%	3%	5%	3%
CHRNB1	3%	3%	4%	2%
CIRBP	3%	2%	3%	5%
CIRBP-AS1	3%	2%	3%	5%
CLDN11	3%	3%	4%	3%
CLSTN2	3%	2%	5%	4%
CNKSR2	3%	3%	2%	4%
CNTNAP3	3%	2%	4%	4%
CSNK2A2	3%	3%	5%	2%
CTNNA3	3%	3%	5%	3%
CXorf65	3%	3%	3%	4%
CYP11B1	3%	4%	2%	3%
CYP4A22	3%	3%	3%	3%
DIP2A	3%	3%	3%	4%
EEA1	3%	3%	3%	3%
EGF	3%	4%	3%	2%
EIF4G3	3%	3%	2%	4%
EML6	3%	2%	4%	4%
EMR3	3%	3%	3%	4%
ENOSF1	3%	2%	4%	4%
ENPP3	3%	2%	4%	4%
ENTPD1-AS1	3%	4%	2%	3%
EPB41L2	3%	3%	4%	2%
EYA1	3%	2%	4%	4%
FADS2	3%	3%	3%	3%
FAM188B	3%	3%	1%	4%
FAM208A	3%	4%	3%	3%
FAM86C1	3%	3%	4%	3%
FNTB	3%	3%	3%	3%
GK	3%	3%	5%	3%
GRIA1	3%	3%	3%	4%
HDAC3	3%	3%	3%	4%
HDAC4	3%	3%	3%	4%
HIVEP1	3%	2%	4%	4%
IKBKB	3%	3%	3%	4%
IL12RB1	3%	4%	2%	2%
IL7	3%	4%	3%	2%
IQCH	3%	4%	2%	3%
ITIH2	3%	1%	6%	4%
ITPA	3%	3%	3%	3%
KCNMB3	3%	3%	3%	4%
KIAA2022	3%	2%	4%	4%
KIRREL	3%	3%	2%	4%
KLRC2	3%	3%	4%	2%
LRIG1	3%	2%	3%	5%
LRRC59	3%	3%	3%	3%
MAP3K5	3%	2%	5%	3%

OPRM1	3%	3%	3%	3%
OSBPL10	3%	3%	2%	4%
PAICS	3%	3%	4%	3%
PDE10A	3%	3%	3%	4%
PGM1	3%	2%	4%	4%
PIP4K2A	3%	3%	3%	3%
PKIG	3%	4%	2%	3%
PLCG2	3%	3%	5%	3%
PPFIA2	3%	3%	3%	4%
PPP6R3	3%	3%	3%	4%
PRDM7	3%	2%	5%	3%
PRPF40B	3%	3%	3%	3%
PSG2	3%	3%	3%	3%
PTPRH	3%	3%	3%	4%
PYGL	3%	3%	3%	3%
RAB27A	3%	4%	3%	2%
RBAK-RBAKDN	3%	2%	3%	5%
RBM18	3%	3%	3%	3%
RBMX	3%	3%	6%	2%
RGS6	3%	3%	4%	3%
RPRD1B	3%	3%	3%	4%
SDHD	3%	3%	3%	4%
SIDT1	3%	3%	3%	3%
SLC2A9	3%	3%	2%	5%
SNTG1	3%	2%	5%	3%
SPATA13	3%	2%	3%	5%
SPRR2B	3%	4%	2%	3%
SPTLC1	3%	3%	2%	4%
SYTL5	3%	4%	2%	3%
TAF4B	3%	4%	3%	3%
TBC1D32	3%	3%	2%	4%
TFIP11	3%	2%	3%	5%
TLE1	3%	4%	3%	3%
TMEM63A	3%	3%	3%	3%
TOP3A	3%	2%	4%	4%
TTC7B	3%	2%	2%	5%
UNC13B	3%	2%	2%	6%
VPS41	3%	3%	3%	3%
WBP11	3%	4%	2%	2%
WFDC10B	3%	4%	1%	3%
XRRA1	3%	3%	4%	3%
ZFP64	3%	3%	4%	3%
ZFP91-CNTF	3%	2%	5%	4%
ZMYM3	3%	3%	4%	3%
ZP4	3%	3%	3%	4%
ABCA5	3%	3%	3%	4%
ABCC2	3%	3%	3%	4%
ACAT1	3%	3%	3%	3%
ADAMTS17	3%	2%	4%	4%
ADAMTS6	3%	2%	3%	4%
ADCYAP1R1	3%	2%	3%	5%
ADH1C	3%	3%	3%	3%
ADSL	3%	3%	3%	4%
AGL	3%	3%	2%	4%
ALDH1A3	3%	3%	3%	3%
ALDH8A1	3%	3%	3%	4%
ANKRD11	3%	3%	4%	3%
AP3D1	3%	3%	2%	3%
ARFGAP3	3%	3%	3%	2%
ARID1A	3%	3%	1%	4%
ARPC4-TTLL3	3%	3%	1%	5%
ATAD5	3%	3%	2%	3%

ATP10D	3%	3%	2%	4%
ATP6V0A4	3%	2%	4%	3%
BCR	3%	2%	4%	4%
BRDT	3%	3%	3%	3%
BTBD16	3%	3%	3%	3%
C19orf24	3%	2%	3%	4%
C2orf61	3%	3%	4%	2%
C7	3%	2%	2%	6%
CCDC108	3%	2%	6%	2%
CCDC150	3%	2%	3%	4%
CD96	3%	2%	2%	5%
CDK11B	3%	3%	4%	3%
CEP250	3%	3%	2%	4%
CNTN1	3%	3%	4%	3%
COL23A1	3%	3%	3%	4%
COL9A3	3%	2%	4%	4%
CPVL	3%	2%	5%	3%
CSTF3	3%	3%	2%	4%
CYFIP1	3%	3%	3%	3%
CYP24A1	3%	3%	2%	3%
DDB1	3%	3%	4%	3%
DENND3	3%	2%	3%	5%
DOCK7	3%	3%	3%	4%
ERMAP	3%	3%	3%	3%
ETV6	3%	2%	4%	4%
EVC2	3%	2%	5%	3%
EXT2	3%	3%	2%	4%
FAM66B	3%	2%	3%	5%
FAT4	3%	1%	4%	5%
FBXO18	3%	3%	3%	4%
FCRL3	3%	2%	2%	5%
FTX	3%	3%	4%	3%
GK5	3%	3%	5%	2%
GOLGA3	3%	2%	4%	4%
GRHL2	3%	3%	3%	3%
GRIA2	3%	2%	3%	5%
GRID1	3%	3%	3%	3%
GZMA	3%	4%	2%	2%
HBP1	3%	3%	5%	2%
HERC2P3	3%	3%	1%	4%
HSD17B7P2	3%	4%	3%	2%
IL3RA	3%	3%	3%	3%
IPO5	3%	3%	2%	4%
IPO7	3%	4%	2%	3%
ITGAM	3%	1%	6%	4%
KAT2B	3%	3%	2%	4%
KIAA0556	3%	2%	3%	4%
KMT2E	3%	2%	4%	4%
KRT75	3%	3%	3%	4%
LHCGR	3%	3%	3%	4%
MAP2K5	3%	3%	3%	3%
MAP7D2	3%	3%	3%	3%
MC2R	3%	4%	2%	2%
MCM3	3%	4%	3%	2%
MICAL2	3%	1%	5%	4%
MMS22L	3%	2%	4%	3%
MTRR	3%	3%	2%	3%
MUC21	3%	4%	3%	2%
MXD1	3%	3%	4%	2%
MYO1H	3%	3%	4%	3%
NCKAP1L	3%	2%	4%	4%
NEDD4	3%	3%	2%	5%

NPL	3%	4%	3%	2%
NRL	3%	2%	3%	5%
NSUN2	3%	3%	3%	4%
NUMA1	3%	3%	2%	5%
NUP107	3%	2%	4%	4%
NXF3	3%	2%	3%	5%
OCRL	3%	2%	4%	5%
PCMTD1	3%	2%	4%	4%
PCNX	3%	3%	4%	3%
PDE3A	3%	3%	4%	3%
PEAR1	3%	2%	4%	4%
PIWIL3	3%	2%	2%	6%
PLCD4	3%	2%	4%	4%
PLEKHA5	3%	4%	3%	2%
PLGLA	3%	3%	3%	4%
PMS2CL	3%	3%	4%	3%
POGZ	3%	2%	4%	4%
POLB	3%	3%	2%	3%
POLR2A	3%	2%	4%	4%
POTEM	3%	3%	3%	3%
PRKAR1A	3%	3%	1%	4%
PTCD3	3%	3%	2%	4%
QRICH2	3%	2%	6%	3%
RASA2	3%	3%	4%	3%
REV1	3%	1%	2%	7%
RICTOR	3%	2%	4%	4%
ROCK1P1	3%	2%	2%	5%
RPGRIP1	3%	3%	4%	2%
RPL28	3%	3%	4%	3%
RPL9	3%	3%	4%	3%
SCLT1	3%	3%	4%	3%
SCN1A	3%	3%	3%	3%
SCUBE2	3%	2%	5%	3%
SDCCAG8	3%	2%	2%	5%
SELP	3%	4%	2%	3%
SETDB1	3%	1%	6%	4%
SHANK1	3%	3%	4%	3%
SIPA1L1	3%	2%	2%	5%
SLC4A1	3%	4%	3%	2%
SLC7A7	3%	2%	3%	5%
SMARCAL1	3%	2%	3%	5%
SMG6	3%	2%	3%	5%
SNORA11	3%	3%	2%	3%
SNRPF	3%	3%	5%	3%
SNTG2	3%	3%	3%	3%
SNX29	3%	2%	3%	5%
SP140L	3%	2%	4%	3%
SPAG9	3%	2%	1%	5%
SPATA18	3%	2%	5%	3%
STRN4	3%	3%	3%	3%
SUPT5H	3%	2%	3%	5%
SWAP70	3%	2%	4%	4%
SYN1	3%	3%	2%	3%
TAPBPL	3%	3%	4%	3%
TBC1D4	3%	3%	2%	3%
TET3	3%	2%	5%	3%
TMEM117	3%	3%	4%	2%
TMEM238	3%	3%	4%	3%
TMEM63C	3%	3%	5%	3%
TNPO1	3%	2%	1%	5%
TRAPPC8	3%	2%	1%	5%
TRHDE	3%	3%	3%	3%

TRMT1L	3%	4%	2%	2%
UBE2Q1	3%	2%	5%	3%
UPF2	3%	3%	4%	3%
VCAN	3%	2%	2%	5%
XPNPEP1	3%	3%	4%	2%
XXYLT1	3%	3%	4%	3%
ZCCHC11	3%	2%	3%	4%
ZNF235	3%	2%	5%	3%
ABCC8	3%	2%	4%	3%
ADAM9	3%	2%	4%	4%
ADAMTS3	3%	2%	4%	4%
AFF3	3%	4%	2%	3%
AGBL4	3%	2%	4%	4%
AKT1	3%	3%	3%	3%
ALDH1L2	3%	2%	1%	5%
ANKRD27	3%	3%	3%	4%
ANKS1B	3%	3%	3%	3%
AQR	3%	3%	2%	3%
ARHGAP12	3%	2%	4%	3%
ARHGAP21	3%	2%	5%	3%
ASMTL	3%	2%	3%	5%
ASNS	3%	3%	2%	3%
ATG16L1	3%	2%	2%	4%
ATP1A1	3%	3%	2%	3%
BTN3A3	3%	2%	3%	4%
C20orf194	3%	3%	2%	3%
C2CD5	3%	2%	2%	4%
C2orf15	3%	3%	4%	2%
CACNB1	3%	2%	2%	4%
CARD16	3%	2%	6%	3%
CAST	3%	3%	2%	3%
CCDC144NL	3%	3%	4%	3%
CCDC148	3%	2%	4%	3%
CDC73	3%	3%	4%	3%
CDH8	3%	3%	2%	3%
CDHR2	3%	3%	5%	2%
CDRT1	3%	3%	5%	2%
CGN	3%	2%	3%	4%
CHMP3	3%	2%	3%	4%
CHRNB4	3%	2%	4%	3%
CHURC1-FNTB	3%	3%	3%	3%
CIITA	3%	2%	4%	4%
CLASP1	3%	2%	4%	3%
CMTR1	3%	3%	4%	2%
CNGB1	3%	3%	3%	3%
COG6	3%	2%	4%	4%
COL25A1	3%	2%	3%	4%
COL2A1	3%	3%	3%	3%
COLGALT2	3%	3%	1%	4%
CR2	3%	2%	3%	5%
CSE1L	3%	2%	5%	3%
DAAM1	3%	3%	2%	4%
DAZL	3%	2%	4%	3%
DDX42	3%	2%	3%	5%
DEPDC1	3%	3%	2%	3%
DHX36	3%	4%	2%	2%
DLG1	3%	3%	2%	4%
DNA2	3%	3%	3%	3%
DPYS	3%	2%	3%	4%
DUOX2	3%	3%	3%	3%
EIF2AK2	3%	3%	3%	3%
EIF5B	3%	2%	3%	4%

ELP2	3%	2%	4%	4%
FAM3D	3%	3%	4%	2%
FAP	3%	3%	3%	4%
FCHSD1	3%	3%	2%	3%
FLT3	3%	2%	4%	3%
FNBP4	3%	2%	3%	4%
FRRS1	3%	3%	1%	4%
GABRQ	3%	4%	2%	3%
GALNTL6	3%	3%	4%	2%
GANAB	3%	2%	4%	3%
GBA2	3%	3%	3%	3%
GDPD4	3%	2%	5%	3%
GNPTAB	3%	2%	2%	5%
GOLGA4	3%	2%	4%	3%
GPLD1	3%	3%	5%	1%
GPR97	3%	3%	2%	4%
GSAP	3%	2%	3%	5%
HEATR1	3%	3%	4%	3%
HFM1	3%	3%	3%	3%
HRG	3%	3%	4%	3%
IGSF10	3%	2%	5%	3%
IL33	3%	4%	3%	1%
IL7R	3%	3%	3%	3%
ITGAV	3%	3%	4%	3%
ITSN2	3%	2%	3%	5%
KDM3B	3%	3%	2%	4%
KIAA0020	3%	2%	3%	4%
KIDINS220	3%	3%	3%	3%
KIF16B	3%	3%	1%	3%
KIF20B	3%	3%	4%	3%
KIF5A	3%	2%	3%	4%
KIF6	3%	3%	4%	2%
KSR1	3%	2%	3%	5%
LINC00221	3%	2%	3%	4%
MAP2K1	3%	3%	2%	3%
MAST1	3%	3%	2%	4%
MCM10	3%	2%	3%	4%
MED1	3%	1%	1%	7%
MLH3	3%	2%	3%	4%
MOV10L1	3%	3%	4%	2%
MTPAP	3%	2%	4%	4%
MUC6	3%	2%	4%	3%
MUTYH	3%	3%	2%	4%
MYO9B	3%	3%	3%	4%
MYZAP	3%	3%	1%	4%
NAALADL2	3%	3%	3%	3%
NCF2	3%	2%	4%	3%
NELL1	3%	2%	4%	4%
NEMF	3%	3%	2%	4%
NME8	3%	2%	5%	3%
NOL8	3%	3%	4%	2%
NUP160	3%	3%	4%	3%
PAH	3%	3%	3%	3%
PAX3	3%	3%	3%	4%
PCDHB16	3%	2%	5%	3%
PCDHGA12	3%	2%	3%	4%
PDE6C	3%	3%	1%	4%
PDGFRA	3%	2%	2%	4%
PDPR	3%	2%	4%	4%
PHACTR3	3%	4%	2%	3%
PIK3R1	3%	3%	2%	3%
PIK3R6	3%	2%	5%	2%

PIN4	3%	3%	3%	3%
PLA2G4A	3%	3%	3%	4%
PLCG1	3%	3%	2%	4%
PLEKHM1P	3%	3%	4%	3%
PMS2	3%	3%	2%	4%
POLR1C	3%	2%	5%	2%
PPP2R3A	3%	2%	4%	4%
PRKCB	3%	4%	3%	1%
PRKG2	3%	2%	4%	3%
PRSS42	3%	4%	3%	1%
PSG5	3%	3%	2%	4%
PSMC3IP	3%	4%	1%	3%
PTPN13	3%	3%	2%	4%
QTRTD1	3%	2%	4%	4%
RANBP3L	3%	2%	4%	4%
RFX2	3%	2%	5%	3%
RFX6	3%	2%	4%	4%
RNF111	3%	2%	5%	3%
RNF185	3%	3%	3%	3%
RPL5	3%	3%	3%	4%
RUFY2	3%	3%	3%	3%
RUNX1T1	3%	3%	2%	3%
SCN4A	3%	3%	3%	3%
SEH1L	3%	3%	4%	3%
SEL1L3	3%	2%	3%	4%
SELL	3%	2%	6%	2%
SENP5	3%	4%	2%	2%
SEPT6	3%	3%	2%	4%
SHPRH	3%	3%	4%	2%
SLC37A2	3%	3%	3%	3%
SLC4A8	3%	2%	4%	4%
SLC6A1-AS1	3%	3%	2%	4%
SPTAN1	3%	3%	2%	4%
ST3GAL6	3%	2%	2%	4%
STARD3	3%	1%	1%	7%
STRIP1	3%	3%	3%	4%
SUSD1	3%	3%	3%	3%
SUV420H1	3%	3%	2%	4%
TAF2	3%	2%	3%	4%
TBC1D9	3%	2%	3%	5%
TBP	3%	2%	4%	3%
TCP11L1	3%	3%	2%	4%
TEC	3%	3%	2%	4%
TMC2	3%	2%	3%	5%
TNFRSF10C	3%	3%	4%	1%
TPM3	3%	1%	4%	6%
TRIM6-TRIM34	3%	2%	2%	5%
TTC18	3%	3%	3%	3%
USHBP1	3%	2%	4%	3%
USO1	3%	2%	4%	3%
USP15	3%	3%	2%	3%
USP47	3%	3%	2%	4%
XPC	3%	3%	1%	4%
XPO1	3%	3%	4%	3%
XRCC5	3%	3%	2%	3%
ZBBX	3%	2%	3%	5%
ZNF208	3%	2%	5%	3%
ZNF701	3%	2%	3%	4%
AARS	3%	2%	5%	2%
ACOXL	3%	2%	5%	2%
ACSM2A	3%	3%	3%	3%
ADAM2	3%	3%	2%	3%

AGAP1	3%	2%	3%	3%
ALPK2	3%	2%	2%	4%
AMPD1	3%	2%	3%	4%
ANAPC4	3%	2%	4%	3%
ANKHD1-EIF4EBP3	3%	3%	3%	3%
ANO2	3%	2%	3%	4%
APOLD1	3%	3%	3%	3%
ARHGAP10	3%	2%	2%	5%
ARHGAP9	3%	3%	4%	3%
ARHGEF6	3%	3%	2%	4%
BIN2	3%	2%	5%	3%
BRD8	3%	3%	3%	2%
BTN3A2	3%	3%	2%	4%
BTNL3	3%	3%	2%	3%
CASQ1	3%	2%	3%	4%
CCAR1	3%	2%	4%	3%
CCBL2	3%	2%	4%	3%
CCDC170	3%	2%	4%	3%
CDH9	3%	3%	2%	3%
CENPI	3%	2%	3%	4%
CHEK2	3%	3%	3%	3%
CLASP2	3%	3%	3%	3%
CNDP2	3%	3%	2%	3%
CNTN2	3%	2%	4%	3%
CNTNAP5	3%	2%	2%	5%
CPNE5	3%	2%	4%	3%
CTAGE11P	3%	3%	4%	2%
CTNNBL1	3%	2%	3%	4%
CUL4B	3%	2%	2%	4%
CXorf22	3%	1%	3%	5%
CYP2B6	3%	2%	5%	3%
DDX52	3%	2%	4%	3%
DENND5B	3%	2%	2%	4%
DISC1	3%	1%	4%	5%
DPYSL5	3%	2%	4%	3%
EBF1	3%	1%	2%	5%
EDA	3%	2%	3%	4%
EDC4	3%	3%	3%	3%
EFHC1	3%	3%	3%	2%
EFTUD2	3%	3%	3%	3%
EIF4A3	3%	3%	2%	3%
EML4	3%	2%	3%	3%
EPHX1	3%	3%	3%	3%
EPHX2	3%	1%	4%	4%
ERAP2	3%	3%	3%	3%
ERC1	3%	4%	2%	2%
ESF1	3%	4%	3%	1%
FAM212B	3%	3%	2%	4%
FAM49B	3%	3%	4%	2%
FAM86JP	3%	3%	2%	4%
FBXW10	3%	2%	4%	3%
FCRL5	3%	2%	4%	4%
FMO1	3%	3%	4%	3%
FOXK2	3%	2%	2%	5%
FOXP1	3%	2%	1%	5%
FRMD4A	3%	2%	4%	3%
FTSJ3	3%	2%	5%	3%
GABBR2	3%	1%	3%	5%
GATS	3%	3%	5%	2%
GHITM	3%	3%	2%	3%
GHR	3%	3%	3%	3%
GIF	3%	3%	3%	1%

GNG12-AS1	3%	2%	3%	4%
GRAMD1C	3%	2%	3%	4%
GRID2	3%	2%	4%	3%
GRIN3A	3%	3%	2%	3%
GSDMB	3%	2%	2%	5%
HM13	3%	3%	3%	3%
HRC	3%	2%	4%	3%
INPP5B	3%	2%	3%	4%
INPPL1	3%	2%	4%	3%
IQSEC1	3%	3%	3%	3%
ITGA4	3%	2%	3%	4%
KATNAL2	3%	3%	2%	3%
KMO	3%	3%	1%	4%
KRT6A	3%	3%	2%	3%
LAMC2	3%	1%	6%	4%
LRMP	3%	3%	3%	2%
LRRC28	3%	3%	4%	2%
LTBP4	3%	2%	2%	5%
MANBA	3%	2%	4%	4%
MAP4K1	3%	2%	3%	4%
MARCH6	3%	2%	4%	3%
MCPH1	3%	2%	4%	3%
MICU3	3%	3%	1%	4%
MIR663B	3%	2%	5%	3%
MUSK	3%	2%	2%	4%
MYH7B	3%	2%	5%	3%
NCKAP5	3%	1%	6%	3%
NCOA2	3%	3%	1%	4%
NECAB1	3%	3%	3%	2%
NHLRC2	3%	2%	5%	2%
NOP14-AS1	3%	3%	3%	3%
NPHP4	3%	3%	3%	3%
NPLOC4	3%	2%	4%	3%
NRD1	3%	3%	3%	1%
NTN4	3%	2%	3%	4%
NUDT13	3%	3%	2%	3%
OTUD7A	3%	3%	3%	2%
PAPPA	3%	3%	1%	4%
PBRM1	3%	3%	3%	3%
PDCD6IP	3%	2%	4%	3%
PDE11A	3%	3%	2%	3%
PFKP	3%	2%	3%	4%
PHC2	3%	3%	2%	4%
PHC3	3%	3%	2%	4%
PLCE1	3%	2%	4%	3%
PLCZ1	3%	1%	4%	4%
POLA2	3%	2%	3%	4%
PSG1	3%	2%	2%	4%
PSG10P	3%	2%	2%	5%
PSG3	3%	2%	3%	4%
PSMD1	3%	2%	3%	4%
PTPN22	3%	2%	3%	3%
PTPRA	3%	2%	4%	3%
PTPRO	3%	3%	4%	2%
RAB37	3%	3%	3%	2%
RACGAP1	3%	3%	3%	2%
RALGAPA1	3%	2%	3%	4%
RASGRF2	3%	3%	2%	3%
RNPEP	3%	2%	2%	4%
ROR1	3%	3%	3%	3%
SCO1	3%	3%	4%	1%
SEMA4D	3%	2%	4%	4%

SETX	3%	2%	3%	4%
SH3D19	3%	2%	2%	5%
SLC10A7	3%	3%	2%	3%
SLC24A5	3%	3%	2%	3%
SLC25A12	3%	2%	4%	4%
SLC30A5	3%	2%	3%	4%
SLC39A11	3%	2%	3%	4%
SLC4A5	3%	2%	4%	3%
SLC5A9	3%	1%	5%	5%
SLC6A5	3%	3%	4%	3%
SLIT3	3%	2%	4%	4%
SMARCA5	3%	3%	2%	4%
SMC2	3%	3%	3%	3%
SMG5	3%	2%	3%	4%
SNX1	3%	3%	3%	3%
SNX25	3%	1%	3%	5%
SP110	3%	3%	2%	4%
SRBD1	3%	2%	3%	4%
STK32A	3%	1%	4%	5%
SUN1	3%	3%	3%	3%
SV2B	3%	3%	3%	3%
SYCP1	3%	3%	4%	2%
TCP11	3%	3%	4%	1%
TDG	3%	3%	4%	2%
TF	3%	2%	4%	4%
THSD4	3%	2%	4%	3%
TMEM258	3%	2%	5%	3%
TNFRSF10A	3%	2%	2%	5%
TP53BP1	3%	3%	2%	3%
USP25	3%	3%	4%	2%
USP39	3%	2%	3%	4%
WLS	3%	3%	3%	3%
XG	3%	2%	3%	4%
ZC3H11A	3%	2%	4%	4%
ZNF585A	3%	3%	2%	4%
ABCA6	3%	2%	4%	3%
ABCF3	3%	2%	4%	3%
ACSS2	3%	2%	2%	4%
AGBL2	3%	2%	3%	4%
AGO2	3%	2%	2%	4%
AKT3	3%	2%	3%	3%
ALDH18A1	3%	3%	2%	3%
ANKRD26	3%	3%	3%	3%
ANO5	3%	3%	3%	3%
ANP32A	3%	3%	3%	2%
ARAP3	3%	3%	2%	2%
ARHGAP30	3%	3%	3%	3%
ARHGEF2	3%	1%	4%	4%
ARMC3	3%	2%	4%	3%
ATP7B	3%	3%	4%	2%
BCAS3	3%	2%	2%	4%
BCRP3	3%	2%	2%	4%
BIVM-ERCC5	3%	3%	4%	1%
BMPER	3%	1%	3%	5%
BTN2A1	3%	2%	3%	3%
C14orf178	3%	2%	4%	3%
C19orf44	3%	3%	3%	3%
C5orf58	3%	3%	2%	2%
C9orf3	3%	2%	2%	4%
CAMK2G	3%	3%	4%	2%
CAPN3	3%	2%	2%	4%
CAPRIN2	3%	3%	2%	3%

CARS2	3%	2%	6%	2%
CCDC158	3%	2%	5%	2%
CCDC168	3%	2%	2%	4%
CCDC30	3%	3%	3%	3%
CCDC88A	3%	1%	5%	3%
CD72	3%	3%	4%	2%
CDC42BPB	3%	2%	4%	3%
CELSR1	3%	2%	5%	3%
CHKA	3%	2%	4%	3%
CLCNKB	3%	2%	3%	3%
CLOCK	3%	3%	2%	3%
CNOT10	3%	3%	3%	3%
COLQ	3%	2%	4%	4%
COPG1	3%	2%	4%	2%
CPSF3	3%	2%	3%	3%
CRAMP1L	3%	2%	3%	3%
CS	3%	2%	3%	4%
CTCFL	3%	2%	4%	3%
CTPS2	3%	3%	2%	3%
CTTN	3%	2%	3%	3%
CYP2E1	3%	3%	2%	3%
CYP4Z2P	3%	3%	2%	4%
DAB2	3%	3%	4%	2%
DCAF11	3%	2%	2%	4%
DDR2	3%	3%	3%	3%
DIDO1	3%	3%	3%	2%
DLC1	3%	2%	3%	4%
DLD	3%	3%	3%	3%
DPYD	3%	3%	2%	3%
DSP	3%	2%	2%	5%
DUS2	3%	3%	2%	3%
DYNC1LI2	3%	2%	3%	4%
ECE1	3%	2%	4%	3%
EIF2AK4	3%	3%	3%	3%
EPHA7	3%	2%	3%	3%
ESPL1	3%	2%	4%	3%
EYA2	3%	3%	2%	3%
FAM227B	3%	2%	4%	2%
FARP2	3%	2%	4%	3%
FCN1	3%	2%	4%	3%
FGD4	3%	2%	2%	4%
FGFR1	3%	3%	4%	2%
FSIP2	3%	3%	3%	2%
GABRA3	3%	3%	2%	3%
GALNT2	3%	2%	2%	5%
GATSL1	3%	2%	5%	2%
GC	3%	3%	3%	3%
GFM1	3%	1%	3%	4%
GGT1	3%	2%	2%	4%
GLT8D2	3%	3%	1%	3%
GOLIM4	3%	2%	5%	2%
GPC5	3%	2%	2%	4%
HEATR5B	3%	2%	3%	3%
HELLS	3%	3%	2%	3%
HHLA1	3%	2%	4%	3%
IFT43	3%	2%	2%	4%
IGF2BP1	3%	3%	2%	4%
IGSF1	3%	2%	5%	3%
IGSF22	3%	2%	2%	4%
IK	3%	2%	3%	4%
IPO9	3%	3%	3%	3%
ITGA6	3%	2%	3%	3%

ITIH4	3%	2%	4%	2%
JAKMIP2	3%	2%	2%	5%
KDM4C	3%	2%	3%	3%
KIAA1468	3%	2%	2%	4%
KIAA2026	3%	2%	3%	3%
KMT2B	3%	2%	5%	3%
KRBOX1	3%	2%	4%	3%
KRTAP5-10	3%	3%	2%	3%
LEPR	3%	3%	3%	3%
LLGL2	3%	1%	4%	4%
LPCAT1	3%	2%	4%	4%
MAU2	3%	2%	4%	2%
MGA	3%	3%	1%	4%
MLH1	3%	3%	1%	4%
MOV10	3%	2%	3%	4%
MTIF2	3%	2%	3%	3%
MYCBPAP	3%	3%	2%	3%
MYSM1	3%	3%	4%	1%
NAALAD2	3%	2%	3%	3%
NCSTN	3%	3%	4%	1%
NLRP1	3%	3%	2%	3%
OPN3	3%	2%	4%	3%
OTUD4	3%	1%	3%	4%
PARK2	3%	2%	3%	4%
PARP14	3%	2%	5%	2%
PDSS1	3%	2%	7%	0%
PGAP1	3%	2%	3%	3%
PGM2L1	3%	3%	2%	3%
PI4K2A	3%	2%	2%	4%
PKP4	3%	2%	2%	4%
PMPCA	3%	2%	3%	3%
POM121L9P	3%	3%	4%	2%
POTEE	3%	3%	3%	3%
PRDM10	3%	2%	2%	4%
PRTG	3%	3%	2%	2%
PSG11	3%	2%	4%	4%
PTGS2	3%	2%	3%	3%
PTPRK	3%	3%	3%	2%
RAD54B	3%	3%	3%	3%
RECQL	3%	2%	5%	3%
ROPN1	3%	4%	2%	1%
RRP12	3%	3%	2%	3%
SCAF4	3%	3%	3%	2%
SCAI	3%	3%	3%	2%
SCAPER	3%	2%	5%	3%
SCN7A	3%	2%	5%	3%
SECISBP2L	3%	2%	5%	2%
SEMA6C	3%	2%	3%	4%
SERINC1	3%	3%	5%	1%
SH3PXD2B	3%	2%	2%	4%
SIDT2	3%	3%	3%	3%
SIK3	3%	3%	2%	3%
SLC22A14	3%	3%	2%	2%
SLC26A8	3%	2%	2%	4%
SLC37A1	3%	1%	5%	3%
SLC38A9	3%	3%	2%	4%
SLC6A13	3%	3%	3%	2%
SLCO1A2	3%	2%	5%	2%
SOAT1	3%	2%	3%	3%
SPTBN5	3%	3%	3%	2%
SRRT	3%	2%	3%	4%
STAG3L5P-PVRIG2P-PILRB	3%	1%	4%	4%

STAT6	3%	3%	3%	3%
STK38	3%	3%	2%	3%
SYN2	3%	1%	4%	4%
TAF6	3%	2%	4%	2%
TEPP	3%	3%	4%	1%
TIAF1	3%	2%	3%	4%
TIAM2	3%	2%	2%	4%
TNRC18	3%	2%	4%	3%
TNS1	3%	2%	4%	3%
TRERF1	3%	2%	4%	2%
TTC13	3%	2%	2%	4%
UBA6	3%	2%	3%	4%
VCL	3%	2%	4%	3%
WDR60	3%	3%	2%	3%
WDR93	3%	3%	2%	3%
XPNPEP3	3%	2%	3%	3%
ZFYVE9	3%	2%	3%	3%
ZNF236	3%	2%	3%	4%
ZNF496	3%	2%	4%	3%
ZNF763	3%	3%	2%	3%
ABCC12	3%	3%	3%	2%
ABL1	3%	3%	3%	2%
ABTB2	3%	1%	2%	5%
ACBD5	3%	2%	2%	4%
ACTA2	3%	2%	3%	3%
ADAM10	3%	2%	3%	4%
AFF1	3%	2%	3%	4%
AGBL3	3%	2%	4%	2%
ALOXE3	3%	2%	3%	3%
ALPK1	3%	2%	4%	3%
ANKFN1	3%	2%	3%	4%
ANKRD55	3%	3%	2%	3%
ARHGAP28	3%	3%	2%	3%
ARL17B	3%	3%	2%	3%
ARNT2	3%	2%	4%	2%
ARSB	3%	3%	3%	3%
ASCC2	3%	2%	2%	4%
ASMT	3%	2%	2%	4%
ATF6	3%	3%	3%	2%
ATP6V0A2	3%	3%	4%	2%
BACH1	3%	3%	2%	3%
BAI1	3%	2%	4%	2%
BBS1	3%	3%	3%	2%
BDH1	3%	3%	2%	2%
BMP5	3%	3%	3%	2%
BOC	3%	3%	3%	2%
BOD1L1	3%	1%	4%	4%
C4BPA	3%	2%	5%	3%
CACNA1C-AS1	3%	1%	4%	3%
CAGE1	3%	2%	3%	3%
CAMSAP2	3%	2%	1%	4%
CAPN14	3%	2%	3%	3%
CAPN8	3%	2%	5%	3%
CAT	3%	3%	4%	2%
CCDC13	3%	3%	3%	2%
CCDC151	3%	2%	4%	3%
CCDC159	3%	2%	4%	2%
CCDC60	3%	2%	3%	3%
CCDC66	3%	2%	3%	3%
CCT3	3%	2%	2%	4%
CCT5	3%	2%	3%	3%
CD46	3%	3%	3%	2%

CD99	3%	2%	3%	3%
CDH26	3%	3%	3%	2%
CDR2	3%	3%	4%	1%
CEP152	3%	1%	4%	4%
COL1A1	3%	2%	2%	4%
CP	3%	2%	2%	4%
CPNE8	3%	2%	2%	4%
CTNNAL1	3%	3%	2%	1%
CTNND2	3%	2%	3%	3%
CWH43	3%	2%	2%	4%
DAPK1	3%	1%	3%	4%
DCAF6	3%	3%	3%	1%
DCST2	3%	2%	3%	3%
DDX3X	3%	1%	4%	5%
DDX4	3%	3%	1%	3%
DENND2C	3%	3%	2%	2%
DIAPH1	3%	3%	2%	3%
DNM2	3%	1%	3%	5%
DPY19L2P2	3%	3%	3%	2%
DPY19L3	3%	3%	1%	3%
DSE	3%	2%	2%	4%
EFCAB5	3%	2%	1%	4%
ELF1	3%	2%	3%	4%
EPB41L4B	3%	2%	3%	3%
EPHA5	3%	1%	3%	4%
EPS15L1	3%	1%	4%	4%
ERMARD	3%	2%	3%	4%
ESYT1	3%	2%	3%	4%
FAAH2	3%	1%	3%	4%
FAM13C	3%	3%	3%	2%
FAM86DP	3%	2%	4%	3%
FAM98A	3%	1%	5%	3%
FIG4	3%	2%	3%	3%
FILIP1	3%	2%	5%	2%
FMN1	3%	1%	4%	4%
FOXP2	3%	3%	5%	1%
FRMD7	3%	2%	3%	3%
FSD1L	3%	3%	2%	3%
GALC	3%	2%	2%	3%
GBP7	3%	2%	4%	2%
GFPT2	3%	2%	4%	3%
GLDC	3%	2%	4%	3%
GLP1R	3%	2%	2%	3%
GPR125	3%	1%	3%	4%
GRIA4	3%	2%	3%	3%
GYPB	3%	2%	3%	3%
GYS2	3%	3%	3%	3%
HDAC9	3%	2%	4%	2%
HEATR4	3%	3%	2%	2%
HHIPL2	3%	2%	4%	3%
HK1	3%	2%	3%	4%
HNRNPM	3%	2%	3%	3%
HRNR	3%	2%	2%	4%
IGF1R	3%	2%	3%	3%
IMPG2	3%	3%	2%	3%
INTS1	3%	2%	3%	3%
IQCG	3%	2%	4%	2%
JAG1	3%	1%	5%	3%
KCNT1	3%	2%	3%	3%
KDM5A	3%	2%	3%	3%
KDM6A	3%	2%	3%	3%
KIAA0100	3%	2%	1%	5%

KIAA1033	3%	3%	3%	2%
KIAA1211	3%	1%	4%	4%
LTBP2	3%	1%	3%	4%
MAP3K14-AS1	3%	3%	2%	2%
MAST3	3%	1%	4%	4%
MCC	3%	3%	3%	3%
MCF2L	3%	2%	4%	3%
ME2	3%	3%	2%	2%
MIPEP	3%	2%	3%	3%
MPHOSPH9	3%	3%	3%	3%
MRPL43	3%	2%	4%	3%
MUC19	3%	2%	2%	4%
NADSYN1	3%	2%	3%	3%
NAPB	3%	3%	2%	3%
NARS2	3%	4%	1%	2%
NBPF8	3%	3%	1%	3%
NCKAP1	3%	2%	2%	4%
NEK11	3%	1%	4%	4%
NFX1	3%	2%	2%	4%
NID1	3%	1%	4%	3%
NLGN3	3%	2%	3%	3%
NLGN4X	3%	2%	2%	4%
NLRC4	3%	4%	1%	2%
NOS2	3%	2%	3%	3%
NTRK2	3%	2%	3%	4%
OPHN1	3%	2%	4%	2%
PARP8	3%	2%	3%	4%
PASK	3%	2%	4%	2%
PCNXL4	3%	2%	3%	3%
PEBP4	3%	3%	3%	2%
PHIP	3%	3%	2%	3%
PIK3C2A	3%	2%	4%	2%
PITRM1	3%	2%	3%	3%
PITRM1-AS1	3%	2%	3%	3%
POF1B	3%	3%	1%	3%
POLDIP3	3%	3%	1%	3%
PPP1R9A	3%	2%	4%	3%
PRRC1	3%	3%	1%	3%
PTDSS1	3%	2%	3%	3%
QSER1	3%	2%	1%	5%
RAPGEF4	3%	3%	3%	2%
RBBP6	3%	3%	2%	3%
RBBP7	3%	3%	1%	4%
RHCE	3%	3%	2%	2%
SAMD7	3%	2%	4%	3%
SEC13	3%	3%	1%	4%
SEZ6L2	3%	3%	1%	4%
SHQ1	3%	4%	2%	1%
SHROOM2	3%	2%	2%	4%
SLC1A6	3%	2%	3%	3%
SLC4A1AP	3%	2%	4%	3%
SLC6A11	3%	3%	3%	3%
SLC9A8	3%	3%	3%	3%
SLC9B1	3%	3%	3%	3%
SMG9	3%	3%	3%	2%
SPATA31C2	3%	2%	2%	3%
SPEN	3%	3%	2%	3%
SPICE1	3%	3%	2%	3%
SPTB	3%	1%	3%	4%
SPTBN4	3%	3%	3%	2%
SRPK2	3%	1%	4%	3%
SSR1	3%	2%	2%	3%

ST5	3%	3%	2%	3%
TBC1D1	3%	3%	0%	4%
TBC1D28	3%	2%	2%	3%
TENM3	3%	2%	3%	3%
TMC3	3%	2%	3%	4%
TMEM214	3%	2%	2%	4%
TTC21B	3%	2%	2%	4%
UBE4B	3%	3%	1%	4%
VPS39	3%	3%	1%	3%
WDR12	3%	2%	3%	3%
WDR7	3%	1%	3%	4%
WWC1	3%	2%	3%	3%
XPOT	3%	2%	5%	2%
YLPM1	3%	3%	2%	2%
ZBTB49	3%	3%	3%	3%
ZC3H7A	3%	1%	3%	4%
ZMYND8	3%	3%	2%	3%
ZNF114	3%	3%	3%	3%
ZNF462	3%	2%	3%	3%
ZNF587	3%	2%	3%	4%
ZWILCH	3%	2%	3%	3%
ABCB4	2%	2%	3%	3%
ABCC11	2%	2%	2%	4%
ACAN	2%	2%	4%	3%
ACOX1	2%	2%	3%	3%
ACSBG2	2%	2%	4%	3%
ACTN1	2%	2%	3%	3%
ADAM22	2%	2%	3%	2%
ADCY1	2%	1%	3%	5%
AHCYL2	2%	2%	4%	3%
AHI1	2%	2%	3%	3%
AKAP2	2%	2%	2%	3%
ARHGEF26	2%	3%	2%	1%
ARHGEF37	2%	3%	2%	3%
ARNTL2	2%	2%	4%	1%
ASAP2	2%	2%	2%	3%
ATXN7L1	2%	1%	5%	3%
BUB1	2%	2%	3%	4%
C8orf44-SGK3	2%	2%	4%	3%
CASP8	2%	2%	4%	3%
CASZ1	2%	2%	3%	3%
CC2D1A	2%	2%	2%	3%
CCDC38	2%	2%	3%	3%
CCDC73	2%	2%	4%	2%
CCDC88C	2%	2%	2%	4%
CCSER2	2%	2%	2%	3%
CCT6B	2%	2%	4%	3%
CD36	2%	1%	3%	4%
CD44	2%	2%	3%	3%
CDHR3	2%	2%	3%	3%
CELA2B	2%	2%	3%	3%
CFHR3	2%	2%	3%	3%
CLCN2	2%	3%	2%	2%
CLCN6	2%	1%	3%	4%
CREB5	2%	2%	3%	3%
CTR9	2%	2%	2%	4%
CWC25	2%	1%	2%	6%
DCLK2	2%	3%	2%	2%
DDX1	2%	2%	3%	3%
DGKD	2%	1%	3%	4%
DHRS4L2	2%	2%	2%	4%
DLG5	2%	2%	2%	3%

DNAJC16	2%	1%	3%	4%
DPYSL2	2%	3%	4%	1%
DRP2	2%	2%	3%	3%
DUOX1	2%	2%	4%	3%
DUSP22	2%	2%	2%	4%
EFCAB13	2%	2%	3%	3%
EIF4G2	2%	3%	3%	2%
ELMO2	2%	2%	3%	3%
EPS15	2%	2%	3%	3%
EPS8	2%	2%	2%	4%
ERC2	2%	3%	1%	2%
ERCC8	2%	3%	2%	2%
ESYT3	2%	2%	4%	3%
EXOC2	2%	2%	3%	3%
EYA4	2%	2%	3%	4%
FAM129C	2%	3%	0%	4%
FAM134C	2%	2%	2%	4%
FAM205B	2%	2%	2%	4%
FAM66C	2%	2%	3%	3%
FAM86B3P	2%	1%	4%	4%
FAM86FP	2%	2%	2%	3%
FGD5	2%	2%	3%	2%
FGD6	2%	2%	3%	3%
FGFR2	2%	2%	1%	4%
FMNL3	2%	1%	3%	4%
FRMD4B	2%	3%	2%	2%
GCLC	2%	3%	2%	2%
GDPD2	2%	2%	3%	3%
GGT3P	2%	2%	2%	3%
GLYR1	2%	2%	2%	3%
GMCL1	2%	2%	4%	2%
GPR110	2%	2%	3%	3%
GPR137B	2%	1%	4%	3%
GRIK1	2%	3%	2%	3%
HAPLN4	2%	2%	3%	3%
HEATR6	2%	2%	2%	3%
HIPK3	2%	2%	2%	4%
HPR	2%	2%	2%	3%
HPS4	2%	3%	4%	1%
IGSF9	2%	2%	3%	3%
INSR	2%	2%	3%	3%
INTS10	2%	1%	2%	4%
ITGAE	2%	2%	3%	4%
ITGB2	2%	2%	4%	1%
JAK1	2%	2%	1%	4%
KCNQ5	2%	1%	4%	3%
KIAA1407	2%	1%	3%	4%
KIF1A	2%	1%	3%	4%
LOXL3	2%	2%	3%	3%
LRGUK	2%	1%	3%	4%
LRRC37A6P	2%	2%	2%	4%
LRSAM1	2%	2%	3%	3%
LYN	2%	2%	1%	4%
MAN2A1	2%	1%	4%	3%
MED13L	2%	2%	2%	3%
MEGF10	2%	3%	3%	1%
MIR555	2%	3%	2%	3%
MLLT10	2%	2%	3%	4%
MMP16	2%	3%	4%	1%
MRPS7	2%	3%	3%	1%
MTHFD1	2%	3%	2%	2%
MTMR8	2%	2%	2%	3%

MYO1E	2%	2%	3%	3%
MYO6	2%	2%	5%	2%
NDST3	2%	2%	2%	4%
NDUFA6-AS1	2%	1%	4%	4%
NDUFAF6	2%	2%	3%	3%
NEK1	2%	3%	2%	3%
NEK5	2%	3%	2%	2%
NFXL1	2%	3%	2%	1%
NOS1AP	2%	2%	3%	3%
NOX4	2%	2%	3%	3%
NPR1	2%	2%	2%	4%
OR4N3P	2%	2%	2%	4%
ORC3	2%	3%	2%	2%
PA2G4	2%	1%	3%	4%
PACS1	2%	2%	3%	3%
PAPOLG	2%	3%	2%	3%
PARD3B	2%	2%	3%	3%
PCBP3	2%	2%	5%	2%
PCM1	2%	2%	3%	3%
PDE8B	2%	2%	3%	3%
PGC	2%	2%	3%	2%
PHKB	2%	2%	3%	4%
PHRF1	2%	3%	1%	3%
PILRB	2%	1%	4%	3%
PIPOX	2%	2%	2%	3%
PKP2	2%	2%	3%	3%
PLA2R1	2%	2%	4%	3%
PLEKHG1	2%	2%	3%	3%
POLQ	2%	2%	3%	3%
POLR2J3	2%	3%	3%	2%
POSTN	2%	2%	3%	3%
PRAMEF4	2%	3%	1%	3%
PRKD3	2%	3%	1%	3%
PRPF8	2%	2%	4%	2%
PSEN2	2%	2%	3%	3%
PSG4	2%	2%	2%	3%
PTPRG-AS1	2%	3%	1%	2%
PTPRU	2%	3%	2%	3%
RAB3GAP1	2%	2%	1%	4%
RBM33	2%	2%	3%	3%
RBM5	2%	3%	1%	3%
RDH5	2%	2%	2%	4%
RFWD2	2%	1%	3%	4%
RGS3	2%	3%	2%	3%
RMND1	2%	2%	3%	3%
RRM1	2%	2%	3%	3%
RTTN	2%	3%	2%	2%
SDCCAG3	2%	3%	2%	3%
SEC31A	2%	2%	2%	4%
SEMA3A	2%	3%	2%	3%
SETD5	2%	2%	3%	3%
SLC18A1	2%	2%	2%	3%
SLC25A14	2%	3%	2%	2%
SLC5A6	2%	2%	2%	3%
SLC6A6	2%	2%	3%	3%
SNCAIP	2%	2%	3%	3%
SOX6	2%	2%	3%	3%
SPEG	2%	3%	3%	2%
SPTBN2	2%	2%	4%	3%
ST7	2%	2%	3%	3%
STARD3NL	2%	1%	3%	4%
SUGP1	2%	2%	4%	2%

SYNE3	2%	2%	3%	3%
SYNJ2	2%	3%	2%	2%
TARBP1	2%	2%	3%	3%
TECTA	2%	1%	3%	4%
TEX35	2%	2%	3%	3%
TGS1	2%	2%	2%	3%
THBS3	2%	2%	2%	3%
THBS4	2%	2%	5%	1%
TLDC2	2%	2%	4%	3%
TLK1	2%	2%	4%	3%
TMCO3	2%	1%	2%	4%
TMEM151B	2%	2%	4%	2%
TMEM189	2%	2%	3%	3%
TNRC6A	2%	1%	3%	4%
TP53TG3C	2%	2%	4%	2%
TRAF5	2%	2%	3%	3%
TRIP11	2%	3%	1%	3%
UBXN8	2%	3%	2%	3%
UNC13D	2%	2%	3%	4%
USP36	2%	2%	3%	3%
VCP	2%	2%	1%	4%
VPS72	2%	2%	2%	3%
WDR19	2%	3%	2%	2%
WDR66	2%	3%	2%	3%
WDR90	2%	1%	3%	4%
WNK4	2%	3%	1%	3%
ZC3H14	2%	2%	2%	3%
ZFP91	2%	2%	4%	3%
ZMAT4	2%	3%	1%	4%
ZNF117	2%	2%	2%	4%
ZNF705E	2%	3%	2%	3%
ABCC6	2%	3%	3%	1%
ABLIM1	2%	2%	4%	2%
ACSM4	2%	2%	3%	3%
ADAM21P1	2%	3%	1%	3%
ADAMTS19	2%	2%	2%	3%
AIFM1	2%	2%	2%	3%
ALG5	2%	2%	4%	3%
ALS2	2%	1%	3%	4%
ANGPT1	2%	1%	4%	3%
ANKRD7	2%	3%	2%	3%
ANTXR2	2%	2%	3%	2%
AP1G1	2%	3%	2%	2%
ARHGAP17	2%	1%	1%	5%
ARHGAP19-SLIT1	2%	2%	2%	4%
ARHGAP24	2%	2%	2%	3%
ARMC10	2%	3%	2%	1%
ARMC8	2%	2%	4%	3%
ARPC3	2%	3%	3%	2%
ATP2B1	2%	2%	3%	2%
ATP2C2	2%	2%	4%	2%
BCOR	2%	2%	2%	3%
BCORL1	2%	2%	2%	3%
BMP1	2%	2%	3%	2%
BRIP1	2%	2%	2%	3%
C14orf39	2%	2%	3%	2%
C6orf201	2%	2%	2%	3%
CABIN1	2%	2%	2%	3%
CACNB2	2%	2%	2%	4%
CALD1	2%	2%	2%	3%
CAPN11	2%	2%	3%	2%
CASQ2	2%	2%	3%	2%

CBFB	2%	3%	1%	3%
CBLB	2%	2%	3%	3%
CD1D	2%	1%	3%	4%
CD97	2%	3%	2%	2%
CECR2	2%	2%	3%	3%
CELA3B	2%	3%	3%	1%
CERKL	2%	1%	3%	4%
CERS2	2%	1%	3%	4%
CHAT	2%	3%	3%	2%
CHFR	2%	2%	3%	3%
CHTOP	2%	3%	1%	3%
CLMN	2%	2%	2%	3%
COL18A1	2%	1%	4%	3%
COL6A1	2%	2%	3%	3%
COPB2	2%	2%	3%	3%
CPNE1	2%	2%	4%	2%
CSHL1	2%	2%	2%	3%
CTCF	2%	3%	2%	2%
CYP11B2	2%	2%	3%	3%
CYP4F3	2%	2%	1%	3%
DACH2	2%	2%	3%	2%
DCAF5	2%	2%	2%	3%
DDX46	2%	1%	1%	5%
DECR1	2%	3%	2%	1%
DENND5A	2%	2%	4%	2%
DHX37	2%	1%	2%	4%
DLG4	2%	2%	3%	3%
DNAJC10	2%	3%	2%	2%
DPP8	2%	3%	2%	3%
EFEMP1	2%	1%	3%	4%
EHBP1	2%	2%	2%	3%
EML2	2%	3%	2%	2%
EPB41L3	2%	1%	5%	3%
ERG	2%	2%	3%	3%
ETNPPL	2%	3%	3%	2%
F13A1	2%	3%	2%	2%
FAM149A	2%	2%	2%	3%
FAM24A	2%	3%	2%	2%
FAM73A	2%	3%	2%	2%
FHAD1	2%	2%	3%	2%
FKBP9L	2%	2%	2%	3%
FLT1	2%	2%	4%	2%
FMNL2	2%	2%	3%	3%
FRMD3	2%	2%	3%	3%
FRMPD1	2%	1%	3%	3%
FXR1	2%	1%	4%	3%
FYB	2%	1%	3%	3%
GAPVD1	2%	2%	2%	3%
GNAS	2%	3%	2%	2%
GOLGA6L5P	2%	2%	4%	1%
GOLGA8S	2%	3%	1%	3%
GPNMB	2%	2%	3%	3%
GPR64	2%	2%	2%	3%
GRIN2B	2%	1%	3%	4%
GSTA1	2%	3%	2%	3%
HELZ2	2%	2%	3%	3%
HERC6	2%	2%	2%	3%
IL4I1	2%	1%	3%	4%
INPP5A	2%	2%	2%	3%
INTS6	2%	2%	4%	2%
IPO11	2%	3%	2%	2%
IPO8	2%	1%	3%	4%

ITGBL1	2%	2%	2%	3%
ITLN1	2%	2%	3%	2%
JADE3	2%	3%	2%	2%
KCNIP4	2%	2%	3%	2%
KCTD5	2%	2%	4%	3%
KDM1B	2%	3%	3%	1%
KIAA0226	2%	2%	3%	3%
KIAA1244	2%	1%	4%	4%
KIAA1524	2%	3%	2%	2%
KIF13B	2%	2%	3%	2%
KLC1	2%	2%	4%	3%
KLKB1	2%	2%	3%	2%
KRT83	2%	3%	2%	2%
KRTAP5-4	2%	3%	1%	3%
LGR4	2%	2%	3%	3%
LPIN1	2%	1%	4%	4%
LRRC41	2%	1%	4%	3%
MAATS1	2%	1%	2%	4%
MAGEC1	2%	2%	3%	2%
MAP3K3	2%	3%	1%	3%
MARK1	2%	2%	3%	3%
MED14	2%	2%	2%	3%
MEGF8	2%	3%	2%	2%
MEIS2	2%	2%	2%	3%
MEP1B	2%	2%	3%	3%
MET	2%	2%	1%	4%
MICAL1	2%	1%	4%	3%
MIR4728	2%	1%	1%	5%
MON2	2%	3%	1%	3%
MS4A14	2%	2%	3%	2%
MTMR7	2%	1%	3%	4%
MUC20	2%	2%	4%	2%
MX2	2%	3%	2%	2%
NCOA1	2%	1%	2%	4%
NDUFS1	2%	2%	2%	4%
NEDD9	2%	3%	1%	2%
NF1P2	2%	3%	2%	3%
NFKBIZ	2%	3%	1%	2%
NIPA1	2%	2%	3%	3%
NIT1	2%	2%	3%	3%
NLRP8	2%	1%	4%	4%
NOX3	2%	2%	2%	3%
NR1H4	2%	2%	2%	4%
NRK	2%	2%	3%	2%
NT5C1B	2%	2%	2%	3%
NUDCD1	2%	1%	4%	4%
OAZ3	2%	2%	3%	2%
OGDH	2%	2%	2%	3%
OSCP1	2%	2%	2%	3%
OSMR	2%	2%	2%	3%
PAPLN	2%	2%	0%	4%
PARP12	2%	2%	2%	3%
PCCA	2%	1%	3%	4%
PDIA5	2%	1%	3%	3%
PER3	2%	3%	2%	2%
PEX1	2%	2%	4%	1%
PHLDB3	2%	3%	2%	2%
PIGO	2%	2%	3%	3%
PLEKHA6	2%	2%	3%	3%
PLEKHA7	2%	3%	3%	1%
PLEKHG2	2%	2%	3%	3%
PLXDC2	2%	2%	4%	2%

PLXNB2	2%	2%	2%	3%
POLR3A	2%	2%	3%	2%
PON1	2%	2%	4%	2%
POP1	2%	2%	2%	4%
PPARGC1A	2%	2%	2%	3%
PPFIA3	2%	2%	2%	3%
PPM1B	2%	2%	3%	3%
PRDM16	2%	2%	2%	3%
PROSER1	2%	3%	1%	3%
PRPF40A	2%	1%	3%	4%
PSAT1	2%	2%	4%	2%
PTPN21	2%	2%	4%	2%
PTPN3	2%	2%	2%	4%
PXDN	2%	2%	4%	2%
RASA3	2%	2%	3%	3%
RASGEF1B	2%	2%	2%	3%
RBBP5	2%	2%	2%	3%
RECQL4	2%	2%	4%	3%
RERE	2%	2%	3%	3%
RGPD4	2%	1%	3%	4%
RHOT1	2%	3%	2%	2%
RMDN1	2%	3%	3%	1%
ROPN1B	2%	2%	1%	4%
RRP1	2%	3%	4%	1%
RSRC2	2%	2%	4%	3%
RTCB	2%	2%	4%	3%
RXFP2	2%	2%	3%	2%
SCN10A	2%	2%	2%	3%
SCYL3	2%	2%	3%	3%
SDHAP3	2%	2%	2%	3%
SEPT7P9	2%	3%	2%	2%
SERF2	2%	2%	3%	2%
SERPINB7	2%	3%	1%	2%
SFMBT2	2%	1%	3%	3%
SHC3	2%	3%	1%	3%
SIMC1	2%	3%	2%	3%
SKA3	2%	3%	3%	1%
SLC18A2	2%	1%	3%	3%
SLC22A20	2%	2%	2%	3%
SLC4A11	2%	3%	2%	2%
SLC51A	2%	3%	2%	3%
SLC6A12	2%	2%	2%	3%
SLCO1C1	2%	2%	4%	2%
SLFN12L	2%	2%	2%	3%
SMARCC2	2%	2%	2%	3%
SMYD5	2%	2%	2%	3%
SNORD21	2%	2%	2%	3%
STAB1	2%	1%	5%	2%
SUPT16H	2%	2%	2%	4%
TAF10	2%	3%	3%	2%
TCTN2	2%	2%	3%	2%
TMBIM6	2%	2%	2%	3%
TMEM132D	2%	2%	2%	3%
TMEM189-UBE2V1	2%	2%	2%	3%
TNIP3	2%	2%	2%	4%
TNNT2	2%	2%	3%	3%
TNRC6C	2%	2%	4%	2%
TRIM33	2%	2%	2%	3%
TRO	2%	1%	3%	3%
TSGA10	2%	2%	3%	3%
TTLL3	2%	2%	2%	3%
TULP3	2%	1%	4%	3%

UBR1	2%	1%	3%	3%
UGT3A1	2%	2%	2%	3%
UNK	2%	2%	3%	3%
URI1	2%	2%	2%	3%
USP11	2%	2%	2%	3%
USP20	2%	1%	4%	3%
VCAM1	2%	3%	2%	2%
VPS33A	2%	3%	2%	1%
WAPAL	2%	3%	1%	3%
XAB2	2%	2%	2%	3%
XPO4	2%	2%	2%	3%
YME1L1	2%	3%	2%	3%
ZBTB20	2%	3%	1%	2%
ZCCHC17	2%	3%	1%	1%
ZEB1	2%	2%	3%	2%
ZMAT2	2%	3%	3%	2%
ZNF385D	2%	2%	1%	4%
ZNF571	2%	3%	1%	2%
ZNF578	2%	2%	3%	2%
AAMDC	2%	2%	3%	2%
AASS	2%	1%	3%	4%
ABCA7	2%	1%	4%	3%
ABCD3	2%	1%	4%	3%
ACAA1	2%	2%	3%	2%
ACAD10	2%	2%	2%	3%
ADAM7	2%	2%	3%	2%
ADAMTS18	2%	1%	3%	4%
AHCTF1P1	2%	2%	2%	3%
AMY2B	2%	2%	2%	3%
AMZ2P1	2%	2%	2%	3%
ANKFY1	2%	2%	2%	3%
ANKRD32	2%	3%	2%	2%
ANO6	2%	1%	2%	4%
ANXA11	2%	3%	1%	3%
AP2A2	2%	2%	2%	3%
APAF1	2%	2%	2%	3%
APOH	2%	2%	3%	3%
ARHGAP26	2%	2%	3%	3%
ARHGEF10L	2%	1%	3%	4%
ARL10	2%	2%	1%	3%
ARMC9	2%	2%	2%	3%
ASIC2	2%	2%	3%	3%
ATIC	2%	3%	2%	2%
ATP12A	2%	2%	2%	3%
ATP8B3	2%	3%	3%	1%
ATRIP	2%	2%	1%	3%
BTAF1	2%	2%	2%	3%
BTG4	2%	3%	2%	2%
C12orf40	2%	2%	3%	3%
C12orf43	2%	3%	0%	3%
C16orf58	2%	2%	2%	2%
C1QTNF9B-AS1	2%	2%	3%	1%
C2orf42	2%	2%	2%	3%
C5orf34	2%	2%	4%	2%
CALCR	2%	2%	3%	3%
CARD8	2%	2%	2%	3%
CASC5	2%	2%	2%	3%
CBS	2%	2%	3%	3%
CCDC136	2%	2%	2%	3%
CCDC140	2%	2%	2%	3%
CCDC50	2%	1%	3%	3%
CCDC69	2%	2%	3%	3%

CCZ1	2%	2%	2%	3%
CD109	2%	2%	3%	2%
CD177	2%	2%	3%	3%
CD300LD	2%	2%	2%	3%
CDKL1	2%	2%	3%	3%
CDYL	2%	1%	2%	4%
CEP63	2%	1%	2%	4%
CEP85L	2%	2%	3%	2%
CFL1	2%	2%	3%	2%
CGB2	2%	1%	3%	4%
CHCHD3	2%	3%	2%	1%
CHN2	2%	1%	2%	4%
CKMT2-AS1	2%	2%	2%	3%
CLEC4M	2%	2%	1%	4%
CLIP4	2%	2%	2%	3%
CLTA	2%	2%	4%	2%
CMSS1	2%	2%	2%	3%
CNTD1	2%	2%	1%	3%
CNTN5	2%	1%	3%	4%
CNTNAP4	2%	1%	3%	3%
COL6A2	2%	2%	2%	3%
CPT1C	2%	2%	2%	3%
CPXM2	2%	3%	2%	1%
CWF19L2	2%	2%	2%	3%
DAP3	2%	1%	4%	3%
DCAF17	2%	3%	2%	1%
DCST1	2%	2%	2%	3%
DDX27	2%	2%	2%	2%
DDX31	2%	1%	3%	3%
DEGS1	2%	1%	3%	3%
DHX35	2%	1%	4%	3%
DIAPH3	2%	3%	2%	2%
DLG3	2%	2%	1%	4%
DNAI1	2%	2%	3%	2%
DNAJC17	2%	2%	0%	5%
DNAJC6	2%	2%	3%	3%
DNMT3A	2%	2%	4%	2%
DOCK6	2%	2%	3%	3%
DPYSL3	2%	2%	3%	3%
DSCAML1	2%	1%	3%	4%
DSTYK	2%	2%	2%	2%
DTNB	2%	2%	1%	4%
DYNC2LI1	2%	3%	4%	1%
ECE2	2%	2%	3%	2%
EFTUD1P1	2%	2%	2%	3%
EIF2B3	2%	2%	1%	4%
EIF2S1	2%	3%	1%	2%
EIF4A2	2%	2%	2%	3%
EIF4G1	2%	1%	2%	4%
ENTPD5	2%	2%	2%	3%
EPHA4	2%	1%	2%	4%
ERBB2IP	2%	2%	2%	3%
ERCC5	2%	3%	3%	1%
ESYT2	2%	2%	2%	3%
EVC	2%	2%	3%	2%
EVI5	2%	1%	2%	4%
EXOC3	2%	2%	0%	4%
FAM193A	2%	2%	2%	3%
FAM65B	2%	1%	4%	3%
FAM86A	2%	2%	2%	3%
FBXO38	2%	2%	2%	3%
FKBP15	2%	1%	4%	2%

FKBP9	2%	3%	1%	2%
FLG2	2%	2%	3%	3%
FNBP1	2%	1%	4%	3%
FSTL4	2%	2%	4%	2%
FYTTD1	2%	2%	3%	3%
GABRA6	2%	3%	3%	1%
GDA	2%	2%	2%	3%
GGA2	2%	2%	2%	3%
GGT5	2%	2%	3%	1%
GIMAP5	2%	2%	1%	4%
GORASP2	2%	2%	4%	1%
GPR126	2%	1%	3%	3%
GPR128	2%	2%	2%	2%
HADHA	2%	3%	1%	2%
HBS1L	2%	1%	2%	4%
HCCAT3	2%	2%	3%	2%
HELQ	2%	1%	3%	3%
HERC4	2%	2%	3%	2%
HHAT	2%	3%	2%	2%
HLTF	2%	3%	2%	2%
IGSF3	2%	2%	2%	3%
IL12RB2	2%	2%	2%	4%
IL20RB	2%	2%	3%	2%
IL4R	2%	1%	3%	3%
INPP4B	2%	2%	2%	3%
INTS2	2%	2%	3%	3%
ITGAD	2%	2%	2%	3%
ITGB7	2%	2%	2%	3%
JMJD1C	2%	2%	3%	3%
KCNK2	2%	2%	1%	3%
KIAA0195	2%	1%	5%	3%
KIAA0319L	2%	2%	3%	3%
KIAA0907	2%	2%	3%	2%
KIFAP3	2%	2%	3%	3%
KLHL3	2%	3%	2%	2%
LACE1	2%	3%	3%	1%
LDLR	2%	3%	3%	1%
LIMS1	2%	2%	2%	4%
LIPH	2%	3%	2%	2%
LMF1	2%	2%	3%	3%
LONRF3	2%	3%	1%	3%
LRP4	2%	2%	1%	3%
LRRC37A2	2%	2%	3%	1%
LRRC37A4P	2%	1%	4%	3%
LRRC6	2%	2%	2%	3%
LSAMP	2%	2%	2%	3%
LTF	2%	2%	2%	3%
LTN1	2%	1%	4%	3%
LY9	2%	1%	3%	3%
MAN2B1	2%	1%	2%	4%
MAP2K3	2%	1%	5%	3%
MAP3K13	2%	2%	1%	4%
MAP3K19	2%	2%	3%	2%
MELK	2%	3%	2%	2%
MFSD11	2%	1%	3%	3%
MGAT5	2%	2%	2%	2%
MORF4L1	2%	2%	3%	2%
MPP7	2%	1%	4%	3%
MR1	2%	2%	3%	2%
MRPS22	2%	1%	3%	4%
MRS2	2%	2%	3%	3%
MS4A4A	2%	2%	1%	4%

MS4A6E	2%	2%	3%	3%
MTMR4	2%	2%	2%	2%
NBN	2%	2%	3%	2%
NCAN	2%	2%	2%	3%
NCAPG2	2%	2%	3%	2%
NELL2	2%	2%	1%	3%
NFIA	2%	2%	1%	3%
NLRP4	2%	2%	2%	3%
NMNAT2	2%	2%	4%	2%
NRG1	2%	2%	2%	2%
NT5DC1	2%	3%	2%	2%
OAS2	2%	1%	2%	4%
OGG1	2%	1%	4%	3%
OGT	2%	1%	3%	3%
OSBPL6	2%	2%	1%	4%
OXR1	2%	1%	2%	4%
PABPC1	2%	2%	3%	3%
PCBP2	2%	2%	2%	3%
PCDH19	2%	2%	3%	3%
PCYT1A	2%	2%	3%	3%
PDE6A	2%	2%	1%	4%
PDE9A	2%	3%	1%	3%
PDPK1	2%	2%	2%	3%
PEG3	2%	1%	3%	4%
PEX5L	2%	2%	3%	3%
PFKM	2%	2%	2%	3%
PGBD5	2%	2%	4%	2%
PHF20	2%	2%	4%	2%
PHLPP2	2%	2%	2%	4%
PIK3AP1	2%	2%	3%	2%
PIK3C3	2%	3%	1%	3%
PKD1	2%	1%	4%	2%
PKD1L3	2%	2%	2%	2%
PLA2G4C	2%	3%	1%	3%
PLEK	2%	2%	2%	3%
PLOD2	2%	2%	3%	2%
PLOD3	2%	2%	3%	1%
POC1B	2%	2%	2%	3%
POMT2	2%	2%	3%	3%
PON2	2%	2%	3%	3%
PRC1	2%	2%	0%	4%
PRDM5	2%	2%	3%	3%
PTGER3	2%	2%	2%	3%
PUS10	2%	2%	3%	2%
RAPH1	2%	3%	2%	2%
RBM25	2%	2%	2%	3%
RBM28	2%	2%	2%	3%
RCE1	2%	3%	2%	1%
RECQL5	2%	1%	2%	4%
RFPL1S	2%	2%	1%	4%
RFTN1	2%	2%	2%	3%
RNF31	2%	2%	3%	2%
RPS27L	2%	2%	2%	3%
RUFY1	2%	2%	2%	3%
SBNO1	2%	2%	3%	3%
SCIN	2%	2%	2%	3%
SEC24C	2%	3%	2%	2%
SEC31B	2%	3%	1%	2%
SENP3-EIF4A1	2%	2%	3%	2%
SGK2	2%	2%	1%	3%
SH3RF1	2%	2%	3%	3%
SLC26A3	2%	3%	2%	2%

SLC28A1	2%	2%	4%	2%
SLC43A1	2%	2%	3%	2%
SLMAP	2%	1%	3%	3%
SLX4	2%	2%	2%	3%
SMARCAD1	2%	3%	3%	1%
SMC4	2%	2%	2%	4%
SNRNP27	2%	2%	3%	1%
SOS2	2%	1%	3%	4%
SOX5	2%	3%	1%	3%
SPATA17	2%	2%	3%	3%
SPATA21	2%	2%	2%	2%
SPATA32	2%	3%	2%	1%
SPATA6	2%	2%	4%	2%
STAT1	2%	2%	3%	3%
STK10	2%	2%	4%	1%
STRN3	2%	3%	0%	3%
SULT1A2	2%	3%	2%	2%
SUMF1	2%	3%	1%	3%
SUMF2	2%	3%	2%	2%
TAF4	2%	2%	4%	1%
TAOK3	2%	2%	3%	2%
TATDN1	2%	3%	2%	2%
TBC1D29	2%	1%	6%	2%
TBC1D2B	2%	1%	3%	3%
TEX11	2%	2%	4%	2%
TFCP2	2%	2%	2%	3%
TGM1	2%	2%	4%	2%
TM9SF4	2%	1%	3%	4%
TNFSF12	2%	3%	2%	2%
TNFSF4	2%	3%	2%	2%
TNPO3	2%	2%	3%	3%
TOM1L1	2%	1%	2%	5%
TP63	2%	1%	2%	4%
TPH2	2%	3%	2%	2%
TPTE2P1	2%	2%	2%	3%
TRIM46	2%	1%	3%	3%
TTC14	2%	1%	3%	4%
TTC37	2%	2%	3%	1%
TUBA1C	2%	2%	3%	1%
TUBGCP4	2%	1%	2%	4%
TUBGCP6	2%	2%	2%	2%
UNC5B	2%	2%	3%	1%
UTP14A	2%	1%	2%	4%
WBSCR17	2%	2%	3%	2%
WDR45B	2%	1%	2%	4%
WDR88	2%	2%	2%	4%
XPO7	2%	3%	0%	2%
XYLB	2%	2%	2%	3%
ZBED1	2%	2%	2%	3%
ZCCHC6	2%	2%	3%	3%
ZFHX3	2%	2%	3%	3%
ZFP30	2%	1%	4%	3%
ZNF106	2%	2%	3%	3%
ZNF443	2%	2%	3%	2%
ACAP3	2%	1%	4%	2%
ACO1	2%	2%	2%	3%
ACSL5	2%	2%	0%	3%
ADAL	2%	1%	2%	4%
ADAM11	2%	2%	2%	3%
ADAM12	2%	2%	2%	3%
AKAP6	2%	1%	2%	4%
AKNA	2%	1%	3%	4%

AKR1B10	2%	1%	4%	2%
ALAS2	2%	1%	5%	3%
ANO10	2%	2%	3%	2%
AP2A1	2%	1%	2%	4%
AP3S2	2%	3%	2%	1%
AQP10	2%	2%	2%	3%
AQP4-AS1	2%	2%	2%	2%
ARHGAP44	2%	2%	2%	3%
ARHGEF15	2%	1%	2%	4%
ARHGEF3	2%	2%	2%	3%
ARHGEF35	2%	2%	4%	1%
ARHGEF40	2%	2%	2%	3%
ARID2	2%	2%	2%	3%
ASS1	2%	3%	2%	1%
ASXL2	2%	2%	2%	3%
ATG4A	2%	1%	3%	3%
ATP6V0A1	2%	2%	1%	3%
BCCIP	2%	2%	4%	2%
BMX	2%	2%	2%	2%
BPIFB1	2%	1%	3%	3%
BRINP1	2%	1%	4%	2%
BTN3A1	2%	1%	4%	3%
C6	2%	2%	3%	2%
CADM2	2%	2%	2%	2%
CAMTA2	2%	1%	4%	3%
CAPN2	2%	1%	3%	4%
CC2D2A	2%	2%	2%	2%
CCDC110	2%	2%	3%	3%
CCDC132	2%	1%	3%	3%
CCDC141	2%	1%	4%	3%
CD163	2%	2%	2%	3%
CD300LF	2%	3%	2%	1%
CD8B	2%	1%	1%	4%
CENPU	2%	2%	2%	2%
CEP164	2%	2%	2%	3%
CEP70	2%	2%	4%	2%
CHI3L2	2%	2%	2%	2%
CHL1	2%	2%	1%	3%
CHRD	2%	2%	3%	1%
CLNK	2%	2%	3%	2%
CLSTN1	2%	2%	2%	3%
CNST	2%	2%	2%	3%
COCH	2%	2%	1%	3%
COMMD7	2%	2%	3%	2%
COPB1	2%	1%	3%	3%
CORIN	2%	2%	3%	2%
CPNE9	2%	2%	3%	2%
CRIPAK	2%	3%	2%	1%
CTNNA1	2%	3%	2%	2%
CUL7	2%	2%	2%	2%
CWC27	2%	2%	1%	3%
DAPK2	2%	3%	2%	2%
DDX55	2%	2%	2%	2%
DENND2A	2%	0%	3%	4%
DQX1	2%	1%	4%	3%
DSCC1	2%	1%	2%	3%
DUSP27	2%	1%	4%	3%
DYTN	2%	1%	3%	3%
EBNA1BP2	2%	1%	4%	2%
EFCAB4B	2%	2%	4%	1%
EMC1	2%	1%	3%	3%
EPHA3	2%	2%	2%	3%

ERLEC1	2%	1%	4%	3%
EXO1	2%	2%	2%	3%
FAM153A	2%	2%	3%	2%
FAM196B	2%	3%	2%	1%
FAM65C	2%	1%	4%	2%
FAM81B	2%	3%	2%	1%
FAM86HP	2%	1%	3%	3%
FAR2	2%	2%	3%	1%
FARSB	2%	1%	4%	3%
FCHSD2	2%	3%	2%	1%
FCRL6	2%	2%	1%	4%
FER	2%	2%	3%	3%
FPGS	2%	1%	2%	3%
FRMD5	2%	2%	3%	2%
FXYD5	2%	2%	1%	4%
GALK2	2%	1%	3%	3%
GALNT11	2%	2%	3%	2%
GBP4	2%	3%	2%	1%
GLB1	2%	2%	3%	1%
GLIPR2	2%	2%	2%	3%
GLP2R	2%	2%	1%	3%
GLS	2%	3%	2%	1%
GOLGA1	2%	2%	2%	3%
GPR113	2%	1%	3%	4%
GRAMD3	2%	2%	3%	1%
GRB10	2%	2%	1%	3%
GRIK4	2%	2%	2%	2%
GUCY1A3	2%	2%	2%	3%
GYG2	2%	2%	2%	3%
HERC2P9	2%	3%	3%	1%
HIST1H2AC	2%	2%	3%	2%
HIVEP3	2%	1%	4%	2%
HMGXB3	2%	2%	3%	2%
HOOK1	2%	2%	3%	2%
HORMAD1	2%	2%	3%	1%
IBTK	2%	2%	2%	2%
ICA1L	2%	1%	3%	3%
IDO2	2%	2%	3%	2%
IFT80	2%	1%	3%	3%
IKZF3	2%	2%	2%	3%
IL2RA	2%	1%	4%	2%
IL5RA	2%	2%	3%	2%
INSRR	2%	1%	4%	2%
IQCA1	2%	2%	1%	3%
IRF4	2%	2%	3%	2%
ITFG2	2%	2%	2%	3%
ITGA11	2%	2%	2%	3%
ITGB3	2%	1%	2%	4%
ITGB3BP	2%	2%	3%	2%
JAK2	2%	1%	4%	3%
KAT6B	2%	3%	2%	2%
KCNIP1	2%	2%	2%	2%
KDM4A	2%	1%	3%	4%
KIAA0391	2%	2%	1%	4%
KIAA0753	2%	2%	3%	2%
KIF7	2%	1%	2%	4%
KRT2	2%	2%	3%	3%
L3MBTL4	2%	2%	1%	3%
LALBA	2%	3%	1%	1%
LAMB3	2%	2%	1%	3%
LAMP2	2%	1%	2%	4%
LAYN	2%	2%	2%	3%

LDB2	2%	3%	1%	2%
LGALS9	2%	1%	3%	3%
LMBRD2	2%	1%	5%	1%
LXN	2%	1%	1%	4%
MAPK8IP3	2%	2%	2%	2%
MAPKBP1	2%	1%	3%	3%
MAPT	2%	2%	2%	3%
MAST2	2%	2%	2%	2%
MBNL3	2%	2%	3%	1%
MBTD1	2%	2%	2%	3%
MDM1	2%	2%	1%	4%
MFN1	2%	2%	2%	3%
MICAL3	2%	1%	3%	3%
MIR5188	2%	1%	3%	3%
MIR548N	2%	2%	3%	2%
MME	2%	1%	2%	4%
MORC2	2%	1%	2%	4%
MPRIP	2%	2%	2%	2%
MRPL9	2%	2%	3%	1%
MRPS15	2%	2%	1%	3%
MTFR1	2%	2%	3%	3%
MTMR14	2%	2%	4%	1%
MTMR3	2%	1%	2%	3%
MTUS2	2%	3%	1%	2%
MX1	2%	1%	4%	2%
MYBPC3	2%	2%	3%	3%
MYO1D	2%	2%	2%	3%
N6AMT2	2%	3%	1%	2%
NDE1	2%	2%	2%	3%
NDRG2	2%	1%	4%	2%
NEK9	2%	2%	2%	2%
NELFCD	2%	1%	2%	4%
NISCH	2%	2%	2%	3%
NLRP13	2%	1%	3%	3%
NNT	2%	2%	1%	3%
NOC3L	2%	2%	3%	2%
NOMO3	2%	1%	3%	3%
NOP14	2%	2%	4%	2%
NR1I3	2%	1%	5%	1%
NRP1	2%	2%	3%	2%
OAS3	2%	2%	1%	3%
OBSL1	2%	3%	3%	1%
OFD1	2%	2%	1%	3%
OR2W3	2%	2%	1%	3%
ORC1	2%	2%	2%	3%
OSBPL1A	2%	2%	3%	3%
PALM2-AKAP2	2%	2%	2%	2%
PARP2	2%	2%	2%	2%
PARP9	2%	2%	2%	3%
PCCB	2%	2%	4%	1%
PCSK1	2%	1%	2%	3%
PCYT1B	2%	2%	3%	2%
PDAP1	2%	2%	1%	3%
PDE2A	2%	2%	4%	1%
PDGFD	2%	2%	3%	2%
PDZRN4	2%	2%	3%	2%
PFKFB4	2%	3%	2%	1%
PIK3R4	2%	2%	3%	3%
PIP5K1B	2%	1%	2%	4%
PIWIL4	2%	2%	2%	3%
PLA2G4F	2%	1%	3%	3%
PLCXD1	2%	1%	1%	5%

PMS2P4	2%	1%	3%	4%
PNLIPRP3	2%	1%	4%	3%
PNPLA6	2%	1%	4%	3%
PNPO	2%	2%	2%	2%
PODXL	2%	1%	2%	4%
POLR2J	2%	2%	3%	1%
POTEF	2%	3%	2%	1%
PRB3	2%	1%	3%	3%
PRKCE	2%	2%	1%	3%
PROS1	2%	2%	3%	2%
PRPSAP2	2%	2%	2%	2%
PSG6	2%	1%	3%	3%
PSIP1	2%	2%	3%	2%
PSPC1	2%	3%	2%	1%
PTBP2	2%	3%	3%	1%
PUM1	2%	2%	3%	2%
PWP2	2%	1%	5%	2%
PYHIN1	2%	2%	1%	3%
R3HDM2	2%	2%	2%	3%
RAD9B	2%	2%	2%	3%
RBM26	2%	2%	3%	2%
REN	2%	1%	3%	4%
RFC4	2%	1%	2%	4%
RGPD1	2%	2%	1%	3%
ROR2	2%	1%	3%	3%
RPS6KA1	2%	2%	4%	2%
RRN3P3	2%	2%	3%	1%
RTN4IP1	2%	2%	2%	2%
RUNX1	2%	2%	3%	3%
S100A13	2%	3%	1%	3%
SALL1	2%	2%	4%	1%
SARS2	2%	2%	2%	3%
SCP2	2%	2%	2%	3%
SCUBE1	2%	1%	4%	3%
SEC14L3	2%	1%	2%	3%
SEC24B	2%	2%	2%	3%
SEMA6A	2%	1%	3%	3%
SEPT4	2%	1%	3%	3%
SF3A3	2%	2%	2%	3%
SHE	2%	1%	3%	3%
SIPA1L3	2%	2%	3%	3%
SKP2	2%	1%	3%	4%
SLAMF7	2%	2%	2%	3%
SLC15A1	2%	2%	2%	2%
SLC22A2	2%	2%	3%	2%
SLC24A2	2%	2%	2%	3%
SLC5A12	2%	2%	3%	1%
SMYD2	2%	2%	2%	3%
SPAG1	2%	2%	3%	1%
SSFA2	2%	2%	2%	3%
STK11IP	2%	1%	2%	3%
STK36	2%	2%	2%	3%
STXBP5	2%	1%	4%	3%
SULT1A1	2%	2%	2%	3%
SUPT20H	2%	2%	2%	3%
SWT1	2%	1%	3%	3%
SYS1-DBNDD2	2%	1%	2%	4%
SYTL2	2%	3%	1%	2%
TBC1D15	2%	2%	2%	3%
TCEA1	2%	2%	2%	2%
TDRD5	2%	1%	4%	3%
TDRKH	2%	2%	3%	2%

TGFBR3	2%	2%	3%	2%
THBS1	2%	2%	4%	1%
THOC6	2%	2%	3%	2%
TJP2	2%	1%	3%	3%
TMEM132B	2%	2%	3%	2%
TNS3	2%	3%	3%	1%
TPM1	2%	2%	1%	3%
TPTEP1	2%	2%	2%	2%
TRIM48	2%	2%	2%	3%
TRIM6	2%	1%	2%	4%
TRIM67	2%	2%	4%	1%
TRMT44	2%	3%	2%	1%
TRPM7	2%	1%	1%	4%
TSHZ2	2%	2%	2%	3%
TSPAN9	2%	2%	1%	3%
TUSC3	2%	2%	3%	2%
UBE2F-SCLY	2%	1%	4%	2%
UBE3B	2%	2%	2%	3%
USP45	2%	2%	3%	1%
USP53	2%	2%	1%	3%
WDHD1	2%	2%	4%	1%
WDR35	2%	2%	1%	3%
WHSC1	2%	1%	2%	4%
YY1AP1	2%	1%	4%	3%
ZCCHC2	2%	2%	4%	1%
ZFR	2%	2%	4%	2%
ZNF280C	2%	2%	2%	3%
ZNF521	2%	2%	3%	2%
ZNF573	2%	2%	3%	3%
ZNF618	2%	2%	3%	2%
ZNF69	2%	2%	3%	1%
ZNF695	2%	2%	2%	3%
