# Supplementary material for: An integrative bioinformatics approach reveals coding and non-coding gene variants associated with gene expression profiles and outcome in breast cancer molecular subtypes
Source: Br J Cancer. 2018 Mar 21;118(8):1107–14. doi: 10.1038/s41416-018-0030-0 (PMC5931099; doi:10.1038/s41416-018-0030-0)
Supplement: Supplementary file 5 — Supplementary Table 4 [file 41416_2018_30_MOESM5_ESM.pdf]

Supplementary Table 4. Validation of genes with sequence variations in non-coding regions using whole genome sequencing data of breast cancer	
**Gene**	**All samples (*n* = 117)**
NBPF1	21%
TTN-AS1	14%
BAGE2	32%
TP53	4%
TTN	23%
RYR2	70%
KMT2C	62%
TPTE	15%
PIK3CA	9%
FLG-AS1	9%
CROCCP2	5%
PCDHGA1	20%
PCDHGA2	18%
SYNE1	50%
EIF2B5	6%
PCDHGA3	18%
MUC16	24%
GON4L	21%
HMCN1	49%
PCDHGB1	18%
COL22A1	63%
DMD	79%
USH2A	67%
MUC4	19%
PCDHGA4	17%
PKHD1L1	27%
OR2L13	37%
CR1	21%
SPTA1	13%
CROCC	9%
FCGR3A	5%
PCDHGB2	17%
DNAH8	44%
HYDIN	58%
DMBT1	8%
OBSCN	19%
FCGR2B	6%
UGT1A8	26%
PCDHGA5	17%
PI4KA	28%
RYR3	56%
SPDYE3	2%
NEB	31%
LRP1B	80%
CSF2RA	37%
CACNA1C	56%
DNAH11	39%
PCDHGB3	17%
ZNF512	12%
DDX11	11%
HUS1	3%

DST	51%
NBPF14	3%
CDC27	10%
NIPBL	23%
SYCP2L	16%
ABI3BP	33%
CACNA1E	44%
FRAS1	43%
PCDHGA6	17%
UGT1A10	24%
CEP170	19%
DZIP3	11%
MYO3B	52%
SDK1	74%
SMG1	23%
HBG2	3%
SYNE2	36%
TG	44%
CSMD1	75%
PLEKHB2	7%
PKHD1	56%
MACF1	50%
ABCA13	57%
PCDHGA7	17%
LPA	26%
PCDHGB4	15%
PRKCA	69%
ACSM3	2%
ANAPC1	21%
FANCD2	15%
GPN1	3%
DNAH17	29%
GOLGB1	14%
MUC17	14%
PKD1L1	32%
PREX2	44%
UGT1A6	20%
C1orf112	12%
DOCK2	60%
HDLBP	11%
KDM2A	24%
UGT1A7	21%
VPS13B	61%
VWF	29%
DNAH9	50%
UBR4	13%
ASAP1	48%
CRTC3	17%
CSMD3	77%
ITPR1	44%
KIAA1109	16%
MST1P2	3%
MTHFD1L	50%
MYO3A	46%
PCDHGA8	15%
AHNAK2	5%
CACNA1B	36%

LRP2	37%
DNAH5	32%
PTPRB	26%
DPP4	15%
RYR1	21%
UGT1A4	15%
UPK3B	1%
C16orf45	31%
FREM1	36%
SBF2	47%
STAG2	26%
SULF1	28%
ANKRD20A5P	24%
AVL9	17%
CFH	23%
DNAH3	38%
DNAH7	39%
ESR2	15%
LAMA1	38%
VWA3A	12%
C14orf37	28%
COL6A6	12%
DIP2B	29%
DNAH14	47%
DOCK4	47%
MYH3	3%
TMEM50A	8%
UGGT1	18%
USP34	38%
DNAH10	34%
FIP1L1	7%
LAMA2	60%
LIPE-AS1	28%
MYOM1	29%
PLB1	20%
PTPRD	79%
TPTE2	30%
TPTE2P6	8%
ANKRD36	30%
CDH1	21%
CDKL5	32%
MSTO2P	3%
PDS5B	19%
PDXDC1	22%
RPH3A	20%
SOD2	16%
BAGE5	33%
BCAS1	20%
CUBN	50%
MED12L	43%
PDXDC2P	21%
RPTOR	65%
STAG3L2	4%
SVIL	44%
KALRN	52%
MROH7-TTC4	18%
NFASC	26%

SACS	16%
AAK1	31%
ASXL3	26%
ATP8A2	65%
COL11A1	35%
CSMD2	60%
DYNC2H1	44%
FER1L6	38%
VIT	17%
COL4A5	35%
CYP3A4	3%
DDC	23%
KRT26	2%
PTCH1	9%
RBX1	5%
SLC4A10	43%
TPR	11%
AKAP13	44%
CACNA2D1	58%
KDM5B	9%
PKD2L2	5%
PRKDC	21%
SP140	15%
CEP112	60%
COL3A1	7%
CPNE4	51%
CTNNA2	75%
DPY19L2	19%
EPB41L4A	26%
LAMA3	34%
MST1L	3%
NF1	33%
PCDHGA9	15%
SYCP2	9%
UBR5	21%
ULK4	62%
USP9X	21%
UTRN	46%
ANK2	57%
CACNA1A	55%
CACNA1D	38%
LYST	27%
MYCBP2	20%
NAA38	6%
SLC13A1	12%
SLC26A5	16%
ANXA6	10%
CBWD1	11%
CEP128	43%
FASTKD1	12%
MDN1	21%
MROH2B	16%
PCDH15	76%
PLXNC1	24%
POTEH	21%
PRKAG2	50%
RNF213	37%

FMN2	63%
PPP1R12A	18%
RANBP2	8%
SNAP25-AS1	38%
CCDC121	1%
COL4A6	38%
FN1	9%
FRA10AC1	3%
GREB1	23%
HOOK2	3%
MROH8	21%
PCDHGB6	15%
RAB3GAP2	18%
RGL1	35%
RGPD3	26%
TPO	49%
ZSCAN5A	23%
AGAP5	6%
AXDND1	35%
COL27A1	21%
CR1L	13%
DGKI	48%
KIF21B	12%
NBPF3	19%
PCDHGA10	15%
PLCB1	68%
TBC1D5	49%
TRIM5	3%
TTC27	28%
USP32	37%
A2ML1	15%
COL7A1	4%
DCDC1	23%
FCGR2C	9%
FLG	3%
GARNL3	17%
SNX29P2	13%
VPS8	26%
ADAM32	21%
ANK1	37%
ANO3	51%
CCDC144A	21%
IQGAP1	22%
KCNU1	28%
LEF1	16%
METTL13	3%
MMP26	2%
PDCD11	9%
STAB2	21%
UGT1A3	14%
WDFY4	41%
ZAN	18%
ACACA	39%
AIM1	10%
AKAP9	24%
ANK3	58%
ATP8B2	7%

DNAH2	20%
DYSF	48%
MERTK	21%
NBPF12	11%
SORCS1	69%
TTC34	77%
ZFAT	33%
BAZ2B	32%
CEP290	11%
CNGB3	30%
HERC2P4	8%
IGF2R	21%
RRN3P2	17%
SCFD1	12%
TBCD	35%
TRIO	45%
AHCTF1	15%
AK9	27%
CHD6	26%
COL5A1	39%
DDX12P	20%
FCGR2A	3%
HBE1	1%
MUC5B	4%
MYH4	8%
RGSL1	26%
RHPN2	25%
SAE1	13%
TBC1D3P1-DHX40P1	19%
UNC13C	60%
ZDHHC11	18%
ANKRD20A9P	25%
ARHGEF11	15%
ATAD2B	33%
C5orf42	15%
CDH23	54%
COL19A1	43%
DENND4A	21%
DEPDC5	18%
FBN1	33%
FEZ2	7%
FOLH1	15%
GPATCH1	10%
HNRNPU	2%
ITPR3	12%
LY75	18%
MAGI1	58%
MED12	5%
MYH1	8%
NCOR1	38%
NPR2	3%
NXF5	8%
PIEZO2	55%
RIMS2	64%
SRGAP3	35%
STAG3	8%
THOC2	21%

UBR3	38%
UNC13A	26%
ABCA8	24%
ADAMTS7	13%
AMPH	38%
ASPH	21%
ATM	20%
CENPF	10%
CLTC	12%
COG3	13%
COL12A1	18%
COL13A1	26%
COL14A1	30%
DMGDH	9%
DNM3	59%
HTT	17%
IARS	13%
ITPR2	49%
KIAA1217	70%
LRRK1	26%
NTRK1	14%
PNPT1	15%
PTPRQ	33%
TAB3	17%
TENM1	54%
TYW1B	38%
VPS13D	31%
ZNF142	2%
ZNF277	13%
AR	31%
ASH1L	27%
ATP8A1	30%
DOCK11	33%
FCGBP	16%
MIA3	11%
MMP21	2%
MYH15	22%
MYLK	23%
NOTCH2	33%
OPA1	9%
PKD1L2	27%
PSG8	4%
PTPRN2	84%
RAB11A	2%
RELN	57%
SMG7	13%
UBE2D3	8%
ANKRA2	2%
ANKRD30B	21%
ART3	22%
ASTN1	42%
CACNA2D3	68%
CBX5	4%
CNOT1	18%
DCC	70%
DNAJC13	15%
FBN2	33%

GRIA3	35%
IARS2	8%
IQGAP2	32%
LY75-CD302	21%
MYH11	32%
MYO5A	30%
MYO5B	46%
NEBL	49%
NOMO1	14%
OTOA	20%
PACRGL	8%
PHF12	7%
SLC12A6	15%
USP6	11%
WDR11	12%
ABL2	16%
AKNAD1	8%
ANKEF1	6%
ANO1	18%
BANK1	32%
CDS2	11%
CHD5	15%
CLEC16A	30%
COL1A2	11%
DLEC1	19%
DOCK1	56%
ERMN	2%
FANCA	15%
FER1L6-AS2	20%
FLNB	22%
GALNT8	9%
KCNN3	35%
KIAA1429	9%
LRP1	13%
NAV2	56%
PCNT	22%
RC3H1	6%
SMARCA4	15%
TAF1	29%
TEP1	3%
TTLL5	28%
WDFY3	27%
ACSM1	12%
ADAMTSL3	44%
ANTXR1	21%
ATAD2	9%
CACNA1S	11%
COL4A2	51%
DYNC1H1	14%
EP400	16%
EPHB1	65%
GTF3C3	1%
HECW1	54%
ITGA1	26%
IWS1	7%
KIAA1257	10%
KIF21A	18%

KIF2A	9%
MTR	12%
NBEA	56%
NPHP3	10%
NRXN3	79%
SI	17%
SMARCA2	21%
SYK	17%
TLE4	23%
TRPA1	21%
TRPC4AP	21%
ZC3HAV1	16%
ADAMTSL1	50%
ADCY2	48%
AGFG1	8%
ANKRD20A11P	17%
ANKRD20A8P	38%
ATR	19%
BIRC6	36%
CASK	40%
CIT	24%
COL24A1	54%
COL5A3	20%
EFCAB12	8%
ENPEP	15%
EPDR1	11%
FAM118A	5%
GRK4	15%
IFT122	14%
IQCK	21%
KIF9	8%
LCP2	9%
LGR5	28%
LYRM1	5%
MCF2L2	37%
MKI67	3%
PRUNE2	40%
SDHAP1	35%
SEC16B	4%
SLC15A2	5%
UNC5D	55%
VWA8	38%
ACSM5	13%
ANKRD30BL	66%
ANKRD36BP2	19%
CADPS	52%
CDC42BPA	43%
COL4A3	29%
F5	9%
FAT3	57%
HEG1	17%
HJURP	3%
IGFN1	5%
IQGAP3	7%
KRT86	8%
NBAS	40%
NBPF22P	2%

NRAP	21%
OVCH1-AS1	21%
PLG	19%
PLXNA2	32%
PMFBP1	11%
RARA	10%
SUPT6H	5%
SZT2	4%
THSD7B	69%
TRPM6	32%
TTC3	22%
U2SURP	8%
UNC80	32%
VPS13C	24%
ZNF551	3%
ZZEF1	25%
ARHGAP6	55%
BPIFB2	5%
CARD11	31%
CCDC180	7%
CCDC7	56%
COPA	5%
CYP2A6	3%
DGKB	68%
DLG2	79%
EDEM2	4%
EGFR	27%
EML5	27%
ERI2	1%
FRY	30%
GABRA2	26%
GIGYF2	24%
GTF3C1	16%
KIF1B	23%
MAP2	31%
MUC2	15%
MYO10	47%
MYOF	30%
MYT1L	59%
NSD1	24%
PFKFB2	7%
PLAA	5%
PSME4	15%
PTPRM	62%
RCHY1	3%
RIF1	12%
SLC17A5	11%
SLIT2	42%
SPEF2	28%
SRCAP	6%
TRAPPC9	65%
ZNF98	11%
A2M	4%
AK7	20%
ARID4B	32%
BCL2L14	7%
BMS1P20	21%

BRWD3	26%
CPNE3	5%
EFCAB6	38%
GRM7	68%
IL1RAP	21%
ITK	20%
KCNT2	50%
KIAA0430	8%
LRRK2	21%
MORC4	8%
MYH9	15%
MYO18A	14%
PDZD7	7%
POLA1	43%
RASSF2	8%
RBFOX1	81%
SDHA	9%
STAT3	15%
TNC	11%
WDR64	26%
ZNF492	12%
ACTR2	9%
ACTR3B	24%
ADCY10	16%
ATP1A4	8%
BRCA1	16%
CCDC93	12%
CDK5RAP2	28%
CENPE	11%
DENND1B	32%
DNAJC18	4%
ERBB3	7%
ESRP1	15%
FANCI	16%
IPO9-AS1	21%
ITGAL	16%
MAGI3	43%
PARP1	10%
PCDHGA11	14%
PCDHGB7	13%
PDHA1	7%
POLR1A	12%
RALGAPA2	35%
ST3GAL3	38%
TLN2	30%
TMPRSS15	22%
UNC79	44%
UTP20	16%
VAPA	9%
XRN1	19%
ADAMTS20	37%
ALMS1	32%
C2CD3	22%
C3	14%
CFHR2	6%
COL6A5	18%
CPED1	39%

DOCK5	26%
DOCK8	36%
EDEM3	7%
ENPP1	22%
ERBB2	9%
EXOSC10	10%
FER1L5	7%
GSTM5	2%
H2AFV	4%
INTS4	15%
KIAA0368	12%
KIAA0922	20%
KLC4	4%
LOXHD1	30%
OVCH1	10%
PCSK5	62%
POLE	9%
PRB1	1%
SLC4A4	42%
SLC6A1	11%
SNRNP200	7%
STIL	13%
TBX20	11%
TTC4	3%
TXK	9%
ACAT2	3%
ACIN1	3%
ANKRD19P	6%
ANKRD36B	15%
AP1B1	15%
BMS1	5%
CASP1	2%
CDK11A	17%
CEACAM1	3%
CEP120	19%
CHD4	6%
CKAP5	14%
CNTN4	74%
CNTNAP2	81%
COG7	14%
COL15A1	20%
COL6A3	11%
CPS1	33%
CRAT	3%
CRLF3	9%
CTTNBP2	24%
CYP4F2	5%
DCHS2	23%
DHRSX	56%
DNAH1	9%
DOCK9	36%
DPP6	80%
DPY19L2P1	21%
DYX1C1-CCPG1	26%
EPG5	15%
EPRS	13%
EYS	76%

GCC2	16%
IL16	23%
KMT2D	3%
LPAL2	8%
MARCH1	75%
MTSS1	31%
MYH13	14%
NAP1L4	9%
NCOA3	29%
NCOR2	43%
NEK10	30%
NTRK3	50%
NUP210L	34%
NVL	21%
PIWIL1	10%
POTEG	21%
PPHLN1	16%
PRSS37	1%
RNF123	9%
SCN5A	19%
SETD2	20%
SIPA1L2	11%
SLC16A10	21%
SLC44A5	50%
SORT1	13%
SPAG5	2%
STK31	20%
SV2C	39%
TRIM37	22%
UQCRC2	6%
ABCA4	17%
ABCC1	36%
B4GALT5	21%
CCDC146	21%
CLSPN	6%
COG5	36%
DGKH	32%
DHX29	5%
DIAPH2	65%
FAM186A	16%
FARP1	47%
FBXO11	19%
FHOD3	52%
FRYL	27%
GBP2	4%
GGA3	7%
GRIP1	43%
HEPHL1	15%
IL1R2	22%
ITIH5	31%
KCNAB1	44%
LRPPRC	20%
LRRC7	52%
MAOB	19%
MROH7	15%
MYBPC1	21%
MYO16	53%

MYO18B	46%
NCAPH	9%
OR4N2	4%
OTOGL	25%
PLD5	65%
PLEKHH2	26%
PLXNA4	55%
POLR2J4	19%
PPFIBP1	28%
RALGPS2	29%
RGS7	68%
ROCK1	26%
RPS6KA2	52%
SDAD1	8%
SLC9C2	18%
SORBS3	2%
SP100	28%
SPINK5	9%
STON1-GTF2A1L	38%
STRADB	6%
SVEP1	35%
TENM2	73%
TIAM1	57%
TLK2	27%
TNNI3K	43%
UBR2	23%
USP43	12%
VPS53	46%
WASH2P	22%
ABCA12	20%
ACTN2	23%
AGBL1	70%
ATP13A3	15%
CREBBP	15%
ENPP2	21%
FAM135B	62%
FPGT-TNNI3K	45%
FRMPD2	25%
GAD2	28%
GCNT2	21%
GPR75-ASB3	30%
HECTD4	24%
IFI44L	9%
KIF13A	36%
KIF26B	67%
KNTC1	15%
KSR2	63%
LRCH3	21%
MAP3K1	10%
MECOM	64%
MLLT10P1	3%
MYO5C	22%
NALCN	41%
NAT10	11%
NPC1	13%
NUP188	14%
PDE1C	56%

PDZD2	62%
PGLYRP4	7%
PHKA2	22%
PIK3C2B	12%
PLD1	30%
PPFIA4	12%
PPP1R12B	32%
PTPN14	31%
PTPRZ1	30%
ROS1	24%
SDK2	44%
SGSM1	23%
SLC12A5	7%
SLC28A2	7%
SLC44A3	9%
SLCO1B7	20%
SMCHD1	28%
TLN1	3%
TMEM14B	3%
TNS4	6%
TRAF3IP3	3%
UBA6-AS1	6%
UBAP2	27%
USP8	16%
VPS13A	25%
WNK1	27%
ALK	64%
AQP7	22%
ARFGEF1	23%
ARHGAP29	15%
ATP2A2	6%
CASC1	21%
CCDC144CP	21%
CDH12	74%
CDH2	30%
CENPP	38%
CRB1	37%
CSPP1	22%
DGKG	38%
DSCAM	74%
ERAP1	6%
FBN3	27%
HCLS1	6%
HERC1	26%
JAKMIP3	25%
KRT6B	3%
LAMB1	11%
LIMCH1	40%
LRP6	27%
MAP3K15	33%
MROH5	17%
MRPS17	3%
MYH6	6%
MYH7	5%
MYO1B	26%
MYO7B	20%
NCAPD2	5%

NFKB1	17%
NIN	15%
ODF2L	5%
PCLO	40%
PPFIA1	20%
PPFIBP2	21%
PSG9	6%
RABGAP1L	62%
REV3L	18%
RIMS1	55%
ROBO1	71%
RPGR	10%
SENP6	17%
SLC5A1	12%
SLCO1B3	26%
SUN5	9%
TEX14	21%
TMTC1	37%
TNRC6B	35%
TOPBP1	9%
TTC7A	21%
UBAP2L	7%
XIRP2	45%
XPO5	5%
ZBTB8OS	5%
ZRANB3	43%
ABCB1	33%
ABCC3	15%
ACACB	32%
ADAM19	16%
AGAP11	7%
ANKRD42	10%
AP4B1-AS1	21%
ARFGEF2	20%
ASB3	18%
ATP13A4	22%
AUTS2	74%
BTNL8	15%
CACNA1G	13%
CD163L1	19%
CEP192	17%
COL4A1	33%
CYP2C19	16%
DNHD1	6%
ERCC6	13%
FNDC1	23%
FOCAD	42%
GABRB3	41%
GOLGA6A	4%
HARS	3%
HDAC1	6%
HSD17B7	7%
HSPG2	11%
IL9R	9%
LIN9	14%
LRBA	63%
MARS	9%

MCF2	20%
METTL5	5%
MORC1	34%
MPP6	15%
NBPF9	69%
NPHP1	14%
NRP2	21%
NUP98	16%
PAPPA2	51%
PDK1	9%
PFKFB1	18%
PIK3C2G	40%
PLCB4	45%
PPEF1	23%
PPM1H	38%
PRRC2C	11%
PSG7	3%
PXDNL	62%
RNF17	26%
SLC22A10	4%
SLC2A5	9%
SLC35G1	3%
ST18	41%
SYMPK	9%
TANC2	39%
TDRD10	14%
THSD7A	46%
TMEM38B	10%
TPCN2	23%
TSC2	4%
TSG101	11%
TTC21A	3%
TTC39A	9%
XPNPEP2	17%
XPR1	37%
ABCC9	26%
ADAMTS12	44%
AHNAK	15%
ALDH1L1	15%
APOB	5%
ATP2C1	25%
BPIFB4	10%
C1orf101	26%
C9	13%
CAPN9	14%
CATSPERB	23%
CCT6P3	6%
CELF2	57%
CEP350	25%
CHEK2P2	72%
CNTRL	10%
CRMP1	18%
DAAM2	21%
DAB1	74%
DMXL1	21%
DOCK10	39%
DROSHA	22%

DTX2P1-UPK3BP1-PMS2P11	33%
DZANK1	16%
FCRL2	12%
FREM2	21%
FUBP3	15%
GADL1	19%
GNPAT	6%
GRIPAP1	11%
GTF2A1L	11%
GUCY2C	15%
HSD17B4	10%
HSPD1	3%
IFT172	9%
INO80	20%
INPP5D	28%
ITGAX	9%
ITGB1	9%
KANSL1L	27%
KCNMA1	64%
KCNQ3	55%
LAMB4	23%
LCT	9%
LIG1	7%
LMAN1L	3%
MASP1	15%
MTOR	16%
MYOM3	12%
NINL	22%
NLK	15%
NPEPPS	12%
NSMAF	13%
NSRP1	9%
NUP205	17%
PAM	23%
PCDH11X	68%
PLCH1	42%
POTEC	11%
PPP3CC	15%
PRIM2	44%
PRKCG	9%
PSMA5	1%
PTEN	11%
PTPRF	11%
PZP	6%
RIMBP2	38%
RRN3P1	4%
SCN8A	19%
SCN9A	26%
SCYL2	16%
SLC9C1	31%
SMARCC1	27%
SMC6	10%
SORBS2	50%
SPAG17	38%
SPTBN1	29%
STRC	3%
TBC1D19	26%

TENM4	61%
TMC5	26%
TNIK	41%
TRPM4	21%
TSPAN8	6%
TVP23C	10%
USH1C	9%
WDR70	53%
ZFHX4	29%
ZNF28	6%
ZNF713	10%
ADAM28	12%
ADH7	3%
ANKRD28	25%
AOX1	10%
AQP7P1	27%
ARNT	15%
ATAD3B	3%
ATG7	38%
ATP10B	39%
CAPN13	18%
CCDC155	6%
CENPN	3%
CHD8	8%
CLIP1	20%
CNTN6	50%
DIP2C	66%
DRG1	9%
FAT1	16%
HDX	25%
ITGA7	4%
KCNH1	50%
KDM3A	9%
KIAA0196	5%
KIAA0319	21%
LMO7	35%
LRRC69	16%
MATN2	32%
MCTP2	32%
MED24	3%
MYH8	5%
NAA60	14%
NAV1	27%
NAV3	46%
NBEAP1	20%
NEDD4L	42%
NEO1	24%
NKAIN3	62%
NUP214	15%
NUP85	10%
OC90	15%
PASD1	29%
PIKFYVE	7%
PRKCQ	32%
PTPRG	58%
RANBP17	44%
REPS2	37%

RFX4	18%
SCN2A	30%
SH3KBP1	46%
SLC12A8	25%
SLC26A9	8%
SLCO1B1	15%
SMC1B	13%
SORCS2	71%
SORL1	20%
STAT4	15%
SULF2	21%
TAOK1	27%
TDRD9	24%
TEK	23%
THADA	45%
TM6SF2	6%
TMEM106B	6%
TMEM8B	4%
TRDN	51%
TVP23C-CDRT4	30%
VAV3	38%
VWA3B	38%
WWP1	24%
ZCCHC10	3%
ZNFX1	6%
ACSM2B	17%
ADAM23	23%
APBB2	48%
ATP13A5	21%
BBS9	53%
BPI	10%
C5	15%
CACNA2D4	26%
COL28A1	21%
COL4A4	32%
CYP2A7	4%
DDX60L	14%
DPY19L1	13%
DYNC1I1	43%
ECM1	8%
ELMO1	62%
EML1	24%
ESPNP	13%
GBAP1	6%
GPA33	9%
GUCY2F	19%
HEPH	19%
HERC3	13%
HOOK3	23%
KIF4A	23%
KRT6C	3%
LARS	15%
LRRC37A3	14%
MADD	6%
MAGEC3	25%
MAGI2	74%
MAP2K4	18%

MARK4	14%
MBTPS2	9%
MCM3AP	5%
MYH10	12%
NLRC5	15%
NUP155	15%
NXPE2	5%
OSBPL3	26%
P2RX7	15%
P4HA3	9%
PHEX	27%
PIP5K1A	14%
PRG4	2%
PRKAR2A	16%
SETBP1	43%
SLC24A4	36%
SLC26A7	35%
SPRN	1%
SRGAP1	36%
STAMBPL1	9%
STARD9	27%
TMEM67	13%
TRPM1	21%
TRPM8	18%
TXLNG	10%
USP42	15%
WDR43	9%
WRN	15%
YEATS2	19%
ZFPM2	68%
ZFYVE26	6%
ZIM2	17%
ZKSCAN7	4%
ZNF285	8%
ABCA1	24%
ABCA10	10%
ABCB5	29%
ABLIM3	17%
ACPP	9%
ANLN	9%
ARHGEF10	41%
ATP11A	50%
ATP6V1C1	9%
ATP9A	30%
AWAT2	3%
BRD9	2%
BRWD1	21%
CA5A	17%
CALN1	73%
CHD2	19%
CHD7	19%
CLSTN3	6%
COX10	14%
CUL9	9%
CWC22	7%
DHRS2	5%
DNMT1	15%

DZIP1L	12%
EHMT1	40%
EP300	23%
EPB42	3%
FAM13A	29%
FAM86C2P	8%
FSTL1	9%
G6PC	3%
GLB1L3	15%
GRHL3	9%
HCAR1	3%
HECTD1	19%
HELZ	25%
HSPE1-MOB4	10%
INMT-FAM188B	16%
ITCH	27%
KIAA1549L	24%
KLF7	10%
KTN1	11%
L3MBTL3	19%
LAMC1	14%
LGR6	35%
LIAS	4%
LRRC37B	9%
MAPKAPK3	5%
MCM7	4%
MLLT3	35%
MRPL3	7%
NAA25	15%
NBEAL1	29%
NF2	20%
NPHS1	13%
NRXN1	68%
P2RY12	11%
POLN	26%
POTEKP	7%
PTPRE	27%
PTPRS	26%
RAPGEF6	21%
RB1CC1	9%
RFX7	20%
RGS22	27%
RNF212	12%
SCN3A	21%
SGIP1	33%
SLC17A8	18%
SLC2A3	5%
SLC4A7	16%
SMYD3	74%
SPAG8	3%
TBC1D31	12%
TBC1D3P2	12%
TMEM254	4%
TMEM87B	15%
TNPO2	6%
TRIM51	3%
TRPM2	38%

TRPM3	62%
TSEN2	9%
TYW1	37%
USP7	11%
WHSC1L1	13%
XDH	15%
YWHAE	12%
ZCWPW1	6%
ZFC3H1	9%
ZMYM4	26%
ZNF432	5%
ZNF512B	9%
ZNF577	3%
ZNF814	6%
ABCB6	3%
ABR	53%
ADCY8	48%
ADPGK	6%
ALG1L2	13%
ANKAR	17%
ANKHD1	21%
AOAH	38%
AP4B1	5%
ARPP21	25%
ATP5J2-PTCD1	10%
ATP6V1D	3%
BBS5	3%
BET1L	1%
CAD	3%
CADPS2	62%
CALR3	5%
CAMK2A	8%
CATSPERG	7%
CDH18	72%
CEP170P1	7%
CGA	3%
COL16A1	9%
COL9A1	15%
CPAMD8	29%
CTNND1	10%
DHX9	7%
DRGX	5%
EIF3I	4%
ERBB4	68%
FAM91A1	3%
FER1L6-AS1	9%
GRIN2A	50%
HGSNAT	13%
IL1RL2	7%
IPMK	19%
ITGA2	7%
KLHL2	19%
MACROD2	81%
METTL2B	11%
MPDZ	21%
MUC12	21%
MYH2	6%

MYO15A	14%
NLRP3	12%
OTOF	15%
PARP11	5%
PIGB	5%
PLEKHA3	4%
PM20D1	4%
PMS2P5	22%
PNLDC1	6%
PPL	15%
PPOX	2%
PRRC2B	11%
PTCD1	7%
PTPRJ	25%
RAD51B	63%
RBM6	26%
RFC1	10%
RMDN2-AS1	20%
SIRPB1	3%
SMARCA1	15%
SNX29P1	4%
SORBS1	28%
SSBP3	26%
STAG1	51%
STXBP1	12%
STXBP3	20%
STYK1	10%
TBC1D23	14%
TMEM234	4%
TRAK2	9%
TRIM14	5%
TXNL4B	3%
VDAC3	2%
WDPCP	43%
WDR17	23%
XKR4	63%
ZNF540	13%
ZNF83	25%
ADA	6%
ADAMTS9	20%
AIF1L	4%
ANKRD17	19%
AP4M1	4%
ARHGEF12	18%
ARHGEF28	30%
ARMC4	24%
ATG2B	9%
ATP2B2	24%
ATP2B4	21%
BAZ1A	17%
C11orf65	15%
C16orf62	22%
CAPS2	16%
CD53	8%
CFTR	29%
CGB1	4%
CLCN1	15%

CLCNKA	5%
CLEC18B	6%
CMYA5	9%
COL17A1	15%
COL21A1	48%
CRLS1	5%
DOPEY1	15%
EI24	4%
ELAVL4	26%
EPT1	12%
FAN1	9%
FAT2	9%
FLNC	7%
GCOM1	19%
GLE1	6%
GLG1	28%
GREB1L	46%
GRIK2	62%
HECW2	38%
HSF2	2%
IFI16	5%
IFT140	13%
IKBKAP	9%
INPP4A	19%
KIAA0586	15%
KIF14	17%
KIF5B	9%
KPNB1	5%
MAP4K4	26%
MARCO	12%
MAST4	54%
MEI1	24%
NCL	4%
NLRP5	19%
NRCAM	41%
NRXN2	25%
PARP4	18%
PDE8A	24%
POLR2B	7%
POLR2M	1%
PRMT8	33%
PTPRT	78%
RB1	25%
ROBO2	63%
SAMSN1	20%
SCEL	14%
SENP2	12%
SEPT10	11%
SERPINB11	3%
SIGLEC6	6%
SLC26A4	14%
SLC37A3	15%
SLC9A9	53%
SLK	9%
SNORA63	3%
SPG11	12%
SSPO	13%

SUCO	8%
SVILP1	19%
TACC1	18%
TARS2	12%
TBCK	26%
TGFBI	8%
TMEM131	24%
TNKS1BP1	1%
TNN	21%
TOP2A	4%
TP53BP2	14%
TRAPPC10	14%
UBN1	3%
UGGT2	26%
USP13	29%
UST	48%
VWDE	15%
WDR41	7%
WDR44	22%
WWP2	29%
ZDHHC9	8%
ABCC4	50%
ABCC5	16%
ACOT11	21%
ACTR3C	18%
ADAMTS16	35%
ADCY5	29%
ALG1	4%
ALG13	11%
ANXA13	15%
ARHGEF33	9%
ASCC3	36%
ATP1A2	7%
C1orf168	16%
CACNA1H	18%
CATSPERD	19%
CCZ1B	11%
CDH17	20%
CEP89	25%
CHD3	3%
CLN3	9%
CTAGE5	26%
CUL3	15%
CYFIP2	20%
DENND4B	3%
DHX57	14%
DMXL2	20%
DOPEY2	32%
EGFLAM	27%
ERMP1	8%
EVA1C	28%
EXOSC3	1%
FAM153B	21%
FNDC3B	48%
FOLH1B	9%
FRMPD4	64%
GOLGA2	3%

GOLGA8A	14%
GSDMC	5%
HAVCR1	13%
HEATR5A	17%
HSPE1	1%
IL18R1	7%
INTS8	10%
ITGA8	36%
ITGA9	38%
ITSN1	37%
KDR	5%
LAMA5	13%
LIPA	7%
LRRIQ1	33%
LTBP1	50%
MAP3K4	12%
MED23	4%
MEP1A	9%
MTBP	9%
MYH14	15%
MYO9A	38%
NOS1	30%
NRG2	26%
OSBPL9	21%
PDE4D	74%
PHF20L1	9%
PREX1	26%
PTK7	13%
PYGB	9%
RAB31	28%
RAD18	15%
RALGPS1	39%
RBBP4	5%
RGS9	21%
RMDN2	24%
RSF1	19%
SCN11A	16%
SEMA3D	22%
SENP7	28%
SF3B1	4%
SKIV2L2	15%
SLC12A2	20%
SLC4A2	3%
SND1	40%
SORCS3	65%
SRRM2	4%
SYN3	60%
TACC2	38%
TANC1	26%
TBC1D8	22%
TCF4	52%
THOC1	9%
TNR	53%
TRIP12	19%
TTF2	5%
TUFT1	8%
UBE3C	21%

UMODL1	18%
VAV1	23%
VAV2	37%
ZBTB41	12%
ZNF638	13%
ZNF670	9%
ZNF813	6%
ACAD11	15%
ACSL6	10%
ADAM15	1%
ADAM17	12%
ADGB	34%
AGBL5	4%
AMOT	13%
ANKRD44	38%
ANPEP	6%
APPL2	12%
ARAP1	8%
ARHGEF39	3%
ARHGEF7	23%
ASTN2	73%
ATP11C	19%
ATP2A1	9%
ATP8B4	27%
ATRN	26%
ATXN2	21%
BLM	20%
C10orf76	23%
C15orf38-AP3S2	15%
CACHD1	28%
CD86	9%
CDKL4	12%
CHD9	30%
CIRBP	1%
CIRBP-AS1	2%
CLDN11	8%
CLSTN2	55%
CNKSR2	39%
CNTNAP3	32%
CSNK2A2	7%
CTNNA3	78%
CXorf65	3%
CYP11B1	4%
CYP4A22	3%
DIP2A	26%
EEA1	26%
EGF	13%
EIF4G3	39%
EML6	31%
ENOSF1	6%
ENPP3	21%
ENTPD1-AS1	37%
EPB41L2	33%
EYA1	26%
FADS2	8%
FAM188B	12%
FAM208A	9%

FAM86C1	10%
FNTB	12%
GK	21%
GRIA1	44%
HDAC3	5%
HDAC4	45%
HIVEP1	18%
IKBKB	10%
IL12RB1	21%
IL7	15%
IQCH	29%
ITIH2	13%
ITPA	6%
KCNMB3	5%
KIAA2022	31%
KIRREL	19%
KLRC2	3%
LRIG1	21%
LRRC59	7%
MAP3K5	35%
OPRM1	37%
OSBPL10	44%
PAICS	7%
PDE10A	49%
PGM1	8%
PIP4K2A	32%
PKIG	15%
PLCG2	24%
PPFIA2	49%
PPP6R3	24%
PRDM7	6%
PRPF40B	5%
PSG2	8%
PTPRH	10%
PYGL	8%
RAB27A	18%
RBAK-RBAKDN	5%
RBM18	4%
RBMX	5%
RGS6	50%
RPRD1B	9%
SIDT1	16%
SLC2A9	27%
SNTG1	73%
SPATA13	31%
SPRR2B	3%
SPTLC1	13%
SYTL5	21%
TAF4B	25%
TBC1D32	25%
TFIP11	3%
TLE1	15%
TMEM63A	10%
TOP3A	9%
TTC7B	27%
UNC13B	27%
VPS41	20%

WBP11	2%
WFDC10B	3%
XRRA1	12%
ZFP64	24%
ZFP91-CNTF	12%
ZMYM3	3%
ZP4	3%
ABCA5	8%
ABCC2	21%
ACAT1	8%
ADAMTS17	43%
ADAMTS6	38%
ADCYAP1R1	10%
ADH1C	5%
ADSL	4%
AGL	12%
ALDH1A3	4%
ALDH8A1	4%
ANKRD11	41%
AP3D1	20%
ARFGAP3	8%
ARID1A	13%
ARPC4-TTLL3	3%
ATAD5	10%
ATP10D	15%
ATP6V0A4	29%
BCR	24%
BRDT	16%
BTBD16	6%
C19orf24	3%
C2orf61	14%
C7	12%
CCDC150	15%
CD96	21%
CDK11B	31%
CEP250	8%
CNTN1	53%
COL23A1	59%
COL9A3	8%
CPVL	24%
CSTF3	6%
CYFIP1	19%
CYP24A1	7%
DDB1	5%
DENND3	10%
DOCK7	22%
ERMAP	3%
ETV6	34%
EVC2	26%
EXT2	26%
FAM66B	5%
FAT4	24%
FBXO18	7%
FCRL3	4%
FTX	38%
GK5	14%
GOLGA3	18%

GRHL2	36%
GRIA2	23%
GRID1	67%
GZMA	8%
HBP1	9%
HERC2P3	20%
HSD17B7P2	9%
IL3RA	21%
IPO5	12%
IPO7	15%
ITGAM	23%
KAT2B	13%
KIAA0556	30%
KMT2E	18%
LHCGR	16%
MAP2K5	31%
MAP7D2	22%
MC2R	8%
MCM3	3%
MICAL2	15%
MMS22L	17%
MTRR	6%
MUC21	1%
MXD1	5%
MYO1H	16%
NCKAP1L	11%
NEDD4	21%
NPL	7%
NRL	2%
NSUN2	7%
NUMA1	14%
NUP107	14%
NXF3	3%
OCRL	8%
PCMTD1	16%
PDE3A	40%
PEAR1	5%
PIWIL3	14%
PLCD4	6%
PLEKHA5	25%
PLGLA	7%
PMS2CL	6%
POGZ	10%
POLB	5%
POLR2A	3%
POTEM	16%
PRKAR1A	24%
PTCD3	5%
QRICH2	9%
RASA2	15%
REV1	12%
RICTOR	20%
ROCK1P1	21%
RPGRIP1	18%
RPL28	1%
RPL9	1%
SCLT1	26%

SCN1A	22%
SCUBE2	10%
SDCCAG8	40%
SELP	11%
SETDB1	9%
SHANK1	19%
SIPA1L1	22%
SLC4A1	3%
SLC7A7	10%
SMARCAL1	9%
SMG6	41%
SNORA11	1%
SNRPF	1%
SNTG2	64%
SNX29	56%
SP140L	15%
SPAG9	26%
SPATA18	9%
STRN4	4%
SUPT5H	6%
SWAP70	15%
SYN1	10%
TAPBPL	3%
TBC1D4	27%
TET3	23%
TMEM117	52%
TMEM238	1%
TMEM63C	15%
TNPO1	19%
TRAPPC8	37%
TRHDE	50%
TRMT1L	3%
UBE2Q1	5%
UPF2	18%
VCAN	17%
XPNPEP1	10%
XXYLT1	37%
ZCCHC11	22%
ZNF235	5%
ABCC8	9%
ADAM9	21%
AKT1	6%
ALDH1L2	14%
ANKRD27	23%
ANKS1B	76%
AQR	13%
ARHGAP12	23%
ARHGAP21	19%
ASMTL	27%
ASNS	8%
ATG16L1	6%
ATP1A1	7%
BTN3A3	3%
C20orf194	29%
C2CD5	15%
C2orf15	3%
CACNB1	3%

CARD16	2%
CAST	9%
CCDC144NL	22%
CCDC148	32%
CDC73	21%
CDH8	58%
CDHR2	16%
CDRT1	11%
CGN	4%
CHMP3	13%
CHRNB4	6%
CHURC1-FNTB	20%
CIITA	10%
CLASP1	38%
CMTR1	6%
CNGB1	17%
COG6	11%
COL25A1	56%
COL2A1	1%
COLGALT2	17%
CR2	4%
CSE1L	15%
DAAM1	28%
DAZL	4%
DDX42	11%
DEPDC1	7%
DHX36	9%
DLG1	30%
DNA2	15%
DPYS	21%
DUOX2	3%
EIF2AK2	14%
EIF5B	8%
ELP2	11%
FAM3D	11%
FAP	15%
FCHSD1	2%
FLT3	19%
FNBP4	15%
FRRS1	12%
GABRQ	2%
GALNTL6	78%
GANAB	9%
GBA2	2%
GDPD4	10%
GNPTAB	21%
GOLGA4	15%
GPLD1	14%
GSAP	16%
HEATR1	6%
HFM1	27%
HRG	5%
IGSF10	5%
IL33	10%
IL7R	5%
ITGAV	15%
ITSN2	21%

KDM3B	23%
KIDINS220	12%
KIF16B	40%
KIF20B	6%
KIF5A	9%
KIF6	45%
KSR1	17%
LINC00221	6%
MAP2K1	25%
MAST1	9%
MCM10	10%
MED1	11%
MLH3	6%
MOV10L1	19%
MTPAP	14%
MUC6	3%
MUTYH	3%
MYO9B	25%
MYZAP	9%
NAALADL2	69%
NCF2	7%
NELL1	75%
NEMF	10%
NME8	22%
NOL8	5%
NUP160	14%
PAH	31%
PAX3	16%
PCDHB16	5%
PCDHGA12	14%
PDE6C	11%
PDGFRA	15%
PDPR	15%
PHACTR3	49%
PIK3R1	12%
PIK3R6	10%
PIN4	18%
PLA2G4A	24%
PLCG1	6%
PMS2	8%
PPP2R3A	15%
PRKCB	61%
PRKG2	23%
PRSS42	1%
PSG5	6%
PSMC3IP	3%
PTPN13	31%
RANBP3L	13%
RFX2	28%
RFX6	10%
RNF111	16%
RNF185	10%
RPL5	5%
RUFY2	16%
RUNX1T1	27%
SCN4A	8%
SEH1L	13%

SEL1L3	12%
SELL	5%
SENP5	18%
SEPT6	20%
SHPRH	12%
SLC37A2	1%
SLC4A8	16%
SLC6A1-AS1	5%
SPTAN1	11%
ST3GAL6	9%
STARD3	2%
STRIP1	1%
SUSD1	26%
TAF2	15%
TBC1D9	13%
TBP	3%
TCP11L1	10%
TEC	21%
TMC2	27%
TNFRSF10C	3%
TPM3	3%
TRIM6-TRIM34	12%
USHBP1	7%
USO1	14%
USP15	17%
USP47	13%
XPC	3%
XPO1	15%
XRCC5	15%
ZBBX	32%
ZNF208	21%
ZNF701	5%
AARS	3%
ACOXL	45%
ACSM2A	10%
ADAM2	21%
AGAP1	63%
ALPK2	21%
AMPD1	7%
ANAPC4	2%
ANKHD1-EIF4EBP3	21%
ANO2	54%
APOLD1	14%
ARHGAP10	29%
ARHGAP9	1%
ARHGEF6	21%
BIN2	10%
BRD8	6%
BTN3A2	5%
BTNL3	3%
CASQ1	3%
CCAR1	12%
CCDC170	23%
CDH9	34%
CENPI	10%
CHEK2	7%
CLASP2	24%

CNDP2	7%
CNTN2	9%
CNTNAP5	71%
CPNE5	21%
CTAGE11P	4%
CTNNBL1	23%
CUL4B	11%
CYP2B6	8%
DDX52	9%
DENND5B	38%
DISC1	55%
DPYSL5	20%
EBF1	50%
EDA	48%
EDC4	2%
EFHC1	12%
EFTUD2	5%
EIF4A3	4%
EML4	24%
EPHX1	9%
EPHX2	4%
ERAP2	8%
ERC1	62%
ESF1	5%
FAM212B	9%
FAM49B	23%
FAM86JP	3%
FBXW10	9%
FCRL5	6%
FMO1	6%
FOXK2	21%
FOXP1	56%
FRMD4A	62%
FTSJ3	2%
GABBR2	53%
GATS	12%
GHITM	3%
GHR	35%
GIF	5%
GNG12-AS1	45%
GRAMD1C	18%
GRID2	75%
GRIN3A	35%
GSDMB	7%
HM13	10%
HRC	1%
INPP5B	15%
IQSEC1	20%
ITGA4	10%
KATNAL2	18%
KMO	15%
KRT6A	3%
LAMC2	14%
LRMP	18%
LRRC28	21%
LTBP4	5%
MANBA	26%

MAP4K1	9%
MARCH6	10%
MCPH1	29%
MICU3	15%
MIR663B	4%
MUSK	19%
MYH7B	5%
NCKAP5	61%
NCOA2	35%
NECAB1	37%
NHLRC2	4%
NOP14-AS1	4%
NPHP4	14%
NPLOC4	22%
NTN4	17%
NUDT13	6%
OTUD7A	50%
PAPPA	30%
PBRM1	20%
PDCD6IP	11%
PDE11A	56%
PFKP	23%
PHC2	13%
PHC3	15%
PLCE1	32%
PLCZ1	11%
POLA2	12%
PSG1	8%
PSG10P	10%
PSG3	7%
PSMD1	12%
PTPN22	12%
PTPRA	27%
PTPRO	36%
RAB37	26%
RACGAP1	9%
RALGAPA1	22%
RASGRF2	36%
RNPEP	6%
ROR1	51%
SCO1	2%
SEMA4D	14%
SETX	18%
SH3D19	15%
SLC10A7	27%
SLC24A5	4%
SLC25A12	25%
SLC30A5	10%
SLC39A11	50%
SLC4A5	23%
SLC5A9	3%
SLC6A5	16%
SLIT3	61%
SMARCA5	2%
SMC2	4%
SMG5	7%
SNX1	9%

SNX25	20%
SP110	6%
SRBD1	26%
STK32A	25%
SUN1	11%
SV2B	32%
SYCP1	21%
TCP11	8%
TDG	3%
TF	8%
THSD4	56%
TNFRSF10A	6%
TP53BP1	16%
USP25	24%
USP39	9%
WLS	22%
XG	19%
ZC3H11A	12%
ZNF585A	6%
ABCA6	10%
ABCF3	3%
ACSS2	21%
AGBL2	21%
AKT3	32%
ALDH18A1	6%
ANKRD26	21%
ANO5	21%
ANP32A	9%
ARAP3	6%
ARHGAP30	16%
ARHGEF2	8%
ARMC3	21%
ATP7B	9%
BCAS3	58%
BCRP3	12%
BIVM-ERCC5	11%
BMPER	29%
BTN2A1	3%
C14orf178	4%
C19orf44	6%
C5orf58	3%
C9orf3	38%
CAMK2G	10%
CAPN3	9%
CAPRIN2	8%
CARS2	14%
CCDC158	10%
CCDC30	21%
CCDC88A	24%
CD72	2%
CDC42BPB	16%
CELSR1	32%
CHKA	13%
CLCNKB	8%
CLOCK	21%
CNOT10	21%
COLQ	12%

COPG1	10%
CPSF3	17%
CS	9%
CTCFL	4%
CTPS2	21%
CTTN	6%
CYP4Z2P	12%
DAB2	10%
DCAF11	4%
DDR2	26%
DIDO1	9%
DLC1	48%
DLD	6%
DPYD	69%
DSP	8%
DUS2	9%
DYNC1LI2	8%
ECE1	15%
EIF2AK4	15%
EPHA7	22%
ESPL1	8%
EYA2	46%
FAM227B	38%
FARP2	22%
FCN1	3%
FGD4	23%
FGFR1	12%
FSIP2	8%
GABRA3	50%
GALNT2	32%
GC	8%
GFM1	8%
GGT1	23%
GLT8D2	15%
GOLIM4	15%
GPC5	81%
HEATR5B	16%
HELLS	9%
HHLA1	9%
IFT43	17%
IGF2BP1	16%
IGSF1	6%
IGSF22	3%
IK	2%
IPO9	9%
ITGA6	13%
ITIH4	7%
JAKMIP2	30%
KDM4C	51%
KIAA1468	15%
KIAA2026	12%
KMT2B	1%
KRBOX1	3%
KRTAP5-10	2%
LEPR	37%
LLGL2	11%
LPCAT1	11%

MAU2	9%
MGA	11%
MLH1	10%
MOV10	4%
MTIF2	5%
MYCBPAP	3%
MYSM1	9%
NAALAD2	10%
NLRP1	13%
OPN3	3%
OTUD4	4%
PARK2	80%
PARP14	6%
PDSS1	10%
PGAP1	12%
PGM2L1	18%
PI4K2A	8%
PKP4	20%
PMPCA	1%
POM121L9P	14%
POTEE	6%
PRDM10	15%
PRTG	23%
PSG11	3%
PTGS2	3%
PTPRK	50%
RAD54B	15%
RECQL	3%
ROPN1	2%
RRP12	9%
SCAF4	15%
SCAI	30%
SCAPER	44%
SCN7A	23%
SECISBP2L	9%
SEMA6C	2%
SH3PXD2B	20%
SIDT2	5%
SIK3	38%
SLC22A14	11%
SLC26A8	19%
SLC37A1	16%
SLC38A9	16%
SLC6A13	10%
SLCO1A2	23%
SOAT1	15%
SPTBN5	4%
SRRT	1%
STAG3L5P-PVRIG2P-PILRB	9%
STAT6	4%
STK38	13%
SYN2	35%
TAF6	9%
TEPP	3%
TIAF1	3%
TIAM2	26%
TNRC18	18%

TNS1	26%
TRERF1	32%
TTC13	11%
UBA6	20%
VCL	21%
WDR60	20%
WDR93	6%
XPNPEP3	13%
ZFYVE9	26%
ZNF236	18%
ZNF496	7%
ZNF763	10%
ABCC12	4%
ABL1	24%
ABTB2	32%
ACBD5	13%
ACTA2	5%
ADAM10	21%
AFF1	32%
AGBL3	26%
ALOXE3	4%
ALPK1	16%
ANKFN1	46%
ANKRD55	32%
ARHGAP28	15%
ARL17B	3%
ARNT2	22%
ARSB	23%
ASCC2	9%
ASMT	28%
ATF6	24%
ATP6V0A2	7%
BACH1	15%
BBS1	7%
BDH1	13%
BMP5	13%
BOC	11%
BOD1L1	8%
C4BPA	9%
CACNA1C-AS1	6%
CAGE1	19%
CAMSAP2	22%
CAPN14	14%
CAPN8	12%
CAT	2%
CCDC13	15%
CCDC151	16%
CCDC159	4%
CCDC60	38%
CCDC66	10%
CCT3	11%
CCT5	2%
CD46	8%
CD99	10%
CDH26	11%
CDR2	9%
CEP152	8%

COL1A1	2%
CP	6%
CPNE8	33%
CTNNAL1	9%
CTNND2	66%
CWH43	19%
DAPK1	30%
DCAF6	15%
DCST2	4%
DDX3X	5%
DDX4	14%
DENND2C	24%
DIAPH1	14%
DNM2	23%
DPY19L2P2	23%
DPY19L3	13%
DSE	21%
EFCAB5	21%
ELF1	9%
EPB41L4B	26%
EPHA5	47%
EPS15L1	23%
ERMARD	6%
ESYT1	1%
FAAH2	32%
FAM13C	21%
FAM86DP	4%
FAM98A	3%
FIG4	20%
FILIP1	28%
FMN1	40%
FOXP2	67%
FRMD7	12%
FSD1L	14%
GALC	9%
GBP7	9%
GFPT2	9%
GLDC	23%
GLP1R	13%
GRIA4	48%
GYPB	9%
GYS2	9%
HDAC9	69%
HEATR4	19%
HHIPL2	8%
HK1	23%
HNRNPM	9%
HRNR	4%
IGF1R	38%
IMPG2	10%
INTS1	7%
IQCG	15%
JAG1	9%
KCNT1	25%
KDM5A	23%
KDM6A	38%
KIAA0100	4%

KIAA1033	7%
KIAA1211	34%
LTBP2	13%
MAP3K14-AS1	3%
MAST3	13%
MCC	43%
MCF2L	48%
ME2	10%
MIPEP	16%
MPHOSPH9	14%
MRPL43	1%
MUC19	21%
NADSYN1	6%
NAPB	8%
NARS2	21%
NBPF8	56%
NCKAP1	15%
NEK11	38%
NFX1	13%
NID1	17%
NLGN3	9%
NLGN4X	50%
NLRC4	11%
NOS2	8%
NTRK2	47%
OPHN1	55%
PARP8	24%
PASK	7%
PEBP4	26%
PHIP	17%
PIK3C2A	17%
PITRM1	9%
PITRM1-AS1	3%
POF1B	18%
POLDIP3	4%
PPP1R9A	40%
PRRC1	3%
PTDSS1	11%
QSER1	14%
RAPGEF4	34%
RBBP6	3%
RBBP7	3%
RHCE	15%
SAMD7	8%
SEC13	5%
SEZ6L2	5%
SHQ1	11%
SHROOM2	31%
SLC1A6	16%
SLC4A1AP	4%
SLC6A11	15%
SLC9A8	10%
SLC9B1	34%
SMG9	3%
SPATA31C2	3%
SPEN	18%
SPICE1	13%

SPTB	16%
SPTBN4	23%
SRPK2	37%
SSR1	7%
ST5	32%
TBC1D1	37%
TBC1D28	3%
TENM3	55%
TMC3	5%
TMEM214	1%
TTC21B	9%
UBE4B	18%
VPS39	9%
WDR12	6%
WDR7	33%
WWC1	26%
XPOT	9%
YLPM1	14%
ZBTB49	9%
ZC3H7A	6%
ZMYND8	23%
ZNF114	9%
ZNF462	15%
ZNF587	4%
ZWILCH	15%
ABCB4	17%
ABCC11	10%
ACAN	10%
ACOX1	6%
ACSBG2	17%
ACTN1	15%
ADAM22	40%
ADCY1	26%
AHCYL2	24%
AHI1	27%
AKAP2	17%
ARHGEF26	22%
ARHGEF37	13%
ARNTL2	13%
ASAP2	22%
ATXN7L1	51%
BUB1	4%
C8orf44-SGK3	27%
CASP8	8%
CASZ1	26%
CC2D1A	9%
CCDC38	11%
CCDC73	32%
CCDC88C	26%
CCSER2	24%
CCT6B	7%
CD36	14%
CD44	10%
CDHR3	15%
CELA2B	3%
CFHR3	6%
CLCN2	3%

CLCN6	8%
CREB5	56%
CTR9	5%
CWC25	7%
DCLK2	28%
DDX1	8%
DGKD	17%
DHRS4L2	6%
DLG5	18%
DNAJC16	7%
DPYSL2	18%
DRP2	9%
DUOX1	7%
DUSP22	13%
EFCAB13	18%
ELMO2	3%
EPS15	21%
EPS8	20%
ERC2	62%
ERCC8	11%
ESYT3	5%
EXOC2	38%
EYA4	44%
FAM129C	12%
FAM134C	3%
FAM66C	13%
FAM86B3P	2%
FAM86FP	3%
FGD5	20%
FGD6	34%
FGFR2	21%
FMNL3	10%
FRMD4B	30%
GCLC	9%
GDPD2	3%
GGT3P	19%
GLYR1	14%
GMCL1	8%
GPR137B	9%
GRIK1	46%
HAPLN4	5%
HEATR6	4%
HIPK3	15%
HPR	7%
HPS4	11%
IGSF9	2%
INSR	40%
INTS10	4%
ITGAE	21%
ITGB2	5%
JAK1	20%
KCNQ5	56%
KIF1A	17%
LOXL3	3%
LRGUK	22%
LRRC37A6P	2%
LRSAM1	11%

LYN	25%
MAN2A1	26%
MED13L	36%
MEGF10	18%
MLLT10	37%
MMP16	38%
MRPS7	2%
MTHFD1	12%
MTMR8	26%
MYO1E	28%
MYO6	22%
NDST3	35%
NDUFA6-AS1	12%
NDUFAF6	4%
NEK1	21%
NEK5	12%
NFXL1	16%
NOS1AP	40%
NOX4	33%
NPR1	3%
OR4N3P	3%
ORC3	9%
PA2G4	6%
PACS1	31%
PAPOLG	9%
PARD3B	63%
PCBP3	44%
PCM1	12%
PDE8B	26%
PGC	3%
PHKB	35%
PHRF1	7%
PILRB	3%
PIPOX	4%
PKP2	18%
PLA2R1	14%
PLEKHG1	30%
POLQ	15%
POLR2J3	13%
POSTN	8%
PRAMEF4	9%
PRKD3	11%
PRPF8	5%
PSEN2	8%
PSG4	4%
PTPRG-AS1	8%
PTPRU	15%
RAB3GAP1	19%
RBM33	21%
RBM5	5%
RDH5	1%
RFWD2	33%
RGS3	18%
RMND1	13%
RRM1	8%
RTTN	22%
SDCCAG3	2%

SEC31A	13%
SEMA3A	46%
SETD5	10%
SLC18A1	8%
SLC25A14	5%
SLC5A6	3%
SLC6A6	13%
SNCAIP	22%
SOX6	46%
SPEG	7%
SPTBN2	8%
ST7	36%
STARD3NL	9%
SUGP1	9%
SYNE3	8%
SYNJ2	23%
TARBP1	8%
TECTA	15%
TEX35	1%
TGS1	11%
THBS3	1%
THBS4	16%
TLDC2	8%
TLK1	37%
TMCO3	19%
TMEM151B	1%
TMEM189	6%
TNRC6A	15%
TP53TG3C	1%
TRAF5	5%
TRIP11	14%
UBXN8	7%
UNC13D	1%
USP36	4%
VCP	4%
VPS72	2%
WDR19	19%
WDR66	21%
WDR90	1%
WNK4	3%
ZC3H14	9%
ZFP91	9%
ZMAT4	51%
ZNF117	7%
ZNF705E	3%
ABCC6	24%
ABLIM1	31%
ACSM4	3%
ADAMTS19	29%
AIFM1	5%
ALG5	9%
ALS2	13%
ANGPT1	43%
ANKRD7	7%
ANTXR2	29%
AP1G1	13%
ARHGAP17	17%

ARHGAP19-SLIT1	26%
ARHGAP24	50%
ARMC10	3%
ARMC8	16%
ARPC3	4%
ATP2B1	13%
ATP2C2	21%
BCOR	22%
BCORL1	9%
BMP1	3%
BRIP1	19%
C14orf39	9%
C6orf201	9%
CABIN1	16%
CACNB2	51%
CALD1	32%
CAPN11	24%
CASQ2	15%
CBFB	13%
CBLB	29%
CD1D	3%
CECR2	38%
CELA3B	4%
CERKL	25%
CERS2	3%
CHAT	13%
CHFR	10%
CHTOP	4%
CLMN	21%
COL18A1	38%
COL6A1	25%
COPB2	3%
CPNE1	2%
CSHL1	4%
CTCF	15%
CYP11B2	6%
CYP4F3	15%
DACH2	66%
DCAF5	9%
DDX46	15%
DECR1	7%
DENND5A	24%
DHX37	14%
DLG4	6%
DNAJC10	10%
DPP8	14%
EFEMP1	10%
EHBP1	35%
EML2	11%
EPB41L3	30%
ERG	36%
ETNPPL	7%
F13A1	35%
FAM149A	5%
FAM24A	2%
FHAD1	28%
FLT1	34%

FMNL2	36%
FRMD3	44%
FRMPD1	15%
FXR1	12%
FYB	29%
GAPVD1	27%
GNAS	8%
GOLGA6L5P	4%
GOLGA8S	4%
GPNMB	9%
GRIN2B	46%
GSTA1	4%
HELZ2	10%
HERC6	4%
IL4I1	7%
INPP5A	35%
INTS6	12%
IPO11	35%
IPO8	12%
ITGBL1	37%
ITLN1	6%
JADE3	26%
KCNIP4	81%
KCTD5	7%
KDM1B	9%
KIAA1524	4%
KIF13B	23%
KLC1	9%
KLKB1	5%
KRT83	2%
KRTAP5-4	3%
LGR4	10%
LPIN1	20%
LRRC41	8%
MAATS1	13%
MAGEC1	1%
MAP3K3	9%
MARK1	21%
MED14	11%
MEGF8	13%
MEIS2	26%
MEP1B	6%
MET	16%
MICAL1	2%
MON2	16%
MS4A14	8%
MTMR7	13%
MUC20	7%
MX2	12%
NCOA1	25%
NDUFS1	4%
NEDD9	26%
NF1P2	16%
NFKBIZ	4%
NIPA1	7%
NIT1	1%
NLRP8	10%

NOX3	15%
NR1H4	17%
NRK	10%
NT5C1B	5%
NUDCD1	10%
OAZ3	4%
OGDH	18%
OSCP1	7%
OSMR	9%
PAPLN	5%
PARP12	6%
PCCA	44%
PDIA5	13%
PER3	9%
PEX1	6%
PHLDB3	15%
PIGO	3%
PLEKHA6	21%
PLEKHA7	39%
PLEKHG2	1%
PLXDC2	56%
PLXNB2	9%
POLR3A	9%
PON1	3%
POP1	9%
PPARGC1A	11%
PPFIA3	9%
PPM1B	16%
PRDM16	64%
PROSER1	6%
PRPF40A	7%
PSAT1	10%
PTPN21	13%
PTPN3	13%
PXDN	24%
RASA3	51%
RASGEF1B	9%
RBBP5	9%
RECQL4	3%
RERE	49%
RGPD4	18%
RHOT1	14%
RMDN1	4%
ROPN1B	1%
RRP1	9%
RSRC2	8%
RTCB	4%
RXFP2	10%
SCN10A	15%
SCYL3	6%
SDHAP3	11%
SEPT7P9	7%
SERF2	5%
SERPINB7	9%
SFMBT2	48%
SHC3	37%
SIMC1	22%

SKA3	2%
SLC18A2	7%
SLC22A20	3%
SLC4A11	2%
SLC51A	3%
SLC6A12	7%
SLCO1C1	12%
SLFN12L	4%
SMARCC2	4%
SMYD5	3%
SNORD21	1%
STAB1	6%
SUPT16H	5%
TAF10	1%
TCTN2	10%
TMBIM6	6%
TMEM132D	78%
TMEM189-UBE2V1	15%
TNIP3	12%
TNNT2	3%
TNRC6C	12%
TRIM33	17%
TRO	6%
TSGA10	27%
TTLL3	3%
TULP3	15%
UBR1	26%
UGT3A1	8%
UNK	5%
URI1	15%
USP11	2%
USP20	9%
VCAM1	2%
VPS33A	9%
XPO4	19%
YME1L1	13%
ZBTB20	62%
ZCCHC17	8%
ZEB1	28%
ZMAT2	4%
ZNF385D	47%
ZNF571	7%
ZNF578	18%
AAMDC	9%
AASS	11%
ABCA7	15%
ABCD3	14%
ACAA1	1%
ACAD10	9%
ADAM7	13%
ADAMTS18	24%
AHCTF1P1	3%
AMY2B	4%
AMZ2P1	1%
ANKFY1	17%
ANO6	26%
ANXA11	7%

AP2A2	17%
APAF1	12%
APOH	7%
ARHGAP26	41%
ARHGEF10L	21%
ARL10	3%
ARMC9	23%
ASIC2	78%
ATIC	6%
ATP12A	15%
ATP8B3	8%
ATRIP	4%
BTAF1	19%
BTG4	6%
C12orf40	43%
C12orf43	4%
C16orf58	1%
C2orf42	16%
C5orf34	4%
CALCR	28%
CARD8	8%
CBS	3%
CCDC136	3%
CCDC140	1%
CCDC50	10%
CCDC69	7%
CCZ1	7%
CD109	23%
CD177	4%
CD300LD	3%
CDKL1	13%
CDYL	39%
CEP63	11%
CEP85L	38%
CFL1	3%
CGB2	3%
CHCHD3	33%
CHN2	49%
CKMT2-AS1	9%
CLEC4M	2%
CLIP4	17%
CLTA	4%
CMSS1	40%
CNTD1	4%
CNTN5	73%
CNTNAP4	38%
COL6A2	10%
CPT1C	8%
CPXM2	15%
CWF19L2	23%
DAP3	15%
DCAF17	9%
DCST1	5%
DDX27	7%
DDX31	15%
DEGS1	3%
DHX35	17%

DIAPH3	43%
DLG3	9%
DNAI1	11%
DNAJC17	7%
DNAJC6	21%
DNMT3A	21%
DOCK6	10%
DPYSL3	10%
DSCAML1	47%
DSTYK	20%
DTNB	33%
DYNC2LI1	12%
ECE2	9%
EFTUD1P1	5%
EIF2B3	24%
EIF2S1	3%
EIF4A2	4%
EIF4G1	2%
ENTPD5	13%
EPHA4	12%
ERCC5	3%
ESYT2	21%
EVC	25%
EVI5	38%
EXOC3	7%
FAM193A	13%
FAM65B	39%
FBXO38	1%
FKBP15	10%
FKBP9	13%
FLG2	1%
FNBP1	35%
FSTL4	49%
FYTTD1	12%
GABRA6	4%
GDA	35%
GGA2	6%
GGT5	7%
GIMAP5	3%
GORASP2	8%
HADHA	9%
HBS1L	11%
HELQ	8%
HERC4	15%
HHAT	47%
HLTF	6%
IGSF3	15%
IL12RB2	16%
IL20RB	7%
IL4R	6%
INPP4B	60%
INTS2	13%
ITGAD	6%
ITGB7	5%
JMJD1C	46%
KCNK2	38%
KIAA0319L	23%

KIAA0907	4%
KIFAP3	18%
KLHL3	25%
LACE1	32%
LDLR	15%
LIMS1	32%
LIPH	12%
LMF1	64%
LONRF3	16%
LRP4	12%
LRRC37A2	4%
LRRC37A4P	3%
LRRC6	20%
LSAMP	69%
LTF	11%
LTN1	14%
LY9	6%
MAN2B1	3%
MAP2K3	11%
MAP3K13	35%
MAP3K19	14%
MELK	17%
MFSD11	8%
MGAT5	21%
MORF4L1	5%
MPP7	32%
MR1	6%
MRPS22	3%
MRS2	4%
MS4A4A	10%
MS4A6E	3%
MTMR4	2%
NBN	6%
NCAN	14%
NCAPG2	13%
NELL2	50%
NFIA	43%
NLRP4	26%
NMNAT2	35%
NRG1	76%
NT5DC1	12%
OAS2	9%
OGG1	4%
OGT	9%
OSBPL6	29%
OXR1	47%
PABPC1	7%
PCBP2	6%
PCDH19	23%
PCYT1A	14%
PDE6A	13%
PDE9A	32%
PDPK1	10%
PEG3	8%
PEX5L	38%
PFKM	6%
PGBD5	27%

PHF20	33%
PHLPP2	9%
PIK3AP1	21%
PIK3C3	18%
PKD1	10%
PKD1L3	13%
PLA2G4C	17%
PLEK	7%
PLOD2	15%
PLOD3	3%
POC1B	15%
POMT2	9%
PON2	6%
PRC1	5%
PRDM5	31%
PTGER3	40%
PUS10	9%
RAPH1	15%
RBM25	14%
RBM28	7%
RCE1	1%
RECQL5	8%
RFPL1S	3%
RFTN1	29%
RNF31	4%
RPS27L	1%
RUFY1	12%
SBNO1	11%
SCIN	15%
SEC24C	3%
SEC31B	5%
SENP3-EIF4A1	5%
SGK2	3%
SH3RF1	24%
SLC26A3	9%
SLC28A1	25%
SLC43A1	10%
SLMAP	21%
SLX4	8%
SMARCAD1	15%
SMC4	9%
SNRNP27	5%
SOS2	24%
SOX5	68%
SPATA17	28%
SPATA21	6%
SPATA32	1%
SPATA6	25%
STAT1	2%
STK10	32%
STRN3	18%
SULT1A2	3%
SUMF1	17%
SUMF2	6%
TAF4	32%
TAOK3	30%
TATDN1	5%

TBC1D29	2%
TBC1D2B	11%
TEX11	56%
TFCP2	14%
TM9SF4	9%
TNFSF12	3%
TNFSF4	2%
TNPO3	21%
TOM1L1	11%
TP63	38%
TPH2	15%
TPTE2P1	15%
TRIM46	3%
TTC14	2%
TTC37	6%
TUBA1C	10%
TUBGCP4	6%
TUBGCP6	11%
UNC5B	18%
UTP14A	5%
WBSCR17	67%
WDR45B	9%
WDR88	12%
XPO7	9%
XYLB	10%
ZBED1	3%
ZCCHC6	8%
ZFHX3	38%
ZFP30	3%
ZNF106	8%
ZNF443	3%
ACAP3	4%
ACO1	9%
ACSL5	15%
ADAL	6%
ADAM11	4%
ADAM12	54%
AKAP6	50%
AKNA	5%
AKR1B10	8%
ALAS2	4%
ANO10	23%
AP2A1	9%
AP3S2	13%
AQP10	2%
AQP4-AS1	13%
ARHGAP44	33%
ARHGEF15	5%
ARHGEF3	36%
ARHGEF35	2%
ARHGEF40	6%
ARID2	24%
ASS1	15%
ASXL2	18%
ATG4A	13%
ATP6V0A1	9%
BCCIP	3%

BMX	9%
BPIFB1	9%
BRINP1	38%
BTN3A1	3%
C6	21%
CADM2	68%
CAMTA2	5%
CAPN2	15%
CC2D2A	10%
CCDC110	3%
CCDC141	34%
CD163	10%
CD300LF	9%
CD8B	10%
CENPU	6%
CEP164	5%
CEP70	13%
CHI3L2	3%
CHL1	36%
CHRD	5%
CLNK	30%
CLSTN1	15%
CNST	19%
COCH	3%
COMMD7	16%
COPB1	10%
CORIN	33%
CPNE9	8%
CRIPAK	1%
CTNNA1	28%
CUL7	2%
CWC27	27%
DAPK2	29%
DDX55	3%
DENND2A	21%
DQX1	4%
DSCC1	6%
DUSP27	5%
DYTN	21%
EBNA1BP2	3%
EMC1	5%
EPHA3	43%
ERLEC1	11%
EXO1	15%
FAM153A	20%
FAM196B	23%
FAM65C	23%
FAM81B	13%
FAM86HP	4%
FAR2	26%
FARSB	8%
FCHSD2	33%
FCRL6	3%
FER	33%
FPGS	2%
FRMD5	38%
FXYD5	5%

GALK2	24%
GALNT11	17%
GBP4	3%
GLB1	14%
GLIPR2	9%
GLP2R	14%
GLS	12%
GOLGA1	8%
GRAMD3	13%
GRB10	22%
GRIK4	50%
GUCY1A3	10%
GYG2	16%
HERC2P9	6%
HIST1H2AC	2%
HIVEP3	53%
HMGXB3	6%
HOOK1	8%
HORMAD1	4%
IBTK	15%
ICA1L	22%
IDO2	18%
IFT80	15%
IKZF3	15%
IL2RA	16%
IL5RA	9%
INSRR	4%
IQCA1	25%
IRF4	6%
ITFG2	4%
ITGA11	15%
ITGB3	11%
ITGB3BP	13%
JAK2	20%
KAT6B	22%
KCNIP1	51%
KDM4A	9%
KIAA0391	21%
KIAA0753	13%
KIF7	4%
L3MBTL4	59%
LALBA	3%
LAMB3	8%
LAMP2	18%
LAYN	6%
LDB2	60%
LGALS9	7%
LMBRD2	7%
LXN	3%
MAPK8IP3	21%
MAPKBP1	4%
MAPT	25%
MAST2	39%
MBNL3	21%
MBTD1	15%
MDM1	4%
MFN1	7%

MICAL3	33%
MIR5188	1%
MIR548N	30%
MME	21%
MORC2	8%
MPRIP	20%
MRPL9	2%
MRPS15	1%
MTFR1	13%
MTMR14	9%
MTMR3	23%
MTUS2	54%
MX1	9%
MYBPC3	4%
MYO1D	34%
NDE1	13%
NDRG2	7%
NEK9	9%
NELFCD	3%
NISCH	4%
NLRP13	14%
NNT	18%
NOC3L	4%
NOMO3	9%
NOP14	9%
NR1I3	4%
NRP1	25%
OAS3	6%
OBSL1	3%
OFD1	3%
ORC1	9%
OSBPL1A	30%
PALM2-AKAP2	40%
PARP2	1%
PARP9	7%
PCCB	14%
PCSK1	3%
PCYT1B	21%
PDAP1	3%
PDE2A	19%
PDGFD	42%
PDZRN4	54%
PFKFB4	7%
PIK3R4	12%
PIP5K1B	41%
PIWIL4	3%
PLA2G4F	1%
PLCXD1	34%
PMS2P4	9%
PNLIPRP3	10%
PNPLA6	3%
PNPO	2%
PODXL	7%
POLR2J	3%
POTEF	15%
PRB3	1%
PRKCE	50%

PROS1	23%
PRPSAP2	5%
PSG6	6%
PSIP1	5%
PSPC1	16%
PTBP2	17%
PUM1	23%
PWP2	3%
PYHIN1	14%
R3HDM2	29%
RAD9B	6%
RBM26	9%
REN	3%
RFC4	5%
RGPD1	41%
ROR2	30%
RPS6KA1	3%
RRN3P3	3%
RTN4IP1	11%
RUNX1	32%
S100A13	4%
SALL1	4%
SARS2	5%
SCP2	16%
SCUBE1	27%
SEC14L3	5%
SEC24B	18%
SEMA6A	15%
SEPT4	2%
SF3A3	13%
SHE	6%
SIPA1L3	38%
SKP2	8%
SLAMF7	4%
SLC15A1	14%
SLC22A2	11%
SLC24A2	44%
SLC5A12	10%
SMYD2	4%
SPAG1	12%
SSFA2	3%
STK11IP	5%
STK36	10%
STXBP5	19%
SULT1A1	5%
SUPT20H	8%
SWT1	16%
SYS1-DBNDD2	5%
SYTL2	18%
TBC1D15	13%
TCEA1	8%
TDRD5	16%
TDRKH	3%
TGFBR3	32%
THBS1	2%
THOC6	5%
TJP2	17%

TMEM132B	52%
TNS3	48%
TPM1	3%
TPTEP1	15%
TRIM48	9%
TRIM6	2%
TRIM67	18%
TRMT44	7%
TRPM7	25%
TSHZ2	59%
TSPAN9	42%
TUSC3	28%
UBE2F-SCLY	23%
UBE3B	5%
USP45	7%
USP53	12%
WDHD1	16%
WDR35	8%
WHSC1	20%
YY1AP1	9%
ZCCHC2	8%
ZFR	14%
ZNF280C	16%
ZNF521	45%
ZNF573	5%
ZNF618	24%
ZNF69	4%
ZNF695	22%
