# Supplementary material for: An integrative bioinformatics approach reveals coding and non-coding gene variants associated with gene expression profiles and outcome in breast cancer molecular subtypes
Source: Br J Cancer. 2018 Mar 21;118(8):1107–14. doi: 10.1038/s41416-018-0030-0 (PMC5931099; doi:10.1038/s41416-018-0030-0)
Supplement: Supplementary file 6 — Supplementary Table 5 [file 41416_2018_30_MOESM6_ESM.pdf]

Supplementary Table 5. Functional variations in non-coding regions of cancer-associated genes in breast cancer patients from the TCGA database

Chromosome	Location	Reference	Alteration	Target gene	Network	Motif analysis	Score
chr17	35347967	C	T	AATF (Intron and Promoter)	AATF:PPI(0.897)REG(0.880)		1.341
chr17	35347865	C	T	AATF (Intron and Promoter)	AATF:PPI(0.897)REG(0.880)		0.718
chr17	35347923	C	T	AATF (Intron and Promoter)	AATF:PPI(0.897)REG(0.880)		0.718
chr17	35376422	C	A	AATF (Intron)	AATF:PPI(0.897)REG(0.880)		1.341
chr17	35307508	C	G	AATF (Intron)	AATF:PPI(0.897)REG(0.880)		0.903
chr17	35348344	C	T	AATF (Intron)	AATF:PPI(0.897)REG(0.880)		0.718
chr10	27060137	C	T	ABI1 (Intron)	ABI1:PPI(0.895)		1.336
chr9	133755882	C	G	ABL1 (Intron)	ABL1:PPI(0.998)REG(0.981)		3.416
chr9	133755645	A	C	ABL1 (Intron)	ABL1:PPI(0.998)REG(0.981)		2.793
chr9	133761992	G	C	ABL1 (UTR)	ABL1:PPI(0.998)REG(0.981)		1.793
chr1	179079595	G	A	ABL2 (Intron)	ABL2:PHOS(0.775)PPI(0.831)REG(0.409)		1.203
chr1	179190233	C	G	ABL2 (Intron)	ABL2:PHOS(0.775)PPI(0.831)REG(0.409)		0.765
chr1	179079293	T	G	ABL2 (Intron)	ABL2:PHOS(0.775)PPI(0.831)REG(0.409)		0.765
chr1	179079192	G	T	ABL2 (Intron)	ABL2:PHOS(0.775)PPI(0.831)REG(0.409)		0.580
chr1	179080150	A	C	ABL2 (Intron)	ABL2:PHOS(0.775)PPI(0.831)REG(0.409)		0.580
chr1	179088024	A	C	ABL2 (Intron)	ABL2:PHOS(0.775)PPI(0.831)REG(0.409)		0.580
chr1	179102623	A	C	ABL2 (Intron)	ABL2:PHOS(0.775)PPI(0.831)REG(0.409)		0.580
chr1	179102753	A	C	ABL2 (Intron)	ABL2:PHOS(0.775)PPI(0.831)REG(0.409)		0.580
chr1	179079801	T	G	ABL2 (UTR)	ABL2:PHOS(0.775)PPI(0.831)REG(0.409)		1.765
chr1	179079801	T	G	ABL2 (UTR)	ABL2:PHOS(0.775)PPI(0.831)REG(0.409)		1.765
chr1	179079801	T	G	ABL2 (UTR)	ABL2:PHOS(0.775)PPI(0.831)REG(0.409)		1.765
chr1	179079801	T	G	ABL2 (UTR)	ABL2:PHOS(0.775)PPI(0.831)REG(0.409)		1.765
chr1	179079801	T	G	ABL2 (UTR)	ABL2:PHOS(0.775)PPI(0.831)REG(0.409)		1.765
chr19	51380015	G	A	AC037199.1 (Promoter), KLK2 (Intron)	KLK2:PPI(0.623)		0.481

chr3	38182136	C	G	ACAA1 (Medial)	ACAA1:PPI(0.307)REG(0.409),MYD88:PPI(0.831)REG(0.409)		0.765
chr17	61590824	A	T	ACE (UTR), KCNH6 (Medial)	ACE:PPI(0.307)REG(0.409),KCNH6:REG(0.409)		1.385
chr17	61590824	A	T	ACE (UTR), KCNH6 (Medial)	ACE:PPI(0.307)REG(0.409),KCNH6:REG(0.409)		1.385
chr11	125542745	C	G	ACRV1 (Intron), CHEK1 (Intron)	CHEK1:PHOS(0.990)PPI(0.957)		0.970
chr10	104262535	A	C	ACTR1A (Promoter), SUFU (Promoter)	ACTR1A:PPI(0.707)REG(0.880),SUFU:PHOS(0.798)PPI(0.863)REG(0.761)	MOTIFG=SETDB1_disc1#104262532#104262557#-#22#15879#11534,Znf143_disc3#104262534#104262553#-#18#12501#8184	3.082
chr7	6048804	G	C	AIMP2 (Promoter), PMS2 (Promoter)	AIMP2:PPI(0.774)REG(0.880),PMS2:PHOS(0.509)PPI(0.890)REG(0.880)		1.499
chr7	91671940	C	G	AKAP9 (Intron)	AKAP9:PPI(0.838)		1.401
chr7	91718574	G	A	AKAP9 (Intron)	AKAP9:PPI(0.838)		1.216
chr7	91621405	G	T	AKAP9 (Intron)	AKAP9:PPI(0.838)		1.216
chr7	91726877	C	A	AKAP9 (Intron)	AKAP9:PPI(0.838)		1.216
chr7	91739309	T	C	AKAP9 (Intron)	AKAP9:PPI(0.838)		1.200
chr7	91671646	C	G	AKAP9 (Intron)	AKAP9:PPI(0.838)		0.779
chr7	91732146	G	T	AKAP9 (Intron)	AKAP9:PPI(0.838)		0.779
chr7	91641695	G	C	AKAP9 (Intron)	AKAP9:PPI(0.838)		0.779
chr7	91670300	A	T	AKAP9 (Intron)	AKAP9:PPI(0.838)		0.593
chr7	91624113	A	C	AKAP9 (Intron)	AKAP9:PPI(0.838)		0.593
chr7	91694866	C	A	AKAP9 (Intron)	AKAP9:PPI(0.838)		0.593
chr7	91599545	A	C	AKAP9 (Intron)	AKAP9:PPI(0.838)		0.593
chr7	91661019	C	G	AKAP9 (Intron)	AKAP9:PPI(0.838)		0.593
chr7	91646442	A	C	AKAP9 (Intron)	AKAP9:PPI(0.838)		0.593
chr7	91715046	G	A	AKAP9 (Intron)	AKAP9:PPI(0.838)		0.593
chr7	91731891	C	G	AKAP9 (Intron)	AKAP9:PPI(0.838)		0.593
chr7	91625183	G	T	AKAP9 (UTR)	AKAP9:PPI(0.838)		0.779
chr14	105239723	G	T	AKT1 (Intron and Promoter)	AKT1:PHOS(0.681)PPI(0.992)		0.976
chr19	40360761	C	A	AKT2 (Distal)	AKT2:PPI(0.917)REG(0.634),LTBP4:PPI(0.408)REG(0.830)		0.951
chr19	41386282	G	C	AKT2 (Distal)	AKT2:PPI(0.917)REG(0.634),CD79A:PPI(0.707)REG(0.761),CEACAM21:REG(0.409),CYP2A7:PHOS(0.276),LTBP4:PPI(0.408)REG(0.830)	MOTIFG=CHD2_disc2#41386279#41386287#-#5#9438#8502,E2F_disc5#41386279#41386287#-#5#9743#8074	1.920

chr19	41386209	A	G	AKT2 (Distal)	AKT2:PPI(0.917)REG(0.634),CD79A:PPI(0.707)REG(0.761),CEACAM21:REG(0.409),CYP2A7:PHOS(0.276),LTBP4:PPI(0.408)REG(0.830)		0.951
chr19	41596102	G	T	AKT2 (Distal)	AKT2:PPI(0.917)REG(0.634),CD79A:PPI(0.707)REG(0.761),CEACAM21:REG(0.409),LTBP4:PPI(0.408)REG(0.830)		1.574
chr19	41354417	G	C	AKT2 (Distal)	AKT2:PPI(0.917)REG(0.634),CEACAM21:REG(0.409),LTBP4:PPI(0.408)REG(0.830)	MOTIFG=CHD2_disc2#41354414#41354422#-#5#9438#8502,E2F_disc5#41354414#41354422#-#5#9743#8074	1.920
chr1	243727150	A	G	AKT3 (Intron)	AKT3:PHOS(0.949)PPI(0.932)		1.658
chr1	243652572	G	A	AKT3 (Intron)	AKT3:PHOS(0.949)PPI(0.932),SDCCAG8:PPI(0.515)REG(0.634)		0.850
chr1	116926554	A	G	AL136376.1 (Intron), ATP1A1 (Intron)	ATP1A1:PPI(0.718)		0.588
chr12	112236049	G	A	ALDH2 (Intron)	ALDH2:PPI(0.657)REG(0.409)		1.139
chr12	112241782	G	C	ALDH2 (Intron)	ALDH2:PPI(0.657)REG(0.409)		0.953
chr12	112237627	G	T	ALDH2 (Intron)	ALDH2:PPI(0.657)REG(0.409)		0.516
chr16	30077131	C	T	ALDOA (Promoter and UTR), PPP4C (Medial)	ALDOA:PHOS(0.276)PPI(0.753)REG(0.952),PPP4C:PHOS(0.651)PPI(0.880)		3.278
chr16	30077131	C	T	ALDOA (Promoter and UTR), PPP4C (Medial)	ALDOA:PHOS(0.276)PPI(0.753)REG(0.952),PPP4C:PHOS(0.651)PPI(0.880)		3.278
chr2	29445378	C	T	ALK (Intron)	ALK:PPI(0.761)REG(0.409)		1.271
chr2	29917567	C	A	ALK (Intron)	ALK:PPI(0.761)REG(0.409)		0.648
chr2	29446092	T	G	ALK (Intron)	ALK:PPI(0.761)REG(0.409)		0.648
chr2	29445031	C	G	ALK (Intron)	ALK:PPI(0.761)REG(0.409)		0.648
chr2	29445061	C	T	ALK (Intron)	ALK:PPI(0.761)REG(0.409)		0.648
chr2	29497862	C	T	ALK (Intron)	ALK:PPI(0.761)REG(0.409)		0.463
chr2	29474222	C	G	ALK (Intron)	ALK:PPI(0.761)REG(0.409)		0.463
chr2	29432776	T	C	ALK (Intron)	ALK:PPI(0.761)REG(0.409)		0.463
chr2	29498378	G	T	ALK (Intron)	ALK:PPI(0.761)REG(0.409)		0.463
chr2	29755090	G	A	ALK (Intron)	ALK:PPI(0.761)REG(0.409)		0.463
chr14	78170475	T	C	ALKBH1 (Intron)	ALKBH1:PHOS(0.509)REG(0.409)		0.390
chr11	43905530	C	G	ALKBH3 (Intron)	ALKBH3:REG(0.409)		0.956
chr8	6377330	A	T	ANGPT2 (Intron), MCPH1 (Intron)	ANGPT2:PPI(0.408)REG(0.409)		0.333
chr8	6384967	C	A	ANGPT2 (Intron), MCPH1 (Intron)	ANGPT2:PPI(0.408)REG(0.409)		0.148

chr8	6377579	G	C	ANGPT2 (Intron), MCPH1 (Intron)	ANGPT2:PPI(0.408)REG(0.409)		0.148
chr8	6378691	A	G	ANGPT2 (Intron), MCPH1 (Intron)	ANGPT2:PPI(0.408)REG(0.409)		0.148
chr1	11253857	G	T	ANGPTL7 (Intron), MTOR (Intron)	MTOR:PPI(0.718)REG(0.909)		0.747
chr19	17396056	G	A	ANKLE1 (Intron)	USHBP1:PPI(0.841)		0.599
chr19	4182580	C	T	ANKRD24 (Promoter), SIRT6 (Promoter and UTR)	ANKRD24:PPI(0.482),SIRT6:PHOS(0.276)PPI(0.482)REG(0.988)	MOTIFBR=TAF1#TATA_disc8_8mer#4182578#4182588#-#8#0085661#0178771,TBP#TATA_disc8_8mer#4182578#4182588#-#8#0085661#0178771,ELK4#Ets_known5_8mer#4182578#4182589#-#9#0155556#0609091,ETS1#Ets_known5_8mer#4182578#4182589#-#9#0155556#0609091,GABPA#Ets_known5_8mer#4182578#4182589#-#9#0155556#0609091,ELF1#ELF1_disc2_8mer#4182580#4182590#-#10#0067771#0593374	2.744
chr11	94225920	C	T	ANKRD49 (Promoter), MRE11A (UTR)	MRE11A:PHOS(0.693)PPI(0.970)		1.095
chr11	112050239	C	A	AP002884.3 (Intron), BCO2 (Intron), SDHD (Intron)	BCO2:REG(0.409),SDHD:PPI(0.448)REG(0.409)		0.791
chr11	112049794	G	T	AP002884.3 (Intron), BCO2 (Intron), SDHD (Intron)	BCO2:REG(0.409),SDHD:PPI(0.448)REG(0.409)		0.353
chr11	112051522	C	T	AP002884.3 (Intron), BCO2 (Intron), SDHD (Intron)	BCO2:REG(0.409),SDHD:PPI(0.448)REG(0.409)		0.353
chr11	112039960	G	T	AP002884.3 (Promoter), BCO2 (Medial)	BCO2:REG(0.409),SDHD:PPI(0.448)REG(0.409)		0.353
chr22	29747040	A	T	AP1B1 (Intron)	AP1B1:PPI(0.846)REG(0.409)		1.232
chr22	29735502	C	A	AP1B1 (Intron)	AP1B1:PPI(0.846)REG(0.409)		0.794
chr22	29750633	C	G	AP1B1 (Intron)	AP1B1:PPI(0.846)REG(0.409)		0.609
chr22	29747550	C	T	AP1B1 (Intron)	AP1B1:PPI(0.846)REG(0.409)		0.609
chr15	83350076	C	G	AP3B2 (Intron)	AP3B2:PPI(0.707)REG(0.409)		0.574

chr15	83345476	C	T	AP3B2 (Intron)	AP3B2:PPI(0.707)REG(0.409)		0.389
chr15	83335316	G	C	AP3B2 (Intron)	AP3B2:PPI(0.707)REG(0.409)		0.389
chr5	112113405	G	A	APC (Intron)	APC:PPI(0.975)REG(0.761)		1.109
chr5	112111452	A	G	APC (Intron)	APC:PPI(0.975)REG(0.761)		0.924
chr5	112173111	C	T	APC (Intron)	APC:PPI(0.975)REG(0.761)		1.547
chr14	20925081	C	A	APEX1 (Intron)	APEX1:PPI(0.890)REG(0.929),OSGEP:PPI(0.781) REG(0.929)		0.982
chrX	55028154	C	A	APEX2 (Intron)	APEX2:PPI(0.707)		0.389
chrX	55032702	T	A	APEX2 (Intron)	APEX2:PPI(0.707)		0.389
chr2	68756151	A	G	APLF (Intron)	APLF:PPI(0.142)REG(0.761)		0.648
chr2	68740841	T	G	APLF (Intron)	APLF:PPI(0.142)REG(0.761)		0.463
chr17	63532982	T	C	APOH (Distal)	APOH:PHOS(0.398)PPI(0.657)REG(0.880),AXIN2 :PPI(0.761)REG(0.880)		2.488
chr17	63532940	G	A	APOH (Distal)	APOH:PHOS(0.398)PPI(0.657)REG(0.880),AXIN2 :PPI(0.761)REG(0.880)		1.865
chr9	32984621	T	A	APTX (Intron)	APTX:PHOS(0.276)PPI(0.695)REG(0.761)		0.648
chr9	33019622	C	A	APTX (Intron)	APTX:PHOS(0.276)PPI(0.695)REG(0.761)		0.648
chr9	32986195	C	A	APTX (Intron)	APTX:PHOS(0.276)PPI(0.695)REG(0.761)		0.463
chrX	66906074	A	C	AR (Intron)	AR:PPI(0.991)		1.973
chrX	66906074	A	C	AR (Intron)	AR:PPI(0.991)		1.973
chrX	66906074	A	C	AR (Intron)	AR:PPI(0.991)		1.973
chrX	66906074	A	C	AR (Intron)	AR:PPI(0.991)		1.973
chrX	66943460	G	T	AR (Intron)	AR:PPI(0.991)		1.781
chrX	66943683	A	T	AR (UTR)	AR:PPI(0.991)		1.596
chrX	47422467	G	A	ARAF (Intron)	ARAF:PHOS(0.997)PPI(0.814)REG(0.634)		1.800
chr11	120347924	G	C	ARHGEF12 (Intron)	ARHGEF12:PPI(0.641)		0.937
chr11	120315996	G	T	ARHGEF12 (Intron)	ARHGEF12:PPI(0.641)		0.937
chr11	120278605	G	A	ARHGEF12 (Intron)	ARHGEF12:PPI(0.641)		0.937
chr11	120346289	C	G	ARHGEF12 (Intron)	ARHGEF12:PPI(0.641)		0.499
chr11	120347910	C	A	ARHGEF12 (Intron)	ARHGEF12:PPI(0.641)		0.314
chr11	120317243	C	A	ARHGEF12 (Intron)	ARHGEF12:PPI(0.641)		0.314
chr11	120348148	C	T	ARHGEF12 (Intron)	ARHGEF12:PPI(0.641)		0.314
chr11	120278332	G	A	ARHGEF12 (Intron)	ARHGEF12:PPI(0.641)		0.314
chr1	27097556	G	A	ARID1A (Intron and Promoter)	ARID1A:PPI(0.869)REG(0.830)		1.453
chr1	27099478	G	A	ARID1A (Intron)	ARID1A:PPI(0.869)REG(0.830)		2.076

chr1	27094278	C	G	ARID1A (Intron)	ARID1A:PPI(0.869)REG(0.830)		1.279
chr1	27099757	G	A	ARID1A (Intron)	ARID1A:PPI(0.869)REG(0.830)		0.656
chr6	157109186	C	G	ARID1B (Intron)	ARID1B:PPI(0.739)REG(0.409)		0.616
chr6	157507481	C	A	ARID1B (UTR)	ARID1B:PPI(0.739)REG(0.409)		0.616
chr12	46230199	T	C	ARID2 (Intron and Promoter)	ARID2:PPI(0.515)		0.209
chr12	46242753	G	A	ARID2 (Intron)	ARID2:PPI(0.515)		2.017
chr12	46242804	C	A	ARID2 (Intron)	ARID2:PPI(0.515)		1.394
chr12	46287180	C	G	ARID2 (Intron)	ARID2:PPI(0.515)		1.006
chr12	46290504	T	G	ARID2 (Intron)	ARID2:PPI(0.515)		0.394
chr12	46208604	A	T	ARID2 (Intron)	ARID2:PPI(0.515)		0.209
chr15	72855682	G	A	ARIH1 (Intron)	ARIH1:PPI(0.718)REG(0.761)		0.463
chr1	150786721	G	C	ARNT (Intron)	ARNT:PPI(0.838)REG(0.409)		2.013
chr1	150789069	G	T	ARNT (Intron)	ARNT:PPI(0.838)REG(0.409)		0.593
chr17	80281541	T	C	ASPSCR1 (Distal)	ASPSCR1:PHOS(0.908)PPI(0.307)REG(0.830),CD7:PPI(0.448)REG(0.409),NOTUM:PPI(0.142)REG(0.761),SECTM1:PPI(0.142)REG(0.909)		0.932
chr17	80281657	T	G	ASPSCR1 (Distal)	ASPSCR1:PHOS(0.908)PPI(0.307)REG(0.830),CD7:PPI(0.448)REG(0.409),NOTUM:PPI(0.142)REG(0.761),SECTM1:PPI(0.142)REG(0.909)		0.932
chr3	130737206	G	T	ASTE1 (Intron)	ASTE1:PHOS(0.276)PPI(0.142)		0.096
chr2	175976433	G	T	ATF2 (Intron)	ATF2:PHOS(0.634)PPI(0.917)REG(0.830)		1.574
chr2	175982713	C	G	ATF2 (Intron)	ATF2:PHOS(0.634)PPI(0.917)REG(0.830)		1.389
chr19	50868754	A	G	ATF5 (Distal)	ATF5:PHOS(0.276)PPI(0.718),IL4I1:PPI(0.142)REG(0.909),KLK10:PPI(0.361),KLK5:PPI(0.307),KLK6:PPI(0.307),MYH14:PPI(0.606)REG(0.409),NAPSA:PPI(0.482)REG(0.409),NR1H2:PPI(0.846)REG(0.830),SPIB:PPI(0.685)		1.544
chr2	216177115	T	G	ATIC (Promoter and UTR)	ATIC:PPI(0.835)REG(0.409)	MOTIFG=ZEB1_known1#216177110#216177116#-#1#6794#3667	2.361
chr2	216182854	T	C	ATIC (UTR)	ATIC:PPI(0.835)REG(0.409)		1.211
chr11	108158517	C	T	ATM (Intron)	ATM:PHOS(0.973)PPI(0.895)REG(0.409)		0.918

chr11	108129982	C	T	ATM (Intron)	ATM:PHOS(0.973)PPI(0.895)REG(0.409)		0.918
chr11	108205573	A	T	ATM (Intron)	ATM:PHOS(0.973)PPI(0.895)REG(0.409)		1.536
chr11	108202297	G	A	ATM (Intron)	ATM:PHOS(0.973)PPI(0.895)REG(0.409)		0.918
chr11	108201175	A	C	ATM (Intron)	ATM:PHOS(0.973)PPI(0.895)REG(0.409)		0.918
chr11	108216947	C	A	ATM (Intron)	ATM:PHOS(0.973)PPI(0.895)REG(0.409)		0.918
chr11	108201227	A	G	ATM (Intron)	ATM:PHOS(0.973)PPI(0.895)REG(0.409)		0.918
chr11	108204543	G	C	ATM (Intron)	ATM:PHOS(0.973)PPI(0.895)REG(0.409)		0.918
chr11	108236253	A	C	ATM (UTR)	ATM:PHOS(0.973)PPI(0.895)REG(0.409)		1.541
chr16	81060105	T	G	ATMIN (Medial)	ATMIN:REG(0.761),CENPN:PPI(0.923)REG(0.409)		2.589
chr16	81060105	T	G	ATMIN (Medial)	ATMIN:REG(0.761),CENPN:PPI(0.923)REG(0.409)		2.589
chr16	81060105	T	G	ATMIN (Medial)	ATMIN:REG(0.761),CENPN:PPI(0.923)REG(0.409)		2.589
chr16	81060105	T	G	ATMIN (Medial)	ATMIN:REG(0.761),CENPN:PPI(0.923)REG(0.409)		2.589
chr1	116929816	T	G	ATP1A1 (Intron)	ATP1A1:PPI(0.718)		0.403
chr1	116932011	C	A	ATP1A1 (Intron)	ATP1A1:PPI(0.718)		0.403
chr1	116952787	C	T	ATP1A1 (UTR)	ATP1A1:PPI(0.718)		1.200
chr21	27107738	G	A	ATP5J (Promoter and UTR), GABPA (UTR)	ATP5J:PPI(0.695),GABPA:PHOS(0.276)PPI(0.729)REG(0.999)		1.796
chr3	142188432	A	T	ATR (Intron and Promoter)	ATR:PPI(0.953)REG(0.634)		0.861
chr3	142234359	T	C	ATR (Intron)	ATR:PPI(0.953)REG(0.634)		1.484
chr3	142185400	A	C	ATR (Intron)	ATR:PPI(0.953)REG(0.634)		1.484
chr3	142216126	A	C	ATR (Intron)	ATR:PPI(0.953)REG(0.634)		1.484
chr3	142211949	G	C	ATR (Intron)	ATR:PPI(0.953)REG(0.634)		1.484
chr3	142226958	C	G	ATR (Intron)	ATR:PPI(0.953)REG(0.634)		0.861
chr3	142259639	T	G	ATR (Intron)	ATR:PPI(0.953)REG(0.634)		0.861
chr3	142257697	A	T	ATR (Intron)	ATR:PPI(0.953)REG(0.634)		0.861
chr3	142272303	C	T	ATR (Intron)	ATR:PPI(0.953)REG(0.634)		0.861
chr3	142268523	A	C	ATR (Intron)	ATR:PPI(0.953)REG(0.634)		0.861
chr3	142284719	C	A	ATR (Intron)	ATR:PPI(0.953)REG(0.634)		0.861

chr3	142185378	G	A	ATR (Intron)	ATR:PPI(0.953)REG(0.634)		0.861
chr3	142234116	A	C	ATR (Intron)	ATR:PPI(0.953)REG(0.634)		0.861
chr3	142274532	C	A	ATR (Intron)	ATR:PPI(0.953)REG(0.634)		0.861
chr3	142218391	C	T	ATR (Intron)	ATR:PPI(0.953)REG(0.634)		0.861
chr3	142171880	C	T	ATR (UTR)	ATR:PPI(0.953)REG(0.634)		0.861
chr3	142169058	A	G	ATR (UTR)	ATR:PPI(0.953)REG(0.634),XRN1:PPI(0.983)		1.134
chr3	48495627	G	T	ATRIP (Intron)	ATRIP:PPI(0.623)		0.296
chr14	92547291	T	G	ATXN3 (Intron)	ATXN3:PHOS(0.398)PPI(0.767)REG(0.830)		1.201
chr14	92559963	C	T	ATXN3 (Intron)	ATXN3:PHOS(0.398)PPI(0.767)REG(0.830)		0.764
chr20	54945166	A	G	AURKA (UTR)	AURKA:PPI(0.969)REG(0.952)		1.092
chr15	45003647	C	T	B2M (Promoter)	B2M:PPI(0.934)REG(0.929)		2.576
chr19	17387297	T	G	BABAM1 (Intron)	BABAM1:PPI(0.142),USHBP1:PPI(0.841)		1.784
chr19	17387297	T	G	BABAM1 (Intron)	BABAM1:PPI(0.142),USHBP1:PPI(0.841)		1.784
chr19	17384879	C	T	BABAM1 (Intron)	BABAM1:PPI(0.142),USHBP1:PPI(0.841)		0.599
chr19	17387534	T	G	BABAM1 (Intron)	BABAM1:PPI(0.142),USHBP1:PPI(0.841)		0.599
chr19	17379534	G	A	BABAM1 (Promoter and UTR)	BABAM1:PPI(0.142)		1.248
chr19	17379534	G	A	BABAM1 (Promoter and UTR)	BABAM1:PPI(0.142)		1.248
chr19	17379558	C	T	BABAM1 (Promoter and UTR)	BABAM1:PPI(0.142)		1.248
chr3	52440787	C	T	BAP1 (Intron and Promoter)	BAP1:PPI(0.142)		0.685
chr3	52438930	G	A	BAP1 (Intron and Promoter)	BAP1:PPI(0.142),PHF7:PHOS(0.276)PPI(0.307)		0.292
chr3	52437324	G	T	BAP1 (Intron)	BAP1:PPI(0.142)		0.685
chr19	49464892	G	A	BAX (UTR)	BAX:PHOS(0.728)PPI(0.817)REG(0.929)		1.604
chr15	80259926	G	C	BCL2A1 (Intron)	BCL2A1:PPI(0.641)REG(0.634)		0.499
chr12	12340340	C	T	BCL2L14 (Intron), LRP6 (Intron)	BCL2L14:PHOS(0.398)PPI(0.361),LRP6:PPI(0.787)REG(0.409)		0.578
chr12	12353467	T	G	BCL2L14 (Intron), LRP6 (Intron)	BCL2L14:PHOS(0.398)PPI(0.361),LRP6:PPI(0.787)REG(0.409)		0.503
chrX	39911657	C	A	BCOR (Intron)	BCOR:PHOS(0.566)PPI(0.685)		1.362
chrX	39911657	C	A	BCOR (Intron)	BCOR:PHOS(0.566)PPI(0.685)		1.362

chrX	39911657	C	A	BCOR (Intron)	BCOR:PHOS(0.566)PPI(0.685)		1.362
chrX	39937317	G	T	BCOR (Intron)	BCOR:PHOS(0.566)PPI(0.685)		1.170
chrX	39935701	T	C	BCOR (Intron)	BCOR:PHOS(0.566)PPI(0.685)		0.985
chrX	39930770	C	T	BCOR (Intron)	BCOR:PHOS(0.566)PPI(0.685)		0.985
chrX	39914590	G	T	BCOR (Intron)	BCOR:PHOS(0.566)PPI(0.685)		0.547
chr22	23603134	C	T	BCR (Intron)	BCR:PHOS(0.925)PPI(0.897)REG(0.986)		2.378
chr22	23653880	G	A	BCR (Intron)	BCR:PHOS(0.925)PPI(0.897)REG(0.986)		1.958
chr22	23653880	G	A	BCR (Intron)	BCR:PHOS(0.925)PPI(0.897)REG(0.986)		1.958
chr22	23656148	T	G	BCR (Intron)	BCR:PHOS(0.925)PPI(0.897)REG(0.986)		1.766
chr22	23540414	T	G	BCR (Intron)	BCR:PHOS(0.925)PPI(0.897)REG(0.986)		1.755
chr22	23606384	C	T	BCR (Intron)	BCR:PHOS(0.925)PPI(0.897)REG(0.986)		1.755
chr22	23631823	T	C	BCR (Intron)	BCR:PHOS(0.925)PPI(0.897)REG(0.986)		1.755
chr22	23659581	G	A	BCR (UTR)	BCR:PHOS(0.925)PPI(0.897)REG(0.986)		1.580
chr13	103519205	C	T	BIVM-ERCC5 (Intron), ERCC5 (Intron)	ERCC5:PPI(0.822)REG(0.409)		0.564
chr15	91308836	G	A	BLM (Intron)	BLM:PPI(0.883)REG(0.634)		1.923
chr15	91308899	G	A	BLM (Intron)	BLM:PPI(0.883)REG(0.634)		1.872
chr15	91303818	C	T	BLM (Intron)	BLM:PPI(0.883)REG(0.634)		1.309
chr15	91308496	G	A	BLM (Intron)	BLM:PPI(0.883)REG(0.634)		1.309
chr15	91304486	G	T	BLM (Intron)	BLM:PPI(0.883)REG(0.634)		1.309
chr15	91306046	G	C	BLM (Intron)	BLM:PPI(0.883)REG(0.634)		0.686
chr15	91295009	T	A	BLM (Intron)	BLM:PPI(0.883)REG(0.634)		0.686
chr15	91312489	T	G	BLM (Intron)	BLM:PPI(0.883)REG(0.634)		0.686
chr10	88683122	T	C	BMPR1A (Intron)	BMPR1A:PHOS(0.736)PPI(0.774)REG(0.634)		1.483
chr10	88683122	T	C	BMPR1A (Intron)	BMPR1A:PHOS(0.736)PPI(0.774)REG(0.634)		1.483
chr12	112121114	A	G	BRAP (Intron and Promoter)	BRAP:PPI(0.718)REG(0.761)		0.648
chr17	41622740	G	T	BRCA1 (Distal)	BRCA1:PHOS(0.651)PPI(0.995)REG(0.992),ETV4 :PHOS(0.693)PPI(0.361)REG(0.952)		1.171
chr17	41228726	C	A	BRCA1 (Intron)	BRCA1:PHOS(0.651)PPI(0.995)REG(0.992)		1.609
chr17	41246929	A	C	BRCA1 (Intron)	BRCA1:PHOS(0.651)PPI(0.995)REG(0.992)		1.609
chr17	41249309	A	T	BRCA1 (Intron)	BRCA1:PHOS(0.651)PPI(0.995)REG(0.992)		1.609
chr17	41219780	T	C	BRCA1 (Intron)	BRCA1:PHOS(0.651)PPI(0.995)REG(0.992)		1.609

chr17	41222831	G	T	BRCA1 (Intron)	BRCA1:PHOS(0.651)PPI(0.995)REG(0.992)		0.986
chr17	41256088	A	G	BRCA1 (Intron)	BRCA1:PHOS(0.651)PPI(0.995)REG(0.992)		0.986
chr17	41275946	C	T	BRCA1 (Intron)	BRCA1:PHOS(0.651)PPI(0.995)REG(0.992)		0.986
chr17	41251612	G	C	BRCA1 (Intron)	BRCA1:PHOS(0.651)PPI(0.995)REG(0.992)		0.986
chr17	41219804	T	C	BRCA1 (Intron)	BRCA1:PHOS(0.651)PPI(0.995)REG(0.992)		0.986
chr13	32932067	G	A	BRCA2 (Intron)	BRCA2:PPI(0.767)REG(0.634)		1.095
chr13	32900583	C	A	BRCA2 (Intron)	BRCA2:PPI(0.767)REG(0.634)		1.095
chr13	32929615	G	T	BRCA2 (Intron)	BRCA2:PPI(0.767)REG(0.634)		0.472
chr13	32920772	G	C	BRCA2 (Intron)	BRCA2:PPI(0.767)REG(0.634)		0.472
chr13	32920818	T	C	BRCA2 (Intron)	BRCA2:PPI(0.767)REG(0.634)		0.472
chr13	32899115	C	T	BRCA2 (Intron)	BRCA2:PPI(0.767)REG(0.634)		0.472
chr9	136913577	C	T	BRD3 (Intron)	BRD3:PHOS(0.709)PPI(0.947)		1.467
chr2	28464261	G	A	BRE (Intron)	BRE:PPI(0.408)		0.771
chr2	28210755	C	G	BRE (Intron)	BRE:PPI(0.408),MRPL33:REG(0.409)		0.148
chr8	37704368	G	A	BRF2 (Intron)	BRF2:PPI(0.942)REG(0.984)		1.759
chr8	37706029	T	A	BRF2 (Intron)	BRF2:PPI(0.942)REG(0.984)		0.951
chr17	59926384	T	G	BRIP1 (Intron)	BRIP1:PPI(0.307)REG(0.761)		1.648
chr17	59926384	T	G	BRIP1 (Intron)	BRIP1:PPI(0.307)REG(0.761)		1.648
chr17	59821953	C	G	BRIP1 (Intron)	BRIP1:PPI(0.307)REG(0.761)		1.271
chr17	59822104	G	C	BRIP1 (Intron)	BRIP1:PPI(0.307)REG(0.761)		0.648
chr17	59822131	G	C	BRIP1 (Intron)	BRIP1:PPI(0.307)REG(0.761)		0.648
chr17	59937116	G	A	BRIP1 (Intron)	BRIP1:PPI(0.307)REG(0.761)		0.463
chr17	59857922	C	G	BRIP1 (Intron)	BRIP1:PPI(0.307)REG(0.761)		0.463
chr17	59822030	G	A	BRIP1 (Intron)	BRIP1:PPI(0.307)REG(0.761)		0.463
chr2	111419153	G	C	BUB1 (Intron)	BUB1:PHOS(0.902)PPI(0.671)REG(0.409)		1.353
chr2	111416320	C	A	BUB1 (Intron)	BUB1:PHOS(0.902)PPI(0.671)REG(0.409)		1.353
chr2	111411203	G	A	BUB1 (Intron)	BUB1:PHOS(0.902)PPI(0.671)REG(0.409)		1.353
chr2	111411005	C	T	BUB1 (Intron)	BUB1:PHOS(0.902)PPI(0.671)REG(0.409)		1.328
chr2	111415911	C	G	BUB1 (Intron)	BUB1:PHOS(0.902)PPI(0.671)REG(0.409)		0.730

chr2	111424039	C	A	BUB1 (Intron)	BUB1:PHOS(0.902)PPI(0.671)REG(0.409)		0.730
chr15	40462972	A	C	BUB1B (Intron)	BUB1B:PPI(0.990)REG(0.761)		0.970
chr15	40505835	C	G	BUB1B (Intron)	BUB1B:PPI(0.990)REG(0.761)		0.970
chr11	76253069	C	G	C11orf30 (Intron and Promoter)			0.626
chr11	76253013	C	T	C11orf30 (Intron and Promoter)			0.003
chr11	76170832	A	G	C11orf30 (Intron)			1.623
chr11	76227182	G	A	C11orf30 (Intron)			0.626
chr19	33464541	A	C	C19orf40 (Intron)		MOTIFG=ZEB1_known1#33464539#33464545#+#3#6794#2424	0.983
chr19	33464469	G	A	C19orf40 (Intron)			0.626
chr1	186342612	A	C	C1orf27 (Promoter), TPR (Intron)	TPR:PPI(0.908)REG(0.761)		0.744
chr19	1369215	T	G	C2CD4C (Distal)			0.188
chr7	47855144	T	G	C7orf69 (Intron), HUS1 (Intron)	HUS1:PPI(0.787)REG(0.409),PKD1L1:REG(0.634)		1.311
chr7	47853690	G	A	C7orf69 (Intron), HUS1 (Intron)	HUS1:PPI(0.787)REG(0.409),PKD1L1:REG(0.634)		0.503
chr7	47849203	A	T	C7orf69 (Intron), HUS1 (Intron)	HUS1:PPI(0.787)REG(0.409),PKD1L1:REG(0.634)		0.688
chr7	47835417	G	A	C7orf69 (Intron), HUS1 (Intron)	HUS1:PPI(0.787)REG(0.409),PKD1L1:REG(0.634)		0.503
chr7	47857537	C	A	C7orf69 (Intron), HUS1 (Intron)	HUS1:PPI(0.787)REG(0.409),PKD1L1:REG(0.634)		0.503
chrX	15800600	A	G	CA5B (Intron), ZRSR2 (Medial)	CA5B:PHOS(0.276),ZRSR2:PPI(0.835)		1.396
chr3	53700328	C	A	CACNA1D (Intron and Promoter)	CACNA1D:PPI(0.718)		0.403
chr3	53783175	T	G	CACNA1D (Intron)	CACNA1D:PPI(0.718)		2.818
chr3	53783269	T	G	CACNA1D (Intron)	CACNA1D:PPI(0.718)		2.200
chr3	53808575	G	A	CACNA1D (Intron)	CACNA1D:PPI(0.718)		1.211
chr3	53685088	C	G	CACNA1D (Intron)	CACNA1D:PPI(0.718)		1.211
chr3	53796135	A	C	CACNA1D (Intron)	CACNA1D:PPI(0.718)		1.025
chr3	53788732	A	C	CACNA1D (Intron)	CACNA1D:PPI(0.718)		1.025
chr3	53753811	C	T	CACNA1D (Intron)	CACNA1D:PPI(0.718)		1.025
chr3	53752101	C	A	CACNA1D (Intron)	CACNA1D:PPI(0.718)		0.596
chr3	53837630	G	T	CACNA1D (Intron)	CACNA1D:PPI(0.718)		0.588

chr3	53837330	T	G	CACNA1D (Intron)	CACNA1D:PPI(0.718)		0.588
chr3	53757377	G	A	CACNA1D (Intron)	CACNA1D:PPI(0.718)		0.403
chr3	53756209	C	T	CACNA1D (Intron)	CACNA1D:PPI(0.718)		0.403
chr1	7815554	G	A	CAMTA1 (Intron)	CAMTA1:REG(0.909)		0.932
chr12	67691711	G	A	CAND1 (Intron and Promoter)	CAND1:PHOS(0.509)PPI(0.540)REG(0.761)		0.463
chr12	67704109	T	C	CAND1 (Intron)	CAND1:PHOS(0.509)PPI(0.540)REG(0.761)		2.055
chr12	67698833	C	G	CAND1 (Intron)	CAND1:PHOS(0.509)PPI(0.540)REG(0.761)		0.463
chr12	67706629	G	T	CAND1 (UTR)	CAND1:PHOS(0.509)PPI(0.540)REG(0.761)		1.085
chr7	2968336	C	G	CARD11 (Intron)	CARD11:PPI(0.695)		1.182
chr7	2976910	A	C	CARD11 (Intron)	CARD11:PPI(0.695)		0.997
chr7	2974013	C	T	CARD11 (Intron)	CARD11:PPI(0.695)		0.559
chr7	2985391	C	G	CARD11 (Intron)	CARD11:PPI(0.695)		0.559
chr7	2958948	C	A	CARD11 (Intron)	CARD11:PPI(0.695)		0.559
chr7	2968189	T	G	CARD11 (Intron)	CARD11:PPI(0.695)		0.374
chr7	2976962	G	C	CARD11 (Intron)	CARD11:PPI(0.695)		0.374
chr7	2977469	G	A	CARD11 (Intron)	CARD11:PPI(0.695)		0.374
chr11	3041444	G	A	CARS (Intron)	CARS:PPI(0.448)REG(0.634)		0.930
chr11	3061192	A	C	CARS (Intron)	CARS:PPI(0.448)REG(0.634)		0.307
chr4	185556618	C	T	CASP3 (Intron)	CASP3:PPI(0.983)		1.134
chr2	202136219	G	T	CASP8 (Intron and Promoter)	CASP8:PPI(0.961)REG(0.634)		0.883
chr2	202150337	A	G	CASP8 (Intron)	CASP8:PPI(0.961)REG(0.634)		1.069
chr7	116165024	C	T	CAV1 (Promoter and UTR)	CAV1:PPI(0.961)REG(0.634)		1.691
chr16	67100703	T	C	CBFB (Intron)	CBFB:PHOS(0.540)PPI(0.448)		0.849
chr11	119158764	C	A	CBL (Intron)	CBL:PHOS(0.903)PPI(0.991)REG(0.409)		1.025
chr11	119158508	G	C	CBL (Intron)	CBL:PHOS(0.903)PPI(0.991)REG(0.409)		0.973
chr3	105423083	G	T	CBLB (Intron and Promoter)	CBLB:PPI(0.956)REG(0.409)		1.492
chr3	105495616	T	A	CBLB (Intron)	CBLB:PPI(0.956)REG(0.409)		1.492
chr3	105404395	G	A	CBLB (Intron)	CBLB:PPI(0.956)REG(0.409)		1.492
chr3	105464677	T	G	CBLB (Intron)	CBLB:PPI(0.956)REG(0.409)		0.869
chr3	105455985	T	C	CBLB (Intron)	CBLB:PPI(0.956)REG(0.409)		0.869
chr3	105439184	A	C	CBLB (Intron)	CBLB:PPI(0.956)REG(0.409)		0.869
chr19	45303631	A	G	CBLC (Intron)	CBLC:PPI(0.908)		0.929
chr7	26242423	T	A	CBX3 (Intron and Medial)	CBX3:PPI(0.826)REG(0.880),HNRNPA2B1:PPI(0.953)REG(0.929)		2.658

chr7	26236284	A	C	CBX3 (Medial)	CBX3:PPI(0.826)REG(0.880),HNRNPA2B1:PPI(0.953)REG(0.929)		2.296
chr7	26240296	G	T	CBX3 (Promoter), HNRNPA2B1 (Medial and UTR)	CBX3:PPI(0.826)REG(0.880),HNRNPA2B1:PPI(0.953)REG(0.929)	MOTIFG=HNF4_known7#26240296#26240302#-#6#6101#3259	5.225
chr2	223160203	C	A	CCDC140 (Medial)	CCDC140:REG(0.761),PAX3:PPI(0.835)REG(0.761)		0.773
chr13	37014112	C	T	CCNA1 (Intron)	CCNA1:PPI(0.947)		0.844
chr4	122741638	C	T	CCNA2 (Intron)	CCNA2:PPI(0.877)		0.673
chr5	68470252	A	C	CCNB1 (Intron)	CCNB1:PHOS(0.276)PPI(0.954)REG(0.409)		0.864
chr5	68463014	G	T	CCNB1 (Promoter and UTR)	CCNB1:PHOS(0.276)PPI(0.954)REG(0.409)		2.661
chr5	68463014	G	T	CCNB1 (Promoter and UTR)	CCNB1:PHOS(0.276)PPI(0.954)REG(0.409)		2.661
chr14	20794744	C	A	CCNB1IP1 (Intron)	CCNB1IP1:PPI(0.361)REG(0.830)		0.764
chr6	41904474	G	A	CCND3 (Intron)	CCND3:PHOS(0.634)PPI(0.835)REG(0.761)		1.385
chr5	86695187	A	T	CCNH (Intron)	CCNH:PHOS(0.398)PPI(0.960)		1.066
chr5	86704006	A	G	CCNH (Intron)	CCNH:PHOS(0.398)PPI(0.960)		0.880
chr5	86697694	A	C	CCNH (Intron)	CCNH:PHOS(0.398)PPI(0.960)		0.880
chr5	86690224	C	A	CCNH (UTR)	CCNH:PHOS(0.398)PPI(0.960)		1.688
chr2	135704036	G	A	CCNT2 (Intron and Promoter)	CCNT2:PPI(0.875)REG(0.989)		1.590
chr2	135710205	T	G	CCNT2 (Intron)	CCNT2:PPI(0.875)REG(0.989)		1.590
chr2	135694379	C	T	CCNT2 (Intron)	CCNT2:PPI(0.875)REG(0.989)		1.590
chr2	135694354	G	C	CCNT2 (Intron)	CCNT2:PPI(0.875)REG(0.989)		1.590
chr2	135700151	A	T	CCNT2 (Intron)	CCNT2:PPI(0.875)REG(0.989)		0.978
chr2	135696673	C	A	CCNT2 (Intron)	CCNT2:PPI(0.875)REG(0.989)		0.967
chr9	5465496	C	G	CD274 (Intron)	CD274:PPI(0.142)REG(0.761)		1.085
chr9	5465354	C	G	CD274 (Intron)	CD274:PPI(0.142)REG(0.761)		0.648
chr9	5456270	G	A	CD274 (Intron)	CD274:PPI(0.142)REG(0.761)		0.463
chr9	5467717	C	T	CD274 (Intron)	CD274:PPI(0.142)REG(0.761)		0.463
chr9	5467955	G	A	CD274 (UTR)	CD274:PPI(0.142)REG(0.761)		1.085
chr19	45912775	C	G	CD3EAP (UTR), ERCC1 (UTR)	CD3EAP:PPI(0.809)REG(0.634),ERCC1:PPI(0.902)REG(0.830)		0.730
chr5	149782267	A	C	CD74 (Intron and Medial)	CD74:PPI(0.718)REG(0.967)		2.086
chr5	149777778	C	T	CD74 (Medial)	CD74:PPI(0.718)REG(0.967),TCOF1:PPI(0.482)		2.086

chr1	43814929	C	T	CDC20 (Medial)	CDC20:PPI(0.973)REG(0.761),MPL:PPI(0.921)		1.715
chr3	48224585	C	A	CDC25A (Intron)	CDC25A:PHOS(0.601)PPI(0.817)REG(0.634)		1.363
chr3	48206079	C	A	CDC25A (Intron)	CDC25A:PHOS(0.601)PPI(0.817)REG(0.634)		0.555
chr20	3782181	C	G	CDC25B (Intron and Medial)	CDC25B:PPI(0.887)		0.881
chr17	45235570	A	C	CDC27 (Intron)	CDC27:PPI(0.908)REG(0.409)		3.161
chr17	45235547	C	A	CDC27 (Intron)	CDC27:PPI(0.908)REG(0.409)		2.539
chr17	45235548	C	A	CDC27 (Intron)	CDC27:PPI(0.908)REG(0.409)		2.539
chr17	45234787	G	T	CDC27 (Intron)	CDC27:PPI(0.908)REG(0.409)	MOTIFBR=IRF3#Irf_disc5_8mer#45234784#45234794#+#4#0000000#0244604	2.520
chr17	45234122	A	C	CDC27 (Intron)	CDC27:PPI(0.908)REG(0.409)		1.541
chr17	45206905	C	T	CDC27 (Intron)	CDC27:PPI(0.908)REG(0.409)		0.744
chr17	45234097	A	C	CDC27 (Intron)	CDC27:PPI(0.908)REG(0.409)		0.744
chr17	38457605	G	C	CDC6 (Intron)	CDC6:PPI(0.921)REG(0.634)		0.961
chr1	193181674	G	A	CDC73 (Intron)	CDC73:PPI(0.781)REG(0.761)		1.116
chr1	193099419	C	A	CDC73 (Intron)	CDC73:PPI(0.781)REG(0.761)		1.100
chr1	193181097	G	A	CDC73 (Intron)	CDC73:PPI(0.781)REG(0.761)		0.679
chr1	193121576	T	C	CDC73 (Intron)	CDC73:PPI(0.781)REG(0.761)		0.494
chr1	193104489	T	C	CDC73 (Intron)	CDC73:PPI(0.781)REG(0.761)		0.494
chr1	193099603	G	T	CDC73 (Intron)	CDC73:PPI(0.781)REG(0.761)		0.494
chr1	193203348	C	T	CDC73 (Intron)	CDC73:PPI(0.781)REG(0.761)		0.494
chr16	68856129	G	A	CDH1 (Intron)	CDH1:PPI(0.953)REG(0.409)		2.484
chr16	68856129	G	T	CDH1 (Intron)	CDH1:PPI(0.953)REG(0.409)		2.484
chr16	68861970	T	G	CDH1 (Intron)	CDH1:PPI(0.953)REG(0.409)		2.046
chr16	68861970	T	G	CDH1 (Intron)	CDH1:PPI(0.953)REG(0.409)		2.046
chr16	68861970	T	G	CDH1 (Intron)	CDH1:PPI(0.953)REG(0.409)		2.046
chr16	68861970	T	G	CDH1 (Intron)	CDH1:PPI(0.953)REG(0.409)		2.046
chr16	68861970	T	G	CDH1 (Intron)	CDH1:PPI(0.953)REG(0.409)		2.046
chr16	68863554	A	G	CDH1 (Intron)	CDH1:PPI(0.953)REG(0.409)		1.669
chr16	68844098	A	G	CDH1 (Intron)	CDH1:PPI(0.953)REG(0.409)		1.669
chr16	68846166	G	A	CDH1 (Intron)	CDH1:PPI(0.953)REG(0.409)		1.669
chr16	68849663	G	A	CDH1 (Intron)	CDH1:PPI(0.953)REG(0.409)		1.484
chr16	68844244	G	A	CDH1 (Intron)	CDH1:PPI(0.953)REG(0.409)		1.484
chr16	68847399	G	C	CDH1 (Intron)	CDH1:PPI(0.953)REG(0.409)		1.484
chr16	68844245	G	A	CDH1 (Intron)	CDH1:PPI(0.953)REG(0.409)		1.484
chr16	68856196	A	C	CDH1 (Intron)	CDH1:PPI(0.953)REG(0.409)		1.234

chr16	68847097	T	G	CDH1 (Intron)	CDH1:PPI(0.953)REG(0.409)		1.046
chr16	68857213	T	G	CDH1 (Intron)	CDH1:PPI(0.953)REG(0.409)		1.046
chr16	68862212	G	C	CDH1 (Intron)	CDH1:PPI(0.953)REG(0.409)		0.861
chr16	68847516	C	G	CDH1 (Intron)	CDH1:PPI(0.953)REG(0.409)		0.861
chr16	68843899	T	G	CDH1 (Intron)	CDH1:PPI(0.953)REG(0.409)		0.861
chr16	68862067	A	C	CDH1 (Intron)	CDH1:PPI(0.953)REG(0.409)		0.861
chr16	65032461	G	A	CDH11 (Intron)	CDH11:PPI(0.408)		0.148
chr16	83519982	G	T	CDH13 (Intron)	CDH13:PPI(0.236)		0.708
chr16	83520078	G	A	CDH13 (Intron)	CDH13:PPI(0.236)		0.270
chr16	89846521	G	C	CDH15 (Distal)	CDH15:PPI(0.515)REG(0.409),DBNDD1:REG(0.409),FANCA:PHOS(0.276)PPI(0.916)REG(0.634)		0.949
chr18	25570330	G	A	CDH2 (Intron)	CDH2:PPI(0.831)		2.203
chr18	25570330	G	A	CDH2 (Intron)	CDH2:PPI(0.831)		2.203
chr18	25570330	G	A	CDH2 (Intron)	CDH2:PPI(0.831)		2.203
chr18	25570318	G	A	CDH2 (Intron)	CDH2:PPI(0.831)		1.580
chr18	25570318	G	A	CDH2 (Intron)	CDH2:PPI(0.831)		1.580
chr18	25543284	G	A	CDH2 (Intron)	CDH2:PPI(0.831)		0.765
chr18	25568423	C	A	CDH2 (Intron)	CDH2:PPI(0.831)		0.580
chr18	25570437	C	T	CDH2 (Intron)	CDH2:PPI(0.831)		0.580
chr18	59170056	T	G	CDH20 (Intron)			0.188
chr18	59206533	T	G	CDH20 (Intron)			0.003
chr18	59167743	A	T	CDH20 (Intron)			0.003
chr18	59157665	C	T	CDH20 (Promoter and UTR)			0.003
chr10	62553823	C	A	CDK1 (UTR)	CDK1:PPI(0.853)REG(0.634)		1.246
chr17	37676485	T	G	CDK12 (Intron)	CDK12:PPI(0.142)REG(0.944)		1.086
chr17	37676443	G	A	CDK12 (Intron)	CDK12:PPI(0.142)REG(0.944)		1.021
chr17	37668049	G	C	CDK12 (Intron)	CDK12:PPI(0.142)REG(0.944)		0.836
chr12	56361549	T	G	CDK2 (Intron)	CDK2:PHOS(0.828)PPI(0.994),RAB5B:PHOS(0.601)PPI(0.583)REG(0.952)		1.780
chr12	56365527	A	C	CDK2 (UTR)	CDK2:PHOS(0.828)PPI(0.994),RAB5B:PHOS(0.601)PPI(0.583)REG(0.952)		1.780
chr12	56365635	A	C	CDK2 (UTR)	CDK2:PHOS(0.828)PPI(0.994),RAB5B:PHOS(0.601)PPI(0.583)REG(0.952)		1.168
chr13	26967647	G	A	CDK8 (Intron)	CDK8:PPI(0.966)		1.706
chr12	12871892	G	A	CDKN1B (Intron and Medial)	CDKN1B:PPI(0.895)REG(0.409)		2.522
chr12	12871894	T	G	CDKN1B (Intron and Medial)	CDKN1B:PPI(0.895)REG(0.409)		1.899

chr12	12870230	G	A	CDKN1B (Promoter and UTR)	CDKN1B:PPI(0.895)REG(0.409)	MOTIFBR=JUND#AP-1_disc10_8mer#12870227#12870237#+#4#0225718#0430917	4.111
chr12	12870769	G	A	CDKN1B (Promoter and UTR)	CDKN1B:PPI(0.895)REG(0.409)		2.522
chr20	5159428	T	G	CDS2 (Intron and Promoter)	CDS2:PPI(0.307)REG(0.761)		0.463
chr20	5155690	T	G	CDS2 (Intron)	CDS2:PPI(0.307)REG(0.761)		1.463
chr20	5155690	T	G	CDS2 (Intron)	CDS2:PPI(0.307)REG(0.761)		1.463
chr20	5155704	T	G	CDS2 (Intron)	CDS2:PPI(0.307)REG(0.761)		1.260
chr20	5155932	G	T	CDS2 (Intron)	CDS2:PPI(0.307)REG(0.761)		0.463
chr20	5099516	G	A	CDS2 (Medial)	CDS2:PPI(0.307)REG(0.761),PCNA:PPI(0.997)REG(0.944)		3.381
chr13	28537537	T	G	CDX2 (Intron)	CDX2:PPI(0.746)REG(0.830)		0.578
chr13	28537242	A	C	CDX2 (UTR)	CDX2:PPI(0.746)REG(0.830)		0.578
chr11	117280711	A	C	CEP164 (Intron)	CEP164:REG(0.409)		0.350
chr11	117222492	C	A	CEP164 (Intron)	CEP164:REG(0.409)		0.148
chr11	117234130	C	T	CEP164 (Intron)	CEP164:REG(0.409)		0.148
chr1	150934382	T	G	CERS2 (Intron), SETDB1 (Intron)	SETDB1:PHOS(0.276)PPI(0.984)REG(0.992)		1.162
chr1	150935895	T	G	CERS2 (Intron), SETDB1 (Intron)	SETDB1:PHOS(0.276)PPI(0.984)REG(0.992)		1.162
chr1	150935931	T	G	CERS2 (Intron), SETDB1 (Intron)	SETDB1:PHOS(0.276)PPI(0.984)REG(0.992)		1.162
chr1	150934684	A	C	CERS2 (Intron), SETDB1 (Intron)	SETDB1:PHOS(0.276)PPI(0.984)REG(0.992)		0.976
chr11	65617898	G	C	CFL1 (Intron), MUS81 (Medial)	CFL1:PPI(0.945)REG(0.929),MUS81:PPI(0.927)REG(0.880),SNX32:PPI(0.142)REG(0.761)		2.647
chr11	65617137	A	C	CFL1 (Intron), MUS81 (Medial)	CFL1:PPI(0.945)REG(0.929),MUS81:PPI(0.927)REG(0.880),SNX32:PPI(0.142)REG(0.761)		2.024
chr11	65620338	C	G	CFL1 (Intron), MUS81 (Medial)	CFL1:PPI(0.945)REG(0.929),MUS81:PPI(0.927)REG(0.880),SNX32:PPI(0.142)REG(0.761)		2.024
chr11	65629556	T	G	CFL1 (Promoter), MUS81 (Intron)	CFL1:PPI(0.945)REG(0.929),MUS81:PPI(0.927)REG(0.880)	MOTIFG=SREBP_known2#65629555#65629562#-#6#8589#4374	2.006
chr11	65630008	C	G	CFL1 (Promoter), MUS81 (Intron)	CFL1:PPI(0.945)REG(0.929),MUS81:PPI(0.927)REG(0.880)		0.839

chr21	37781255	T	C	CHAF1B (Intron)	CHAF1B:PHOS(0.670)PPI(0.934)		0.809
chr12	6710219	C	A	CHD4 (Intron)	CHD4:PHOS(0.459)PPI(0.912)		1.562
chr12	6690650	C	T	CHD4 (Intron)	CHD4:PHOS(0.459)PPI(0.912)		1.562
chr12	6691750	C	G	CHD4 (Intron)	CHD4:PHOS(0.459)PPI(0.912)		0.939
chr12	6696353	A	G	CHD4 (Intron)	CHD4:PHOS(0.459)PPI(0.912)		0.754
chr12	6691059	C	T	CHD4 (Intron)	CHD4:PHOS(0.459)PPI(0.912)		0.754
chr12	6690450	C	T	CHD4 (Intron)	CHD4:PHOS(0.459)PPI(0.912)		0.754
chr12	6679760	C	G	CHD4 (UTR)	CHD4:PHOS(0.459)PPI(0.912),NOP2:PPI(0.142)		1.551
chr11	125507534	G	C	CHEK1 (Intron)	CHEK1:PHOS(0.990)PPI(0.957)		0.970
chr22	29090987	G	A	CHEK2 (Intron)	CHEK2:PHOS(0.947)PPI(0.990)REG(0.409)		0.970
chr22	29092744	G	T	CHEK2 (Intron)	CHEK2:PHOS(0.947)PPI(0.990)REG(0.409)		0.970
chr22	29090111	G	T	CHEK2 (Intron)	CHEK2:PHOS(0.947)PPI(0.990)REG(0.409)		0.970
chr22	29095962	G	A	CHEK2 (Intron)	CHEK2:PHOS(0.947)PPI(0.990)REG(0.409)		0.970
chr4	54915108	A	G	CHIC2 (Intron)	CHIC2:PHOS(0.736)PPI(0.685)REG(0.409),FIP1L1:PHOS(0.928)PPI(0.817)REG(0.409)		0.979
chr4	54876362	C	A	CHIC2 (Intron)	CHIC2:PHOS(0.736)PPI(0.685)REG(0.409),FIP1L1:PHOS(0.928)PPI(0.817)REG(0.409)		0.794
chr2	175779926	T	G	CHN1 (Intron and Medial)	CHN1:PPI(0.792)		0.697
chr2	175783503	C	T	CHN1 (Intron)	CHN1:PPI(0.792)		1.134
chr2	175676341	C	A	CHN1 (Intron)	CHN1:PPI(0.792)		1.134
chr2	175742515	C	A	CHN1 (Intron)	CHN1:PPI(0.792)		1.134
chr2	175676814	T	G	CHN1 (Intron)	CHN1:PPI(0.792)		0.697
chr2	175816937	G	C	CHN1 (Intron)	CHN1:PPI(0.792)		0.513
chr2	175711875	G	C	CHN1 (Promoter and UTR)	CHN1:PPI(0.792)		1.308
chr15	90776984	G	C	CIB1 (Intron)	CIB1:PPI(0.729)	MOTIFBR=EGR1#Egr-1_disc6_8mer#90776974#90776985#+#11#0459191#0495042,MOTIFG=E2F_known4#90776980#90776986#-#2#6852#4988	3.330
chr16	10996399	C	A	CIITA (Intron)	CIITA:PPI(0.657)REG(0.929)		0.982
chr16	11023208	T	C	CIITA (UTR)	CIITA:PPI(0.657)REG(0.929),DEXI:PPI(0.236)REG(0.634)		1.604

chr14	102821981	G	C	CINP (Intron)	CINP:PPI(0.142)		0.674
chr3	184081510	G	C	CLCN2 (Promoter), EIF2B5 (Intron), POLR2H (Intron)	CLCN2:PPI(0.307),EIF2B5:PHOS(0.566)PPI(0.746)REG(0.761),POLR2H:PHOS(0.398)PPI(0.996)REG(0.634)	MOTIFBR=GATA3#GATA_known8_8mer#184081499#184081511#+#12#0000000#0142857	3.731
chr1	36204046	G	A	CLSPN (Intron)	CLSPN:PPI(0.781)REG(0.409)		0.494
chr1	36201913	G	C	CLSPN (Intron)	CLSPN:PPI(0.781)REG(0.409)		0.494
chr1	36202024	T	G	CLSPN (UTR)	CLSPN:PPI(0.781)REG(0.409)		2.116
chr1	36202024	T	G	CLSPN (UTR)	CLSPN:PPI(0.781)REG(0.409)		2.116
chr1	36202022	A	G	CLSPN (UTR)	CLSPN:PPI(0.781)REG(0.409)		1.494
chr1	36202022	A	G	CLSPN (UTR)	CLSPN:PPI(0.781)REG(0.409)		1.494
chr1	36202022	A	G	CLSPN (UTR)	CLSPN:PPI(0.781)REG(0.409)		1.494
chr1	36202022	A	G	CLSPN (UTR)	CLSPN:PPI(0.781)REG(0.409)		1.494
chr1	36202022	A	G	CLSPN (UTR)	CLSPN:PPI(0.781)REG(0.409)		1.494
chr17	57760936	G	C	CLTC (Intron and Promoter)	CLTC:PPI(0.983)REG(0.761),PTRH2:PHOS(0.698)PPI(0.761)REG(0.880)		1.756
chr17	57760912	C	T	CLTC (Intron and Promoter)	CLTC:PPI(0.983)REG(0.761),PTRH2:PHOS(0.698)PPI(0.761)REG(0.880)		1.756
chr17	57743688	G	C	CLTC (Intron)	CLTC:PPI(0.983)REG(0.761)		2.756
chr17	57743693	G	T	CLTC (Intron)	CLTC:PPI(0.983)REG(0.761)		2.134
chr17	57743693	G	T	CLTC (Intron)	CLTC:PPI(0.983)REG(0.761)		2.134
chr17	57744355	G	C	CLTC (Intron)	CLTC:PPI(0.983)REG(0.761)		0.948
chr17	57767882	C	A	CLTC (Intron)	CLTC:PPI(0.983)REG(0.761),PTRH2:PHOS(0.698)PPI(0.761)REG(0.880)		1.571
chr17	57758203	G	A	CLTC (Intron)	CLTC:PPI(0.983)REG(0.761),PTRH2:PHOS(0.698)PPI(0.761)REG(0.880)		0.948
chr17	57752241	C	G	CLTC (Intron)	CLTC:PPI(0.983)REG(0.761),PTRH2:PHOS(0.698)PPI(0.761)REG(0.880)		0.948
chr17	57771002	G	C	CLTC (Intron)	CLTC:PPI(0.983)REG(0.761),PTRH2:PHOS(0.698)PPI(0.761)REG(0.880)		0.948
chr17	57697449	C	T	CLTC (UTR)	CLTC:PPI(0.983)REG(0.761)		2.368
chr17	57771421	G	T	CLTC (UTR)	CLTC:PPI(0.983)REG(0.761)		1.571
chr9	17235801	T	C	CNTLN (Intron)	CNTLN:REG(0.634)		1.115
chr9	17134994	G	T	CNTLN (Promoter and UTR)	CNTLN:REG(0.634)		1.727
chr17	48275184	G	C	COL1A1 (Intron and Medial)	COL1A1:PPI(0.857)REG(0.634)		0.816
chr17	48264333	G	T	COL1A1 (Intron and Medial)	COL1A1:PPI(0.857)REG(0.634)		0.816

chr17	48266658	G	A	COL1A1 (Intron and Medial)	COL1A1:PPI(0.857)REG(0.634)		1.439
chr17	48275869	G	C	COL1A1 (Intron and Medial)	COL1A1:PPI(0.857)REG(0.634)		2.051
chr17	48274712	G	T	COL1A1 (Intron)	COL1A1:PPI(0.857)REG(0.634)		1.243
chr17	48265009	C	T	COL1A1 (Intron)	COL1A1:PPI(0.857)REG(0.634)		0.631
chr17	48266719	G	T	COL1A1 (Intron)	COL1A1:PPI(0.857)REG(0.634)		0.631
chr17	48273149	C	T	COL1A1 (Intron)	COL1A1:PPI(0.857)REG(0.634)		0.631
chr8	100904286	G	C	COX6C (Intron)	COX6C:PHOS(0.509)PPI(0.695)		0.374
chr19	17063015	G	A	CPAMD8 (Intron), TPM4 (Distal)	CPAMD8:PPI(0.307),TPM4:PPI(0.307)REG(0.830)		0.764
chr9	126129970	C	T	CRB2 (Intron and Promoter)	CRB2:PPI(0.142)REG(0.634)	MOTIFG=SREBP_known2#126129970#126129977#-#7#8589#4194	1.475
chr2	208425838	C	T	CREB1 (Intron)	CREB1:PHOS(0.276)PPI(0.941)REG(0.409)		1.636
chr16	3817982	T	G	CREBBP (Intron and Promoter)	CREBBP:PPI(0.998)		1.181
chr16	3827658	T	C	CREBBP (Intron)	CREBBP:PPI(0.998)		1.804
chr16	3811457	A	G	CREBBP (Intron)	CREBBP:PPI(0.998)		1.181
chr16	3786266	C	T	CREBBP (Intron)	CREBBP:PPI(0.998)		1.181
chr16	3817716	C	T	CREBBP (Intron)	CREBBP:PPI(0.998)		1.181
chr16	3827767	A	C	CREBBP (Intron)	CREBBP:PPI(0.998)		0.996
chr16	3827714	C	A	CREBBP (Intron)	CREBBP:PPI(0.998)		0.996
chr16	3807240	G	A	CREBBP (Intron)	CREBBP:PPI(0.998)		0.996
chrX	1327655	T	A	CRLF2 (Intron)	CRLF2:PPI(0.361)		0.924
chrX	1317417	A	T	CRLF2 (Intron)	CRLF2:PPI(0.361)		0.606
chr15	91163160	A	C	CRTC3 (Intron)	CRTC3:PPI(0.142)		1.248
chr15	91163160	A	C	CRTC3 (Intron)	CRTC3:PPI(0.142)		1.248
chr15	91163160	A	C	CRTC3 (Intron)	CRTC3:PPI(0.142)		1.248
chr15	91163160	A	C	CRTC3 (Intron)	CRTC3:PPI(0.142)		1.248
chr15	91163160	A	C	CRTC3 (Intron)	CRTC3:PPI(0.142)		1.248
chr15	91163160	A	C	CRTC3 (Intron)	CRTC3:PPI(0.142)		1.248
chr15	91163160	A	C	CRTC3 (Intron)	CRTC3:PPI(0.142)		1.248
chr15	91163160	A	C	CRTC3 (Intron)	CRTC3:PPI(0.142)		1.248
chr15	91163160	A	C	CRTC3 (Intron)	CRTC3:PPI(0.142)		1.248
chr15	91147799	C	A	CRTC3 (Intron)	CRTC3:PPI(0.142)		0.871
chr5	149453130	A	C	CSF1R (Intron)	CSF1R:PPI(0.718)		0.588
chr5	149457839	A	C	CSF1R (Intron)	CSF1R:PPI(0.718)		0.403
chr1	36940935	A	G	CSF3R (Intron)	CSF3R:PPI(0.938)REG(0.409)		1.005

chr1	36932767	T	G	CSF3R (Intron)	CSF3R:PPI(0.938)REG(0.409)		0.820
chr22	38757479	A	G	CSNK1E (UTR)	CSNK1E:PHOS(0.941)PPI(0.968)REG(0.409)		1.089
chr20	54970822	G	T	CSTF1 (Intron)	CSTF1:PPI(0.967)REG(0.967)		0.901
chrX	100078868	T	G	CSTF2 (Intron)	CSTF2:PHOS(0.459)PPI(0.979)		1.121
chrX	100083164	G	C	CSTF2 (Intron)	CSTF2:PHOS(0.459)PPI(0.979)		0.936
chr10	126727559	C	T	CTBP2 (Intron)	CTBP2:PPI(0.880)REG(0.992)		2.396
chr16	67645518	T	G	CTCF (Intron)	CTCF:PPI(0.774)REG(1.000)		1.810
chr20	56089496	C	A	CTCFL (Intron)	CTCFL:PPI(0.583)REG(0.992)	MOTIFBR=CTCF#CTCF_known1_8mer#56089479#56089498#+#18#0128855#0354626,RAD21#Rad21_disc1_8mer#56089480#56089500#+#17#0108000#0392000,CTCF#CTCF_disc1_8mer#56089481#56089503#-#7#0137097#0213710	2.752
chr17	29647327	A	C	CTD-2370N5.3 (Promoter), EVI2A (Intron), EVI2B (Medial)	EVI2B:REG(0.409),NF1:PPI(0.774)REG(0.409)		0.668
chr3	41265579	G	A	CTNNB1 (Intron)	CTNNB1:PPI(0.997)REG(0.409)		2.178
chr3	41265682	C	T	CTNNB1 (UTR)	CTNNB1:PPI(0.997)REG(0.409)		2.800
chr13	113907391	A	G	CUL4A (Intron)	CUL4A:PPI(0.822)REG(0.409)		0.564
chrX	119663851	C	G	CUL4B (Intron)	CUL4B:PPI(0.361)		0.320
chrX	119678184	G	A	CUL4B (Intron)	CUL4B:PPI(0.361)		0.312
chrX	119708301	C	A	CUL4B (Intron)	CUL4B:PPI(0.361)		0.127
chrX	70331219	G	A	CXorf65 (Medial)	IL2RG:PPI(0.943),MED12:PPI(0.835)		1.631
chrX	70329022	T	G	CXorf65 (Medial)	IL2RG:PPI(0.943),MED12:PPI(0.835)		1.019
chr16	50815383	C	G	CYLD (Intron)	CYLD:PPI(0.482)REG(0.409)		0.996
chr15	51508120	A	C	CYP19A1 (Intron and Promoter)	CYP19A1:PPI(0.236)		0.085
chr15	51520206	T	C	CYP19A1 (Intron)	CYP19A1:PPI(0.236)		0.270
chr15	51535147	C	A	CYP19A1 (UTR)	CYP19A1:PPI(0.236)		0.708
chr15	51535153	G	A	CYP19A1 (UTR)	CYP19A1:PPI(0.236)		0.708
chr9	90261372	C	G	DAPK1 (Intron)	DAPK1:PPI(0.671)		0.346
chr9	90313528	T	G	DAPK1 (Intron)	DAPK1:PPI(0.671)		0.346
chr9	90263947	G	C	DAPK1 (Intron)	DAPK1:PPI(0.671)		0.346
chr9	90252781	C	T	DAPK1 (Intron)	DAPK1:PPI(0.671)		0.346
chr6	117862290	T	C	DCBLD1 (Intron and Medial)	DCBLD1:PPI(0.142),GOPC:PPI(0.866)REG(0.409)		0.835

chr6	117825230	C	T	DCBLD1 (Intron), GOPC (Intron)	DCBLD1:PPI(0.142),GOPC:PPI(0.866)REG(0.409)		1.273
chr1	114450578	G	T	DCLRE1B (Intron)	DCLRE1B:PHOS(0.276)PPI(0.142)REG(0.944)		1.021
chr10	14974843	G	A	DCLRE1C (Intron)	DCLRE1C:PPI(0.515)REG(0.634)		0.307
chr10	14950229	G	A	DCLRE1C (UTR)	DCLRE1C:PPI(0.515)REG(0.634)		0.307
chr11	61068139	C	T	DDB1 (Intron)	DDB1:PHOS(0.540)PPI(0.921)		1.399
chr11	61097627	C	A	DDB1 (Intron)	DDB1:PHOS(0.540)PPI(0.921)		0.961
chr11	61090036	G	T	DDB1 (Promoter and UTR)	DDB1:PHOS(0.540)PPI(0.921)		1.584
chr11	61070652	G	C	DDB1 (Promoter and UTR)	DDB1:PHOS(0.540)PPI(0.921)		1.382
chr11	61089964	G	T	DDB1 (Promoter and UTR)	DDB1:PHOS(0.540)PPI(0.921)		0.961
chr1	162722987	G	A	DDR2 (Intron)	DDR2:PHOS(0.828)PPI(0.515)		1.995
chr1	162742095	C	T	DDR2 (UTR)	DDR2:PHOS(0.828)PPI(0.515)		0.575
chr2	15732292	C	G	DDX1 (Intron)	DDX1:PPI(0.515)REG(0.634)	MOTIFBR=NFKB1#NF-kappaB_known4_8mer#15732280#15732294#-#2#0000000#0615385	2.089
chr2	15735419	G	A	DDX1 (Intron)	DDX1:PPI(0.515)REG(0.634)		0.492
chr2	15768876	G	A	DDX1 (Intron)	DDX1:PPI(0.515)REG(0.634)		0.307
chr2	15747442	A	C	DDX1 (Intron)	DDX1:PPI(0.515)REG(0.634)		0.307
chr2	15761418	A	G	DDX1 (Intron)	DDX1:PPI(0.515)REG(0.634)		0.307
chr11	108550439	A	C	DDX10 (Intron)	DDX10:PHOS(0.509)PPI(0.917)REG(0.634)		1.389
chr11	108564404	G	A	DDX10 (Intron)	DDX10:PHOS(0.509)PPI(0.917)REG(0.634)		0.951
chr6	18225877	C	A	DEK (UTR)	DEK:PPI(0.540)REG(0.634)		1.115
chr22	42018233	C	T	DESI1 (Promoter), XRCC6 (Intron)	XRCC6:PHOS(0.276)PPI(0.993)		1.165
chr5	79952419	G	A	DHFR (Promoter), MSH3 (Intron)	DHFR:PPI(0.641)REG(0.830),MSH3:PPI(0.850)REG(0.830)		1.414
chr17	41609932	T	G	DHX8 (Intron), ETV4 (Intron and Promoter)	DHX8:PPI(0.761)REG(0.952),ETV4:PHOS(0.693)PPI(0.361)REG(0.952)		0.858
chr1	182825720	G	T	DHX9 (Intron)	DHX9:PPI(0.972)REG(0.409)		1.538
chr1	182850800	C	A	DHX9 (Intron)	DHX9:PPI(0.972)REG(0.409)		1.538
chr1	182812683	G	A	DHX9 (Intron)	DHX9:PPI(0.972)REG(0.409)		0.915
chr1	182811957	C	T	DHX9 (Intron)	DHX9:PPI(0.972)REG(0.409)		0.915
chr1	182823369	C	T	DHX9 (Intron)	DHX9:PPI(0.972)REG(0.409)		0.915
chr1	182846145	A	G	DHX9 (Intron)	DHX9:PPI(0.972)REG(0.409)		0.915
chr14	95583040	A	C	DICER1 (Intron)	DICER1:PPI(0.685)REG(0.409)		0.985
chr14	95583936	A	G	DICER1 (Intron)	DICER1:PPI(0.685)REG(0.409)		0.547

chr14	95556760	T	C	DICER1 (UTR)	DICER1:PPI(0.685)REG(0.409)		0.362
chr22	38948498	C	T	DMC1 (Intron)	DMC1:PPI(0.787)		0.740
chr19	46274972	A	C	DMPK (Intron), SIX5 (Promoter)	DMPK:PHOS(0.769)PPI(0.657),SIX5:PHOS(0.276)PPI(0.408)REG(0.997)		1.615
chr10	70174883	G	C	DNA2 (Intron)	DNA2:REG(0.409)		0.771
chr10	70178611	G	A	DNA2 (Intron)	DNA2:REG(0.409)		0.148
chr10	70181888	G	C	DNA2 (Intron)	DNA2:REG(0.409)		0.148
chr1	78444801	C	T	DNAJB4 (Promoter), FUBP1 (Promoter)	DNAJB4:PPI(0.761)REG(0.944),FUBP1:PHOS(0.585)PPI(0.739)REG(0.761)		2.256
chr19	10935731	A	T	DNM2 (Intron)	DNM2:PHOS(0.398)PPI(0.930)REG(0.959)		1.500
chr19	10909393	T	C	DNM2 (Intron)	DNM2:PHOS(0.398)PPI(0.930)REG(0.959)		0.878
chr19	10870408	T	G	DNM2 (Intron)	DNM2:PHOS(0.398)PPI(0.930)REG(0.959),ICAM3:PPI(0.540)REG(0.634),ILF3:PHOS(0.276)PPI(0.781)REG(0.952),P2RY11:REG(0.909)		2.298
chr19	10265457	G	C	DNMT1 (Intron)	DNMT1:PPI(0.846)REG(0.761)		0.794
chr19	10260685	G	A	DNMT1 (Intron)	DNMT1:PPI(0.846)REG(0.761)		0.609
chr19	10273880	T	G	DNMT1 (Intron)	DNMT1:PPI(0.846)REG(0.761)		0.609
chr19	10267193	G	A	DNMT1 (Intron)	DNMT1:PPI(0.846)REG(0.761)		0.609
chr2	25471147	A	C	DNMT3A (Intron and Medial)	DNMT3A:PHOS(0.661)PPI(0.746)REG(0.761)		1.883
chr2	25470718	G	A	DNMT3A (Intron)	DNMT3A:PHOS(0.661)PPI(0.746)REG(0.761)		1.271
chr2	25498483	A	C	DNMT3A (Intron)	DNMT3A:PHOS(0.661)PPI(0.746)REG(0.761)		1.260
chr2	25472324	G	T	DNMT3A (Intron)	DNMT3A:PHOS(0.661)PPI(0.746)REG(0.761)		0.648
chr2	25458737	A	C	DNMT3A (Intron)	DNMT3A:PHOS(0.661)PPI(0.746)REG(0.761)		0.463
chr2	25469913	C	T	DNMT3A (Intron)	DNMT3A:PHOS(0.661)PPI(0.746)REG(0.761)		0.463
chr6	83872644	G	A	DOPEY1 (Intron), PGM3 (Intron)	DOPEY1:REG(0.634),PGM3:PPI(0.606)		0.307
chr19	2225246	T	G	DOT1L (Intron and Promoter)	DOT1L:PPI(0.307)REG(0.761)		0.648
chr19	2180578	T	G	DOT1L (Intron)	DOT1L:PPI(0.307)REG(0.761)		0.463
chr19	2202670	T	C	DOT1L (Intron)	DOT1L:PPI(0.307)REG(0.761)		0.463
chr10	103345020	C	A	DPCD (Intron), POLL (Intron and Promoter)	DPCD:PPI(0.142)REG(0.634),POLL:PHOS(0.566)PPI(0.707)REG(0.634)		1.197

chr1	212236296	T	C	DTL (Intron)	DTL:PPI(0.408)REG(0.909)		0.932
chr1	212240254	G	A	DTL (Intron)	DTL:PPI(0.408)REG(0.909)		0.747
chr1	212209203	C	A	DTL (UTR)	DTL:PPI(0.408)REG(0.909),INTS7:REG(0.880)		2.513
chr2	11598235	G	A	E2F6 (Intron)	E2F6:PPI(0.583)REG(0.996)		1.797
chr5	158522624	T	G	EBF1 (Intron)	EBF1:PHOS(0.398)PPI(0.822)REG(0.996)		2.409
chr5	158500570	G	C	EBF1 (Intron)	EBF1:PHOS(0.398)PPI(0.822)REG(0.996)		1.612
chr5	158500589	C	T	EBF1 (Intron)	EBF1:PHOS(0.398)PPI(0.822)REG(0.996)		1.612
chr5	158422006	C	A	EBF1 (Intron)	EBF1:PHOS(0.398)PPI(0.822)REG(0.996)		0.989
chr6	139165717	G	A	ECT2L (Intron)			0.811
chr6	139222082	C	T	ECT2L (Intron)			0.626
chr6	139186404	A	C	ECT2L (Intron)			0.626
chr6	139167490	G	T	ECT2L (Intron)			0.188
chr6	139143798	G	A	ECT2L (Intron)			0.003
chr6	139167820	G	A	ECT2L (Intron)			0.003
chr7	36324436	G	A	EEPD1 (Intron)			0.188
chr7	36336983	G	A	EEPD1 (Intron)			0.188
chr7	55224355	G	A	EGFR (Intron)	EGFR:PHOS(0.743)PPI(0.999)		1.807
chr7	55210952	G	C	EGFR (Intron)	EGFR:PHOS(0.743)PPI(0.999)		1.622
chr7	55214295	G	A	EGFR (Intron)	EGFR:PHOS(0.743)PPI(0.999)		1.622
chr7	55239086	C	G	EGFR (Intron)	EGFR:PHOS(0.743)PPI(0.999)		1.184
chr7	55236202	C	T	EGFR (Intron)	EGFR:PHOS(0.743)PPI(0.999)		0.999
chr7	55238412	C	T	EGFR (UTR)	EGFR:PHOS(0.743)PPI(0.999)		0.999
chr12	124114672	G	A	EIF2B1 (Intron), GTF2H3 (Medial)	EIF2B1:PHOS(0.398)PPI(0.910)REG(0.830),GTF2H3:PHOS(0.276)PPI(0.937)REG(0.830)		1.003
chr12	124118273	C	T	EIF2B1 (Promoter and UTR), GTF2H3 (Promoter)	EIF2B1:PHOS(0.398)PPI(0.910)REG(0.830),GTF2H3:PHOS(0.276)PPI(0.937)REG(0.830)	MOTIFG=Ets_known10#124118272#124118278#-#5#5743#1893	2.595
chr3	186505676	G	C	EIF4A2 (Intron)	EIF4A2:PHOS(0.931)PPI(0.927)REG(0.944)		2.644
chr3	186501241	T	G	EIF4A2 (Promoter and UTR)	EIF4A2:PHOS(0.931)PPI(0.927)REG(0.944)		3.602
chr13	41515512	C	G	ELF1 (Intron)	ELF1:PHOS(0.276)PPI(0.606)REG(0.999)		1.622

chr13	41517081	C	T	ELF1 (Intron)	ELF1:PHOS(0.276)PPI(0.606)REG(0.999)		1.622
chr13	41525416	A	G	ELF1 (Intron)	ELF1:PHOS(0.276)PPI(0.606)REG(0.999)		1.051
chr13	41636948	A	G	ELF1 (Promoter)	ELF1:PHOS(0.276)PPI(0.606)REG(0.999),WBP4:PPI(0.872)REG(0.634)		2.807
chr13	41635808	T	C	ELF1 (Promoter)	ELF1:PHOS(0.276)PPI(0.606)REG(0.999),WBP4:PPI(0.872)REG(0.634)		3.419
chrX	129206511	G	T	ELF4 (Intron)	ELF4:PPI(0.408)		0.333
chrX	129215382	A	C	ELF4 (UTR)	ELF4:PPI(0.408)		0.148
chr19	18557627	A	T	ELL (Intron)	ELL:PHOS(0.276)PPI(0.872)REG(0.409)		0.848
chr17	48456042	G	C	EME1 (Intron)	EME1:PPI(0.236)REG(0.830),LRRC59:PPI(0.739)REG(0.761)		1.201
chr17	48458411	G	A	EME1 (UTR)	EME1:PPI(0.236)REG(0.830)		2.376
chr2	42491751	C	T	EML4 (Intron)	EML4:PPI(0.307)		0.915
chr2	42396871	C	T	EML4 (Intron)	EML4:PPI(0.307)		0.904
chr2	42483626	C	G	EML4 (Intron)	EML4:PPI(0.307)		0.729
chr2	42511903	A	G	EML4 (Intron)	EML4:PPI(0.307)		0.729
chr2	42511627	T	G	EML4 (Intron)	EML4:PPI(0.307)		0.729
chr2	42515615	G	A	EML4 (Intron)	EML4:PPI(0.307)		0.729
chr2	42556022	A	T	EML4 (Intron)	EML4:PPI(0.307)		0.292
chr2	42522756	A	G	EML4 (Intron)	EML4:PPI(0.307)		0.107
chr2	42469875	G	A	EML4 (Intron)	EML4:PPI(0.307)		0.107
chr2	42531760	C	G	EML4 (Intron)	EML4:PPI(0.307)		0.107
chr22	41513085	A	G	EP300 (Intron)	EP300:PPI(0.998)REG(0.999)		1.184
chr22	41548393	G	T	EP300 (Intron)	EP300:PPI(0.998)REG(0.999)		0.999
chr22	41568480	T	C	EP300 (Intron)	EP300:PPI(0.998)REG(0.999)		0.999
chr22	41527343	T	G	EP300 (Intron)	EP300:PPI(0.998)REG(0.999)		0.999
chr22	41560254	C	A	EP300 (Intron)	EP300:PPI(0.998)REG(0.999)		0.999
chr22	41532025	A	G	EP300 (Intron)	EP300:PPI(0.998)REG(0.999)		0.999
chr22	41566595	C	T	EP300 (Intron)	EP300:PPI(0.998)REG(0.999)		0.999
chr22	41568498	C	T	EP300 (Intron)	EP300:PPI(0.998)REG(0.999)		0.999

chr22	41488397	G	A	EP300 (UTR)	EP300:PPI(0.998)REG(0.999)	MOTIFBR=ELF1#ELF1_disc2_8mer#41488389#41488399#+#9#0085843#0243976,YY1#YY1_disc3_8mer#41488389#41488399#+#9#0084218#0424403,TAF1#TATA_disc10_8mer#41488391#41488401#-#4#0061111#0361667,TAF7#TATA_disc10_8mer#41488391#41488401#-#4#0061111#0361667,TBP#TATA_disc10_8mer#41488391#41488401#-#4#0061111#0361667,EP300#p300_disc8_8mer#41488391#41488401#-#4#0014286#0085714,E2F4#E2F_disc8_8mer#41488393#41488404#+#5#0085332#0379473,E2F6#E2F_disc8_8mer#41488393#41488404#+#5#0085332#0379473	2.978
chr22	41488927	T	G	EP300 (UTR)	EP300:PPI(0.998)REG(0.999)		2.184
chr3	89390877	C	T	EPHA3 (Intron)	EPHA3:PHOS(0.918)PPI(0.540)		1.391
chr3	89259798	C	A	EPHA3 (Intron)	EPHA3:PHOS(0.918)PPI(0.540)		1.391
chr3	89176423	G	T	EPHA3 (Intron)	EPHA3:PHOS(0.918)PPI(0.540)		1.391
chr3	89456555	T	A	EPHA3 (Intron)	EPHA3:PHOS(0.918)PPI(0.540)		1.391
chr4	66279932	T	G	EPHA5 (Intron)	EPHA5:PPI(0.448)		0.791
chr4	66201987	G	A	EPHA5 (Intron)	EPHA5:PPI(0.448)		0.791
chr4	66439485	T	C	EPHA5 (Intron)	EPHA5:PPI(0.448)		0.353
chr4	66213968	G	A	EPHA5 (Intron)	EPHA5:PPI(0.448)		0.168
chr3	97311586	G	T	EPHA6 (Intron)	EPHA6:PPI(0.236)		0.708
chr3	97198102	C	A	EPHA6 (Intron)	EPHA6:PPI(0.236)		0.672
chr6	94067928	G	A	EPHA7 (Intron)	EPHA7:PHOS(0.756)PPI(0.872)		1.285
chr6	93956746	C	T	EPHA7 (Intron)	EPHA7:PHOS(0.756)PPI(0.872)		1.285
chr6	93956440	T	G	EPHA7 (Intron)	EPHA7:PHOS(0.756)PPI(0.872)		0.662
chr6	93967401	T	C	EPHA7 (Intron)	EPHA7:PHOS(0.756)PPI(0.872)		0.662
chr6	94068134	G	A	EPHA7 (Intron)	EPHA7:PHOS(0.756)PPI(0.872)		0.662
chr6	93979124	C	T	EPHA7 (Intron)	EPHA7:PHOS(0.756)PPI(0.872)		0.662

chr3	134344092	C	G	EPHB1 (Intron)	EPHB1:PHOS(0.722)PPI(0.657)REG(0.634),KY:PPI(0.540)		0.408
chr7	100410366	C	A	EPHB4 (Intron)	EPHB4:PHOS(0.709)PPI(0.307)REG(0.409)		1.014
chr7	100414793	C	T	EPHB4 (Intron)	EPHB4:PHOS(0.709)PPI(0.307)REG(0.409)		0.391
chr1	51831570	T	C	EPS15 (Intron)	EPS15:PPI(0.919)REG(0.830)		1.579
chr1	51864616	G	T	EPS15 (Intron)	EPS15:PPI(0.919)REG(0.830)		0.771
chr1	51926538	A	T	EPS15 (Intron)	EPS15:PPI(0.919)REG(0.830)		0.771
chr1	51926948	A	C	EPS15 (Intron)	EPS15:PPI(0.919)REG(0.830)		0.771
chr1	51869004	T	C	EPS15 (Intron)	EPS15:PPI(0.919)REG(0.830)		0.771
chr1	51926978	C	G	EPS15 (Intron)	EPS15:PPI(0.919)REG(0.830)		0.771
chr1	51931089	C	T	EPS15 (Intron)	EPS15:PPI(0.919)REG(0.830)		0.771
chr17	37871614	T	G	ERBB2 (Intron and Promoter)	ERBB2:PPI(0.977)REG(0.761)	MOTIFG=ZEB1_known1#37871609#37871615#-#1#6794#3667	3.715
chr17	37871919	G	A	ERBB2 (Intron and Promoter)	ERBB2:PPI(0.977)REG(0.761)		2.738
chr17	37866568	G	T	ERBB2 (Intron and Promoter)	ERBB2:PPI(0.977)REG(0.761)		1.738
chr17	37871432	G	C	ERBB2 (Intron and Promoter)	ERBB2:PPI(0.977)REG(0.761)		1.553
chr17	37880335	C	G	ERBB2 (Intron)	ERBB2:PPI(0.977)REG(0.761)		3.039
chr17	37879768	G	A	ERBB2 (Intron)	ERBB2:PPI(0.977)REG(0.761)		2.738
chr17	37880102	C	A	ERBB2 (Intron)	ERBB2:PPI(0.977)REG(0.761)		2.727
chr17	37883062	C	T	ERBB2 (Intron)	ERBB2:PPI(0.977)REG(0.761)		1.738
chr17	37882147	G	A	ERBB2 (Intron)	ERBB2:PPI(0.977)REG(0.761)		1.115
chr17	37868370	A	C	ERBB2 (Intron)	ERBB2:PPI(0.977)REG(0.761)		0.930
chr17	37883381	A	C	ERBB2 (Intron)	ERBB2:PPI(0.977)REG(0.761)		0.930
chr17	37880843	C	T	ERBB2 (Intron)	ERBB2:PPI(0.977)REG(0.761)		0.930
chr12	56486609	G	T	ERBB3 (Intron and Promoter)	ERBB3:PPI(0.959)REG(0.409)		1.500
chr12	56482901	C	T	ERBB3 (Intron)	ERBB3:PPI(0.959)REG(0.409)		0.878
chr12	56492748	T	C	ERBB3 (Intron)	ERBB3:PPI(0.959)REG(0.409)		0.878
chr12	56496079	C	T	ERBB3 (UTR)	ERBB3:PPI(0.959)REG(0.409),PA2G4:PPI(0.792)REG(0.409)		0.878
chr2	212615430	C	A	ERBB4 (Intron)	ERBB4:PPI(0.866)		1.458
chr2	212589798	T	A	ERBB4 (Intron)	ERBB4:PPI(0.866)		1.273
chr2	212288745	G	A	ERBB4 (Intron)	ERBB4:PPI(0.866)		0.650
chr2	212484074	G	C	ERBB4 (Intron)	ERBB4:PPI(0.866)		0.650

chr2	212295632	A	C	ERBB4 (Intron)	ERBB4:PPI(0.866)		0.650
chr2	212495140	C	G	ERBB4 (Intron)	ERBB4:PPI(0.866)		0.650
chr2	212578160	G	A	ERBB4 (Intron)	ERBB4:PPI(0.866)		0.650
chr2	212355500	T	C	ERBB4 (Intron)	ERBB4:PPI(0.866)		0.650
chr2	212248171	C	G	ERBB4 (UTR)	ERBB4:PPI(0.866)		1.273
chr2	212243422	G	A	ERBB4 (UTR)	ERBB4:PPI(0.866)		0.835
chr19	45918222	G	C	ERCC1 (Intron)	ERCC1:PPI(0.902)REG(0.830)		1.353
chr19	45860637	G	A	ERCC2 (Intron)	ERCC2:PPI(0.979)REG(0.761)		1.744
chr19	45859051	A	C	ERCC2 (Intron)	ERCC2:PPI(0.979)REG(0.761)		1.186
chr19	45856420	T	G	ERCC2 (Intron)	ERCC2:PPI(0.979)REG(0.761)		0.936
chr2	128046536	C	G	ERCC3 (Intron)	ERCC3:PHOS(0.276)PPI(0.946)		0.841
chr16	14027963	C	T	ERCC4 (Intron)	ERCC4:PPI(0.967)REG(0.880)		0.901
chr13	103525876	G	T	ERCC5 (Intron)	ERCC5:PPI(0.822)REG(0.409)		0.564
chr10	50678066	G	A	ERCC6 (Intron)	ERCC6:PPI(0.236)REG(0.634)		0.307
chr10	50668398	C	G	ERCC6 (Intron)	ERCC6:PPI(0.236)REG(0.634)		0.307
chr10	50682415	C	A	ERCC6 (Intron)	ERCC6:PPI(0.236)REG(0.634)		0.307
chr10	50681157	A	G	ERCC6 (Intron)	ERCC6:PPI(0.236)REG(0.634)		0.307
chr10	50732034	G	C	ERCC6 (Intron)	ERCC6:PPI(0.236)REG(0.634),PGBD3:PPI(0.938) REG(0.409)		2.817
chr10	50732075	C	T	ERCC6 (Intron)	ERCC6:PPI(0.236)REG(0.634),PGBD3:PPI(0.938) REG(0.409)		2.817
chr10	50736350	A	C	ERCC6 (Intron)	ERCC6:PPI(0.236)REG(0.634),PGBD3:PPI(0.938) REG(0.409)		1.443
chr10	50666482	G	C	ERCC6 (UTR)	ERCC6:PPI(0.236)REG(0.634)		0.307
chr5	60241020	G	A	ERCC8 (Promoter)	ERCC8:PHOS(0.509)PPI(0.804)		1.329
chr21	39870183	T	G	ERG (Intron and Medial)	ERG:PPI(0.540)REG(0.634)		1.115
chr18	19146237	C	A	ESCO1 (Intron)	ESCO1:PPI(0.761)REG(0.634)		1.085
chr8	27633752	A	C	ESCO2 (Intron)	ESCO2:REG(0.634)		0.307
chr11	64067987	C	G	ESRRA (Medial)	ESRRA:PPI(0.774)REG(0.988)		1.149
chr11	128443047	A	T	ETS1 (Intron)	ETS1:PHOS(0.922)PPI(0.835)REG(0.993)		1.763
chr11	128443046	G	T	ETS1 (Intron)	ETS1:PHOS(0.922)PPI(0.835)REG(0.993)		1.165
chr7	13950939	G	A	ETV1 (Intron)	ETV1:PHOS(0.276)PPI(0.583)		1.068
chr7	13933933	A	T	ETV1 (UTR)	ETV1:PHOS(0.276)PPI(0.583)		0.260
chr12	11905307	G	A	ETV6 (Intron and Promoter)	ETV6:PPI(0.685)REG(0.409)		0.362
chr12	11803047	C	G	ETV6 (UTR)	ETV6:PPI(0.685)REG(0.409)		1.782

chr6	36355343	C	A	ETV7 (Medial and UTR)	ETV7:PHOS(0.868)PPI(0.781)REG(0.409)	MOTIFG=SMC3_disc4#36355337#36355345#+#7#8918#6195	2.425
chr17	29654935	A	C	EVI2A (Medial)	NF1:PPI(0.774)REG(0.409)		0.668
chr22	29693681	C	G	EWSR1 (Intron)	EWSR1:PPI(0.990)REG(0.409)		0.970
chr22	29683215	G	A	EWSR1 (Intron)	EWSR1:PPI(0.990)REG(0.409)		0.970
chr22	29694713	C	T	EWSR1 (Intron)	EWSR1:PPI(0.990)REG(0.409)		0.970
chr22	29682701	A	T	EWSR1 (Intron)	EWSR1:PPI(0.990)REG(0.409)		0.970
chr22	29693705	G	C	EWSR1 (Intron)	EWSR1:PPI(0.990)REG(0.409)		0.970
chr22	29694406	G	C	EWSR1 (Intron)	EWSR1:PPI(0.990)REG(0.409)		0.970
chr22	29694575	G	A	EWSR1 (Intron)	EWSR1:PPI(0.990)REG(0.409)		0.970
chr22	29669908	G	A	EWSR1 (Intron)	EWSR1:PPI(0.990)REG(0.409),RHBDD3:REG(0.409)		1.155
chr1	242042032	C	T	EXO1 (Intron and Promoter)	EXO1:PPI(0.850)REG(0.409)		0.617
chr1	242030128	C	T	EXO1 (Intron)	EXO1:PPI(0.850)REG(0.409)		1.240
chr1	242013671	C	G	EXO1 (Intron)	EXO1:PPI(0.850)REG(0.409)		1.240
chr1	242024245	T	A	EXO1 (Intron)	EXO1:PPI(0.850)REG(0.409)		1.240
chr1	242020495	C	T	EXO1 (Intron)	EXO1:PPI(0.850)REG(0.409)		1.239
chr1	242015820	A	G	EXO1 (Intron)	EXO1:PPI(0.850)REG(0.409)		0.802
chr1	242013952	C	G	EXO1 (Intron)	EXO1:PPI(0.850)REG(0.409)		0.617
chr8	118830770	C	G	EXT1 (Intron)	EXT1:PPI(0.448)REG(0.409)		0.791
chr8	118842589	C	G	EXT1 (Intron)	EXT1:PPI(0.448)REG(0.409)		0.791
chr8	118830646	C	T	EXT1 (Intron)	EXT1:PPI(0.448)REG(0.409)		0.168
chr11	44255863	C	T	EXT2 (Intron)	EXT2:PPI(0.729)REG(0.409)		1.040
chr11	44254137	G	T	EXT2 (Intron)	EXT2:PPI(0.729)REG(0.409)		0.417
chr8	72229769	C	A	EYA1 (Intron)	EYA1:PHOS(0.276)PPI(0.875)		0.669
chr20	45618809	A	C	EYA2 (Intron and Medial)	EYA2:PHOS(0.566)PPI(0.482)		1.044
chr20	45771831	A	C	EYA2 (Intron)	EYA2:PHOS(0.566)PPI(0.482)		1.054
chr20	45630115	G	C	EYA2 (Intron)	EYA2:PHOS(0.566)PPI(0.482)		0.869
chr20	45809592	G	A	EYA2 (Intron)	EYA2:PHOS(0.566)PPI(0.482)		0.432
chr20	45812051	A	C	EYA2 (Intron)	EYA2:PHOS(0.566)PPI(0.482)		0.432
chr20	45812085	A	C	EYA2 (Intron)	EYA2:PHOS(0.566)PPI(0.482)		0.432
chr20	45701033	G	T	EYA2 (Intron)	EYA2:PHOS(0.566)PPI(0.482)		0.246
chr6	133833918	G	T	EYA4 (Intron)	EYA4:PPI(0.236)		0.893
chr6	159205668	G	A	EZR (Intron)	EZR:PPI(0.606)		1.088
chr6	159205632	C	T	EZR (Intron)	EZR:PPI(0.606)		0.466
chr6	159204426	T	A	EZR (Intron)	EZR:PPI(0.606)		0.466
chr6	159206251	T	C	EZR (Intron)	EZR:PPI(0.606)		0.280
chr6	159239157	G	A	EZR (UTR)	EZR:PPI(0.606)		1.078

chr5	156590805	G	T	FAM71B (Intron), ITK (Intron)	ITK:PPI(0.826)		0.571
chr15	31217935	T	G	FAN1 (Intron)			1.003
chr15	31217935	T	G	FAN1 (Intron)			1.003
chr15	31221344	T	A	FAN1 (Intron)			0.199
chr15	31220920	A	C	FAN1 (Intron)			0.003
chr15	31235005	G	C	FAN1 (UTR)			0.003
chr15	31235086	G	A	FAN1 (UTR)			0.003
chr16	89869761	T	C	FANCA (Intron)	FANCA:PHOS(0.276)PPI(0.916)REG(0.634)		2.572
chr16	89869761	T	C	FANCA (Intron)	FANCA:PHOS(0.276)PPI(0.916)REG(0.634)		2.572
chr16	89838078	A	G	FANCA (Intron)	FANCA:PHOS(0.276)PPI(0.916)REG(0.634)		1.949
chr16	89838078	A	G	FANCA (Intron)	FANCA:PHOS(0.276)PPI(0.916)REG(0.634)		1.949
chr16	89849583	C	T	FANCA (Intron)	FANCA:PHOS(0.276)PPI(0.916)REG(0.634)		1.949
chr16	89849629	T	A	FANCA (Intron)	FANCA:PHOS(0.276)PPI(0.916)REG(0.634)		1.949
chr16	89874628	C	T	FANCA (Intron)	FANCA:PHOS(0.276)PPI(0.916)REG(0.634)		1.764
chr16	89874628	C	T	FANCA (Intron)	FANCA:PHOS(0.276)PPI(0.916)REG(0.634)		1.764
chr16	89818491	T	C	FANCA (Intron)	FANCA:PHOS(0.276)PPI(0.916)REG(0.634)		1.386
chr16	89869544	C	T	FANCA (Intron)	FANCA:PHOS(0.276)PPI(0.916)REG(0.634)		0.949
chr16	89836865	A	G	FANCA (Intron)	FANCA:PHOS(0.276)PPI(0.916)REG(0.634)		0.949
chr16	89818775	A	C	FANCA (Intron)	FANCA:PHOS(0.276)PPI(0.916)REG(0.634)		0.949
chr16	89842090	C	T	FANCA (Intron)	FANCA:PHOS(0.276)PPI(0.916)REG(0.634)		0.949
chr16	89862441	G	A	FANCA (Intron)	FANCA:PHOS(0.276)PPI(0.916)REG(0.634)		0.949
chr16	89871680	G	A	FANCA (Intron)	FANCA:PHOS(0.276)PPI(0.916)REG(0.634)		0.949
chr16	89857964	T	C	FANCA (Intron)	FANCA:PHOS(0.276)PPI(0.916)REG(0.634)		0.949

chr16	89858505	T	C	FANCA (Intron)	FANCA:PHOS(0.276)PPI(0.916)REG(0.634)		0.949
chr16	89858024	A	G	FANCA (Intron)	FANCA:PHOS(0.276)PPI(0.916)REG(0.634)		0.764
chr16	89845194	A	G	FANCA (Intron)	FANCA:PHOS(0.276)PPI(0.916)REG(0.634)		0.764
chr16	89865242	T	C	FANCA (UTR)	FANCA:PHOS(0.276)PPI(0.916)REG(0.634)		0.949
chr16	89804330	A	C	FANCA (UTR)	FANCA:PHOS(0.276)PPI(0.916)REG(0.634),ZNF276:PPI(0.408)REG(0.409)		1.949
chrX	14891661	T	G	FANCB (Promoter)	FANCB:PPI(0.408)REG(0.409),MOSPD2:PPI(0.540)		0.412
chr3	10085280	A	G	FANCD2 (Intron)	FANCD2:PPI(0.746)REG(0.880)		1.784
chr3	10085280	A	G	FANCD2 (Intron)	FANCD2:PPI(0.746)REG(0.880)		1.784
chr3	10105319	G	A	FANCD2 (Intron)	FANCD2:PPI(0.746)REG(0.880)		1.680
chr3	10105319	G	A	FANCD2 (Intron)	FANCD2:PPI(0.746)REG(0.880)		1.680
chr3	10105319	G	A	FANCD2 (Intron)	FANCD2:PPI(0.746)REG(0.880)		1.680
chr3	10105319	G	A	FANCD2 (Intron)	FANCD2:PPI(0.746)REG(0.880)		1.680
chr3	10105319	G	A	FANCD2 (Intron)	FANCD2:PPI(0.746)REG(0.880)		1.680
chr3	10105319	G	A	FANCD2 (Intron)	FANCD2:PPI(0.746)REG(0.880)		1.680
chr3	10105319	G	A	FANCD2 (Intron)	FANCD2:PPI(0.746)REG(0.880)		1.680
chr3	10105319	G	A	FANCD2 (Intron)	FANCD2:PPI(0.746)REG(0.880)		1.680
chr3	10070484	C	G	FANCD2 (Intron)	FANCD2:PPI(0.746)REG(0.880)		1.477
chr3	10084149	G	A	FANCD2 (Intron)	FANCD2:PPI(0.746)REG(0.880)		1.303
chr3	10106195	A	C	FANCD2 (Intron)	FANCD2:PPI(0.746)REG(0.880)		0.865
chr3	10089597	C	G	FANCD2 (Intron)	FANCD2:PPI(0.746)REG(0.880)		0.865
chr3	10114510	T	A	FANCD2 (Intron)	FANCD2:PPI(0.746)REG(0.880)		0.865
chr3	10106408	C	T	FANCD2 (Intron)	FANCD2:PPI(0.746)REG(0.880)		0.865
chr3	10091028	A	G	FANCD2 (Intron)	FANCD2:PPI(0.746)REG(0.880)		0.680
chr3	10085467	C	T	FANCD2 (Intron)	FANCD2:PPI(0.746)REG(0.880)		0.680
chr3	10076975	A	C	FANCD2 (Intron)	FANCD2:PPI(0.746)REG(0.880)		0.680
chr3	10104032	C	T	FANCD2 (Intron)	FANCD2:PPI(0.746)REG(0.880)		0.680
chr3	10114327	A	G	FANCD2 (Intron)	FANCD2:PPI(0.746)REG(0.880)		0.680
chr3	10091239	G	T	FANCD2 (Intron)	FANCD2:PPI(0.746)REG(0.880)		0.680
chr9	35079415	G	T	FANCG (Intron)	FANCG:PPI(0.583)REG(0.409)		1.680
chr9	35078737	G	C	FANCG (Intron)	FANCG:PPI(0.583)REG(0.409)		0.883
chr9	35074917	T	C	FANCG (Intron)	FANCG:PPI(0.583)REG(0.409),VCP:PPI(0.961)REG(0.634)	MOTIFG=GATA_known5#35074914#35074919#-#2#4839#0542	2.674

chr9	35074770	G	C	FANCG (Intron)	FANCG:PPI(0.583)REG(0.409),VCP:PPI(0.961)REG(0.634)		0.883
chr15	89843466	C	A	FANCI (Intron)	FANCI:PPI(0.767)REG(0.409)		1.280
chr15	89824390	C	T	FANCI (Intron)	FANCI:PPI(0.767)REG(0.409)		1.095
chr15	89824738	C	G	FANCI (Intron)	FANCI:PPI(0.767)REG(0.409)		1.095
chr15	89858081	T	C	FANCI (Intron)	FANCI:PPI(0.767)REG(0.409)		0.657
chr15	89833739	C	A	FANCI (Intron)	FANCI:PPI(0.767)REG(0.409)		0.472
chr15	89842938	G	C	FANCI (Intron)	FANCI:PPI(0.767)REG(0.409)		0.472
chr15	89836122	G	C	FANCI (Intron)	FANCI:PPI(0.767)REG(0.409)		0.472
chr15	89824342	C	A	FANCI (Intron)	FANCI:PPI(0.767)REG(0.409)		0.472
chr15	89811518	T	G	FANCI (Intron)	FANCI:PPI(0.767)REG(0.409)		0.472
chr2	58468259	C	T	FANCL (Intron)	FANCL:PPI(0.623)REG(0.634)		1.104
chr2	58457010	C	G	FANCL (Intron)	FANCL:PPI(0.623)REG(0.634)		0.930
chr2	58456947	A	C	FANCL (Intron)	FANCL:PPI(0.623)REG(0.634)		0.930
chr2	58388852	G	T	FANCL (Intron)	FANCL:PPI(0.623)REG(0.634)		0.307
chr14	45620591	A	C	FANCM (Intron)	FANCM:PPI(0.798)		1.144
chr14	45633522	A	G	FANCM (Intron)	FANCM:PPI(0.798)		0.521
chr2	48061657	A	T	FBXO11 (Intron and Promoter)	FBXO11:PPI(0.657)	MOTIFG=GATA_known5#48061657#48061662#-#5#4839#3491	1.921
chr2	48041205	G	A	FBXO11 (Intron)	FBXO11:PPI(0.657)		1.139
chr2	48030838	A	T	FBXO11 (Intron)	FBXO11:PPI(0.657),MSH6:PPI(0.908)REG(0.409)		2.552
chr2	48030347	G	A	FBXO11 (Intron)	FBXO11:PPI(0.657),MSH6:PPI(0.908)REG(0.409)		2.541
chr2	48030458	G	C	FBXO11 (Intron)	FBXO11:PPI(0.657),MSH6:PPI(0.908)REG(0.409)		1.929
chr2	48032312	G	A	FBXO11 (Intron)	FBXO11:PPI(0.657),MSH6:PPI(0.908)REG(0.409)		0.744
chr2	48040181	C	A	FBXO11 (Medial and UTR)	FBXO11:PPI(0.657)		1.922
chr10	5951018	G	A	FBXO18 (Intron)	FBXO18:PPI(0.408)REG(0.880)		2.488
chr10	5951025	T	C	FBXO18 (Intron)	FBXO18:PPI(0.408)REG(0.880)		1.865
chr10	5966597	C	G	FBXO18 (Intron)	FBXO18:PPI(0.408)REG(0.880)		0.680
chr10	5966527	C	G	FBXO18 (Intron)	FBXO18:PPI(0.408)REG(0.880)		0.680
chr10	5966622	C	G	FBXO18 (Intron)	FBXO18:PPI(0.408)REG(0.880)		0.680
chr1	161565237	T	C	FCGR2B (Intron)	FCGR2B:PPI(0.515),FCGR3A:PPI(0.875)		1.854
chr1	161565164	T	G	FCGR2B (Intron)	FCGR2B:PPI(0.515),FCGR3A:PPI(0.875)		1.854
chr1	161565129	C	A	FCGR2B (Intron)	FCGR2B:PPI(0.515),FCGR3A:PPI(0.875)		1.854
chr1	161576509	C	T	FCGR2B (Intron)	FCGR2B:PPI(0.515),FCGR3A:PPI(0.875)		2.425
chr1	161559256	C	T	FCGR2B (Intron)	FCGR2B:PPI(0.515),FCGR3A:PPI(0.875)		1.854

chr1	161559571	C	T	FCGR2B (Intron)	FCGR2B:PPI(0.515),FCGR3A:PPI(0.875)		1.854
chr1	161576425	G	A	FCGR2B (Intron)	FCGR2B:PPI(0.515),FCGR3A:PPI(0.875)		1.854
chr1	161559256	C	T	FCGR2B (Intron)	FCGR2B:PPI(0.515),FCGR3A:PPI(0.875)		1.854
chr1	161569497	C	T	FCGR2B (Intron)	FCGR2B:PPI(0.515),FCGR3A:PPI(0.875)		1.854
chr1	161565479	G	A	FCGR2B (Intron)	FCGR2B:PPI(0.515),FCGR3A:PPI(0.875)		1.669
chr1	161565479	G	A	FCGR2B (Intron)	FCGR2B:PPI(0.515),FCGR3A:PPI(0.875)		1.669
chr1	161565479	G	A	FCGR2B (Intron)	FCGR2B:PPI(0.515),FCGR3A:PPI(0.875)		1.669
chr1	161565399	C	T	FCGR2B (Intron)	FCGR2B:PPI(0.515),FCGR3A:PPI(0.875)		0.854
chr1	161561716	G	A	FCGR2B (Intron)	FCGR2B:PPI(0.515),FCGR3A:PPI(0.875)		0.669
chr1	161595801	G	A	FCGR2B (Intron)	FCGR2B:PPI(0.515),FCGR3A:PPI(0.875),FCGR3B:PPI(0.361)		0.854
chr11	61564299	G	T	FEN1 (UTR)	FEN1:PPI(0.952)REG(0.880)		1.473
chr19	48657227	G	C	FGF21 (Distal)	FGF21:PPI(0.482),LIG1:PHOS(0.980)PPI(0.814)REG(0.409)		1.124
chr8	38285614	G	A	FGFR1 (Intron and Medial)	FGFR1:PHOS(0.855)PPI(0.978)		1.118
chr6	167446254	G	A	FGFR1OP (Intron)	FGFR1OP:PHOS(0.792)PPI(0.307)		0.511
chr6	167453478	G	A	FGFR1OP (UTR)	FGFR1OP:PHOS(0.792)PPI(0.307)		0.511
chr10	123277642	A	G	FGFR2 (Intron)	FGFR2:PHOS(0.967)PPI(0.971)		1.535
chr10	123311082	T	G	FGFR2 (Intron)	FGFR2:PHOS(0.967)PPI(0.971)		0.912
chr10	123288091	C	T	FGFR2 (Intron)	FGFR2:PHOS(0.967)PPI(0.971)		0.912
chr10	123310626	C	T	FGFR2 (Intron)	FGFR2:PHOS(0.967)PPI(0.971)		0.912
chr10	123310738	A	C	FGFR2 (Intron)	FGFR2:PHOS(0.967)PPI(0.971)		0.912
chr10	123311056	C	T	FGFR2 (Intron)	FGFR2:PHOS(0.967)PPI(0.971)		0.912
chr10	123315285	C	G	FGFR2 (Intron)	FGFR2:PHOS(0.967)PPI(0.971)		0.912
chr1	241669256	C	T	FH (Intron)	FH:PPI(0.767)		0.472
chr1	241666183	C	A	FH (Intron)	FH:PPI(0.767)		0.472
chr2	105984216	G	T	FHL2 (Intron)	FHL2:PHOS(0.717)PPI(0.943)REG(0.880)		0.833
chr4	54308815	T	C	FIP1L1 (Intron)	FIP1L1:PHOS(0.928)PPI(0.817)REG(0.409)		1.417
chr4	54310041	T	G	FIP1L1 (Intron)	FIP1L1:PHOS(0.928)PPI(0.817)REG(0.409)		1.417
chr4	54852930	C	A	FIP1L1 (Intron)	FIP1L1:PHOS(0.928)PPI(0.817)REG(0.409)		0.979
chr4	54853122	T	G	FIP1L1 (Intron)	FIP1L1:PHOS(0.928)PPI(0.817)REG(0.409)		0.979
chr4	54954323	G	A	FIP1L1 (Intron)	FIP1L1:PHOS(0.928)PPI(0.817)REG(0.409)		0.794
chr4	54853303	C	G	FIP1L1 (Intron)	FIP1L1:PHOS(0.928)PPI(0.817)REG(0.409)		0.794

chr4	54373301	G	T	FIP1L1 (Intron)	FIP1L1:PHOS(0.928)PPI(0.817)REG(0.409),LNX1:PPI(0.921)		1.979
chr4	54373311	C	A	FIP1L1 (Intron)	FIP1L1:PHOS(0.928)PPI(0.817)REG(0.409),LNX1:PPI(0.921)		1.979
chr4	55127224	C	G	FIP1L1 (Intron)	FIP1L1:PHOS(0.928)PPI(0.817)REG(0.409),PDGFRA:PHOS(0.954)PPI(0.930)REG(0.634)		1.669
chr4	55156431	A	G	FIP1L1 (Intron)	FIP1L1:PHOS(0.928)PPI(0.817)REG(0.409),PDGFRA:PHOS(0.954)PPI(0.930)REG(0.634)		0.864
chr4	55154891	T	C	FIP1L1 (Intron)	FIP1L1:PHOS(0.928)PPI(0.817)REG(0.409),PDGFRA:PHOS(0.954)PPI(0.930)REG(0.634)		0.864
chr11	128628222	G	A	FLI1 (Intron and Medial)	FLI1:PPI(0.606)REG(0.761)		1.883
chr13	28913624	C	T	FLT1 (Intron)	FLT1:PHOS(0.585)PPI(0.866)		1.458
chr13	28970896	G	T	FLT1 (Intron)	FLT1:PHOS(0.585)PPI(0.866)		0.835
chr13	28880602	C	G	FLT1 (Intron)	FLT1:PHOS(0.585)PPI(0.866)		0.835
chr13	28624423	G	A	FLT3 (Intron)	FLT3:PPI(0.623)		0.481
chr13	28597479	G	A	FLT3 (Intron)	FLT3:PPI(0.623)		0.481
chr13	28578107	G	T	FLT3 (UTR)	FLT3:PPI(0.623)		0.296
chr5	180047560	T	G	FLT4 (Intron and Promoter)	FLT4:PPI(0.623)		0.482
chr5	180043819	T	G	FLT4 (Intron)	FLT4:PPI(0.623)		0.296
chr5	180050854	T	G	FLT4 (Intron)	FLT4:PPI(0.623)		0.296
chr9	132689621	C	T	FNBP1 (Intron)	FNBP1:PPI(0.707)		1.011
chr14	38064103	C	T	FOXA1 (Intron and Medial)	FOXA1:PPI(0.787)REG(0.999)		2.419
chr12	2973706	A	C	FOXM1 (Intron)	FOXM1:PPI(0.482)REG(0.634)		0.307
chr3	71090684	C	T	FOXP1 (Intron)	FOXP1:PHOS(0.276)PPI(0.515)REG(0.409)		0.832
chr3	71021682	G	A	FOXP1 (Intron)	FOXP1:PHOS(0.276)PPI(0.515)REG(0.409)		0.832
chr3	71026198	G	A	FOXP1 (Intron)	FOXP1:PHOS(0.276)PPI(0.515)REG(0.409)		0.832
chr3	71026789	C	T	FOXP1 (Intron)	FOXP1:PHOS(0.276)PPI(0.515)REG(0.409)		0.832
chr3	71019854	C	T	FOXP1 (Intron)	FOXP1:PHOS(0.276)PPI(0.515)REG(0.409)		0.394
chr16	53860411	C	T	FTO (Intron)	FTO:REG(0.761)		0.463
chr16	53968097	C	T	FTO (Intron)	FTO:REG(0.761)		0.463
chr1	78422074	T	G	FUBP1 (Intron)	FUBP1:PHOS(0.585)PPI(0.739)REG(0.761)		0.463

chr21	27134679	G	C	GABPA (Intron)	GABPA:PHOS(0.276)PPI(0.729)REG(0.999)		1.622
chr21	27135249	C	T	GABPA (Intron)	GABPA:PHOS(0.276)PPI(0.729)REG(0.999)		1.184
chr21	27130585	C	T	GABPA (Intron)	GABPA:PHOS(0.276)PPI(0.729)REG(0.999)		0.999
chr21	27135159	C	T	GABPA (Intron)	GABPA:PHOS(0.276)PPI(0.729)REG(0.999)		0.999
chr21	27135211	C	T	GABPA (Intron)	GABPA:PHOS(0.276)PPI(0.729)REG(0.999)		0.999
chr9	92220171	G	T	GADD45G (UTR)	GADD45G:PHOS(0.276)PPI(0.902)REG(0.409)		0.915
chr17	9843517	C	G	GAS7 (Intron)	GAS7:PHOS(0.693)PPI(0.809)		1.163
chr17	9843383	A	G	GAS7 (Intron)	GAS7:PHOS(0.693)PPI(0.809)		0.540
chr19	18331210	C	A	GDF15 (Distal)	GDF15:PPI(0.236)REG(0.929),JAK3:PPI(0.955),PDE4C:REG(0.409),SSBP4:PHOS(0.944)PPI(0.482)		1.674
chr2	17954647	T	G	GEN1 (Intron)	GEN1:REG(0.409),SMC6:PPI(0.739)		1.054
chr2	17955766	G	C	GEN1 (Intron)	GEN1:REG(0.409),SMC6:PPI(0.739)		0.910
chr2	17941423	T	G	GEN1 (Intron)	GEN1:REG(0.409),SMC6:PPI(0.739)		0.616
chr2	17955750	C	A	GEN1 (Intron)	GEN1:REG(0.409),SMC6:PPI(0.739)		0.431
chr2	17959186	C	A	GEN1 (Intron)	GEN1:REG(0.409),SMC6:PPI(0.739)		0.431
chr2	17959454	T	C	GEN1 (Intron)	GEN1:REG(0.409),SMC6:PPI(0.739)		0.431
chr2	17959099	T	G	GEN1 (Intron)	GEN1:REG(0.409),SMC6:PPI(0.739)		0.431
chr3	155615495	G	A	GMPS (Intron)	GMPS:PPI(0.671)		0.969
chr3	155611514	G	C	GMPS (Intron)	GMPS:PPI(0.671)		0.969
chr3	155634009	A	C	GMPS (Intron)	GMPS:PPI(0.671)		0.531
chr3	155615557	G	A	GMPS (Intron)	GMPS:PPI(0.671)		0.346
chr3	155615711	C	T	GMPS (Intron)	GMPS:PPI(0.671)		0.346
chr3	155633781	A	T	GMPS (Intron)	GMPS:PPI(0.671)		0.346
chr3	155633782	G	T	GMPS (Intron)	GMPS:PPI(0.671)		0.346
chr19	3118919	C	T	GNA11 (Intron)	GNA11:PPI(0.912)		1.377
chr20	57448746	C	T	GNAS (Intron)	GNAS:PPI(0.938)REG(0.634)		1.005
chr20	57485882	T	G	GNAS (UTR)	GNAS:PPI(0.938)REG(0.634)		1.617
chr14	93299812	G	C	GOLGA5 (Intron)	GOLGA5:PHOS(0.927)PPI(0.540)REG(0.409)		0.808
chr6	117894936	C	T	GOPC (Intron)	GOPC:PPI(0.866)REG(0.409)		1.273
chr6	117753140	G	A	GOPC (Intron)	GOPC:PPI(0.866)REG(0.409)		0.835
chr6	117896728	A	C	GOPC (Intron)	GOPC:PPI(0.866)REG(0.409)		0.650
chr6	117894799	C	G	GOPC (Intron)	GOPC:PPI(0.866)REG(0.409)		0.650

chr6	117725597	G	A	GOPC (Intron)	GOPC:PPI(0.866)REG(0.409),ROS1:PPI(0.739)		1.458
chr6	117681402	G	T	GOPC (Intron)	GOPC:PPI(0.866)REG(0.409),ROS1:PPI(0.739)		1.273
chr6	117737586	G	T	GOPC (Intron)	GOPC:PPI(0.866)REG(0.409),ROS1:PPI(0.739)		0.900
chr6	117714346	C	G	GOPC (Intron)	GOPC:PPI(0.866)REG(0.409),ROS1:PPI(0.739)		0.835
chr6	117649988	T	C	GOPC (Intron)	GOPC:PPI(0.866)REG(0.409),ROS1:PPI(0.739)		0.650
chr6	117739330	G	A	GOPC (Intron)	GOPC:PPI(0.866)REG(0.409),ROS1:PPI(0.739)		0.650
chr6	117645482	C	A	GOPC (Intron)	GOPC:PPI(0.866)REG(0.409),ROS1:PPI(0.739)		0.650
chr6	117687562	T	C	GOPC (Intron)	GOPC:PPI(0.866)REG(0.409),ROS1:PPI(0.739)		0.650
chr6	117737149	A	T	GOPC (Intron)	GOPC:PPI(0.866)REG(0.409),ROS1:PPI(0.739)		0.650
chr6	117683745	C	A	GOPC (Intron)	GOPC:PPI(0.866)REG(0.409),ROS1:PPI(0.739)		0.650
chr6	117881605	A	T	GOPC (UTR)	GOPC:PPI(0.866)REG(0.409)		1.458
chrX	132796046	C	T	GPC3 (Intron)	GPC3:PPI(0.361)		0.127
chrX	132826192	C	T	GPC3 (Intron)	GPC3:PPI(0.361)		0.127
chr14	67647404	C	T	GPHN (Intron and Promoter)	GPHN:PPI(0.623)		0.919
chr14	66974474	T	C	GPHN (Promoter and UTR)	GPHN:PPI(0.623)	MOTIFG=YY1_known1#66974469#66974475#+#6#6430#5491	2.681
chr8	37690520	C	T	GPR124 (Intron)	GPR124:PPI(0.142)		0.248
chr1	89353061	A	C	GTF2B (Intron)	GTF2B:PHOS(0.622)PPI(0.978)REG(0.996)		1.797
chr19	6381057	C	T	GTF2F1 (Intron)	GTF2F1:PHOS(0.911)PPI(0.990)REG(0.989),PSPN:PPI(0.307)		1.155
chr11	18373756	C	T	GTF2H1 (Intron)	GTF2H1:PHOS(0.761)PPI(0.973)REG(0.944)		0.918
chr5	68875576	C	T	GTF2H2C (Intron)			0.003
chr6	158579523	G	T	GTF2H5 (Medial)	GTF2H5:PHOS(0.398)PPI(0.641)		1.017
chr6	43578461	G	T	GTPBP2 (Intron), POLH (Intron)	GTPBP2:PPI(0.857)REG(0.909),POLH:PHOS(0.276)PPI(0.895)REG(0.761)		1.555
chr11	106681302	T	C	GUCY1A2 (Intron)	GUCY1A2:PPI(0.482)		0.811
chr11	106697992	C	A	GUCY1A2 (Intron)	GUCY1A2:PPI(0.482)		0.373

chr11	106694829	G	T	GUCY1A2 (Intron)	GUCY1A2:PPI(0.482)		0.188
chr11	106695702	T	G	GUCY1A2 (Intron)	GUCY1A2:PPI(0.482)		0.188
chr4	2240221	T	G	HAUS3 (Intron), POLN (Intron)			0.003
chr1	32768220	A	G	HDAC1 (Intron)	HDAC1:PHOS(0.276)PPI(0.997)		1.615
chr1	32794778	A	C	HDAC1 (Intron)	HDAC1:PHOS(0.276)PPI(0.997)		1.541
chr1	32794829	A	C	HDAC1 (Intron)	HDAC1:PHOS(0.276)PPI(0.997)		0.992
chr1	32798616	A	T	HDAC1 (Intron)	HDAC1:PHOS(0.276)PPI(0.997),MARCKSL1:PPI(0.236)REG(0.409)		3.412
chr1	32798017	C	G	HDAC1 (Intron)	HDAC1:PHOS(0.276)PPI(0.997),MARCKSL1:PPI(0.236)REG(0.409)		2.790
chr16	56975830	T	G	HERPUD1 (Intron)	HERPUD1:PPI(0.583)REG(0.830)		1.376
chr12	66309001	C	A	HMGA2 (UTR)	HMGA2:PPI(0.563)REG(0.409)		0.244
chr13	31039984	G	A	HMGB1 (Promoter and UTR)	HMGB1:PPI(0.990)REG(0.409)		2.390
chr20	43030141	G	C	HNF4A (Intron and Medial)	HNF4A:PPI(0.869)REG(0.995)		2.783
chr20	43031282	G	A	HNF4A (Intron and Medial)	HNF4A:PPI(0.869)REG(0.995)		2.783
chr8	76468377	C	A	HNF4G (Intron)	HNF4G:PPI(0.671)REG(0.991)		1.596
chr8	76472705	A	C	HNF4G (Intron)	HNF4G:PPI(0.671)REG(0.991)		1.596
chr8	76472485	G	A	HNF4G (Intron)	HNF4G:PPI(0.671)REG(0.991)		0.973
chr8	76468139	C	G	HNF4G (Intron)	HNF4G:PPI(0.671)REG(0.991)		0.973
chr8	76476457	G	A	HNF4G (UTR)	HNF4G:PPI(0.671)REG(0.991)		1.596
chr7	26235792	C	T	HNRNPA2B1 (Intron)	HNRNPA2B1:PPI(0.953)REG(0.929)		2.046
chr8	42821608	G	T	HOOK3 (Intron)	HOOK3:PPI(0.540)REG(0.409)		0.226
chr8	42873640	A	G	HOOK3 (UTR)	HOOK3:PPI(0.540)REG(0.409)		0.849
chr11	533450	C	T	HRAS (Intron)	HRAS:PPI(0.958)REG(0.409),LRRC56:REG(0.409)		1.683
chr14	102550935	A	G	HSP90AA1 (Intron)	HSP90AA1:PPI(0.781)REG(0.880)		0.865
chr7	48016442	G	A	HUS1 (Intron)	HUS1:PPI(0.787)REG(0.409)		1.126
chr7	48008738	A	G	HUS1 (Intron)	HUS1:PPI(0.787)REG(0.409)		0.688
chr7	48016442	G	A	HUS1 (Intron)	HUS1:PPI(0.787)REG(0.409)		1.126
chr7	48008738	A	G	HUS1 (Intron)	HUS1:PPI(0.787)REG(0.409)		0.688
chr7	47897185	T	G	HUS1 (Intron)	HUS1:PPI(0.787)REG(0.409),PKD1L1:REG(0.634)		2.126
chr7	47897185	T	G	HUS1 (Intron)	HUS1:PPI(0.787)REG(0.409),PKD1L1:REG(0.634)		2.126
chr7	47979716	A	C	HUS1 (Intron)	HUS1:PPI(0.787)REG(0.409),PKD1L1:REG(0.634)		1.688
chr7	47979996	G	A	HUS1 (Intron)	HUS1:PPI(0.787)REG(0.409),PKD1L1:REG(0.634)		1.688

chr7	47924106	T	G	HUS1 (Intron)	HUS1:PPI(0.787)REG(0.409),PKD1L1:REG(0.634)		1.503
chr7	47924120	T	G	HUS1 (Intron)	HUS1:PPI(0.787)REG(0.409),PKD1L1:REG(0.634)		1.503
chr7	47924106	T	G	HUS1 (Intron)	HUS1:PPI(0.787)REG(0.409),PKD1L1:REG(0.634)		1.503
chr7	47924106	T	G	HUS1 (Intron)	HUS1:PPI(0.787)REG(0.409),PKD1L1:REG(0.634)		1.503
chr7	47924120	T	G	HUS1 (Intron)	HUS1:PPI(0.787)REG(0.409),PKD1L1:REG(0.634)		1.503
chr7	47924106	T	G	HUS1 (Intron)	HUS1:PPI(0.787)REG(0.409),PKD1L1:REG(0.634)		1.503
chr7	47924120	T	G	HUS1 (Intron)	HUS1:PPI(0.787)REG(0.409),PKD1L1:REG(0.634)		1.503
chr7	47924106	T	G	HUS1 (Intron)	HUS1:PPI(0.787)REG(0.409),PKD1L1:REG(0.634)		1.503
chr7	47971937	A	T	HUS1 (Intron)	HUS1:PPI(0.787)REG(0.409),PKD1L1:REG(0.634)		0.688
chr7	47968699	G	T	HUS1 (Intron)	HUS1:PPI(0.787)REG(0.409),PKD1L1:REG(0.634)		0.688
chr7	47983252	G	T	HUS1 (Intron)	HUS1:PPI(0.787)REG(0.409),PKD1L1:REG(0.634)		0.688
chr7	47872658	A	G	HUS1 (Intron)	HUS1:PPI(0.787)REG(0.409),PKD1L1:REG(0.634)		0.688
chr7	47898561	C	T	HUS1 (Intron)	HUS1:PPI(0.787)REG(0.409),PKD1L1:REG(0.634)		0.504
chr7	47867074	G	A	HUS1 (Intron)	HUS1:PPI(0.787)REG(0.409),PKD1L1:REG(0.634)		0.503
chr7	47970985	G	A	HUS1 (Intron)	HUS1:PPI(0.787)REG(0.409),PKD1L1:REG(0.634)		0.503
chr7	47873803	C	A	HUS1 (Intron)	HUS1:PPI(0.787)REG(0.409),PKD1L1:REG(0.634)		0.503
chr7	47882467	T	G	HUS1 (Intron)	HUS1:PPI(0.787)REG(0.409),PKD1L1:REG(0.634)		0.503
chr7	47915651	G	C	HUS1 (Intron)	HUS1:PPI(0.787)REG(0.409),PKD1L1:REG(0.634)		0.503
chr7	47915677	A	G	HUS1 (Intron)	HUS1:PPI(0.787)REG(0.409),PKD1L1:REG(0.634)		0.503
chr7	47897185	T	G	HUS1 (Intron)	HUS1:PPI(0.787)REG(0.409),PKD1L1:REG(0.634)		2.126

chr7	47897185	T	G	HUS1 (Intron)	HUS1:PPI(0.787)REG(0.409),PKD1L1:REG(0.634)		2.126
chr7	47979716	A	C	HUS1 (Intron)	HUS1:PPI(0.787)REG(0.409),PKD1L1:REG(0.634)		1.688
chr7	47979996	G	A	HUS1 (Intron)	HUS1:PPI(0.787)REG(0.409),PKD1L1:REG(0.634)		1.688
chr7	47924106	T	G	HUS1 (Intron)	HUS1:PPI(0.787)REG(0.409),PKD1L1:REG(0.634)		1.503
chr7	47924120	T	G	HUS1 (Intron)	HUS1:PPI(0.787)REG(0.409),PKD1L1:REG(0.634)		1.503
chr7	47924106	T	G	HUS1 (Intron)	HUS1:PPI(0.787)REG(0.409),PKD1L1:REG(0.634)		1.503
chr7	47924106	T	G	HUS1 (Intron)	HUS1:PPI(0.787)REG(0.409),PKD1L1:REG(0.634)		1.503
chr7	47924120	T	G	HUS1 (Intron)	HUS1:PPI(0.787)REG(0.409),PKD1L1:REG(0.634)		1.503
chr7	47924106	T	G	HUS1 (Intron)	HUS1:PPI(0.787)REG(0.409),PKD1L1:REG(0.634)		1.503
chr7	47924120	T	G	HUS1 (Intron)	HUS1:PPI(0.787)REG(0.409),PKD1L1:REG(0.634)		1.503
chr7	47924106	T	G	HUS1 (Intron)	HUS1:PPI(0.787)REG(0.409),PKD1L1:REG(0.634)		1.503
chr7	47971937	A	T	HUS1 (Intron)	HUS1:PPI(0.787)REG(0.409),PKD1L1:REG(0.634)		0.688
chr7	47968699	G	T	HUS1 (Intron)	HUS1:PPI(0.787)REG(0.409),PKD1L1:REG(0.634)		0.688
chr7	47983252	G	T	HUS1 (Intron)	HUS1:PPI(0.787)REG(0.409),PKD1L1:REG(0.634)		0.688
chr7	47872658	A	G	HUS1 (Intron)	HUS1:PPI(0.787)REG(0.409),PKD1L1:REG(0.634)		0.688
chr7	47898561	C	T	HUS1 (Intron)	HUS1:PPI(0.787)REG(0.409),PKD1L1:REG(0.634)		0.504
chr7	47867074	G	A	HUS1 (Intron)	HUS1:PPI(0.787)REG(0.409),PKD1L1:REG(0.634)		0.503
chr7	47970985	G	A	HUS1 (Intron)	HUS1:PPI(0.787)REG(0.409),PKD1L1:REG(0.634)		0.503
chr7	47873803	C	A	HUS1 (Intron)	HUS1:PPI(0.787)REG(0.409),PKD1L1:REG(0.634)		0.503
chr7	47882467	T	G	HUS1 (Intron)	HUS1:PPI(0.787)REG(0.409),PKD1L1:REG(0.634)		0.503

chr7	47915651	G	C	HUS1 (Intron)	HUS1:PPI(0.787)REG(0.409),PKD1L1:REG(0.634)		0.503
chr7	47915677	A	G	HUS1 (Intron)	HUS1:PPI(0.787)REG(0.409),PKD1L1:REG(0.634)		0.503
chr2	209104866	T	C	IDH1 (Intron)	IDH1:PPI(0.739)REG(0.409)		1.228
chr3	129156953	T	A	IFT122 (Promoter), MBD4 (Intron)	IFT122:PPI(0.142)REG(0.634),MBD4:PHOS(0.276)PPI(0.482)REG(0.634)		0.307
chr3	129156797	G	A	IFT122 (Promoter), MBD4 (Intron)	IFT122:PPI(0.142)REG(0.634),MBD4:PHOS(0.276)PPI(0.482)REG(0.634)		0.307
chr3	129158797	G	C	IFT122 (Promoter), MBD4 (Promoter and UTR)	IFT122:PPI(0.142)REG(0.634),MBD4:PHOS(0.276)PPI(0.482)REG(0.634)		1.104
chr15	99465924	C	T	IGF1R (Intron and Promoter)	IGF1R:PHOS(0.709)PPI(0.936)		0.815
chr15	99439738	A	G	IGF1R (Intron)	IGF1R:PHOS(0.709)PPI(0.936)		2.612
chr15	99439878	C	G	IGF1R (Intron)	IGF1R:PHOS(0.709)PPI(0.936)		2.612
chr15	99465184	C	T	IGF1R (Intron)	IGF1R:PHOS(0.709)PPI(0.936)		1.623
chr15	99456511	G	A	IGF1R (Intron)	IGF1R:PHOS(0.709)PPI(0.936)		1.438
chr6	160461866	C	G	IGF2R (Intron)	IGF2R:PPI(0.671)REG(0.409)		0.531
chr6	160510343	G	A	IGF2R (Intron)	IGF2R:PPI(0.671)REG(0.409)		0.531
chr6	160524773	T	A	IGF2R (Intron)	IGF2R:PPI(0.671)REG(0.409)		0.531
chr6	160412144	G	T	IGF2R (Intron)	IGF2R:PPI(0.671)REG(0.409)		0.346
chr6	160479882	G	A	IGF2R (Intron)	IGF2R:PPI(0.671)REG(0.409)		0.346
chr11	68701118	C	T	IGHMBP2 (Intron)	IGHMBP2:PPI(0.482)REG(0.409)		1.188
chr11	68701118	C	T	IGHMBP2 (Intron)	IGHMBP2:PPI(0.482)REG(0.409)		1.188
chr11	68706935	A	G	IGHMBP2 (Intron)	IGHMBP2:PPI(0.482)REG(0.409)		0.811
chr11	68705876	G	A	IGHMBP2 (Intron)	IGHMBP2:PPI(0.482)REG(0.409)		0.373
chr11	68702632	C	T	IGHMBP2 (Intron)	IGHMBP2:PPI(0.482)REG(0.409)		0.373
chr11	68700701	G	C	IGHMBP2 (Intron)	IGHMBP2:PPI(0.482)REG(0.409)		0.188
chr7	50358702	G	T	IKZF1 (Intron)	IKZF1:PHOS(0.585)PPI(0.850)		1.240
chr11	71715256	C	T	IL18BP (Intron), NUMA1 (Intron)	IL18BP:PPI(0.236),NUMA1:PPI(0.718)REG(0.409)		1.588
chr5	131894751	T	C	IL5 (Promoter), RAD50 (Intron)	IL5:PPI(0.841),RAD50:PPI(0.964)REG(0.761)		0.892
chr5	35857177	G	C	IL7R (Intron and Medial)	IL7R:PPI(0.934)		3.229
chr5	35857177	G	C	IL7R (Intron and Medial)	IL7R:PPI(0.934)		3.229
chr5	35857289	C	G	IL7R (Intron and Medial)	IL7R:PPI(0.934)		2.607
chr5	35874668	A	T	IL7R (Intron and Promoter)	IL7R:PPI(0.934)		1.779
chr5	35861152	C	G	IL7R (Intron)	IL7R:PPI(0.934)		0.809
chr5	35857053	C	G	IL7R (Medial and UTR)	IL7R:PPI(0.934)		2.607
chr15	41361867	C	T	INO80 (Intron)	INO80:REG(0.634)		0.930

chr15	41365696	G	A	INO80 (Intron)	INO80:REG(0.634)		0.930
chr15	41280283	G	A	INO80 (Intron)	INO80:REG(0.634)		0.307
chr15	41350768	C	T	INO80 (Intron)	INO80:REG(0.634)		0.307
chr19	17941235	G	T	INSL3 (Medial)	INSL3:PPI(0.236),JAK3:PPI(0.955)	MOTIFBR=NRF1#Nrf1_disc1_8mer#17941228#17941243#-#8#0004202#0978991,E2F6#E2F_disc2_8mer#17941229#17941239#+#7#0000000#1000000,E2F6#E2F_disc2_8mer#17941229#17941239#-#4#0000000#1000000	2.655
chr19	7119209	T	C	INSR (Intron)	INSR:PPI(0.945)		1.462
chr19	7150893	C	A	INSR (Intron)	INSR:PPI(0.945)		1.024
chr19	7119656	C	T	INSR (Intron)	INSR:PPI(0.945)		1.024
chr19	7170936	G	A	INSR (Intron)	INSR:PPI(0.945)		0.839
chr19	7163367	C	T	INSR (Intron)	INSR:PPI(0.945)		0.839
chr19	7250316	A	C	INSR (Intron)	INSR:PPI(0.945)		0.839
chr19	7172209	C	T	INSR (Intron)	INSR:PPI(0.945)		0.839
chr1	156819035	C	T	INSRR (Intron), NTRK1 (Intron)	INSRR:PPI(0.142),NTRK1:PPI(0.923)		1.589
chr1	156815921	C	T	INSRR (Intron), NTRK1 (Intron)	INSRR:PPI(0.142),NTRK1:PPI(0.923)		1.589
chr1	156814478	G	A	INSRR (Intron), NTRK1 (Intron)	INSRR:PPI(0.142),NTRK1:PPI(0.923)		1.404
chr1	156812579	C	T	INSRR (Intron), NTRK1 (Intron)	INSRR:PPI(0.142),NTRK1:PPI(0.923)		1.404
chrX	70519994	A	C	ITGB1BP2 (Promoter), NONO (UTR)	ITGB1BP2:PPI(0.408),NONO:PHOS(0.398)PPI(0.831)		1.388
chr5	156641422	G	C	ITK (Intron and Medial)	ITK:PPI(0.826)		0.756
chr5	156644736	G	A	ITK (Intron)	ITK:PPI(0.826)		1.194
chr5	156644920	G	A	ITK (Intron)	ITK:PPI(0.826)		1.194
chr5	156635862	C	A	ITK (Intron)	ITK:PPI(0.826)		1.192
chr5	156665038	C	A	ITK (Intron)	ITK:PPI(0.826)		0.571
chr1	65310574	T	G	JAK1 (Intron)	JAK1:PPI(0.967)		2.102
chr1	65310630	A	C	JAK1 (Intron)	JAK1:PPI(0.967)		2.086
chr1	65313138	T	G	JAK1 (Intron)	JAK1:PPI(0.967)		1.709
chr1	65307285	C	G	JAK1 (Intron)	JAK1:PPI(0.967)		1.523
chr1	65305575	C	A	JAK1 (Intron)	JAK1:PPI(0.967)		0.901
chr1	65301977	A	C	JAK1 (Intron)	JAK1:PPI(0.967)		0.901

chr9	5089672	A	G	JAK2 (Intron)	JAK2:PHOS(0.931)PPI(0.981)		1.565
chr9	5021874	C	G	JAK2 (Intron)	JAK2:PHOS(0.931)PPI(0.981)		0.942
chr19	17954734	C	G	JAK3 (Intron)	JAK3:PPI(0.955)		1.674
chr19	17945532	T	C	JAK3 (Intron)	JAK3:PPI(0.955)		1.052
chr19	17948029	A	G	JAK3 (Intron)	JAK3:PPI(0.955)		1.052
chr5	78595909	T	G	JMY (Intron)			0.003
chr5	78608509	G	A	JMY (Intron)			0.003
chr19	1367352	A	T	JSRP1 (Distal)			1.188
chr19	1367226	G	A	JSRP1 (Distal)			1.188
chr3	20164345	C	T	KAT2B (Intron)	KAT2B:PPI(0.761)REG(0.634)		1.271
chr3	20141482	C	G	KAT2B (Intron)	KAT2B:PPI(0.761)REG(0.634)		0.648
chr3	20156484	A	C	KAT2B (Intron)	KAT2B:PPI(0.761)REG(0.634)		0.463
chr3	20113719	T	G	KAT2B (Intron)	KAT2B:PPI(0.761)REG(0.634)		0.463
chr17	61600958	G	T	KCNH6 (Intron)	KCNH6:REG(0.409)	MOTIFBR=REST#NRSF_disc1_8mer#61600952#61600967#-#9#0021008#0936976,REST#NRSF_disc5_8mer#61600954#61600965#+#5#0000000#0903673	1.936
chr12	419869	G	T	KDM5A (Intron)	KDM5A:PPI(0.142)REG(0.634)		0.930
chr12	420278	T	G	KDM5A (Intron)	KDM5A:PPI(0.142)REG(0.634)		0.930
chr12	406157	C	A	KDM5A (Intron)	KDM5A:PPI(0.142)REG(0.634)		0.412
chr12	438226	C	T	KDM5A (Intron)	KDM5A:PPI(0.142)REG(0.634)		0.307
chr12	433111	C	T	KDM5A (Intron)	KDM5A:PPI(0.142)REG(0.634)		0.307
chr12	433027	C	A	KDM5A (Intron)	KDM5A:PPI(0.142)REG(0.634)		0.307
chr12	472379	C	A	KDM5A (Intron)	KDM5A:PPI(0.142)REG(0.634)		0.307
chrX	44870265	G	T	KDM6A (Intron)	KDM6A:PPI(0.142)REG(0.634)		1.899
chrX	44969495	G	C	KDM6A (Intron)	KDM6A:PPI(0.142)REG(0.634)		1.115
chrX	44880139	G	C	KDM6A (Intron)	KDM6A:PPI(0.142)REG(0.634)		0.930
chrX	44938599	A	C	KDM6A (Intron)	KDM6A:PPI(0.142)REG(0.634)		0.930
chrX	44897018	A	G	KDM6A (Intron)	KDM6A:PPI(0.142)REG(0.634)		0.930
chrX	44894258	C	G	KDM6A (Intron)	KDM6A:PPI(0.142)REG(0.634)		0.930
chrX	44896936	T	G	KDM6A (Intron)	KDM6A:PPI(0.142)REG(0.634)		0.307
chr4	55971831	G	A	KDR (Intron)	KDR:PPI(0.921)		0.961
chr4	55948071	C	G	KDR (Intron)	KDR:PPI(0.921)		0.776
chr4	55956414	C	T	KDR (Intron)	KDR:PPI(0.921)		0.776
chr4	55976999	C	T	KDR (Intron)	KDR:PPI(0.921)		0.776
chr4	55979365	G	T	KDR (Intron)	KDR:PPI(0.921)		0.776
chr10	32327865	A	C	KIF5B (Intron)	KIF5B:PPI(0.883)		1.872

chr10	32327865	A	C	KIF5B (Intron)	KIF5B:PPI(0.883)		1.872
chr10	32344728	G	C	KIF5B (Intron)	KIF5B:PPI(0.883)		1.484
chr10	7824956	A	G	KIN (Intron)	KIN:PPI(0.515)REG(0.409)		0.832
chr10	7811698	T	C	KIN (Intron)	KIN:PPI(0.515)REG(0.409)		0.209
chr4	55561630	C	T	KIT (Intron)	KIT:PHOS(0.601)PPI(0.899)		0.723
chr4	55573644	A	G	KIT (Intron)	KIT:PHOS(0.601)PPI(0.899)		0.723
chr14	104167075	C	T	KLC1 (Intron), XRCC3 (Intron)	XRCC3:PHOS(0.276)PPI(0.908)REG(0.634)		0.929
chr19	51361711	G	A	KLK3 (Intron)	KLK3:PPI(0.671)		0.346
chr19	51358028	C	A	KLK3 (Promoter)	KLK3:PPI(0.671)		0.531
chr12	49448983	A	G	KMT2D (Intron), MLL2 (Medial)	MLL2:PPI(0.685)		2.159
chr12	49449027	C	A	KMT2D (Intron), MLL2 (Medial)	MLL2:PPI(0.685)		1.547
chr12	25378346	C	A	KRAS (Intron)	KRAS:PPI(0.980)REG(0.409)		0.939
chr12	52910647	G	A	KRT5 (Intron)	KRT5:PPI(0.606)REG(0.634)		0.918
chr14	56108520	A	G	KTN1 (Intron and Promoter)	KTN1:PPI(0.563)		0.244
chr14	56101212	C	T	KTN1 (Intron)	KTN1:PPI(0.563)		1.052
chr14	56106433	G	A	KTN1 (Intron)	KTN1:PPI(0.563)		0.867
chr14	56147673	C	A	KTN1 (Intron)	KTN1:PPI(0.563)		0.867
chr14	56119454	C	T	KTN1 (Intron)	KTN1:PPI(0.563)		0.867
chr14	56125337	G	T	KTN1 (Intron)	KTN1:PPI(0.563)		0.244
chr14	56095054	A	G	KTN1 (Intron)	KTN1:PPI(0.563)		0.244
chr14	56114885	G	T	KTN1 (Intron)	KTN1:PPI(0.563)		0.244
chr14	56115633	T	C	KTN1 (Intron)	KTN1:PPI(0.563)		0.244
chr14	56145005	T	A	KTN1 (Intron)	KTN1:PPI(0.563)		0.244
chr14	56104620	C	T	KTN1 (Intron)	KTN1:PPI(0.563)		0.244
chr14	56133942	C	T	KTN1 (Intron)	KTN1:PPI(0.563)		0.244
chr14	56147540	G	T	KTN1 (Intron)	KTN1:PPI(0.563)		0.244
chr14	56078739	T	G	KTN1 (UTR)	KTN1:PPI(0.563)		1.052
chr17	37054773	G	C	LASP1 (Intron)	LASP1:PPI(0.685)REG(0.952)		1.666
chr1	32740687	G	A	LCK (Intron and Medial)	LCK:PHOS(0.953)PPI(0.987)REG(0.409)		1.769
chr5	38493888	C	G	LIFR (Intron)	LIFR:PPI(0.941)REG(0.409)		2.248
chr5	38482787	T	G	LIFR (Intron)	LIFR:PPI(0.941)REG(0.409)		0.828
chr19	48626066	T	G	LIG1 (Intron)	LIG1:PHOS(0.980)PPI(0.814)REG(0.409)		2.124
chr19	48626408	G	C	LIG1 (Intron)	LIG1:PHOS(0.980)PPI(0.814)REG(0.409)		2.124
chr19	48660401	G	A	LIG1 (Intron)	LIG1:PHOS(0.980)PPI(0.814)REG(0.409)		1.562
chr19	48653103	G	A	LIG1 (Intron)	LIG1:PHOS(0.980)PPI(0.814)REG(0.409)		1.124
chr19	48634540	G	T	LIG1 (Intron)	LIG1:PHOS(0.980)PPI(0.814)REG(0.409)		0.939

chr17	33326876	G	T	LIG3 (Intron)	LIG3:PPI(0.695)REG(0.409)		0.997
chr17	33318193	A	C	LIG3 (Intron)	LIG3:PPI(0.695)REG(0.409)		0.997
chr17	33319222	T	C	LIG3 (Intron)	LIG3:PPI(0.695)REG(0.409)		0.559
chr13	76430749	G	C	LMO7 (Intron)	LMO7:PPI(0.583)		0.883
chr13	76383238	T	C	LMO7 (Intron)	LMO7:PPI(0.583)		0.859
chr13	76398220	T	C	LMO7 (Intron)	LMO7:PPI(0.583)		0.260
chr13	76398089	A	C	LMO7 (Intron)	LMO7:PPI(0.583)		0.260
chr13	76432182	T	A	LMO7 (UTR)	LMO7:PPI(0.583)		0.260
chr3	188584192	A	C	LPP (Intron)	LPP:PPI(0.515)REG(0.409)		0.209
chr2	141116551	C	T	LRP1B (Intron and Promoter)	LRP1B:PPI(0.482)		0.188
chr2	141215326	G	A	LRP1B (Intron)	LRP1B:PPI(0.482)		1.996
chr2	141215314	A	G	LRP1B (Intron)	LRP1B:PPI(0.482)		1.996
chr2	141473709	G	C	LRP1B (Intron)	LRP1B:PPI(0.482)		1.811
chr2	141473709	G	C	LRP1B (Intron)	LRP1B:PPI(0.482)		1.811
chr2	141200205	A	C	LRP1B (Intron)	LRP1B:PPI(0.482)		1.373
chr2	141200205	A	C	LRP1B (Intron)	LRP1B:PPI(0.482)		1.373
chr2	141200205	A	C	LRP1B (Intron)	LRP1B:PPI(0.482)		1.373
chr2	141200205	A	C	LRP1B (Intron)	LRP1B:PPI(0.482)		1.373
chr2	141200205	A	C	LRP1B (Intron)	LRP1B:PPI(0.482)		1.373
chr2	141773529	A	C	LRP1B (Intron)	LRP1B:PPI(0.482)		1.188
chr2	141773529	A	C	LRP1B (Intron)	LRP1B:PPI(0.482)		1.188
chr2	141773529	A	C	LRP1B (Intron)	LRP1B:PPI(0.482)		1.188
chr2	141812677	C	A	LRP1B (Intron)	LRP1B:PPI(0.482)		0.996
chr2	141474094	G	C	LRP1B (Intron)	LRP1B:PPI(0.482)		0.811
chr2	141607919	C	G	LRP1B (Intron)	LRP1B:PPI(0.482)		0.811
chr2	141232909	A	C	LRP1B (Intron)	LRP1B:PPI(0.482)		0.811
chr2	141473863	C	T	LRP1B (Intron)	LRP1B:PPI(0.482)		0.811
chr2	141113820	G	A	LRP1B (Intron)	LRP1B:PPI(0.482)		0.811
chr2	141055237	T	G	LRP1B (Intron)	LRP1B:PPI(0.482)		0.811
chr2	141135886	C	G	LRP1B (Intron)	LRP1B:PPI(0.482)		0.811
chr2	141751710	G	C	LRP1B (Intron)	LRP1B:PPI(0.482)		0.811
chr2	141709514	G	A	LRP1B (Intron)	LRP1B:PPI(0.482)		0.811
chr2	141607616	A	G	LRP1B (Intron)	LRP1B:PPI(0.482)		0.810
chr2	141202381	G	A	LRP1B (Intron)	LRP1B:PPI(0.482)		0.809
chr2	141656244	A	C	LRP1B (Intron)	LRP1B:PPI(0.482)		0.373
chr2	141440940	C	T	LRP1B (Intron)	LRP1B:PPI(0.482)		0.373
chr2	141292006	T	G	LRP1B (Intron)	LRP1B:PPI(0.482)		0.188
chr2	141732178	G	T	LRP1B (Intron)	LRP1B:PPI(0.482)		0.188

chr2	141751736	A	G	LRP1B (Intron)	LRP1B:PPI(0.482)		0.188
chr2	141055140	T	G	LRP1B (Intron)	LRP1B:PPI(0.482)		0.188
chr2	141055226	T	G	LRP1B (Intron)	LRP1B:PPI(0.482)		0.188
chr2	141208062	T	G	LRP1B (Intron)	LRP1B:PPI(0.482)		0.188
chr2	141250045	T	G	LRP1B (Intron)	LRP1B:PPI(0.482)		0.188
chr2	141298303	C	G	LRP1B (Intron)	LRP1B:PPI(0.482)		0.188
chr2	141122395	C	T	LRP1B (Intron)	LRP1B:PPI(0.482)		0.188
chr2	141128719	A	C	LRP1B (Intron)	LRP1B:PPI(0.482)		0.188
chr2	141299562	T	C	LRP1B (Intron)	LRP1B:PPI(0.482)		0.188
chr2	141459204	G	T	LRP1B (Intron)	LRP1B:PPI(0.482)		0.188
chr2	141816732	C	T	LRP1B (Intron)	LRP1B:PPI(0.482)		0.188
chr2	142074279	C	A	LRP1B (Intron)	LRP1B:PPI(0.482)		0.188
chr2	142567718	G	T	LRP1B (Intron)	LRP1B:PPI(0.482)		0.188
chr2	142489077	A	T	LRP1B (Intron)	LRP1B:PPI(0.482)		0.188
chr2	141115424	G	A	LRP1B (Intron)	LRP1B:PPI(0.482)		0.188
chr2	141709652	G	A	LRP1B (Intron)	LRP1B:PPI(0.482)		0.188
chr2	141299304	G	T	LRP1B (Intron)	LRP1B:PPI(0.482)		0.188
chr2	142012027	G	T	LRP1B (Intron)	LRP1B:PPI(0.482)		0.188
chr2	142238110	G	C	LRP1B (Intron)	LRP1B:PPI(0.482)		0.188
chr2	142520423	C	A	LRP1B (Intron)	LRP1B:PPI(0.482)		0.188
chr2	142888376	G	C	LRP1B (Medial and UTR)	LRP1B:PPI(0.482)	MOTIFG=E2F_disc8#142888371#142888382#-#6#8256#7602	1.337
chr12	12273928	C	A	LRP6 (UTR)	LRP6:PPI(0.787)REG(0.409)		1.126
chr1	46739168	C	T	LRRC41 (Intron), RAD54L (Intron)	LRRC41:PPI(0.606)REG(0.909),RAD54L:PPI(0.930)REG(0.830)		1.607
chr1	46727077	C	T	LRRC41 (Intron), RAD54L (Intron)	LRRC41:PPI(0.606)REG(0.909),RAD54L:PPI(0.930)REG(0.830)		1.049
chr3	37096688	A	C	LRRFIP2 (Intron), MLH1 (Intron)	LRRFIP2:PPI(0.361)REG(0.409),MLH1:PPI(0.930)REG(0.880)		0.984
chr3	37100144	T	G	LRRFIP2 (Intron), MLH1 (Intron)	LRRFIP2:PPI(0.361)REG(0.409),MLH1:PPI(0.930)REG(0.880)		0.799
chr3	14220095	C	G	LSM3 (UTR), XPC (Promoter and UTR)	LSM3:PHOS(0.398)PPI(0.893)REG(0.830),XPC:PPI(0.897)REG(0.830)		2.515

chr3	14220332	G	C	LSM3 (UTR), XPC (Promoter)	LSM3:PHOS(0.398)PPI(0.893)REG(0.830),XPC:PPI(0.897)REG(0.830)	MOTIFBR=BRCA1#BRCA1_disc1_8mer#14220323#14220333#+#10#0000000#0037037,BRCA1#BRCA1_disc1_8mer#14220323#14220333#-#1#0066667#0192593,CHD2#CHD2_disc1_8mer#14220323#14220333#-#1#0000000#0141509,ELK4#Ets_disc3_8mer#14220323#14220333#-#1#0000000#0108333,ETS1#Ets_disc3_8mer#14220323#14220333#-#1#0000000#0108333,GABPA#Ets_disc3_8mer#14220323#14220333#-#1#0000000#0108333,E2F1#E2F_disc3_8mer#14220330#14220343#-#11#0157895#0457490,E2F4#E2F_disc3_8mer#14220330#14220343#-#11#0157895#0457490,E2F6#E2F_disc3_8mer#14220330#14220343#-#11#0157895#0457490	4.117
chr15	41799911	C	T	LTK (Intron)	LTK:PPI(0.482)		0.985
chr15	41801532	C	T	LTK (Intron)	LTK:PPI(0.482)		0.188
chr22	38609899	G	A	MAFF (Intron)	MAFF:PHOS(0.276)PPI(0.623)REG(0.992)		1.784
chr18	56383049	G	A	MALT1 (Intron)	MALT1:PPI(0.685)		0.583
chr11	95713175	C	T	MAML2 (Intron)	MAML2:PPI(0.583)REG(0.830)		0.578
chr15	66781485	G	T	MAP2K1 (Intron)	MAP2K1:PHOS(0.651)PPI(0.902)REG(0.634)		0.730
chr15	66781484	T	G	MAP2K1 (Intron)	MAP2K1:PHOS(0.651)PPI(0.902)REG(0.634)		0.730
chr15	66679819	G	C	MAP2K1 (Intron)	MAP2K1:PHOS(0.651)PPI(0.902)REG(0.634),TIPIN:PHOS(0.622)PPI(0.236)REG(0.761)	MOTIFBR=GABPA#Ets_disc7_8mer#66679819#66679833#+#1#0193789#0806211	3.134

chr17	11984852	G	T	MAP2K4 (Intron)	MAP2K4:PHOS(0.769)PPI(0.853)		1.246
chr17	12043202	G	A	MAP2K4 (Intron)	MAP2K4:PHOS(0.769)PPI(0.853)		1.246
chr17	12028690	T	C	MAP2K4 (Intron)	MAP2K4:PHOS(0.769)PPI(0.853)		1.246
chr17	12011252	C	T	MAP2K4 (Intron)	MAP2K4:PHOS(0.769)PPI(0.853)		0.808
chr17	12011253	A	T	MAP2K4 (Intron)	MAP2K4:PHOS(0.769)PPI(0.853)		0.808
chr5	56161284	G	T	MAP3K1 (Intron)	MAP3K1:PPI(0.988)		1.587
chr5	56160761	G	A	MAP3K1 (Intron)	MAP3K1:PPI(0.988)		1.587
chr5	56161289	T	G	MAP3K1 (Intron)	MAP3K1:PPI(0.988)		1.587
chr5	56183347	G	A	MAP3K1 (Intron)	MAP3K1:PPI(0.988)		1.587
chr5	56168646	T	G	MAP3K1 (Intron)	MAP3K1:PPI(0.988)		1.587
chr5	56177099	G	T	MAP3K1 (Intron)	MAP3K1:PPI(0.988)		1.587
chr5	56176513	G	C	MAP3K1 (Intron)	MAP3K1:PPI(0.988)		1.587
chr5	56174974	G	T	MAP3K1 (Intron)	MAP3K1:PPI(0.988)		1.442
chr5	56150969	T	C	MAP3K1 (Intron)	MAP3K1:PPI(0.988)		1.149
chr5	56168407	G	T	MAP3K1 (Intron)	MAP3K1:PPI(0.988)		0.964
chr5	56152416	C	A	MAP3K1 (Intron)	MAP3K1:PPI(0.988)		0.964
chr5	56189312	G	C	MAP3K1 (Intron)	MAP3K1:PPI(0.988)		0.964
chr11	64572435	G	T	MAP4K2 (Promoter), MEN1 (Intron)	MAP4K2:PHOS(0.984)PPI(0.641),MEN1:PHOS(0.398)PPI(0.774)REG(0.409)		1.137
chr11	64571733	G	C	MAP4K2 (Promoter), MEN1 (UTR)	MAP4K2:PHOS(0.984)PPI(0.641),MEN1:PHOS(0.398)PPI(0.774)REG(0.409)		1.749
chr14	65568197	C	A	MAX (Intron)	MAX:PHOS(0.803)PPI(0.898)REG(1.000)		1.002
chr8	48879850	A	G	MCM4 (Intron and Medial)	MCM4:PHOS(0.276)PPI(0.880)REG(0.830),PRKDC:PPI(0.938)REG(0.761)		1.005
chr8	48874387	G	A	MCM4 (Intron and Medial)	MCM4:PHOS(0.276)PPI(0.880)REG(0.830),PRKDC:PPI(0.938)REG(0.761)		1.617
chr8	6337999	A	G	MCPH1 (Intron)			0.003
chr8	6479438	A	G	MCPH1 (Intron)			0.003
chrX	70341169	A	C	MED12 (Intron)	MED12:PPI(0.835)		1.773
chrX	70341169	A	C	MED12 (Intron)	MED12:PPI(0.835)		1.773
chrX	70343577	A	C	MED12 (Intron)	MED12:PPI(0.835)		1.773
chrX	70351767	C	A	MED12 (Intron)	MED12:PPI(0.835)		1.773
chrX	70351718	G	C	MED12 (Intron)	MED12:PPI(0.835)		1.773
chrX	70343577	A	C	MED12 (Intron)	MED12:PPI(0.835)		1.773
chrX	70344763	G	T	MED12 (Intron)	MED12:PPI(0.835)		1.588
chrX	70344763	G	T	MED12 (Intron)	MED12:PPI(0.835)		1.588
chrX	70344763	G	T	MED12 (Intron)	MED12:PPI(0.835)		1.588
chrX	70339498	C	T	MED12 (Intron)	MED12:PPI(0.835)		1.385
chrX	70350110	C	A	MED12 (Intron)	MED12:PPI(0.835)		1.211

chrX	70348080	G	T	MED12 (Intron)	MED12:PPI(0.835)		1.211
chrX	70348344	G	A	MED12 (Intron)	MED12:PPI(0.835)		1.211
chrX	70361967	G	A	MED12 (Intron)	MED12:PPI(0.835)		0.773
chrX	70344561	T	G	MED12 (Intron)	MED12:PPI(0.835)		0.773
chrX	70360813	A	C	MED12 (Intron)	MED12:PPI(0.835)		0.588
chrX	70352417	T	C	MED12 (Intron)	MED12:PPI(0.835)		0.588
chrX	70356642	T	C	MED12 (Intron)	MED12:PPI(0.835)		0.588
chrX	70361608	T	C	MED12 (Intron)	MED12:PPI(0.835)		0.588
chrX	70341669	A	C	MED12 (Intron)	MED12:PPI(0.835)		0.588
chrX	70340817	C	T	MED12 (Intron)	MED12:PPI(0.835)		0.588
chrX	70347179	C	G	MED12 (Intron)	MED12:PPI(0.835)		0.588
chrX	70341924	G	T	MED12 (Intron)	MED12:PPI(0.835)		0.588
chrX	70346998	G	A	MED12 (Intron)	MED12:PPI(0.835)		0.588
chr15	100243672	G	A	MEF2A (Intron)	MEF2A:PHOS(0.601)PPI(0.761)REG(0.989)		1.775
chr15	100215862	C	G	MEF2A (Intron)	MEF2A:PHOS(0.601)PPI(0.761)REG(0.989)		0.967
chr15	100214935	T	C	MEF2A (Intron)	MEF2A:PHOS(0.601)PPI(0.761)REG(0.989)		0.967
chr15	100215596	G	A	MEF2A (Intron)	MEF2A:PHOS(0.601)PPI(0.761)REG(0.989)		0.967
chr5	88119642	T	C	MEF2C (Promoter and UTR)	MEF2C:PPI(0.817)REG(0.985)		1.763
chr7	116417747	G	A	MET (Intron)	MET:PHOS(0.717)PPI(0.923)REG(0.409)		2.589
chr7	116417683	T	G	MET (Intron)	MET:PHOS(0.717)PPI(0.923)REG(0.409)		1.966
chr7	116399619	G	A	MET (Intron)	MET:PHOS(0.717)PPI(0.923)REG(0.409)		0.781
chr17	74734968	T	G	MFSD11 (Intron), SRSF2 (Promoter)	MFSD11:PPI(0.142)REG(0.761),SRSF2:PPI(0.361)		1.648
chr17	74731884	C	T	MFSD11 (Promoter), SRSF2 (UTR)	MFSD11:PPI(0.142)REG(0.761),SRSF2:PPI(0.361)		3.852
chr17	74730725	C	T	MFSD11 (Promoter), SRSF2 (UTR)	MFSD11:PPI(0.142)REG(0.761),SRSF2:PPI(0.361)	MOTIFG=ZEB1_known1#74730721#74730727#+#5#6794#3506	3.248
chr17	74732149	C	G	MFSD11 (Promoter), SRSF2 (UTR)	MFSD11:PPI(0.142)REG(0.761),SRSF2:PPI(0.361)		2.260
chr17	74734279	C	G	MFSD11 (UTR), SRSF2 (Promoter)	MFSD11:PPI(0.142)REG(0.761),SRSF2:PPI(0.361)		2.260
chr3	69998084	G	A	MITF (Intron)	MITF:PHOS(0.769)PPI(0.707)REG(0.409)		0.660
chr3	158310389	G	C	MLF1 (Intron)	MLF1:PPI(0.583)REG(0.634)		0.307

chr3	37058852	T	G	MLH1 (Intron)	MLH1:PPI(0.930)REG(0.880)		0.799
chr3	37090170	A	C	MLH1 (Intron)	MLH1:PPI(0.930)REG(0.880)		0.799
chr3	37058893	G	T	MLH1 (Intron)	MLH1:PPI(0.930)REG(0.880)		0.799
chr3	37090130	C	T	MLH1 (Intron)	MLH1:PPI(0.930)REG(0.880)		0.799
chr3	37092142	T	A	MLH1 (UTR)	MLH1:PPI(0.930)REG(0.880)		0.799
chr10	22016857	G	C	MLLT10 (Intron)	MLLT10:PPI(0.671)REG(0.634)		1.154
chr10	21971050	G	C	MLLT10 (Intron)	MLLT10:PPI(0.671)REG(0.634)		0.969
chr10	21827677	T	G	MLLT10 (Intron)	MLLT10:PPI(0.671)REG(0.634)		0.531
chr9	20353597	G	A	MLLT3 (Intron)	MLLT3:PPI(0.641)REG(0.409)		2.122
chr9	20353714	C	G	MLLT3 (Intron)	MLLT3:PPI(0.641)REG(0.409)		2.111
chr9	20622269	G	A	MLLT3 (Promoter and UTR)	MLLT3:PPI(0.641)REG(0.409)		1.734
chr6	168280961	C	G	MLLT4 (Intron and Promoter)	MLLT4:PHOS(0.398)PPI(0.895)		1.336
chr6	168323403	G	A	MLLT4 (Intron)	MLLT4:PHOS(0.398)PPI(0.895)		0.714
chr6	168344026	C	T	MLLT4 (Intron)	MLLT4:PHOS(0.398)PPI(0.895)		0.714
chr6	168303191	G	T	MLLT4 (Intron)	MLLT4:PHOS(0.398)PPI(0.895)		0.714
chr6	168226763	C	T	MLLT4 (Promoter)	MLLT4:PHOS(0.398)PPI(0.895)		1.899
chr6	168226812	C	G	MLLT4 (Promoter)	MLLT4:PHOS(0.398)PPI(0.895)		1.899
chr6	168323687	G	T	MLLT4 (UTR)	MLLT4:PHOS(0.398)PPI(0.895)		0.714
chr17	36868057	C	A	MLLT6 (Intron)	MLLT6:PPI(0.361)REG(0.634)		0.492
chr10	99240647	G	A	MMS19 (Intron)	MMS19:PPI(0.955)REG(0.409)		1.674
chr10	99218509	G	T	MMS19 (Intron)	MMS19:PPI(0.955)REG(0.409)		1.052
chr15	79177235	G	T	MORF4L1 (Intron)	MORF4L1:PHOS(0.276)PPI(0.951)		1.478
chr11	94212048	C	T	MRE11A (Intron)	MRE11A:PHOS(0.693)PPI(0.970)		1.532
chr11	94179125	T	C	MRE11A (Intron)	MRE11A:PHOS(0.693)PPI(0.970)		1.095
chr11	94203868	A	T	MRE11A (Intron)	MRE11A:PHOS(0.693)PPI(0.970)		0.909
chr11	94212154	A	G	MRE11A (Intron)	MRE11A:PHOS(0.693)PPI(0.970)		0.909
chr2	47703500	T	C	MSH2 (Intron)	MSH2:PHOS(0.398)PPI(0.941)REG(0.880)		1.451
chr5	80074489	G	C	MSH3 (Intron)	MSH3:PPI(0.850)REG(0.830)		1.240
chr5	80109666	C	T	MSH3 (Intron)	MSH3:PPI(0.850)REG(0.830)		0.617
chr5	79960955	G	A	MSH3 (Intron)	MSH3:PPI(0.850)REG(0.830)		0.617
chr1	76356648	G	A	MSH4 (Intron)	MSH4:PPI(0.729)REG(0.634)		1.040
chr1	76272837	A	G	MSH4 (Intron)	MSH4:PPI(0.729)REG(0.634)		1.040
chr1	76345697	G	A	MSH4 (Intron)	MSH4:PPI(0.729)REG(0.634)		0.602
chr1	76345569	G	T	MSH4 (Intron)	MSH4:PPI(0.729)REG(0.634)		0.417
chr1	76260655	A	G	MSH4 (Promoter)	MSH4:PPI(0.729)REG(0.634),RABGGTB:PPI(0.814)REG(0.761)		0.734
chr2	47958612	C	A	MSH6 (Intron)	MSH6:PPI(0.908)REG(0.409)		1.123

chr2	48035673	A	G	MSH6 (UTR)	MSH6:PPI(0.908)REG(0.409)		0.744
chr17	55334658	C	G	MSI2 (Intron and Medial)	MSI2:PPI(0.671)REG(0.409)		1.154
chr17	55729577	G	C	MSI2 (Intron)	MSI2:PPI(0.671)REG(0.409)		1.154
chr17	55607136	C	G	MSI2 (Intron)	MSI2:PPI(0.671)REG(0.409)		0.346
chrX	64954987	C	T	MSN (Intron)	MSN:PHOS(0.398)PPI(0.866)		0.650
chr1	11319488	G	A	MTOR (Intron)	MTOR:PPI(0.718)REG(0.909)		1.369
chr1	11188906	C	T	MTOR (Intron)	MTOR:PPI(0.718)REG(0.909)		1.369
chr1	11293621	A	C	MTOR (Intron)	MTOR:PPI(0.718)REG(0.909)		1.369
chr1	11319181	T	G	MTOR (Intron)	MTOR:PPI(0.718)REG(0.909)		1.265
chr1	11217115	G	T	MTOR (Intron)	MTOR:PPI(0.718)REG(0.909)		0.932
chr1	11292473	C	T	MTOR (Intron)	MTOR:PPI(0.718)REG(0.909)		0.932
chr1	11318503	T	G	MTOR (Intron)	MTOR:PPI(0.718)REG(0.909)		0.747
chr1	11288580	C	A	MTOR (Intron)	MTOR:PPI(0.718)REG(0.909)		0.747
chr1	11292408	T	G	MTOR (Intron)	MTOR:PPI(0.718)REG(0.909)		0.747
chr1	11318486	T	G	MTOR (Intron)	MTOR:PPI(0.718)REG(0.909)		0.747
chr1	11181582	G	A	MTOR (Intron)	MTOR:PPI(0.718)REG(0.909)		0.747
chr1	11188191	A	T	MTOR (Intron)	MTOR:PPI(0.718)REG(0.909)		0.747
chr1	11190018	G	A	MTOR (Intron)	MTOR:PPI(0.718)REG(0.909)		0.747
chr1	11184539	C	T	MTOR (Intron)	MTOR:PPI(0.718)REG(0.909)		0.747
chr1	11169890	T	C	MTOR (Intron)	MTOR:PPI(0.718)REG(0.909)		0.747
chr19	1372979	T	G	MUM1 (Intron)			1.188
chr19	1373009	G	A	MUM1 (Intron)			1.188
chr11	65633368	G	A	MUS81 (UTR)	MUS81:PPI(0.927)REG(0.880)		1.599
chr1	45800215	G	A	MUTYH (Intron and Promoter)	MUTYH:PPI(0.448)REG(0.634)		0.492
chr1	45797085	C	G	MUTYH (Intron)	MUTYH:PPI(0.448)REG(0.634)		1.115
chr1	45807108	A	T	MUTYH (Promoter)	MUTYH:PPI(0.448)REG(0.634),TOE1:PHOS(0.693)PPI(0.641)		1.169
chr6	135511176	T	G	MYB (Intron)	MYB:PPI(0.826)		1.379
chr16	15850195	T	G	MYH11 (Intron)	MYH11:PPI(0.408)		2.568
chr16	15850195	T	C	MYH11 (Intron)	MYH11:PPI(0.408)		2.568
chr16	15847245	C	T	MYH11 (Intron)	MYH11:PPI(0.408)		0.956
chr16	15838871	C	T	MYH11 (Intron)	MYH11:PPI(0.408)		0.333
chr16	15841421	C	A	MYH11 (Intron)	MYH11:PPI(0.408)		0.333
chr16	15892482	A	C	MYH11 (Intron)	MYH11:PPI(0.408)		0.148
chr16	15831186	G	A	MYH11 (Intron)	MYH11:PPI(0.408)		0.148
chr16	15854537	C	T	MYH11 (Intron)	MYH11:PPI(0.408)		0.148
chr16	15857527	A	T	MYH11 (Intron)	MYH11:PPI(0.408)		0.148
chr16	15841597	G	A	MYH11 (Intron)	MYH11:PPI(0.408)		0.148

chr16	15813909	T	G	MYH11 (Intron)	MYH11:PPI(0.408),NDE1:PPI(0.935)REG(0.409)		0.997
chr22	36737237	C	T	MYH9 (Intron)	MYH9:PPI(0.872)REG(0.830)		1.285
chr22	36694846	A	G	MYH9 (Intron)	MYH9:PPI(0.872)REG(0.830)		0.848
chr22	36710183	T	C	MYH9 (Intron)	MYH9:PPI(0.872)REG(0.830)		0.848
chr22	36680349	G	A	MYH9 (Intron)	MYH9:PPI(0.872)REG(0.830)		0.848
chr22	36691795	G	C	MYH9 (Intron)	MYH9:PPI(0.872)REG(0.830)		0.662
chr22	36687930	C	T	MYH9 (Intron)	MYH9:PPI(0.872)REG(0.830)		0.662
chr22	36714242	G	C	MYH9 (Intron)	MYH9:PPI(0.872)REG(0.830)		0.662
chr12	57108231	G	C	NACA (Intron)	NACA:PPI(0.482)REG(0.761)		2.271
chr12	57106810	G	A	NACA (Intron)	NACA:PPI(0.482)REG(0.761)		1.271
chr12	57125557	G	C	NACA (Promoter)	NACA:PPI(0.482)REG(0.761),PRIM1:PPI(0.956)		0.905
chr12	57108485	C	A	NACA (UTR)	NACA:PPI(0.482)REG(0.761)		1.648
chr12	7947017	T	C	NANOG (Intron)	NANOG:PPI(0.515)REG(0.970)		1.095
chr19	50865578	G	T	NAPSA (Intron), NR1H2 (Intron)	NAPSA:PPI(0.482)REG(0.409),NR1H2:PPI(0.846) REG(0.830)		0.794
chr1	16902372	G	C	NBPF1 (Intron and Promoter)			
chr1	16902439	C	G	NBPF1 (Intron and Promoter)			
chr1	16902449	C	A	NBPF1 (Intron and Promoter)			
chr1	16902416	C	T	NBPF1 (Intron and Promoter)			
chr1	16902566	C	A	NBPF1 (Intron and Promoter)			
chr1	16902590	C	A	NBPF1 (Intron and Promoter)			
chr1	16930598	G	T	NBPF1 (Intron)			
chr1	16894034	G	C	NBPF1 (Intron)			
chr1	16894095	G	C	NBPF1 (Intron)			
chr1	16894052	C	T	NBPF1 (Intron)			
chr1	16894087	A	G	NBPF1 (Intron)			
chr1	16890855	G	A	NBPF1 (Intron)			
chr1	16933219	G	A	NBPF1 (Intron)			
chr1	16921749	T	C	NBPF1 (Intron)			
chr1	16927619	C	G	NBPF1 (Intron)			
chr1	16894181	A	G	NBPF1 (Intron)			
chr1	16890737	C	A	NBPF1 (Intron)			

chr1	16890771	G	A	NBPF1 (Intron)			
chr1	16936965	T	C	NBPF1 (Intron)			
chr1	16938292	G	A	NBPF1 (Intron)			
chr1	16938308	C	T	NBPF1 (Intron)			
chr1	16930210	G	C	NBPF1 (Intron)			
chr1	16922127	C	G	NBPF1 (Intron)			
chr1	16889985	T	C	NBPF1 (UTR)			
chr1	16890070	G	C	NBPF1 (UTR)			
chr1	16890189	A	G	NBPF1 (UTR)			
chr17	41345357	C	G	NBR1 (Intron)	NBR1:PHOS(0.276)PPI(0.448)REG(0.830)		0.578
chr17	41341211	A	C	NBR1 (Intron)	NBR1:PHOS(0.276)PPI(0.448)REG(0.830)		0.578
chr17	41343208	T	G	NBR1 (Intron)	NBR1:PHOS(0.276)PPI(0.448)REG(0.830)		0.578
chr8	71060455	G	C	NCOA2 (Intron and Medial)	NCOA2:PHOS(0.509)PPI(0.875)		2.089
chr8	71060466	T	C	NCOA2 (Intron and Medial)	NCOA2:PHOS(0.509)PPI(0.875)		2.089
chr8	71126133	C	T	NCOA2 (Intron)	NCOA2:PHOS(0.509)PPI(0.875)		1.477
chr8	71075695	T	A	NCOA2 (Intron)	NCOA2:PHOS(0.509)PPI(0.875)		1.292
chr20	46277674	T	G	NCOA3 (Intron)	NCOA3:PPI(0.914)REG(0.634)		1.556
chr20	46251081	G	A	NCOA3 (Intron)	NCOA3:PPI(0.914)REG(0.634)		1.556
chr20	46255926	C	T	NCOA3 (Intron)	NCOA3:PPI(0.914)REG(0.634)		0.759
chr20	46282355	T	C	NCOA3 (UTR)	NCOA3:PPI(0.914)REG(0.634)		0.759
chr20	46284588	C	T	NCOA3 (UTR)	NCOA3:PPI(0.914)REG(0.634)		0.759
chr20	33370246	G	A	NCOA6 (Intron)	NCOA6:PPI(0.932)REG(0.409)		1.427
chr20	33370220	G	C	NCOA6 (Intron)	NCOA6:PPI(0.932)REG(0.409)		1.427
chr20	33369794	C	T	NCOA6 (Intron)	NCOA6:PPI(0.932)REG(0.409)		0.804
chr17	15983589	C	T	NCOR1 (Intron and Promoter)	NCOR1:PPI(0.980)		0.939
chr17	15983410	A	C	NCOR1 (Intron and Promoter)	NCOR1:PPI(0.980)		0.939
chr17	16075115	A	G	NCOR1 (Intron)	NCOR1:PPI(0.980)	MOTIFBR=FOXA1#Foxa_known4_8mer#16075101#16075116#-#1#0230769#0461538,FOXA2#Foxa_known4_8mer#16075101#16075116#-#1#0230769#0461538	3.509
chr17	16053003	G	A	NCOR1 (Intron)	NCOR1:PPI(0.980)		1.562
chr17	16042501	C	A	NCOR1 (Intron)	NCOR1:PPI(0.980)		1.562

chr17	15968797	A	G	NCOR1 (Intron)	NCOR1:PPI(0.980)		1.562
chr17	15973477	A	G	NCOR1 (Intron)	NCOR1:PPI(0.980)		1.562
chr17	15969076	G	A	NCOR1 (Intron)	NCOR1:PPI(0.980)		1.124
chr17	16041529	G	T	NCOR1 (Intron)	NCOR1:PPI(0.980)		0.939
chr17	15973914	C	A	NCOR1 (Intron)	NCOR1:PPI(0.980)		0.939
chr8	134296659	C	T	NDRG1 (Intron)	NDRG1:PPI(0.908)REG(0.830)		0.744
chr15	75646294	C	G	NEIL1 (Intron)	NEIL1:PHOS(0.398)PPI(0.307)REG(0.409)		0.148
chr17	29684678	C	A	NF1 (Intron and Promoter)	NF1:PPI(0.774)REG(0.409)	MOTIFBR=MAFF#Maf_known4_8mer#29684676#29684693#-#15#0110132#0242224,MAFK#Maf_known4_8mer#29684676#29684693#-#15#0110132#0242224	1.458
chr17	29527434	C	A	NF1 (Intron and Promoter)	NF1:PPI(0.774)REG(0.409)		1.291
chr17	29684279	C	G	NF1 (Intron and Promoter)	NF1:PPI(0.774)REG(0.409)		1.291
chr17	29684425	C	G	NF1 (Intron and Promoter)	NF1:PPI(0.774)REG(0.409)		1.105
chr17	29684638	C	A	NF1 (Intron and Promoter)	NF1:PPI(0.774)REG(0.409)		0.668
chr17	29562484	C	T	NF1 (Intron)	NF1:PPI(0.774)REG(0.409)		1.291
chr17	29587328	C	T	NF1 (Intron)	NF1:PPI(0.774)REG(0.409)		1.291
chr17	29585350	C	G	NF1 (Intron)	NF1:PPI(0.774)REG(0.409)		1.105
chr17	29562791	G	T	NF1 (Intron)	NF1:PPI(0.774)REG(0.409)		1.105
chr17	29563041	T	C	NF1 (Intron)	NF1:PPI(0.774)REG(0.409)		1.105
chr17	29579808	A	G	NF1 (Intron)	NF1:PPI(0.774)REG(0.409)		1.105
chr17	29654472	G	C	NF1 (Intron)	NF1:PPI(0.774)REG(0.409)		0.668
chr17	29667684	G	T	NF1 (Intron)	NF1:PPI(0.774)REG(0.409)		0.668
chr17	29672998	A	G	NF1 (Intron)	NF1:PPI(0.774)REG(0.409)		0.668
chr17	29527838	T	C	NF1 (Intron)	NF1:PPI(0.774)REG(0.409)		0.483
chr17	29482986	T	G	NF1 (Intron)	NF1:PPI(0.774)REG(0.409)		0.483
chr17	29558082	T	C	NF1 (Intron)	NF1:PPI(0.774)REG(0.409)		0.483
chr17	29509435	C	T	NF1 (Intron)	NF1:PPI(0.774)REG(0.409)		0.483
chr17	29556772	G	T	NF1 (Intron)	NF1:PPI(0.774)REG(0.409)		0.483
chr17	29587668	C	A	NF1 (Intron)	NF1:PPI(0.774)REG(0.409)		0.483
chr17	29490045	T	C	NF1 (Intron)	NF1:PPI(0.774)REG(0.409)		0.483
chr22	30054096	T	G	NF2 (Intron)	NF2:PPI(0.964)REG(0.409)		1.892
chr22	30054096	T	G	NF2 (Intron)	NF2:PPI(0.964)REG(0.409)		1.892
chr22	30054436	G	C	NF2 (Intron)	NF2:PPI(0.964)REG(0.409)		1.515
chr22	30053976	G	A	NF2 (Intron)	NF2:PPI(0.964)REG(0.409)		1.077

chr2	178099088	G	T	NFE2L2 (Intron and Promoter)	NFE2L2:PPI(0.761)REG(0.969)		1.522
chr9	14125459	C	A	NFIB (Intron)	NFIB:PPI(0.361)REG(0.409)		0.148
chr4	103514532	T	G	NFKB1 (Intron)	NFKB1:PHOS(0.276)PPI(0.990)REG(0.992)		1.599
chr6	41059458	T	G	NFYA (Intron)	NFYA:PHOS(0.276)PPI(0.826)REG(0.996)		0.989
chr6	41051988	G	T	NFYA (Intron)	NFYA:PHOS(0.276)PPI(0.826)REG(0.996)		0.989
chr6	41039395	G	T	NFYA (Promoter)	NFYA:PHOS(0.276)PPI(0.826)REG(0.996)		1.786
chr14	51205047	A	C	NIN (Intron)	NIN:PPI(0.685)		1.362
chr14	51205047	A	C	NIN (Intron)	NIN:PPI(0.685)		1.362
chr14	51221453	G	A	NIN (Intron)	NIN:PPI(0.685)		0.362
chr14	51218975	C	G	NIN (Intron)	NIN:PPI(0.685)		0.362
chr14	51227118	C	A	NIN (Intron)	NIN:PPI(0.685)		0.362
chr14	51194219	C	G	NIN (UTR)	NIN:PPI(0.685)		0.985
chr20	25436306	C	T	NINL (Intron)	NINL:PPI(0.822)		1.372
chr20	25490269	C	T	NINL (Intron)	NINL:PPI(0.822)		0.749
chr20	25448162	A	C	NINL (Intron)	NINL:PPI(0.822)		0.564
chr20	25433536	T	C	NINL (UTR)	NINL:PPI(0.822)		1.372
chr17	49238971	C	T	NME1 (UTR)	NME1:PPI(0.563)REG(0.929)		0.982
chrX	70517644	C	T	NONO (Intron)	NONO:PHOS(0.398)PPI(0.831)		1.203
chr1	120495990	T	G	NOTCH2 (Intron)	NOTCH2:PPI(0.841)		1.407
chr1	120510602	T	G	NOTCH2 (Intron)	NOTCH2:PPI(0.841)		1.396
chr1	120529828	A	C	NOTCH2 (Intron)	NOTCH2:PPI(0.841)		1.396
chr1	120467701	T	G	NOTCH2 (Intron)	NOTCH2:PPI(0.841)		1.222
chr1	120465619	A	C	NOTCH2 (Intron)	NOTCH2:PPI(0.841)		1.222
chr1	120471904	A	C	NOTCH2 (Intron)	NOTCH2:PPI(0.841)		0.849
chr1	120463144	A	C	NOTCH2 (Intron)	NOTCH2:PPI(0.841)		0.784
chr1	120463160	A	C	NOTCH2 (Intron)	NOTCH2:PPI(0.841)		0.784
chr1	120497886	T	C	NOTCH2 (Intron)	NOTCH2:PPI(0.841)		0.784
chr1	120459339	G	A	NOTCH2 (Intron)	NOTCH2:PPI(0.841)		0.784
chr1	120506483	A	C	NOTCH2 (Intron)	NOTCH2:PPI(0.841)		0.599
chr1	120464504	A	C	NOTCH2 (Intron)	NOTCH2:PPI(0.841)		0.599
chr1	120508192	C	G	NOTCH2 (Intron)	NOTCH2:PPI(0.841)		0.599
chr1	120467787	T	G	NOTCH2 (Intron)	NOTCH2:PPI(0.841)		0.599
chr1	120479788	T	G	NOTCH2 (Intron)	NOTCH2:PPI(0.841)		0.599
chr1	120496444	A	C	NOTCH2 (Intron)	NOTCH2:PPI(0.841)		0.599
chr1	120507891	T	G	NOTCH2 (Intron)	NOTCH2:PPI(0.841)		0.599

chr1	120507904	T	G	NOTCH2 (Intron)	NOTCH2:PPI(0.841)		0.599
chr1	120460241	G	A	NOTCH2 (Intron)	NOTCH2:PPI(0.841)		0.599
chr1	120509843	G	A	NOTCH2 (Intron)	NOTCH2:PPI(0.841)		0.599
chr1	120612343	G	A	NOTCH2 (Promoter)	NOTCH2:PPI(0.841)		0.784
chr1	120457861	G	A	NOTCH2 (UTR)	NOTCH2:PPI(0.841)		1.222
chr19	50886181	C	G	NR1H2 (UTR)	NR1H2:PPI(0.846)REG(0.830),POLD1:PPI(0.925) REG(0.634)		0.971
chr3	15057656	C	G	NR2C2 (Intron)	NR2C2:PPI(0.739)REG(0.998)		1.618
chr3	15065743	C	T	NR2C2 (Intron)	NR2C2:PPI(0.739)REG(0.998)		1.181
chr5	142662130	T	A	NR3C1 (Intron)	NR3C1:PPI(0.979)REG(0.998)		1.618
chr5	142678385	A	G	NR3C1 (Intron)	NR3C1:PPI(0.979)REG(0.998)		0.996
chr5	176675126	T	G	NSD1 (Intron)	NSD1:PPI(0.482)		1.811
chr5	176675126	T	G	NSD1 (Intron)	NSD1:PPI(0.482)		1.811
chr5	176675126	T	G	NSD1 (Intron)	NSD1:PPI(0.482)		1.811
chr5	176709636	T	C	NSD1 (Intron)	NSD1:PPI(0.482)		1.188
chr5	176709636	T	C	NSD1 (Intron)	NSD1:PPI(0.482)		1.188
chr5	176675061	G	A	NSD1 (Intron)	NSD1:PPI(0.482)		0.996
chr5	176618877	G	T	NSD1 (Intron)	NSD1:PPI(0.482)		0.811
chr5	176671408	C	A	NSD1 (Intron)	NSD1:PPI(0.482)		0.373
chr5	176673823	A	C	NSD1 (Intron)	NSD1:PPI(0.482)		0.373
chr5	176707336	T	G	NSD1 (Intron)	NSD1:PPI(0.482)		0.188
chr5	176711080	A	C	NSD1 (Intron)	NSD1:PPI(0.482)		0.188
chr5	176700664	T	C	NSD1 (Intron)	NSD1:PPI(0.482)		0.188
chr16	27236579	A	C	NSMCE1 (Intron)	NSMCE1:PPI(0.606)REG(0.634)		0.492
chr10	104855741	G	A	NT5C2 (Intron and Promoter)	NT5C2:PPI(0.448)		0.168
chr10	104865623	T	C	NT5C2 (Intron)	NT5C2:PPI(0.448)		0.168
chr10	104899383	A	G	NT5C2 (Intron)	NT5C2:PPI(0.448)		0.168
chr16	2090326	A	C	NTHL1 (Intron)	NTHL1:PHOS(0.459)PPI(0.718)REG(0.909)		0.932
chr16	2098871	C	T	NTHL1 (Promoter)	NTHL1:PHOS(0.459)PPI(0.718)REG(0.909),TSC2: PPI(0.838)REG(0.909)		1.555
chr9	87366895	C	G	NTRK2 (Intron)	NTRK2:PHOS(0.868)PPI(0.809)		0.839
chr9	87323155	A	G	NTRK2 (Intron)	NTRK2:PHOS(0.868)PPI(0.809)		0.654
chr15	88678186	T	G	NTRK3 (Intron)	NTRK3:PHOS(0.942)PPI(0.685)		2.016
chr15	88678186	T	G	NTRK3 (Intron)	NTRK3:PHOS(0.942)PPI(0.685)		2.016
chr11	71730455	T	G	NUMA1 (Intron)	NUMA1:PPI(0.718)REG(0.409)		1.588
chr11	71730455	T	G	NUMA1 (Intron)	NUMA1:PPI(0.718)REG(0.409)		1.588
chr11	71733514	C	T	NUMA1 (Intron)	NUMA1:PPI(0.718)REG(0.409)		0.403

chr9	134011251	T	G	NUP214 (Intron and Promoter)	NUP214:PPI(0.875)REG(0.987)	MOTIFG=ZEB1_known1#134011246#134011252#-#1#6794#3667	3.732
chr9	134064345	T	C	NUP214 (Intron and Promoter)	NUP214:PPI(0.875)REG(0.987)		2.381
chr9	134053542	C	A	NUP214 (Intron)	NUP214:PPI(0.875)REG(0.987)		2.727
chr9	134022731	T	G	NUP214 (Intron)	NUP214:PPI(0.875)REG(0.987)		1.758
chr9	134062848	A	C	NUP214 (Intron)	NUP214:PPI(0.875)REG(0.987)		1.758
chr9	134008183	T	G	NUP214 (Intron)	NUP214:PPI(0.875)REG(0.987)		1.758
chr9	134016121	C	T	NUP214 (Intron)	NUP214:PPI(0.875)REG(0.987)		1.758
chr9	134001019	C	T	NUP214 (Promoter and UTR)	NUP214:PPI(0.875)REG(0.987)		3.381
chr9	134001029	G	A	NUP214 (Promoter and UTR)	NUP214:PPI(0.875)REG(0.987)		2.763
chr17	5323021	C	T	NUP88 (Promoter and UTR), RPAIN (Promoter and UTR)	NUP88:PPI(0.895),RPAIN:PPI(0.236)	MOTIFBR=NFYA#NF-Y_known1_8mer#5323018#5323034#+#4#0025862#0120690,NFYB#NF-Y_known1_8mer#5323018#5323034#+#4#0025862#0120690	3.311
chr17	5323631	A	G	NUP88 (Promoter), RPAIN (Intron)	NUP88:PPI(0.895),RPAIN:PPI(0.236)		2.511
chr11	3790044	A	C	NUP98 (Intron and Medial)	NUP98:PPI(0.994)REG(0.634)		1.168
chr11	3701050	C	T	NUP98 (Intron and Promoter)	NUP98:PPI(0.994)REG(0.634)		0.983
chr11	3734011	A	C	NUP98 (Intron)	NUP98:PPI(0.994)REG(0.634)		2.168
chr11	3734087	A	G	NUP98 (Intron)	NUP98:PPI(0.994)REG(0.634)		2.168
chr11	3800096	C	T	NUP98 (Intron)	NUP98:PPI(0.994)REG(0.634)		1.791
chr11	3803165	A	C	NUP98 (Intron)	NUP98:PPI(0.994)REG(0.634)		1.606
chr11	3774448	C	A	NUP98 (Intron)	NUP98:PPI(0.994)REG(0.634)		1.168
chr11	3746529	G	C	NUP98 (Intron)	NUP98:PPI(0.994)REG(0.634)		0.983
chr11	3723545	T	A	NUP98 (Intron)	NUP98:PPI(0.994)REG(0.634)		0.983
chr11	3720709	A	C	NUP98 (Intron)	NUP98:PPI(0.994)REG(0.634)		0.983
chr11	3752911	A	C	NUP98 (Intron)	NUP98:PPI(0.994)REG(0.634)		0.983
chr11	3746284	G	A	NUP98 (Intron)	NUP98:PPI(0.994)REG(0.634)		0.983
chr11	3720237	A	G	NUP98 (Intron)	NUP98:PPI(0.994)REG(0.634)		0.983
chr11	3723547	T	A	NUP98 (Intron)	NUP98:PPI(0.994)REG(0.634)		0.983
chr11	3714611	C	T	NUP98 (Intron)	NUP98:PPI(0.994)REG(0.634)		0.983
chr11	3697271	A	G	NUP98 (UTR)	NUP98:PPI(0.994)REG(0.634)		1.168

chr1	228465370	T	G	OBSCN (Intron and Medial)	OBSCN:PPI(0.482)		0.373
chr1	228461757	G	A	OBSCN (Intron and Medial)	OBSCN:PPI(0.482)		0.373
chr1	228432987	G	C	OBSCN (Intron and Medial)	OBSCN:PPI(0.482)		1.373
chr1	228433071	G	T	OBSCN (Intron and Medial)	OBSCN:PPI(0.482)		1.373
chr1	228431876	G	A	OBSCN (Intron and Medial)	OBSCN:PPI(0.482)		1.373
chr1	228431930	C	T	OBSCN (Intron and Medial)	OBSCN:PPI(0.482)		1.373
chr1	228464468	G	A	OBSCN (Intron and Promoter)	OBSCN:PPI(0.482)		1.373
chr1	228464463	G	A	OBSCN (Intron and Promoter)	OBSCN:PPI(0.482)		1.373
chr1	228444297	T	G	OBSCN (Intron and Promoter)	OBSCN:PPI(0.482)		0.811
chr1	228464842	C	G	OBSCN (Intron and Promoter)	OBSCN:PPI(0.482)		0.373
chr1	228520809	G	T	OBSCN (Intron and Promoter)	OBSCN:PPI(0.482)		0.188
chr1	228557595	C	G	OBSCN (Intron)	OBSCN:PPI(0.482)		1.985
chr1	228557816	C	T	OBSCN (Intron)	OBSCN:PPI(0.482)		1.373
chr1	228558362	C	G	OBSCN (Intron)	OBSCN:PPI(0.482)		1.373
chr1	228543818	G	A	OBSCN (Intron)	OBSCN:PPI(0.482)		0.996
chr1	228565185	C	T	OBSCN (Intron)	OBSCN:PPI(0.482)		0.996
chr1	228544071	A	C	OBSCN (Intron)	OBSCN:PPI(0.482)		0.995
chr1	228412555	C	T	OBSCN (Intron)	OBSCN:PPI(0.482)		0.397
chr1	228491701	A	C	OBSCN (Intron)	OBSCN:PPI(0.482)		0.373
chr1	228527844	T	G	OBSCN (Intron)	OBSCN:PPI(0.482)		0.373
chr1	228503907	G	A	OBSCN (Intron)	OBSCN:PPI(0.482)		0.373
chr1	228456669	G	A	OBSCN (Intron)	OBSCN:PPI(0.482)		0.373
chr1	228451712	T	G	OBSCN (Intron)	OBSCN:PPI(0.482)		0.373
chr1	228482285	T	C	OBSCN (Intron)	OBSCN:PPI(0.482)		0.373
chr1	228524919	G	A	OBSCN (Intron)	OBSCN:PPI(0.482)		0.373
chr1	228505142	T	C	OBSCN (Intron)	OBSCN:PPI(0.482)		0.188
chr1	228447513	T	G	OBSCN (Intron)	OBSCN:PPI(0.482)		0.188
chr1	228447519	T	A	OBSCN (Intron)	OBSCN:PPI(0.482)		0.188
chr1	228407010	C	T	OBSCN (Intron)	OBSCN:PPI(0.482)		0.188
chr1	228525898	C	G	OBSCN (Intron)	OBSCN:PPI(0.482)		0.188
chr1	228525584	C	T	OBSCN (Intron)	OBSCN:PPI(0.482)		0.188
chr1	228494375	G	A	OBSCN (Intron)	OBSCN:PPI(0.482)		0.188
chr3	9829046	A	C	OGG1 (Intron)	OGG1:PHOS(0.276)PPI(0.761)REG(0.634),TADA3:PPI(0.671)REG(0.830)		0.764

chr3	9791929	C	G	OGG1 (Promoter and UTR)	OGG1:PHOS(0.276)PPI(0.761)REG(0.634)		1.260
chr3	9807889	G	T	OGG1 (UTR)	OGG1:PHOS(0.276)PPI(0.761)REG(0.634)		1.260
chr9	95177241	G	T	OMD (UTR)			0.003
chr5	38945514	C	T	OSMR (Intron), RICTOR (Intron)	OSMR:PPI(0.916)REG(0.409),RICTOR:PPI(0.408)		1.561
chr5	131598453	G	C	P4HA2 (Intron), PDLIM4 (Intron and Medial)	P4HA2:PPI(0.448),PDLIM4:PHOS(0.585)PPI(0.448)		1.070
chrX	110388050	C	T	PAK3 (Intron)	PAK3:PPI(0.814)		1.172
chrX	110437327	C	T	PAK3 (Intron)	PAK3:PPI(0.814)		1.172
chrX	110439046	G	T	PAK3 (Intron)	PAK3:PPI(0.814)		1.172
chrX	110388176	C	G	PAK3 (Intron)	PAK3:PPI(0.814)		1.148
chrX	110435462	C	A	PAK3 (Intron)	PAK3:PPI(0.814)		0.549
chrX	110463789	C	A	PAK3 (UTR)	PAK3:PPI(0.814)		0.549
chr1	226580021	G	T	PARP1 (Intron)	PARP1:PPI(0.927)		1.791
chr1	226580021	G	T	PARP1 (Intron)	PARP1:PPI(0.927)		1.791
chr1	226580021	G	T	PARP1 (Intron)	PARP1:PPI(0.927)		1.791
chr1	226570735	A	G	PARP1 (Intron)	PARP1:PPI(0.927)		1.414
chr1	226573402	T	C	PARP1 (Intron)	PARP1:PPI(0.927)		0.977
chr1	226558296	A	C	PARP1 (Intron)	PARP1:PPI(0.927)		0.977
chr1	226576497	A	C	PARP1 (Intron)	PARP1:PPI(0.927)		0.977
chr1	226564609	T	G	PARP1 (Intron)	PARP1:PPI(0.927)		0.791
chr1	226565110	A	C	PARP1 (Intron)	PARP1:PPI(0.927)		0.791
chr1	226574238	A	C	PARP1 (Intron)	PARP1:PPI(0.927)		0.791
chr1	226549856	C	G	PARP1 (Intron)	PARP1:PPI(0.927)		0.791
chr1	226574342	C	T	PARP1 (Intron)	PARP1:PPI(0.927)		0.791
chr1	226561922	A	C	PARP1 (Intron)	PARP1:PPI(0.927)		0.791
chr1	226576218	G	A	PARP1 (Intron)	PARP1:PPI(0.927)		0.791
chr1	226549089	T	G	PARP1 (UTR)	PARP1:PPI(0.927)		1.791
chr1	226549089	T	G	PARP1 (UTR)	PARP1:PPI(0.927)		1.791
chr14	20813289	G	A	PARP2 (Intron)	PARP2:PPI(0.563)REG(0.909)		1.747
chr14	20813289	G	A	PARP2 (Intron)	PARP2:PPI(0.563)REG(0.909)		1.747
chr14	20813289	G	A	PARP2 (Intron)	PARP2:PPI(0.563)REG(0.909)		1.747
chr14	20813637	G	A	PARP2 (Intron)	PARP2:PPI(0.563)REG(0.909)		1.555
chr14	20814975	G	T	PARP2 (Intron)	PARP2:PPI(0.563)REG(0.909)		0.932
chr14	20815084	G	T	PARP2 (Intron)	PARP2:PPI(0.563)REG(0.909)		0.932
chr14	20812984	G	C	PARP2 (Intron)	PARP2:PPI(0.563)REG(0.909)		0.747
chr14	20824951	G	C	PARP2 (Intron)	PARP2:PPI(0.563)REG(0.909)		0.747
chr14	20813306	G	A	PARP2 (Intron)	PARP2:PPI(0.563)REG(0.909)		0.747

chr3	51980071	G	C	PARP3 (Intron)	PARP3:PHOS(0.398)PPI(0.563)REG(0.634)		0.307
chr3	51981722	G	A	PARP3 (Intron)	PARP3:PHOS(0.398)PPI(0.563)REG(0.634)		0.307
chr3	51979451	G	C	PARP3 (Intron)	PARP3:PHOS(0.398)PPI(0.563)REG(0.634)		0.307
chr3	51980090	G	C	PARP3 (Intron)	PARP3:PHOS(0.398)PPI(0.563)REG(0.634)		0.307
chr13	25016202	T	C	PARP4 (Intron)	PARP4:PPI(0.142)REG(0.409)		0.333
chr13	25060456	G	A	PARP4 (Intron)	PARP4:PPI(0.142)REG(0.409)		0.333
chr13	25077609	C	T	PARP4 (Intron)	PARP4:PPI(0.142)REG(0.409)		0.333
chr13	25066764	G	T	PARP4 (Intron)	PARP4:PPI(0.142)REG(0.409)		0.148
chr13	25016853	C	T	PARP4 (Intron)	PARP4:PPI(0.142)REG(0.409)		0.148
chr13	25052420	T	A	PARP4 (Intron)	PARP4:PPI(0.142)REG(0.409)		0.148
chr13	25065008	A	G	PARP4 (Intron)	PARP4:PPI(0.142)REG(0.409)		0.148
chr9	37014901	G	C	PAX5 (Intron)	PAX5:PPI(0.809)REG(0.992)		1.162
chr1	18961601	G	C	PAX7 (Intron and Medial)	PAX7:PPI(0.482)REG(0.761)		1.271
chr2	114000264	C	T	PAX8 (Intron and Promoter)	PAX8:PPI(0.515)REG(0.634)		0.930
chr3	52661475	C	T	PBRM1 (Intron)	PBRM1:PHOS(0.276)PPI(0.942)REG(0.634)		1.453
chr3	52662814	T	G	PBRM1 (Intron)	PBRM1:PHOS(0.276)PPI(0.942)REG(0.634)		0.831
chr3	52597293	C	G	PBRM1 (Intron)	PBRM1:PHOS(0.276)PPI(0.942)REG(0.634)		1.446
chr3	52595650	T	G	PBRM1 (Intron)	PBRM1:PHOS(0.276)PPI(0.942)REG(0.634)		1.016
chr3	52613002	T	G	PBRM1 (Intron)	PBRM1:PHOS(0.276)PPI(0.942)REG(0.634)		1.016
chr1	164789425	G	T	PBX1 (Intron)	PBX1:PPI(0.850)		1.240
chr9	128725132	G	T	PBX3 (Intron)	PBX3:PPI(0.515)REG(0.997)		1.615
chr8	17819685	T	C	PCM1 (Intron)	PCM1:PHOS(0.634)PPI(0.685)REG(0.880)		1.488
chr8	17820838	A	C	PCM1 (Intron)	PCM1:PHOS(0.634)PPI(0.685)REG(0.880)		0.865
chr8	17824701	G	T	PCM1 (Intron)	PCM1:PHOS(0.634)PPI(0.685)REG(0.880)		0.824
chr8	17797098	C	T	PCM1 (Intron)	PCM1:PHOS(0.634)PPI(0.685)REG(0.880)		0.680
chr8	17814096	A	C	PCM1 (Intron)	PCM1:PHOS(0.634)PPI(0.685)REG(0.880)		0.680

chr8	17848934	T	G	PCM1 (Intron)	PCM1:PHOS(0.634)PPI(0.685)REG(0.880)		0.680
chr8	17849552	T	G	PCM1 (Intron)	PCM1:PHOS(0.634)PPI(0.685)REG(0.880)		0.680
chr8	17868350	C	T	PCM1 (Intron)	PCM1:PHOS(0.634)PPI(0.685)REG(0.880)		0.680
chr11	117098039	C	T	PCSK7 (Intron)	PCSK7:PPI(0.897)		1.341
chr5	149513324	C	G	PDGFRB (Intron)	PDGFRB:PHOS(0.750)PPI(0.962)		1.509
chr19	7696695	C	T	PET100 (UTR), XAB2 (Promoter)	XAB2:PPI(0.893)REG(0.830)		1.332
chr6	83881913	C	T	PGM3 (Intron)	PGM3:PPI(0.606)		0.280
chr11	100933085	A	T	PGR (Intron)	PGR:PPI(0.846)		1.232
chr16	71692272	A	G	PHLPP2 (Intron)			0.055
chr16	71682766	C	T	PHLPP2 (UTR)			0.811
chr11	85737414	A	G	PICALM (Intron)	PICALM:PPI(0.746)REG(0.761)		1.085
chr11	85694862	C	G	PICALM (Intron)	PICALM:PPI(0.746)REG(0.761)		0.463
chr3	178938031	C	G	PIK3CA (Intron)	PIK3CA:PPI(0.957)REG(0.634)		1.495
chr3	178937846	T	G	PIK3CA (Intron)	PIK3CA:PPI(0.957)REG(0.634)		1.495
chr3	178927500	C	T	PIK3CA (Intron)	PIK3CA:PPI(0.957)REG(0.634)		0.872
chr3	178938747	A	T	PIK3CA (Intron)	PIK3CA:PPI(0.957)REG(0.634)		0.872
chr3	178939018	T	C	PIK3CA (Intron)	PIK3CA:PPI(0.957)REG(0.634)		0.872
chr3	178938711	G	C	PIK3CA (Intron)	PIK3CA:PPI(0.957)REG(0.634)		0.872
chr3	178937877	A	G	PIK3CA (Intron)	PIK3CA:PPI(0.957)REG(0.634)		0.872
chr3	178952507	G	T	PIK3CA (UTR)	PIK3CA:PPI(0.957)REG(0.634)		2.495
chr3	178952507	G	T	PIK3CA (UTR)	PIK3CA:PPI(0.957)REG(0.634)		2.495
chr3	178957484	C	T	PIK3CA (UTR)	PIK3CA:PPI(0.957)REG(0.634)		0.872
chr7	106513054	A	G	PIK3CG (Intron and Medial)	PIK3CG:PPI(0.938)REG(0.761)		1.990
chr7	106522660	G	A	PIK3CG (Intron)	PIK3CG:PPI(0.938)REG(0.761)		0.820
chr7	106545845	G	C	PIK3CG (UTR)	PIK3CG:PPI(0.938)REG(0.761)		0.820
chr5	67569613	T	G	PIK3R1 (Intron)	PIK3R1:PPI(0.998)REG(0.761)		1.804
chr5	67569198	T	G	PIK3R1 (Intron)	PIK3R1:PPI(0.998)REG(0.761)		1.618
chr5	67593109	T	G	PIK3R1 (Intron)	PIK3R1:PPI(0.998)REG(0.761)		1.618
chr5	67593467	A	C	PIK3R1 (UTR)	PIK3R1:PPI(0.998)REG(0.761)		1.618
chr6	51919051	A	C	PKHD1 (Intron)	PKHD1:PPI(0.142)		1.682
chr6	51919051	A	C	PKHD1 (Intron)	PKHD1:PPI(0.142)		1.682
chr6	51913240	G	A	PKHD1 (Intron)	PKHD1:PPI(0.142)		1.063
chr6	51913240	G	A	PKHD1 (Intron)	PKHD1:PPI(0.142)		1.063
chr6	51900357	G	C	PKHD1 (Intron)	PKHD1:PPI(0.142)		0.871
chr6	51512917	C	G	PKHD1 (Intron)	PKHD1:PPI(0.142)		0.685

chr6	51949675	C	T	PKHD1 (Intron)	PKHD1:PPI(0.142)		0.685
chr6	51611517	A	G	PKHD1 (Intron)	PKHD1:PPI(0.142)		0.685
chr6	51938068	A	C	PKHD1 (Intron)	PKHD1:PPI(0.142)		0.680
chr6	51909927	G	A	PKHD1 (Intron)	PKHD1:PPI(0.142)		0.492
chr6	51503801	C	T	PKHD1 (Intron)	PKHD1:PPI(0.142)		0.207
chr6	51934157	T	G	PKHD1 (Intron)	PKHD1:PPI(0.142)		0.063
chr6	51513713	G	C	PKHD1 (Intron)	PKHD1:PPI(0.142)		0.063
chr6	51948111	C	G	PKHD1 (Intron)	PKHD1:PPI(0.142)		0.063
chr6	51611733	A	C	PKHD1 (Intron)	PKHD1:PPI(0.142)		0.063
chr6	51611972	G	C	PKHD1 (Intron)	PKHD1:PPI(0.142)		0.063
chr6	51768573	A	G	PKHD1 (Intron)	PKHD1:PPI(0.142)		0.063
chr6	51695570	T	A	PKHD1 (Intron)	PKHD1:PPI(0.142)		0.063
chr6	51586751	C	G	PKHD1 (UTR)	PKHD1:PPI(0.142)		0.063
chr20	39792127	C	T	PLCG1 (Intron)	PLCG1:PPI(0.993)		2.165
chr20	39791808	G	T	PLCG1 (Intron)	PLCG1:PPI(0.993)		2.165
chr20	39793416	C	T	PLCG1 (Intron)	PLCG1:PPI(0.993)		0.980
chr1	45268284	C	T	PLK3 (Intron)	PLK3:PHOS(0.736)PPI(0.540)REG(0.830)		0.764
chr15	74327471	A	T	PML (UTR)	PML:PPI(0.934)REG(0.634)		1.432
chr15	74328401	A	G	PML (UTR)	PML:PPI(0.934)REG(0.634)		0.995
chr2	190717612	C	A	PMS1 (Intron)	PMS1:PPI(0.236)		0.708
chr2	190687062	G	A	PMS1 (Intron)	PMS1:PPI(0.236)		0.275
chr2	190687496	A	G	PMS1 (Intron)	PMS1:PPI(0.236)		0.270
chr2	190656439	C	T	PMS1 (Promoter and UTR)	PMS1:PPI(0.236)		0.270
chr7	6043697	G	T	PMS2 (Intron and Promoter)	PMS2:PHOS(0.509)PPI(0.890)REG(0.880)		0.702
chr7	6038722	T	C	PMS2 (Intron)	PMS2:PHOS(0.509)PPI(0.890)REG(0.880)		0.702
chr7	6043112	C	T	PMS2 (Intron)	PMS2:PHOS(0.509)PPI(0.890)REG(0.880)		0.702
chrX	24744961	G	T	POLA1 (Intron)	POLA1:PPI(0.887)REG(0.409)		1.318
chrX	24722586	G	T	POLA1 (Intron)	POLA1:PPI(0.887)REG(0.409)		1.318
chrX	24742339	G	T	POLA1 (Intron)	POLA1:PPI(0.887)REG(0.409)		1.318
chrX	24733254	C	T	POLA1 (Intron)	POLA1:PPI(0.887)REG(0.409)		1.318
chrX	24807291	G	T	POLA1 (Intron)	POLA1:PPI(0.887)REG(0.409)		0.881
chrX	24742311	G	T	POLA1 (Intron)	POLA1:PPI(0.887)REG(0.409)		0.695
chrX	24759269	C	T	POLA1 (Intron)	POLA1:PPI(0.887)REG(0.409)		0.695
chrX	24741480	A	C	POLA1 (Intron)	POLA1:PPI(0.887)REG(0.409)		0.695
chrX	24751822	A	T	POLA1 (Intron)	POLA1:PPI(0.887)REG(0.409)		0.695
chrX	24906372	A	C	POLA1 (Intron)	POLA1:PPI(0.887)REG(0.409)		0.695

chrX	24750579	T	C	POLA1 (Intron)	POLA1:PPI(0.887)REG(0.409)		0.695
chrX	25014240	T	C	POLA1 (UTR)	POLA1:PPI(0.887)REG(0.409)		0.695
chr8	42196591	G	A	POLB (Intron)	POLB:PHOS(0.585)PPI(0.657)		2.751
chr8	42215068	A	G	POLB (Intron)	POLB:PHOS(0.585)PPI(0.657)		0.953
chr8	42214918	A	T	POLB (Intron)	POLB:PHOS(0.585)PPI(0.657)		0.953
chr8	42199215	A	T	POLB (Intron)	POLB:PHOS(0.585)PPI(0.657)		0.516
chr8	42196046	C	G	POLB (UTR)	POLB:PHOS(0.585)PPI(0.657)		2.751
chr8	42226560	T	A	POLB (UTR)	POLB:PHOS(0.585)PPI(0.657)		0.331
chr7	44157539	C	G	POLD2 (Intron and Promoter)	POLD2:PHOS(0.566)PPI(0.831)		1.388
chr7	44157413	A	C	POLD2 (Intron and Promoter)	POLD2:PHOS(0.566)PPI(0.831)		0.580
chr12	133233849	G	A	POLE (Intron)	POLE:PHOS(0.398)PPI(0.940)REG(0.634)		1.011
chr14	50117262	G	A	POLE2 (Intron)	POLE2:PPI(0.905)		1.545
chr14	50146540	C	T	POLE2 (Intron)	POLE2:PPI(0.905)		1.360
chr14	50146398	C	A	POLE2 (UTR)	POLE2:PPI(0.905)		1.545
chr6	43550722	T	G	POLH (Intron)	POLH:PHOS(0.276)PPI(0.895)REG(0.761)		0.714
chr5	74872601	G	C	POLK (Intron)	POLK:PHOS(0.698)PPI(0.142)REG(0.409)		1.185
chr4	2091611	C	G	POLN (Intron and Medial)			0.188
chr4	2091641	C	G	POLN (Intron and Medial)			0.188
chr4	2130018	A	C	POLN (Intron)			1.188
chr4	2130015	C	T	POLN (Intron)			1.188
chr4	2074890	A	G	POLN (Intron)			0.381
chr4	2090759	C	T	POLN (Intron)			0.188
chr4	2214767	G	A	POLN (Intron)			0.188
chr4	2214673	T	G	POLN (Intron)			0.003
chr4	2175752	A	T	POLN (Intron)			0.003
chr3	121228694	G	A	POLQ (Intron)	POLQ:PPI(0.307)REG(0.409)		0.771
chr3	121228870	G	C	POLQ (Intron)	POLQ:PPI(0.307)REG(0.409)		0.771
chr3	121228881	C	A	POLQ (Intron)	POLQ:PPI(0.307)REG(0.409)		0.771
chr3	121159008	C	T	POLQ (Intron)	POLQ:PPI(0.307)REG(0.409)		0.771
chr3	121213671	C	T	POLQ (Intron)	POLQ:PPI(0.307)REG(0.409)		0.333
chr3	121168276	G	A	POLQ (Intron)	POLQ:PPI(0.307)REG(0.409)		0.148
chr3	121228338	G	A	POLQ (Intron)	POLQ:PPI(0.307)REG(0.409)		0.148
chr3	121186543	A	G	POLQ (Intron)	POLQ:PPI(0.307)REG(0.409)		0.148
chr3	121228833	A	C	POLQ (Intron)	POLQ:PPI(0.307)REG(0.409)		0.148
chr3	121239167	G	C	POLQ (Intron)	POLQ:PPI(0.307)REG(0.409)		0.148

chr3	121265043	C	T	POLQ (Promoter)	POLQ:PPI(0.307)REG(0.409)		1.568
chr3	121151152	T	G	POLQ (UTR)	POLQ:PPI(0.307)REG(0.409)		1.568
chr17	7402556	G	A	POLR2A (Intron)			1.188
chr17	7402352	C	G	POLR2A (Intron)			1.188
chr17	7394968	A	G	POLR2A (Intron)			0.811
chr17	7415701	C	T	POLR2A (Intron)			0.188
chr17	7400614	C	T	POLR2A (Intron)			0.188
chr17	7400390	T	G	POLR2A (Intron)			0.188
chr17	7412212	A	G	POLR2A (Intron)			0.003
chr8	101165702	G	T	POLR2K (UTR)	POLR2K:PHOS(0.566)PPI(0.992)		0.976
chr10	79789070	C	G	POLR3A (Intron)	POLR3A:PPI(0.949)REG(0.988)		1.761
chr10	79784907	C	T	POLR3A (Intron)	POLR3A:PPI(0.949)REG(0.988)		1.587
chr7	124537213	T	C	POT1 (Intron)	POT1:PPI(0.739)		1.054
chr19	42597920	C	T	POU2F2 (UTR)	POU2F2:PPI(0.753)REG(0.991)		0.973
chr11	64700125	G	A	PPP2R5B (Intron)	PPP2R5B:PHOS(0.276)PPI(0.515)REG(0.409)		0.832
chr11	64700721	G	C	PPP2R5B (Intron)	PPP2R5B:PHOS(0.276)PPI(0.515)REG(0.409)		0.832
chr11	64700407	G	C	PPP2R5B (Intron)	PPP2R5B:PHOS(0.276)PPI(0.515)REG(0.409)		0.209
chr11	64700159	G	A	PPP2R5B (Intron)	PPP2R5B:PHOS(0.276)PPI(0.515)REG(0.409)		0.209
chr11	64699449	G	C	PPP2R5B (Intron)	PPP2R5B:PHOS(0.276)PPI(0.515)REG(0.409)		0.209
chr14	102349548	C	A	PPP2R5C (Intron and Promoter)	PPP2R5C:PHOS(0.398)PPI(0.908)		0.744
chr14	102349907	A	G	PPP2R5C (Intron)	PPP2R5C:PHOS(0.398)PPI(0.908)		0.929
chr14	102391434	G	C	PPP2R5C (Intron)	PPP2R5C:PHOS(0.398)PPI(0.908)		0.929
chr14	102285424	T	C	PPP2R5C (Intron)	PPP2R5C:PHOS(0.398)PPI(0.908),TRAF3:PPI(0.885)REG(0.409)		0.929
chr14	63881855	T	G	PPP2R5E (Intron)	PPP2R5E:PHOS(0.276)PPI(0.746)		1.064
chr16	30094063	A	T	PPP4C (Intron)	PPP4C:PHOS(0.651)PPI(0.880)		2.100
chr1	156752048	C	G	PRCC (Intron)	PRCC:PPI(0.307)REG(0.830)		0.578
chr1	156737529	C	T	PRCC (UTR)	PRCC:PPI(0.307)REG(0.830)		1.998
chr1	3216440	A	G	PRDM16 (Intron)	PRDM16:PPI(0.448)		0.168
chr1	3322049	G	A	PRDM16 (Intron)	PRDM16:PPI(0.448)		0.168
chr17	66528778	C	G	PRKAR1A (UTR)	PRKAR1A:PHOS(0.938)PPI(0.912)REG(0.634)		1.443
chr17	66527802	T	G	PRKAR1A (UTR)	PRKAR1A:PHOS(0.938)PPI(0.912)REG(0.634)		0.820

chr19	54387512	A	C	PRKCG (Intron and Medial)	PRKCG:PHOS(0.938)PPI(0.949)	MOTIFG=TCF12_disc1#54387509#54387517#+#4#8740#4745,ZEB1_known1#54387510#54387516#+#3#6794#2424	2.640
chr19	54403482	C	T	PRKCG (Intron)	PRKCG:PHOS(0.938)PPI(0.949)		0.850
chr8	48691393	A	G	PRKDC (Intron and Promoter)	PRKDC:PPI(0.938)REG(0.761)		0.820
chr8	48739210	C	A	PRKDC (Intron)	PRKDC:PPI(0.938)REG(0.761)		1.617
chr8	48845575	C	A	PRKDC (Intron)	PRKDC:PPI(0.938)REG(0.761)		1.443
chr8	48801041	A	C	PRKDC (Intron)	PRKDC:PPI(0.938)REG(0.761)		1.443
chr8	48841743	C	G	PRKDC (Intron)	PRKDC:PPI(0.938)REG(0.761)		1.443
chr8	48825174	G	C	PRKDC (Intron)	PRKDC:PPI(0.938)REG(0.761)		1.443
chr8	48761915	G	A	PRKDC (Intron)	PRKDC:PPI(0.938)REG(0.761)		1.005
chr8	48723754	G	A	PRKDC (Intron)	PRKDC:PPI(0.938)REG(0.761)		1.005
chr8	48847423	G	A	PRKDC (Intron)	PRKDC:PPI(0.938)REG(0.761)		0.820
chr8	48716139	A	C	PRKDC (Intron)	PRKDC:PPI(0.938)REG(0.761)		0.820
chr8	48817699	A	C	PRKDC (Intron)	PRKDC:PPI(0.938)REG(0.761)		0.820
chr8	48741023	C	T	PRKDC (Intron)	PRKDC:PPI(0.938)REG(0.761)		0.820
chr8	48744491	G	C	PRKDC (Intron)	PRKDC:PPI(0.938)REG(0.761)		0.820
chr8	48843556	G	A	PRKDC (Intron)	PRKDC:PPI(0.938)REG(0.761)		0.820
chr8	48770995	A	T	PRKDC (Intron)	PRKDC:PPI(0.938)REG(0.761)		0.820
chr8	48847418	A	G	PRKDC (Intron)	PRKDC:PPI(0.938)REG(0.761)		0.820
chr8	48771357	A	G	PRKDC (Intron)	PRKDC:PPI(0.938)REG(0.761)		0.820
chr8	48841617	C	A	PRKDC (Intron)	PRKDC:PPI(0.938)REG(0.761)		0.820
chr11	60666542	T	G	PRPF19 (Intron and Promoter)	PRPF19:PPI(0.958)REG(0.409)	MOTIFG=SREBP_known2#60666536#60666543#-#1#8589#4194	3.043
chr11	60666784	G	A	PRPF19 (Intron and Promoter)	PRPF19:PPI(0.958)REG(0.409)		2.672
chr17	38142969	T	C	PSMD3 (Intron)	PSMD3:PPI(0.937)REG(0.909)		0.817
chr9	98244061	T	C	PTCH1 (Intron and Promoter)	PTCH1:PPI(0.948)	MOTIFG=Foxa_disc3#98244061#98244069#+#1#9952#5558,GR_disc5#98244061#98244069#+#1#9914#5519	2.453
chr9	98248229	A	C	PTCH1 (Intron)	PTCH1:PPI(0.948)		2.032
chr9	98248229	A	C	PTCH1 (Intron)	PTCH1:PPI(0.948)		2.032
chr9	98238441	C	A	PTCH1 (Intron)	PTCH1:PPI(0.948)		1.470
chr9	98211617	G	T	PTCH1 (Intron)	PTCH1:PPI(0.948)		1.470
chr9	98229391	G	A	PTCH1 (Intron)	PTCH1:PPI(0.948)		0.847

chr1	45307941	C	T	PTCH2 (Intron and Medial)	PTCH2:PPI(0.408)		0.333
chr10	89711873	A	G	PTEN (Intron)	PTEN:PPI(0.482)REG(0.761)		1.271
chr10	89685423	G	T	PTEN (Intron)	PTEN:PPI(0.482)REG(0.761)		1.085
chr10	89725042	A	G	PTEN (Intron)	PTEN:PPI(0.482)REG(0.761)		1.085
chr10	89712018	T	C	PTEN (Intron)	PTEN:PPI(0.482)REG(0.761)		1.085
chr10	89712355	G	T	PTEN (Intron)	PTEN:PPI(0.482)REG(0.761)		0.648
chr10	89693018	T	G	PTEN (Intron)	PTEN:PPI(0.482)REG(0.761)		0.463
chr12	112915655	T	G	PTPN11 (Intron)	PTPN11:PPI(0.981)REG(0.409)		1.565
chr9	8733633	G	T	PTPRD (Intron and Medial)	PTPRD:PPI(0.657)		1.751
chr9	8404763	G	A	PTPRD (Intron)	PTPRD:PPI(0.657)		1.953
chr9	8404763	G	A	PTPRD (Intron)	PTPRD:PPI(0.657)		1.953
chr9	8338792	C	T	PTPRD (Intron)	PTPRD:PPI(0.657)		1.139
chr9	8404701	G	T	PTPRD (Intron)	PTPRD:PPI(0.657)		0.953
chr9	8389124	G	A	PTPRD (Intron)	PTPRD:PPI(0.657)		0.953
chr9	8485756	T	C	PTPRD (Intron)	PTPRD:PPI(0.657)		0.953
chr9	8465361	G	A	PTPRD (Intron)	PTPRD:PPI(0.657)		0.953
chr9	8375931	C	T	PTPRD (Intron)	PTPRD:PPI(0.657)		0.953
chr9	8454419	C	A	PTPRD (Intron)	PTPRD:PPI(0.657)		0.953
chr9	8470969	G	T	PTPRD (Intron)	PTPRD:PPI(0.657)		0.953
chr9	8341085	G	A	PTPRD (Intron)	PTPRD:PPI(0.657)		0.516
chr9	8341993	G	C	PTPRD (Intron)	PTPRD:PPI(0.657)		0.331
chr9	8504101	G	T	PTPRD (Intron)	PTPRD:PPI(0.657)		0.331
chr9	8521623	G	A	PTPRD (Intron)	PTPRD:PPI(0.657)		0.331
chr9	8460222	A	G	PTPRD (Intron)	PTPRD:PPI(0.657)		0.331
chr9	8342093	A	C	PTPRD (Intron)	PTPRD:PPI(0.657)		0.331
chr9	8507150	A	G	PTPRD (Intron)	PTPRD:PPI(0.657)		0.331
chr9	8492743	G	A	PTPRD (Intron)	PTPRD:PPI(0.657)		0.331
chr9	8465316	G	A	PTPRD (Intron)	PTPRD:PPI(0.657)		0.331
chr19	55708834	A	C	PTPRH (Intron)	PTPRH:PPI(0.482)REG(0.409)		1.373
chr19	55708264	C	G	PTPRH (Intron)	PTPRH:PPI(0.482)REG(0.409)		1.373
chr19	55708422	C	G	PTPRH (Intron)	PTPRH:PPI(0.482)REG(0.409)		1.373
chr19	55710008	C	A	PTPRH (Intron)	PTPRH:PPI(0.482)REG(0.409)		0.996
chr19	55698859	G	A	PTPRH (Intron)	PTPRH:PPI(0.482)REG(0.409)		0.373
chr19	55702938	G	T	PTPRH (Intron)	PTPRH:PPI(0.482)REG(0.409)		0.373
chr19	55699702	G	A	PTPRH (Intron)	PTPRH:PPI(0.482)REG(0.409)		0.188
chr5	159855492	T	G	PTTG1 (Intron)	PTTG1:PHOS(0.540)PPI(0.307)		0.243
chr3	8955258	G	T	RAD18 (Intron)	RAD18:PHOS(0.276)PPI(0.857)REG(0.409)		1.254
chr3	8989069	G	T	RAD18 (Intron)	RAD18:PHOS(0.276)PPI(0.857)REG(0.409)		0.816

chr3	8988809	C	T	RAD18 (Intron)	RAD18:PHOS(0.276)PPI(0.857)REG(0.409)		0.631
chr3	8931950	C	T	RAD18 (UTR)	RAD18:PHOS(0.276)PPI(0.857)REG(0.409)		0.631
chr19	13058658	C	G	RAD23A (Intron and Promoter)	RAD23A:PHOS(0.622)PPI(0.883)REG(0.929)		1.594
chr9	110062416	C	T	RAD23B (Intron)	RAD23B:PHOS(0.803)PPI(0.974)REG(0.761)		1.544
chr5	131940358	A	G	RAD50 (Intron)	RAD50:PPI(0.964)REG(0.761)		0.892
chr14	68331659	G	T	RAD51B (Intron)			0.626
chr14	68290372	T	G	RAD51B (Intron)			0.626
chr14	68964032	C	G	RAD51B (Intron)			0.433
chr14	69117387	A	G	RAD51B (Intron)			0.188
chr14	68290426	T	C	RAD51B (Intron)			0.003
chr17	56770326	G	C	RAD51C (Intron and Promoter)	RAD51C:PHOS(0.540)PPI(0.583)REG(0.830),TEX14:REG(0.761)		1.998
chr17	56773834	T	C	RAD51C (Intron)	RAD51C:PHOS(0.540)PPI(0.583)REG(0.830)		1.386
chr17	56811476	C	T	RAD51C (Intron)	RAD51C:PHOS(0.540)PPI(0.583)REG(0.830)		1.201
chr17	56774225	G	T	RAD51C (Intron)	RAD51C:PHOS(0.540)PPI(0.583)REG(0.830)		1.201
chr17	56780487	G	A	RAD51C (Intron)	RAD51C:PHOS(0.540)PPI(0.583)REG(0.830)		0.764
chr17	56769983	G	A	RAD51C (Promoter and UTR)	RAD51C:PHOS(0.540)PPI(0.583)REG(0.830),TEX14:REG(0.761)		1.376
chr17	56811599	C	G	RAD51C (UTR)	RAD51C:PHOS(0.540)PPI(0.583)REG(0.830)		1.386
chr17	56810018	G	A	RAD51C (UTR)	RAD51C:PHOS(0.540)PPI(0.583)REG(0.830)		1.201
chr17	33444177	A	C	RAD51D (Intron)			0.188
chr17	33445707	A	C	RAD51D (Intron)			0.188
chr17	33433863	T	G	RAD51D (Intron)			0.003
chr17	33444300	A	T	RAD51D (Intron)			0.003
chr17	33443764	C	G	RAD51D (Intron)			0.003
chr17	33443786	C	T	RAD51D (Intron)			0.003
chr1	46743953	G	T	RAD54L (UTR)	RAD54L:PPI(0.930)REG(0.830)		1.533
chr12	110959979	C	G	RAD9B (Intron)	RAD9B:PPI(0.606)REG(0.634)		0.930
chr12	110940035	T	G	RAD9B (Promoter and UTR)	RAD9B:PPI(0.606)REG(0.634),VPS29:PPI(0.774)REG(0.634)		2.249

chr3	12627391	A	C	RAF1 (Intron)	RAF1:PHOS(0.786)PPI(0.969)REG(0.830)		1.092
chr3	12627396	A	C	RAF1 (Intron)	RAF1:PHOS(0.786)PPI(0.969)REG(0.830)		1.092
chr9	135984052	C	G	RALGDS (Intron and Promoter)	RALGDS:PPI(0.739)REG(0.761)		0.648
chr9	135982295	C	T	RALGDS (Intron)	RALGDS:PPI(0.739)REG(0.761)		0.648
chr9	135979772	A	C	RALGDS (Intron)	RALGDS:PPI(0.739)REG(0.761)		0.463
chr9	135975880	C	A	RALGDS (Intron)	RALGDS:PPI(0.739)REG(0.761)		0.463
chr5	170330931	C	T	RANBP17 (Intron)			0.188
chr5	170679996	G	C	RANBP17 (Intron)			0.188
chr5	170380886	C	G	RANBP17 (Intron)			0.003
chr5	170626669	G	C	RANBP17 (Intron)			0.003
chr4	99273614	C	G	RAP1GDS1 (Intron)	RAP1GDS1:PPI(0.641)		1.122
chr4	99313099	A	G	RAP1GDS1 (Intron)	RAP1GDS1:PPI(0.641)		1.122
chr4	99182684	C	T	RAP1GDS1 (UTR)	RAP1GDS1:PPI(0.641)		0.499
chr17	38499208	T	G	RARA (Intron and Promoter)	RARA:PPI(0.935)REG(0.929)		1.609
chr17	38473315	G	A	RARA (Intron and Promoter)	RARA:PPI(0.935)REG(0.929)		1.609
chr17	38496871	T	G	RARA (Intron and Promoter)	RARA:PPI(0.935)REG(0.929)		1.435
chr17	38512088	A	C	RARA (Intron)	RARA:PPI(0.935)REG(0.929)		0.997
chr15	79298793	G	A	RASGRF1 (Intron and Promoter)	RASGRF1:PPI(0.767)		0.507
chr15	79350823	G	T	RASGRF1 (Intron)	RASGRF1:PPI(0.767)		1.280
chr15	79312447	C	G	RASGRF1 (Intron)	RASGRF1:PPI(0.767)		1.095
chr15	79282444	A	G	RASGRF1 (Intron)	RASGRF1:PPI(0.767)		1.095
chr15	79382400	C	G	RASGRF1 (Intron)	RASGRF1:PPI(0.767)		0.657
chr15	79307632	A	T	RASGRF1 (Intron)	RASGRF1:PPI(0.767)		0.472
chr15	79356631	A	C	RASGRF1 (Intron)	RASGRF1:PPI(0.767)		0.472
chr3	50379705	C	A	RASSF1 (Promoter)	RASSF1:PPI(0.814)REG(0.409),ZMYND10:PPI(0.236)		0.549
chr13	48939108	G	A	RB1 (Intron)	RB1:PHOS(0.276)PPI(0.988)REG(0.634)		1.587
chr13	48954248	T	G	RB1 (Intron)	RB1:PHOS(0.276)PPI(0.988)REG(0.634)		1.587
chr13	48891016	G	A	RB1 (Intron)	RB1:PHOS(0.276)PPI(0.988)REG(0.634)		1.149
chr1	33116171	C	A	RBBP4 (Promoter)	RBBP4:PPI(0.974)REG(0.761),ZBTB8OS:PPI(0.606)REG(0.634)		1.718
chrX	16870107	G	A	RBBP7 (Intron)	RBBP7:PPI(0.942)		1.453
chrX	16863372	C	A	RBBP7 (Intron)	RBBP7:PPI(0.942)		0.831

chrX	16867552	T	C	RBBP7 (Intron)	RBBP7:PPI(0.942)		0.831
chrX	16859481	A	G	RBBP7 (Intron)	RBBP7:PPI(0.942),TXLNG:PPI(0.142)		2.379
chrX	16859481	A	G	RBBP7 (Intron)	RBBP7:PPI(0.942),TXLNG:PPI(0.142)		2.379
chrX	16867280	C	T	RBBP7 (UTR)	RBBP7:PPI(0.942)		0.831
chr18	20573661	G	A	RBBP8 (Intron)	RBBP8:PHOS(0.622)PPI(0.774)REG(0.409)		1.105
chr18	20526531	G	C	RBBP8 (Intron)	RBBP8:PHOS(0.622)PPI(0.774)REG(0.409)		0.485
chr18	20581793	G	A	RBBP8 (Intron)	RBBP8:PHOS(0.622)PPI(0.774)REG(0.409)		0.483
chr16	53499248	T	G	RBL2 (Intron)	RBL2:PPI(0.999)		0.999
chr22	41368620	G	T	RBX1 (UTR)	RBX1:PPI(0.967)		1.901
chr22	41368620	G	T	RBX1 (UTR)	RBX1:PPI(0.967)		1.901
chr22	41368620	G	T	RBX1 (UTR)	RBX1:PPI(0.967)		1.901
chr22	41368598	G	T	RBX1 (UTR)	RBX1:PPI(0.967)		1.523
chr12	21644667	G	A	RECQL (Intron)	RECQL:PHOS(0.661)PPI(0.914)REG(0.830)		0.759
chr12	21624092	T	G	RECQL (Intron)	RECQL:PHOS(0.661)PPI(0.914)REG(0.830)		0.759
chr8	145738870	G	A	RECQL4 (Intron)	RECQL4:PHOS(0.276)		0.282
chr8	145737040	C	T	RECQL4 (Intron)	RECQL4:PHOS(0.276)		0.096
chr17	73659194	C	G	RECQL5 (Intron and Promoter)	RECQL5:PPI(0.707)		1.011
chr17	73626817	G	A	RECQL5 (Intron and Promoter)	RECQL5:PPI(0.707)		0.574
chr17	73626956	A	C	RECQL5 (Intron and Promoter)	RECQL5:PPI(0.707)		0.389
chr17	73646647	G	C	RECQL5 (UTR)	RECQL5:PPI(0.707)		0.389
chr10	43617388	C	T	RET (Intron)	RET:PHOS(0.991)PPI(0.880)		1.117
chr10	43615505	G	A	RET (Intron)	RET:PHOS(0.991)PPI(0.880)		0.973
chr2	100038145	A	C	REV1 (Intron)	REV1:PPI(0.877)REG(0.634)		1.481
chr2	100029068	C	T	REV1 (Intron)	REV1:PPI(0.877)REG(0.634)		0.673
chr6	111679869	G	T	REV3L (Intron)	REV3L:PPI(0.860)REG(0.409)		1.445
chr6	111656631	C	G	REV3L (Intron)	REV3L:PPI(0.860)REG(0.409)		1.260
chr6	111631309	G	A	REV3L (Intron)	REV3L:PPI(0.860)REG(0.409)		1.260
chr6	111643993	A	C	REV3L (Intron)	REV3L:PPI(0.860)REG(0.409)		1.260
chr6	111702463	C	G	REV3L (Intron)	REV3L:PPI(0.860)REG(0.409)		0.822
chr6	111709378	A	C	REV3L (Intron)	REV3L:PPI(0.860)REG(0.409)		0.637
chr6	111670623	A	T	REV3L (Intron)	REV3L:PPI(0.860)REG(0.409)		0.637
chr6	111636322	G	A	REV3L (Intron)	REV3L:PPI(0.860)REG(0.409)		0.637

chr6	111751174	G	A	REV3L (Intron)	REV3L:PPI(0.860)REG(0.409)		0.637
chr6	111621076	C	T	REV3L (UTR)	REV3L:PPI(0.860)REG(0.409)		0.637
chr4	39306336	T	G	RFC1 (Intron)	RFC1:PPI(0.942)REG(0.634)		1.016
chr4	39328325	C	A	RFC1 (Intron)	RFC1:PPI(0.942)REG(0.634)		1.016
chr4	39312841	A	G	RFC1 (Intron)	RFC1:PPI(0.942)REG(0.634)		0.831
chr4	39297495	A	C	RFC1 (Intron)	RFC1:PPI(0.942)REG(0.634)		0.831
chr4	39304087	C	A	RFC1 (Intron)	RFC1:PPI(0.942)REG(0.634)		0.831
chr4	39297382	G	C	RFC1 (Intron)	RFC1:PPI(0.942)REG(0.634)		0.831
chr3	186508201	T	C	RFC4 (Intron)	RFC4:PHOS(0.398)PPI(0.869)REG(0.634)		2.453
chr1	176153835	A	G	RFWD2 (Intron and Promoter)	RFWD2:PPI(0.606)REG(0.409)		0.280
chr1	175957417	C	T	RFWD2 (Intron)	RFWD2:PPI(0.606)REG(0.409)		0.903
chr1	176144938	G	C	RFWD2 (Intron)	RFWD2:PPI(0.606)REG(0.409)		0.903
chr16	74678359	A	G	RFWD3 (Intron)	RFWD3:PPI(0.540)REG(0.761)		0.463
chr16	74702456	G	T	RFWD3 (Promoter)	RFWD3:PPI(0.540)REG(0.761)		1.648
chr16	74702242	G	A	RFWD3 (Promoter)	RFWD3:PPI(0.540)REG(0.761)		1.648
chr1	151317982	G	A	RFX5 (Intron and Promoter)	RFX5:PPI(0.408)REG(0.990)		0.970
chr5	38953434	A	G	RICTOR (Intron)	RICTOR:PPI(0.408)		0.771
chr5	38962896	C	G	RICTOR (Intron)	RICTOR:PPI(0.408)		0.771
chr5	38957623	G	C	RICTOR (Intron)	RICTOR:PPI(0.408)		0.172
chr5	38947606	G	A	RICTOR (Intron)	RICTOR:PPI(0.408)		0.148
chr5	38957865	G	C	RICTOR (Intron)	RICTOR:PPI(0.408)		0.148
chr3	196230093	G	C	RNF168 (UTR)	RNF168:PPI(0.515)REG(0.830)		1.998
chr17	56431485	G	C	RNF43 (UTR)	RNF43:PPI(0.606)REG(0.761),SUPT4H1:PHOS(0.634)PPI(0.974)REG(0.830)		1.527
chr6	37336126	G	T	RNF8 (Intron and Promoter)	RNF8:PPI(0.707)REG(0.409)		0.389
chr6	117638450	G	T	ROS1 (Intron)	ROS1:PPI(0.739)		1.239
chr6	117629955	A	G	ROS1 (Intron)	ROS1:PPI(0.739)		1.054
chr6	117632392	G	C	ROS1 (Intron)	ROS1:PPI(0.739)		1.054
chr6	117631537	G	T	ROS1 (Intron)	ROS1:PPI(0.739)		1.054
chr6	117638473	G	T	ROS1 (Intron)	ROS1:PPI(0.739)		0.616
chr6	117631189	C	T	ROS1 (Intron)	ROS1:PPI(0.739)		0.431
chr6	117609588	G	C	ROS1 (UTR)	ROS1:PPI(0.739)		0.431
chr16	12059378	C	T	RP11-166B2.1 (Intron), TNFRSF17 (Intron and Medial)	TNFRSF17:PPI(0.408)REG(0.634)		1.104

chr3	14189335	G	T	RP11-434D12.1 (Intron), XPC (Intron)	XPC:PPI(0.897)REG(0.830)		0.903
chr17	1779191	T	C	RPA1 (Intron)	RPA1:PHOS(0.844)PPI(0.983)REG(0.634)		0.948
chr1	28240985	G	C	RPA2 (UTR)	RPA2:PHOS(0.921)PPI(0.992)REG(0.880)		1.774
chr1	93298416	T	G	RPL5 (Intron)	RPL5:PPI(0.980)REG(0.761)		2.736
chr1	93297636	C	A	RPL5 (Promoter and UTR)	RPL5:PPI(0.980)REG(0.761)		3.709
chr1	93297705	G	C	RPL5 (UTR)	RPL5:PPI(0.980)REG(0.761)		3.706
chr3	128348787	G	C	RPN1 (Intron)	RPN1:PHOS(0.459)PPI(0.869)		1.279
chr15	63447989	G	A	RPS27L (Intron)	RPS27L:PPI(0.448)		0.965
chr15	63449714	G	C	RPS27L (Promoter and UTR)	RPS27L:PPI(0.448)	MOTIFBR=ZNF263#ZNF263_disc1_8mer#63449707#63449717#+#8#0000000#0032000,MOTIFG=E2F_known4#63449712#63449718#+#3#6852#2458	2.922
chr11	75113345	C	T	RPS3 (Intron)	RPS3:PPI(0.995)REG(0.634)		1.783
chr17	78931638	G	A	RPTOR (Intron)	RPTOR:PPI(0.361)REG(0.409)		0.956
chr17	78858805	C	T	RPTOR (Intron)	RPTOR:PPI(0.361)REG(0.409)		0.956
chr17	78599488	C	G	RPTOR (Intron)	RPTOR:PPI(0.361)REG(0.409)		0.956
chr17	78860747	G	C	RPTOR (Intron)	RPTOR:PPI(0.361)REG(0.409)		0.333
chr17	78796766	T	A	RPTOR (Intron)	RPTOR:PPI(0.361)REG(0.409)		0.333
chr17	78831542	G	A	RPTOR (Intron)	RPTOR:PPI(0.361)REG(0.409)		0.333
chr17	78899315	T	C	RPTOR (Intron)	RPTOR:PPI(0.361)REG(0.409)		0.333
chr8	103244186	G	T	RRM2B (Intron)	RRM2B:PPI(0.448)REG(0.634)		0.492
chr20	62324691	C	T	RTEL1 (Intron and Medial)	RTEL1:REG(0.634),TNFRSF6B:PPI(0.482)REG(0.409)		0.492
chr20	62293195	C	G	RTEL1 (Intron)	RTEL1:REG(0.634)		0.930
chr20	62293996	C	A	RTEL1 (Intron)	RTEL1:REG(0.634)		0.492
chr20	62293714	C	G	RTEL1 (Intron)	RTEL1:REG(0.634)		0.307
chr21	36421254	A	C	RUNX1 (Medial and UTR)	RUNX1:PPI(0.853)REG(0.880)		2.100
chr21	36421296	A	C	RUNX1 (Medial and UTR)	RUNX1:PPI(0.853)REG(0.880)		1.477
chr20	43956241	G	A	SDC4 (Intron)	SDC4:PPI(0.746)REG(0.830)		0.578
chr1	161310376	C	A	SDHC (Intron)	SDHC:PHOS(0.276)PPI(0.408)		0.771
chrX	118770811	T	G	SEPT6 (Intron and Medial)	SEPT6:PPI(0.707)	MOTIFG=ZEB1_known1#118770806#118770812#-#1#6794#3667	2.550
chrX	118770983	G	A	SEPT6 (Intron and Medial)	SEPT6:PPI(0.707)		1.574
chrX	118774593	A	G	SEPT6 (Intron)	SEPT6:PPI(0.707)		1.389

chrX	118774593	A	G	SEPT6 (Intron)	SEPT6:PPI(0.707)		1.389
chrX	118780559	C	G	SEPT6 (Intron)	SEPT6:PPI(0.707)		0.574
chrX	118784135	A	C	SEPT6 (Intron)	SEPT6:PPI(0.707)		0.389
chrX	118767248	C	T	SEPT6 (Intron)	SEPT6:PPI(0.707)		0.389
chr9	131456171	T	A	SET (Intron)	SET:PHOS(0.670)PPI(0.928)REG(0.761)		0.794
chr18	42406242	A	C	SETBP1 (Intron)	SETBP1:PHOS(0.398)PPI(0.236)		1.328
chr18	42406242	A	C	SETBP1 (Intron)	SETBP1:PHOS(0.398)PPI(0.236)		1.328
chr18	42449086	C	A	SETBP1 (Intron)	SETBP1:PHOS(0.398)PPI(0.236)		0.766
chr18	42413786	C	A	SETBP1 (Intron)	SETBP1:PHOS(0.398)PPI(0.236)		0.766
chr18	42400171	A	G	SETBP1 (Intron)	SETBP1:PHOS(0.398)PPI(0.236)		0.328
chr18	42375513	T	C	SETBP1 (Intron)	SETBP1:PHOS(0.398)PPI(0.236)		0.143
chr3	47061024	C	G	SETD2 (Intron)	SETD2:PPI(0.965)		1.692
chr3	47158329	C	T	SETD2 (Intron)	SETD2:PPI(0.965)		1.518
chr3	47087949	G	C	SETD2 (Intron)	SETD2:PPI(0.965)		1.482
chr3	47144704	C	A	SETD2 (Intron)	SETD2:PPI(0.965)		1.080
chr3	47137302	G	T	SETD2 (Intron)	SETD2:PPI(0.965)		0.895
chr3	47080933	G	A	SETD2 (Intron)	SETD2:PPI(0.965)		0.895
chr3	47080954	C	T	SETD2 (Intron)	SETD2:PPI(0.965)		0.895
chr3	47117841	T	C	SETD2 (Intron)	SETD2:PPI(0.965)		0.895
chr3	47083569	T	C	SETD2 (Intron)	SETD2:PPI(0.965)		0.895
chr3	47104708	T	C	SETD2 (Intron)	SETD2:PPI(0.965)		0.895
chr3	47100268	A	G	SETD2 (Intron)	SETD2:PPI(0.965)		0.895
chr3	47137493	G	A	SETD2 (Intron)	SETD2:PPI(0.965)		0.895
chr1	150915576	A	C	SETDB1 (Intron)	SETDB1:PHOS(0.276)PPI(0.984)REG(0.992)		1.162
chr1	150932931	T	G	SETDB1 (Intron)	SETDB1:PHOS(0.276)PPI(0.984)REG(0.992)		1.162
chr1	150912555	A	C	SETDB1 (Intron)	SETDB1:PHOS(0.276)PPI(0.984)REG(0.992)		0.976
chr1	150916234	G	C	SETDB1 (Intron)	SETDB1:PHOS(0.276)PPI(0.984)REG(0.992)		0.976
chr1	150912576	G	A	SETDB1 (Intron)	SETDB1:PHOS(0.276)PPI(0.984)REG(0.992)		0.976
chr9	135164044	G	A	SETX (Intron)	SETX:PPI(0.729)REG(0.409)		1.040
chr9	135153668	C	T	SETX (Intron)	SETX:PPI(0.729)REG(0.409)		1.040
chr9	135173950	A	G	SETX (Intron)	SETX:PPI(0.729)REG(0.409)		0.602
chr9	135187128	G	A	SETX (Intron)	SETX:PPI(0.729)REG(0.409)		0.417
chr9	135161945	G	A	SETX (Intron)	SETX:PPI(0.729)REG(0.409)		0.417
chr9	135153673	T	C	SETX (Intron)	SETX:PPI(0.729)REG(0.409)		0.417
chr2	198262848	A	C	SF3B1 (Intron)	SF3B1:PHOS(0.681)PPI(0.981)		1.565

chr2	198288388	G	C	SF3B1 (Intron)	SF3B1:PHOS(0.681)PPI(0.981)		0.942
chrX	123499676	T	G	SH2D1A (Intron), STAG2 (Intron)	SH2D1A:PPI(0.739),STAG2:PPI(0.515)REG(0.634)		1.054
chr6	146215987	G	A	SHPRH (Intron and Promoter)	SHPRH:PPI(0.307)REG(0.409)		0.148
chr6	146242178	G	T	SHPRH (Intron)	SHPRH:PPI(0.307)REG(0.409)		0.150
chr6	146240443	C	A	SHPRH (Intron)	SHPRH:PPI(0.307)REG(0.409)		0.148
chr6	146247216	C	A	SHPRH (Intron)	SHPRH:PPI(0.307)REG(0.409)		0.148
chr15	75704263	C	A	SIN3A (Intron)	SIN3A:PPI(0.996)REG(0.994)		0.989
chr4	42062197	C	A	SLC30A9 (Intron)	SLC30A9:PHOS(0.276)		0.148
chr4	25674706	C	T	SLC34A2 (Intron)	SLC34A2:REG(0.409)		0.956
chr4	25664505	C	G	SLC34A2 (Intron)	SLC34A2:REG(0.409)		0.333
chr4	25669640	G	A	SLC34A2 (Intron)	SLC34A2:REG(0.409)		0.333
chr4	25677695	C	G	SLC34A2 (Intron)	SLC34A2:REG(0.409)		0.333
chr4	25677553	G	A	SLC34A2 (Intron)	SLC34A2:REG(0.409)		0.148
chr4	25677609	C	G	SLC34A2 (Intron)	SLC34A2:REG(0.409)		0.148
chr1	205633797	G	A	SLC45A3 (UTR)	SLC45A3:REG(0.409)		0.333
chr10	105727694	A	C	SLK (Intron)	SLK:PPI(0.448)		2.588
chr10	105727694	A	C	SLK (Intron)	SLK:PPI(0.448)		2.588
chr10	105765667	C	T	SLK (Intron)	SLK:PPI(0.448)		0.791
chr10	105761821	C	T	SLK (Intron)	SLK:PPI(0.448)		0.791
chr10	105752478	T	C	SLK (Intron)	SLK:PPI(0.448)		0.168
chr10	105785576	A	C	SLK (UTR)	SLK:PPI(0.448)		0.791
chr16	3641352	G	A	SLX4 (Intron)	SLX4:PPI(0.142)		0.248
chr16	3647707	A	G	SLX4 (Intron)	SLX4:PPI(0.142)		0.063
chr16	3652016	T	C	SLX4 (Intron)	SLX4:PPI(0.142)		0.063
chr18	45371964	C	T	SMAD2 (Intron)	SMAD2:PHOS(0.540)PPI(0.996)REG(0.409)		1.612
chr15	67430492	A	C	SMAD3 (Intron and Promoter)	SMAD3:PHOS(0.717)PPI(0.996)REG(0.830)		1.174
chrX	128633880	C	A	SMARCA1 (Intron)	SMARCA1:PHOS(0.459)PPI(0.361)REG(0.634)		0.930
chrX	128626944	G	C	SMARCA1 (Intron)	SMARCA1:PHOS(0.459)PPI(0.361)REG(0.634)		0.930
chrX	128650475	C	G	SMARCA1 (Intron)	SMARCA1:PHOS(0.459)PPI(0.361)REG(0.634)		0.930
chrX	128599370	G	C	SMARCA1 (Intron)	SMARCA1:PHOS(0.459)PPI(0.361)REG(0.634)		0.307
chrX	128615043	C	A	SMARCA1 (Intron)	SMARCA1:PHOS(0.459)PPI(0.361)REG(0.634)		0.307

chr9	2186306	G	T	SMARCA2 (Intron)	SMARCA2:PPI(0.982)REG(0.409)		1.464
chr9	2040026	C	G	SMARCA2 (Intron)	SMARCA2:PPI(0.982)REG(0.409)		1.131
chr9	2033182	A	C	SMARCA2 (Intron)	SMARCA2:PPI(0.982)REG(0.409)		0.945
chr9	2083313	C	G	SMARCA2 (Intron)	SMARCA2:PPI(0.982)REG(0.409)		0.945
chr9	2070400	G	A	SMARCA2 (Intron)	SMARCA2:PPI(0.982)REG(0.409)		0.945
chr9	2191588	T	C	SMARCA2 (Intron)	SMARCA2:PPI(0.982)REG(0.409)		0.945
chr9	2088674	A	C	SMARCA2 (Intron)	SMARCA2:PPI(0.982)REG(0.409)		0.945
chr9	2193411	C	G	SMARCA2 (UTR)	SMARCA2:PPI(0.982)REG(0.409)		1.568
chr19	11123791	G	C	SMARCA4 (Intron)	SMARCA4:PHOS(0.509)PPI(0.994)REG(0.994)		1.791
chr19	11113651	T	C	SMARCA4 (Intron)	SMARCA4:PHOS(0.509)PPI(0.994)REG(0.994)		1.168
chr19	11129600	C	T	SMARCA4 (Intron)	SMARCA4:PHOS(0.509)PPI(0.994)REG(0.994)		1.168
chr19	11137180	T	C	SMARCA4 (Intron)	SMARCA4:PHOS(0.509)PPI(0.994)REG(0.994)		0.983
chr22	24135986	G	C	SMARCB1 (Intron)	SMARCB1:PHOS(0.276)PPI(0.990)REG(0.993)		0.980
chr22	24129335	G	C	SMARCB1 (UTR)	SMARCB1:PHOS(0.276)PPI(0.990)REG(0.993)		2.400
chr10	112343591	G	A	SMC3 (Intron)	SMC3:PHOS(0.722)PPI(0.937)REG(0.990)		1.593
chr9	72961240	T	G	SMC5 (Intron)	SMC5:PPI(0.563)		0.867
chr9	72929769	C	T	SMC5 (Intron)	SMC5:PPI(0.563)		0.867
chr9	72929759	C	A	SMC5 (Intron)	SMC5:PPI(0.563)		0.244
chr2	17883088	T	G	SMC6 (Intron)	SMC6:PPI(0.739)		1.054
chr2	17888693	T	G	SMC6 (Intron)	SMC6:PPI(0.739)		1.054
chr2	17923166	G	C	SMC6 (Intron)	SMC6:PPI(0.739)		0.433
chr2	17888374	G	C	SMC6 (Intron)	SMC6:PPI(0.739)		0.431
chr2	17902115	G	A	SMC6 (Intron)	SMC6:PPI(0.739)		0.431
chr2	17902640	C	T	SMC6 (Intron)	SMC6:PPI(0.739)		0.431
chr16	18839496	G	C	SMG1 (Intron)	SMG1:PPI(0.307)REG(0.761)		1.883
chr16	18880347	C	T	SMG1 (Intron)	SMG1:PPI(0.307)REG(0.761)		1.271
chr16	18875240	G	A	SMG1 (Intron)	SMG1:PPI(0.307)REG(0.761)		1.085
chr16	18851325	G	T	SMG1 (Intron)	SMG1:PPI(0.307)REG(0.761)		1.085
chr16	18823080	C	T	SMG1 (Intron)	SMG1:PPI(0.307)REG(0.761)		1.085
chr16	18882675	C	A	SMG1 (Intron)	SMG1:PPI(0.307)REG(0.761)		1.085
chr16	18849858	C	T	SMG1 (Intron)	SMG1:PPI(0.307)REG(0.761)		1.085
chr16	18859363	G	A	SMG1 (Intron)	SMG1:PPI(0.307)REG(0.761)		1.085
chr16	18879677	C	A	SMG1 (Intron)	SMG1:PPI(0.307)REG(0.761)		1.085

chr16	18848768	G	A	SMG1 (Intron)	SMG1:PPI(0.307)REG(0.761)		1.085
chr16	18844184	C	T	SMG1 (Intron)	SMG1:PPI(0.307)REG(0.761)		0.463
chr16	18875262	G	T	SMG1 (Intron)	SMG1:PPI(0.307)REG(0.761)		0.463
chr16	18848055	A	C	SMG1 (Intron)	SMG1:PPI(0.307)REG(0.761)		0.463
chr16	18903415	G	C	SMG1 (Intron)	SMG1:PPI(0.307)REG(0.761)		0.463
chr16	18839605	C	A	SMG1 (Intron)	SMG1:PPI(0.307)REG(0.761)		0.463
chr16	18863244	A	G	SMG1 (Intron)	SMG1:PPI(0.307)REG(0.761)		0.463
chr16	18823192	A	G	SMG1 (Intron)	SMG1:PPI(0.307)REG(0.761)		0.463
chr16	18860472	T	C	SMG1 (Intron)	SMG1:PPI(0.307)REG(0.761)		0.463
chr16	18881889	G	A	SMG1 (Intron)	SMG1:PPI(0.307)REG(0.761)		0.463
chr16	18907650	T	C	SMG1 (Intron)	SMG1:PPI(0.307)REG(0.761)		0.463
chr16	18839496	G	C	SMG1 (Intron)	SMG1:PPI(0.307)REG(0.761)		1.883
chr16	18880347	C	T	SMG1 (Intron)	SMG1:PPI(0.307)REG(0.761)		1.271
chr16	18875240	G	A	SMG1 (Intron)	SMG1:PPI(0.307)REG(0.761)		1.085
chr16	18851325	G	T	SMG1 (Intron)	SMG1:PPI(0.307)REG(0.761)		1.085
chr16	18823080	C	T	SMG1 (Intron)	SMG1:PPI(0.307)REG(0.761)		1.085
chr16	18882675	C	A	SMG1 (Intron)	SMG1:PPI(0.307)REG(0.761)		1.085
chr16	18849858	C	T	SMG1 (Intron)	SMG1:PPI(0.307)REG(0.761)		1.085
chr16	18859363	G	A	SMG1 (Intron)	SMG1:PPI(0.307)REG(0.761)		1.085
chr16	18879677	C	A	SMG1 (Intron)	SMG1:PPI(0.307)REG(0.761)		1.085
chr16	18848768	G	A	SMG1 (Intron)	SMG1:PPI(0.307)REG(0.761)		1.085
chr16	18844184	C	T	SMG1 (Intron)	SMG1:PPI(0.307)REG(0.761)		0.463
chr16	18875262	G	T	SMG1 (Intron)	SMG1:PPI(0.307)REG(0.761)		0.463
chr16	18848055	A	C	SMG1 (Intron)	SMG1:PPI(0.307)REG(0.761)		0.463
chr16	18903415	G	C	SMG1 (Intron)	SMG1:PPI(0.307)REG(0.761)		0.463
chr16	18839605	C	A	SMG1 (Intron)	SMG1:PPI(0.307)REG(0.761)		0.463
chr16	18863244	A	G	SMG1 (Intron)	SMG1:PPI(0.307)REG(0.761)		0.463
chr16	18823192	A	G	SMG1 (Intron)	SMG1:PPI(0.307)REG(0.761)		0.463
chr16	18860472	T	C	SMG1 (Intron)	SMG1:PPI(0.307)REG(0.761)		0.463
chr16	18881889	G	A	SMG1 (Intron)	SMG1:PPI(0.307)REG(0.761)		0.463
chr16	18907650	T	C	SMG1 (Intron)	SMG1:PPI(0.307)REG(0.761)		0.463
chr16	18887331	T	G	SMG1 (UTR)	SMG1:PPI(0.307)REG(0.761)		1.463
chr16	18887331	T	G	SMG1 (UTR)	SMG1:PPI(0.307)REG(0.761)		1.463
chr16	18887331	T	G	SMG1 (UTR)	SMG1:PPI(0.307)REG(0.761)		1.463
chr16	18887331	T	G	SMG1 (UTR)	SMG1:PPI(0.307)REG(0.761)		1.463
chr17	70120527	G	C	SOX9 (UTR)	SOX9:PHOS(0.585)PPI(0.685)REG(0.409)		1.782
chr17	46000069	C	T	SP2 (Intron)	SP2:PHOS(0.276)PPI(0.236)REG(0.994)		1.605
chr17	46000787	C	T	SP2 (Intron)	SP2:PHOS(0.276)PPI(0.236)REG(0.994)		0.983

chr17	46005398	G	A	SP2 (UTR)	SP2:PHOS(0.276)PPI(0.236)REG(0.994)		1.168
chr17	47699117	G	A	SPOP (Intron)	SPOP:PPI(0.863)REG(0.761)		1.266
chr17	47700060	G	T	SPOP (Intron)	SPOP:PPI(0.863)REG(0.761)		1.266
chr17	47679176	C	T	SPOP (Intron)	SPOP:PPI(0.863)REG(0.761)		0.643
chr17	47677543	T	G	SPOP (UTR)	SPOP:PPI(0.863)REG(0.761)		2.451
chr17	47677547	C	A	SPOP (UTR)	SPOP:PPI(0.863)REG(0.761)		1.829
chr17	47700236	A	C	SPOP (UTR)	SPOP:PPI(0.863)REG(0.761)		1.266
chr17	47677000	C	A	SPOP (UTR)	SPOP:PPI(0.863)REG(0.761)		1.266
chr17	47676501	C	T	SPOP (UTR)	SPOP:PPI(0.863)REG(0.761)		0.829
chr2	45800487	C	G	SRBD1 (Intron)	SRBD1:PHOS(0.276)PPI(0.408)REG(0.409)		0.956
chr2	45812666	A	G	SRBD1 (Intron)	SRBD1:PHOS(0.276)PPI(0.408)REG(0.409)		0.333
chr2	45778210	G	A	SRBD1 (Intron)	SRBD1:PHOS(0.276)PPI(0.408)REG(0.409)		0.148
chr6	43146627	A	T	SRF (Intron)	SRF:PHOS(0.775)PPI(0.923)REG(0.995)		2.794
chr6	43146640	A	C	SRF (Intron)	SRF:PHOS(0.775)PPI(0.923)REG(0.995)		2.171
chr6	43146522	C	A	SRF (Intron)	SRF:PHOS(0.775)PPI(0.923)REG(0.995)		1.609
chr6	43144410	A	G	SRF (Intron)	SRF:PHOS(0.775)PPI(0.923)REG(0.995)		1.609
chr6	43143723	G	T	SRF (Intron)	SRF:PHOS(0.775)PPI(0.923)REG(0.995)		1.171
chr6	43146993	C	T	SRF (UTR)	SRF:PHOS(0.775)PPI(0.923)REG(0.995)		1.609
chrX	123339316	G	A	STAG2 (Intron)	STAG2:PPI(0.515)REG(0.634)		1.492
chrX	123338806	C	T	STAG2 (Intron)	STAG2:PPI(0.515)REG(0.634)		1.492
chrX	123339488	G	T	STAG2 (Intron)	STAG2:PPI(0.515)REG(0.634)		1.492
chrX	123181099	G	A	STAG2 (Intron)	STAG2:PPI(0.515)REG(0.634)		0.926
chrX	123415243	C	T	STAG2 (Intron)	STAG2:PPI(0.515)REG(0.634)		0.492
chrX	123196909	G	T	STAG2 (Intron)	STAG2:PPI(0.515)REG(0.634)		0.307
chrX	123156313	G	C	STAG2 (Intron)	STAG2:PPI(0.515)REG(0.634)		0.307
chrX	123211707	T	G	STAG2 (Intron)	STAG2:PPI(0.515)REG(0.634)		0.307
chrX	123156630	A	C	STAG2 (Intron)	STAG2:PPI(0.515)REG(0.634)		0.307
chrX	123165162	G	T	STAG2 (Intron)	STAG2:PPI(0.515)REG(0.634)		0.307
chrX	123171256	A	C	STAG2 (Intron)	STAG2:PPI(0.515)REG(0.634)		0.307
chrX	123185392	G	A	STAG2 (Intron)	STAG2:PPI(0.515)REG(0.634)		0.307
chrX	123540352	A	C	STAG2 (Intron)	STAG2:PPI(0.515)REG(0.634)		1.115
chrX	123156454	G	T	STAG2 (UTR)	STAG2:PPI(0.515)REG(0.634)		0.930
chrX	123234621	C	T	STAG2 (UTR)	STAG2:PPI(0.515)REG(0.634)		0.930
chr12	56750077	C	G	STAT2 (Intron)	STAT2:PPI(0.846)REG(0.989)		1.775
chr12	56745356	G	C	STAT2 (Intron)	STAT2:PPI(0.846)REG(0.989)		1.590
chr12	56748403	G	A	STAT2 (Intron)	STAT2:PPI(0.846)REG(0.989)		1.152
chr12	56750385	C	T	STAT2 (Intron)	STAT2:PPI(0.846)REG(0.989)		0.967

chr17	40481245	C	A	STAT3 (Intron and Medial)	STAT3:PHOS(0.459)PPI(0.978)REG(0.996)		2.409
chr17	40477238	G	A	STAT3 (Intron)	STAT3:PHOS(0.459)PPI(0.978)REG(0.996)		1.786
chr17	40475711	A	G	STAT3 (Intron)	STAT3:PHOS(0.459)PPI(0.978)REG(0.996)		1.174
chr17	40490579	G	T	STAT3 (Intron)	STAT3:PHOS(0.459)PPI(0.978)REG(0.996)		1.174
chr17	40489964	T	C	STAT3 (Intron)	STAT3:PHOS(0.459)PPI(0.978)REG(0.996)		1.174
chr17	40460948	C	A	STAT5A (Intron)	STAT5A:PPI(0.945)		1.647
chr17	40460191	C	T	STAT5A (Intron)	STAT5A:PPI(0.945)		0.839
chr17	40462837	G	A	STAT5A (UTR)	STAT5A:PPI(0.945)		1.636
chr17	40376887	C	T	STAT5B (Intron)	STAT5B:PHOS(0.509)PPI(0.917)		1.574
chr19	1222012	G	C	STK11 (Intron)	STK11:PHOS(0.896)PPI(0.781)REG(0.634)		0.901
chr13	48563257	T	G	SUCLA2 (Intron)	SUCLA2:PPI(0.606)		0.280
chr13	53262072	G	T	SUGT1 (UTR)	SUGT1:PPI(0.781)		1.116
chr14	21836692	T	C	SUPT16H (Intron)	SUPT16H:PPI(0.987)REG(0.880)		1.584
chr14	21822087	G	T	SUPT16H (Intron)	SUPT16H:PPI(0.987)REG(0.880)		0.961
chr14	21826399	T	G	SUPT16H (Intron)	SUPT16H:PPI(0.987)REG(0.880)		0.961
chr14	21831133	G	T	SUPT16H (Intron)	SUPT16H:PPI(0.987)REG(0.880)		0.961
chr14	21840280	C	A	SUPT16H (Intron)	SUPT16H:PPI(0.987)REG(0.880)		0.961
chr17	27000300	T	G	SUPT6H (Intron)	SUPT6H:PHOS(0.736)PPI(0.787)		1.503
chr17	27000300	T	G	SUPT6H (Intron)	SUPT6H:PHOS(0.736)PPI(0.787)		1.503
chr17	27012045	A	G	SUPT6H (Intron)	SUPT6H:PHOS(0.736)PPI(0.787)		1.300
chr17	27013255	G	A	SUPT6H (Intron)	SUPT6H:PHOS(0.736)PPI(0.787)		1.126
chr17	27024780	A	G	SUPT6H (Intron)	SUPT6H:PHOS(0.736)PPI(0.787)		0.503
chr17	27011599	C	G	SUPT6H (Intron)	SUPT6H:PHOS(0.736)PPI(0.787)		0.503
chr17	30302735	A	C	SUZ12 (Intron)	SUZ12:PPI(0.540)REG(0.997)		1.800
chr17	30303726	G	A	SUZ12 (Intron)	SUZ12:PPI(0.540)REG(0.997)		1.615
chr17	30302393	G	A	SUZ12 (Intron)	SUZ12:PPI(0.540)REG(0.997)		1.615
chr17	30300157	C	G	SUZ12 (Intron)	SUZ12:PPI(0.540)REG(0.997)		1.615
chr9	93650733	T	G	SYK (Intron)	SYK:PHOS(0.983)PPI(0.945)REG(0.634)		1.948
chr9	93629296	A	G	SYK (Intron)	SYK:PHOS(0.983)PPI(0.945)REG(0.634)		1.948
chr9	93629296	A	G	SYK (Intron)	SYK:PHOS(0.983)PPI(0.945)REG(0.634)		1.948
chr9	93650733	T	G	SYK (Intron)	SYK:PHOS(0.983)PPI(0.945)REG(0.634)		1.948
chr9	93650916	A	G	SYK (Intron)	SYK:PHOS(0.983)PPI(0.945)REG(0.634)		1.756
chr9	93624481	C	T	SYK (Intron)	SYK:PHOS(0.983)PPI(0.945)REG(0.634)		1.571
chr9	93639746	T	G	SYK (Intron)	SYK:PHOS(0.983)PPI(0.945)REG(0.634)		0.948

chr9	93650742	G	T	SYK (Intron)	SYK:PHOS(0.983)PPI(0.945)REG(0.634)		0.948
chr9	93627090	G	A	SYK (Intron)	SYK:PHOS(0.983)PPI(0.945)REG(0.634)		0.948
chrX	70602269	C	G	TAF1 (Intron and Medial)	TAF1:PHOS(0.878)PPI(0.979)REG(1.000)		1.971
chrX	70749590	T	C	TAF1 (Intron)	TAF1:PHOS(0.878)PPI(0.979)REG(1.000)		1.810
chrX	70641269	T	G	TAF1 (Intron)	TAF1:PHOS(0.878)PPI(0.979)REG(1.000)		1.187
chrX	70627530	C	A	TAF1 (Intron)	TAF1:PHOS(0.878)PPI(0.979)REG(1.000)		1.187
chrX	70640600	C	A	TAF1 (Intron)	TAF1:PHOS(0.878)PPI(0.979)REG(1.000)		1.187
chrX	70678341	T	A	TAF1 (Intron)	TAF1:PHOS(0.878)PPI(0.979)REG(1.000)		1.002
chrX	70586113	T	G	TAF1 (Promoter and UTR)	TAF1:PHOS(0.878)PPI(0.979)REG(1.000)		2.799
chrX	70586113	T	G	TAF1 (Promoter and UTR)	TAF1:PHOS(0.878)PPI(0.979)REG(1.000)		2.799
chrX	70586108	T	G	TAF1 (Promoter)	TAF1:PHOS(0.878)PPI(0.979)REG(1.000)		2.799
chr17	27809114	G	A	TAOK1 (Intron)	TAOK1:PPI(0.938)		2.628
chr17	27809114	G	A	TAOK1 (Intron)	TAOK1:PPI(0.938)		2.628
chr17	27822515	G	C	TAOK1 (Intron)	TAOK1:PPI(0.938)		0.828
chr17	27744623	C	T	TAOK1 (Intron)	TAOK1:PPI(0.938)		0.820
chr16	30000824	T	C	TAOK2 (Intron)	TAOK2:PPI(0.657)REG(0.761)		0.463
chr16	29982865	G	A	TAOK2 (Promoter)	TAOK2:PPI(0.657)REG(0.761)		1.085
chr16	29982906	T	G	TAOK2 (Promoter)	TAOK2:PPI(0.657)REG(0.761)		0.463
chr12	118682668	C	G	TAOK3 (Intron and Promoter)	TAOK3:PPI(0.307)REG(0.634)	MOTIFG=TCF12_disc4#118682664#118682672#-#4#9802#5408	1.290
chr12	118684348	G	C	TAOK3 (Intron)	TAOK3:PPI(0.307)REG(0.634)		0.492
chr3	176743323	T	C	TBL1XR1 (Intron)	TBL1XR1:PPI(0.964)REG(0.634)		0.892
chr6	170880592	G	A	TBP (Intron)	TBP:PPI(0.998)REG(0.996)		1.618
chrX	79282045	C	T	TBX22 (Intron)	TBX22:PHOS(0.276)		0.096
chr8	54882758	C	G	TCEA1 (Intron)	TCEA1:REG(0.634)		0.307
chr8	54891381	C	T	TCEA1 (Intron)	TCEA1:REG(0.634)		0.307
chr8	54906334	C	G	TCEA1 (Intron)	TCEA1:REG(0.634)		0.307
chr15	57526179	G	C	TCF12 (Intron)	TCF12:PHOS(0.398)PPI(0.853)REG(0.996)		0.989

chr15	57212095	G	A	TCF12 (Medial and UTR)	TCF12:PHOS(0.398)PPI(0.853)REG(0.996),ZNF280D:REG(0.761)		1.797
chr19	1624007	A	G	TCF3 (Intron)	TCF3:PHOS(0.509)PPI(0.917)		0.951
chr18	53123172	A	G	TCF4 (Intron)	TCF4:PHOS(0.276)PPI(0.804)REG(0.997)		1.800
chr10	114912203	G	C	TCF7L2 (Intron and Medial)	TCF7L2:PPI(0.814)REG(0.409)		1.357
chr10	114905728	A	C	TCF7L2 (Intron and Promoter)	TCF7L2:PPI(0.814)REG(0.409)		0.549
chr10	114724501	G	A	TCF7L2 (Intron)	TCF7L2:PPI(0.814)REG(0.409)		0.799
chr10	114901140	G	T	TCF7L2 (Intron)	TCF7L2:PPI(0.814)REG(0.409)		0.742
chr12	110344308	G	T	TCHP (Intron)	TCHP:PPI(0.448)		0.168
chr12	110344483	C	T	TCHP (Intron)	TCHP:PPI(0.448)		0.168
chr12	104379616	G	A	TDG (Intron)	TDG:PPI(0.792)REG(0.761)		0.511
chr12	104380905	T	G	TDG (UTR)	TDG:PPI(0.792)REG(0.761)		1.245
chr16	1553023	C	T	TELO2 (Intron)	TELO2:PPI(0.890)		0.702
chr16	1556811	C	G	TELO2 (Intron)	TELO2:PPI(0.890)		0.702
chr5	1268749	C	A	TERT (Intron)	TERT:PPI(0.798)		1.144
chr5	1268808	C	G	TERT (Intron)	TERT:PPI(0.798)		0.521
chr4	106164216	C	A	TET2 (Intron)	TET2:PPI(0.142)REG(0.409)		0.148
chr4	106158749	C	T	TET2 (UTR)	TET2:PPI(0.142)REG(0.409)		0.148
chr6	10407113	C	T	TFAP2A (Intron and Promoter)	TFAP2A:PHOS(0.398)PPI(0.798)REG(0.991)		1.781
chr20	55204370	C	T	TFAP2C (Promoter and UTR)	TFAP2C:PHOS(0.651)PPI(0.448)REG(0.993)		1.165
chr3	141671859	C	G	TFDP2 (Intron)	TFDP2:PPI(0.774)		1.291
chr3	195798261	A	G	TFRC (Intron)	TFRC:PPI(0.685)REG(0.929)		2.217
chr3	30713977	A	C	TGFBR2 (Intron)	TGFBR2:PHOS(0.722)PPI(0.883)REG(0.929)		0.982
chr3	30686414	A	G	TGFBR2 (Intron)	TGFBR2:PHOS(0.722)PPI(0.883)REG(0.929)		0.982
chr19	19241254	C	T	TMEM161A (Intron)	TMEM161A:PPI(0.236)REG(0.880)		0.865
chr20	48731885	T	G	TMEM189 (Intron), TMEM189-UBE2V1 (Intron), UBE2V1 (Intron and Promoter)	TMEM189-UBE2V1:REG(0.409),TMEM189:REG(0.409),UBE2V1:PHOS(0.634)PPI(0.822)REG(0.880)	MOTIFG=ZEB1_known1#48731880#48731886#-#1#6794#3667	3.453
chr6	138192351	T	G	TNFAIP3 (Medial and UTR)	TNFAIP3:PPI(0.729)REG(0.880)	MOTIFG=ZEB1_known1#138192346#138192352#-#1#6794#3667	2.453
chr1	2491205	C	T	TNFRSF14 (Intron)	TNFRSF14:PPI(0.935)REG(0.409)		0.812

chr16	12059075	C	G	TNFRSF17 (Promoter and UTR)	TNFRSF17:PPI(0.408)REG(0.634)		1.104
chr8	9537348	T	G	TNKS (Intron)	TNKS:PPI(0.707)		2.011
chr8	9537348	T	G	TNKS (Intron)	TNKS:PPI(0.707)		2.011
chr8	9605522	C	T	TNKS (Intron)	TNKS:PPI(0.707)		0.574
chr8	9567646	C	A	TNKS (Intron)	TNKS:PPI(0.707)		0.389
chr8	9618878	T	A	TNKS (Intron)	TNKS:PPI(0.707)		0.389
chr10	93596466	T	C	TNKS2 (Intron)	TNKS2:PPI(0.685)		0.362
chr20	39741414	C	G	TOP1 (Intron)	TOP1:PPI(0.967)REG(0.761)		2.321
chr20	39706158	C	G	TOP1 (Intron)	TOP1:PPI(0.967)REG(0.761)		0.901
chr17	38547615	G	C	TOP2A (Intron and Promoter)	TOP2A:PPI(0.482)REG(0.909)		1.555
chr17	18186158	G	C	TOP3A (Intron)	TOP3A:PPI(0.860)REG(0.634)		0.822
chr17	18206071	C	G	TOP3A (Intron)	TOP3A:PPI(0.860)REG(0.634)		0.637
chr17	18185943	T	G	TOP3A (Intron)	TOP3A:PPI(0.860)REG(0.634)		0.637
chr17	18185971	T	G	TOP3A (Intron)	TOP3A:PPI(0.860)REG(0.634)		0.637
chr17	18193837	C	T	TOP3A (Intron)	TOP3A:PPI(0.860)REG(0.634)		0.637
chr17	18195811	C	G	TOP3A (Intron)	TOP3A:PPI(0.860)REG(0.634)		0.637
chr3	133347335	G	T	TOPBP1 (Intron)	TOPBP1:PPI(0.729)		1.225
chr3	133343047	T	C	TOPBP1 (Intron)	TOPBP1:PPI(0.729)		1.040
chr3	133342833	C	G	TOPBP1 (Intron)	TOPBP1:PPI(0.729)		1.040
chr3	133338878	T	C	TOPBP1 (Intron)	TOPBP1:PPI(0.729)		0.602
chr3	133347604	T	C	TOPBP1 (Intron)	TOPBP1:PPI(0.729)		0.417
chr3	133336999	T	C	TOPBP1 (Intron)	TOPBP1:PPI(0.729)		0.417
chr3	133331104	G	A	TOPBP1 (Intron)	TOPBP1:PPI(0.729)		0.417
chr16	52484462	G	A	TOX3 (Intron)	TOX3:PPI(0.959)		0.878
chr17	7578564	G	A	TP53 (Intron and Promoter)	TP53:PHOS(0.601)PPI(0.999)REG(0.909)	MOTIFG=PU1_known2#7578562#7578570#+#3#9093#8031	3.150
chr17	7578555	C	T	TP53 (Intron and Promoter)	TP53:PHOS(0.601)PPI(0.999)REG(0.909)		2.807
chr17	7578556	T	C	TP53 (Intron and Promoter)	TP53:PHOS(0.601)PPI(0.999)REG(0.909)		2.807
chr17	7578556	T	G	TP53 (Intron and Promoter)	TP53:PHOS(0.601)PPI(0.999)REG(0.909)		2.807
chr17	7578290	C	T	TP53 (Intron and Promoter)	TP53:PHOS(0.601)PPI(0.999)REG(0.909)		2.622
chr17	7578290	C	G	TP53 (Intron and Promoter)	TP53:PHOS(0.601)PPI(0.999)REG(0.909)		2.622

chr17	7577610	T	C	TP53 (Intron and Promoter)	TP53:PHOS(0.601)PPI(0.999)REG(0.909)		2.622
chr17	7577610	T	A	TP53 (Intron and Promoter)	TP53:PHOS(0.601)PPI(0.999)REG(0.909)		2.622
chr17	7578175	A	T	TP53 (Intron and Promoter)	TP53:PHOS(0.601)PPI(0.999)REG(0.909)		1.807
chr17	7577609	C	T	TP53 (Intron and Promoter)	TP53:PHOS(0.601)PPI(0.999)REG(0.909)		1.622
chr17	7577156	C	A	TP53 (Intron and Promoter)	TP53:PHOS(0.601)PPI(0.999)REG(0.909)		1.622
chr17	7577213	C	G	TP53 (Intron and Promoter)	TP53:PHOS(0.601)PPI(0.999)REG(0.909)		0.999
chr17	7577161	C	G	TP53 (Intron and Promoter)	TP53:PHOS(0.601)PPI(0.999)REG(0.909)		0.999
chr17	7576851	A	C	TP53 (Intron)	TP53:PHOS(0.601)PPI(0.999)REG(0.909)		2.419
chr17	7579591	C	G	TP53 (Intron)	TP53:PHOS(0.601)PPI(0.999)REG(0.909)		1.622
chr17	7579622	C	A	TP53 (Intron)	TP53:PHOS(0.601)PPI(0.999)REG(0.909)		0.999
chr15	43707677	G	A	TP53BP1 (Intron and Promoter)	TP53BP1:PPI(0.895)REG(0.409)		0.714
chr15	43739559	C	T	TP53BP1 (Intron)	TP53BP1:PPI(0.895)REG(0.409)		1.336
chr15	43773248	G	A	TP53BP1 (Intron)	TP53BP1:PPI(0.895)REG(0.409)		1.336
chr15	43770189	C	T	TP53BP1 (Intron)	TP53BP1:PPI(0.895)REG(0.409)		1.335
chr15	43783846	A	C	TP53BP1 (Intron)	TP53BP1:PPI(0.895)REG(0.409)		0.899
chr15	43769752	C	A	TP53BP1 (Intron)	TP53BP1:PPI(0.895)REG(0.409)		0.899
chr15	43733525	G	T	TP53BP1 (Intron)	TP53BP1:PPI(0.895)REG(0.409)		0.899
chr15	43708081	A	C	TP53BP1 (Intron)	TP53BP1:PPI(0.895)REG(0.409)		0.714
chr15	43773343	A	C	TP53BP1 (Intron)	TP53BP1:PPI(0.895)REG(0.409)		0.714
chr15	43724968	A	G	TP53BP1 (Intron)	TP53BP1:PPI(0.895)REG(0.409)		0.714
chr15	43734055	C	A	TP53BP1 (Intron)	TP53BP1:PPI(0.895)REG(0.409)		0.714
chr15	43785307	G	A	TP53BP1 (Promoter and UTR)	TP53BP1:PPI(0.895)REG(0.409)		0.899
chr1	3598910	C	T	TP73 (Promoter and UTR)	TP73:PPI(0.925)REG(0.409)		0.786
chr1	154148581	C	T	TPM3 (Intron)	TPM3:PPI(0.914)REG(0.761)		0.759
chr1	154163890	C	T	TPM3 (Intron)	TPM3:PPI(0.914)REG(0.761)		0.759
chr19	16178548	G	A	TPM4 (Medial and UTR)	TPM4:PPI(0.307)REG(0.830)		1.386
chr19	16178630	G	A	TPM4 (Medial and UTR)	TPM4:PPI(0.307)REG(0.830)		0.764
chr1	186331735	T	G	TPR (Intron)	TPR:PPI(0.908)REG(0.761)		1.367

chr1	186308714	T	A	TPR (Intron)	TPR:PPI(0.908)REG(0.761)		1.367
chr1	186283758	C	A	TPR (Intron)	TPR:PPI(0.908)REG(0.761)		1.367
chr1	186314830	T	C	TPR (Intron)	TPR:PPI(0.908)REG(0.761)		1.367
chr1	186319198	G	T	TPR (Intron)	TPR:PPI(0.908)REG(0.761)		1.367
chr1	186316287	C	G	TPR (Intron)	TPR:PPI(0.908)REG(0.761)		1.367
chr1	186321092	C	T	TPR (Intron)	TPR:PPI(0.908)REG(0.761)		1.367
chr1	186325258	T	C	TPR (Intron)	TPR:PPI(0.908)REG(0.761)		0.929
chr1	186324711	G	A	TPR (Intron)	TPR:PPI(0.908)REG(0.761)		0.744
chr1	186309009	A	C	TPR (Intron)	TPR:PPI(0.908)REG(0.761)		0.744
chr1	186327859	C	T	TPR (Intron)	TPR:PPI(0.908)REG(0.761)		0.744
chr1	186322673	A	G	TPR (Intron)	TPR:PPI(0.908)REG(0.761)		0.744
chr1	186292667	G	T	TPR (Intron)	TPR:PPI(0.908)REG(0.761)		0.744
chr1	186287471	C	A	TPR (Intron)	TPR:PPI(0.908)REG(0.761)		0.744
chr1	186329343	G	A	TPR (Intron)	TPR:PPI(0.908)REG(0.761)		0.744
chr1	186310039	G	T	TPR (Intron)	TPR:PPI(0.908)REG(0.761)		0.744
chr1	186312694	A	C	TPR (Intron)	TPR:PPI(0.908)REG(0.761)		0.744
chr1	186320319	A	G	TPR (Intron)	TPR:PPI(0.908)REG(0.761)		0.744
chr1	186300729	C	G	TPR (Intron)	TPR:PPI(0.908)REG(0.761)		0.744
chr16	2225153	T	C	TRAF7 (Intron)	TRAF7:PPI(0.583)REG(0.761)		1.085
chr16	2225093	A	T	TRAF7 (Intron)	TRAF7:PPI(0.583)REG(0.761)		0.463
chr16	2225153	T	C	TRAF7 (Intron)	TRAF7:PPI(0.583)REG(0.761)		1.085
chr16	2225093	A	T	TRAF7 (Intron)	TRAF7:PPI(0.583)REG(0.761)		0.463
chr14	92484111	G	T	TRIP11 (Intron and Promoter)	TRIP11:PPI(0.482)		0.811
chr14	92483914	C	T	TRIP11 (Intron and Promoter)	TRIP11:PPI(0.482)		0.188
chr14	92460257	C	T	TRIP11 (Intron)	TRIP11:PPI(0.482)		0.811
chr14	92480950	C	G	TRIP11 (Intron)	TRIP11:PPI(0.482)		0.811
chr14	92474248	A	G	TRIP11 (Intron)	TRIP11:PPI(0.482)		0.811
chr14	92491861	C	A	TRIP11 (Intron)	TRIP11:PPI(0.482)		0.811
chr14	92438977	A	C	TRIP11 (Intron)	TRIP11:PPI(0.482)		0.188
chr14	92454567	G	T	TRIP11 (Intron)	TRIP11:PPI(0.482)		0.188
chr14	92477545	C	A	TRIP11 (Intron)	TRIP11:PPI(0.482)		0.188
chr14	92435877	T	A	TRIP11 (UTR)	TRIP11:PPI(0.482)		0.188
chr9	135804110	C	G	TSC1 (Intron)	TSC1:PPI(0.921)REG(0.634)		1.394
chr9	135801345	A	G	TSC1 (Intron)	TSC1:PPI(0.921)REG(0.634)		0.776
chr9	135804110	C	G	TSC1 (Intron)	TSC1:PPI(0.921)REG(0.634)		1.394
chr9	135801345	A	G	TSC1 (Intron)	TSC1:PPI(0.921)REG(0.634)		0.776
chr16	2104451	A	C	TSC2 (Intron and Promoter)	TSC2:PPI(0.838)REG(0.909)		0.932

chr16	2127528	C	G	TSC2 (Intron)	TSC2:PPI(0.838)REG(0.909)		1.369
chr16	2100397	C	G	TSC2 (Intron)	TSC2:PPI(0.838)REG(0.909)		0.747
chr14	81563053	T	C	TSHR (Intron)	TSHR:PPI(0.739)		1.054
chr14	81563053	T	C	TSHR (Intron)	TSHR:PPI(0.739)		1.054
chr1	161011904	A	C	TSTD1 (Medial)	TSTD1:REG(0.409),USF1:PHOS(0.398)PPI(0.739) REG(0.998)		1.804
chr14	20768623	G	A	TTC5 (Intron)	TTC5:PPI(0.142)REG(0.409)		0.333
chr17	40766772	G	T	TUBG1 (Intron)	TUBG1:PPI(0.898)		0.906
chr15	43672278	A	G	TUBGCP4 (Intron)	TUBGCP4:PPI(0.767)		1.280
chr15	43670226	C	A	TUBGCP4 (Intron)	TUBGCP4:PPI(0.767)		1.095
chr15	43696494	T	G	TUBGCP4 (Intron)	TUBGCP4:PPI(0.767)		0.472
chr22	50657489	G	C	TUBGCP6 (Intron)	TUBGCP6:PPI(0.408)		0.148
chr18	662103	G	A	TYMS (Intron)	TYMS:PPI(0.448)REG(0.409)		0.353
chr21	44520685	A	C	U2AF1 (Intron)	U2AF1:PHOS(0.622)PPI(0.969)REG(0.761)		1.529
chr4	103722779	T	A	UBE2D3 (Intron)	UBE2D3:PPI(0.995)REG(0.952)		2.179
chr4	103722779	T	A	UBE2D3 (Intron)	UBE2D3:PPI(0.995)REG(0.952)		2.179
chr4	103722788	C	A	UBE2D3 (Intron)	UBE2D3:PPI(0.995)REG(0.952)		1.609
chr4	103748797	A	G	UBE2D3 (Promoter and UTR)	UBE2D3:PPI(0.995)REG(0.952)		2.033
chr22	21968864	T	G	UBE2L3 (Intron)	UBE2L3:PHOS(0.769)PPI(0.831)REG(0.977)		1.727
chr20	48732236	C	A	UBE2V1 (Medial and UTR)	UBE2V1:PHOS(0.634)PPI(0.822)REG(0.880)		3.100
chr20	48732234	A	C	UBE2V1 (Medial and UTR)	UBE2V1:PHOS(0.634)PPI(0.822)REG(0.880)		2.477
chr1	10187226	G	A	UBE4B (Intron)	UBE4B:PPI(0.826)		1.194
chr1	10205225	A	C	UBE4B (Intron)	UBE4B:PPI(0.826)		0.587
chr1	10197397	A	C	UBE4B (Intron)	UBE4B:PPI(0.826)		0.571
chr1	10093590	C	G	UBE4B (Promoter and UTR)	UBE4B:PPI(0.826)		1.991
chr8	103310982	A	G	UBR5 (Intron and Medial)	UBR5:PPI(0.838)		1.401
chr8	103291039	C	G	UBR5 (Intron and Promoter)	UBR5:PPI(0.838)		0.629
chr8	103298852	T	A	UBR5 (Intron)	UBR5:PPI(0.838)		1.216
chr8	103326720	T	C	UBR5 (Intron)	UBR5:PPI(0.838)		1.216
chr8	103323956	C	G	UBR5 (Intron)	UBR5:PPI(0.838)		1.216
chr8	103288061	A	G	UBR5 (Intron)	UBR5:PPI(0.838)		0.966
chr8	103287702	A	G	UBR5 (Intron)	UBR5:PPI(0.838)		0.593
chr8	103283511	T	C	UBR5 (Intron)	UBR5:PPI(0.838)		0.593

chr8	103327102	C	A	UBR5 (Intron)	UBR5:PPI(0.838)		0.593
chr8	103373989	C	A	UBR5 (Intron)	UBR5:PPI(0.838)		0.593
chr8	103276506	C	T	UBR5 (Intron)	UBR5:PPI(0.838)		0.593
chr8	103373437	G	C	UBR5 (Intron)	UBR5:PPI(0.838)		0.593
chr5	176402395	A	G	UIMC1 (Intron)	UIMC1:PPI(0.563)REG(0.409)		0.867
chr5	176402275	A	G	UIMC1 (Intron)	UIMC1:PPI(0.563)REG(0.409)		0.244
chr12	109540636	G	T	UNG (Intron)	UNG:PHOS(0.509)PPI(0.563)REG(0.880)		1.680
chr12	109540636	G	T	UNG (Intron)	UNG:PHOS(0.509)PPI(0.563)REG(0.880)		1.680
chr19	18974975	G	T	UPF1 (Intron)	UPF1:PPI(0.905)		1.545
chr19	18971322	T	C	UPF1 (Intron)	UPF1:PPI(0.905)		0.922
chr1	62902810	C	T	USP1 (UTR)	USP1:PPI(0.307)REG(0.409)		0.946
chr11	113701709	G	C	USP28 (Intron and Promoter)			0.188
chr11	113701362	G	A	USP28 (Intron and Promoter)			0.003
chr11	113692386	G	T	USP28 (Intron)			0.188
chr11	113698197	C	G	USP28 (Intron)			0.003
chr11	113685827	T	G	USP28 (Intron)			0.003
chr17	5035657	G	T	USP6 (Intron)	USP6:PPI(0.448)		1.353
chr17	5035657	G	T	USP6 (Intron)	USP6:PPI(0.448)		1.353
chr17	5051848	T	C	USP6 (Intron)	USP6:PPI(0.448)		0.791
chr17	5050510	C	T	USP6 (Intron)	USP6:PPI(0.448)		0.791
chr17	5042975	G	A	USP6 (Intron)	USP6:PPI(0.448)		0.353
chr17	5040665	C	T	USP6 (Intron)	USP6:PPI(0.448)		0.353
chr17	5049510	C	T	USP6 (Intron)	USP6:PPI(0.448)		0.168
chr17	5050359	C	G	USP6 (Intron)	USP6:PPI(0.448)		0.168
chr17	5036329	A	G	USP6 (Intron)	USP6:PPI(0.448)		0.168
chr17	5034086	C	T	USP6 (Intron)	USP6:PPI(0.448)		0.168
chr17	5038421	C	G	USP6 (Intron)	USP6:PPI(0.448)		0.168
chr17	5038683	C	G	USP6 (Intron)	USP6:PPI(0.448)		0.168
chr17	5045583	C	G	USP6 (Intron)	USP6:PPI(0.448)		0.168
chrX	41031320	T	G	USP9X (Intron)	USP9X:PPI(0.869)		1.656
chrX	41031320	T	G	USP9X (Intron)	USP9X:PPI(0.869)		1.656
chrX	41031320	T	G	USP9X (Intron)	USP9X:PPI(0.869)		1.656
chrX	41057778	T	A	USP9X (Intron)	USP9X:PPI(0.869)		1.464
chrX	41043944	T	C	USP9X (Intron)	USP9X:PPI(0.869)		1.464
chrX	41073963	G	A	USP9X (Intron)	USP9X:PPI(0.869)		1.279
chrX	41073966	C	T	USP9X (Intron)	USP9X:PPI(0.869)		1.279
chrX	41055615	G	A	USP9X (Intron)	USP9X:PPI(0.869)		1.279
chrX	41010337	C	T	USP9X (Intron)	USP9X:PPI(0.869)		1.279

chrX	40949692	G	A	USP9X (Intron)	USP9X:PPI(0.869)		1.279
chrX	41047223	C	A	USP9X (Intron)	USP9X:PPI(0.869)		1.279
chrX	41055729	G	A	USP9X (Intron)	USP9X:PPI(0.869)		1.227
chrX	41083904	A	C	USP9X (Intron)	USP9X:PPI(0.869)		0.841
chrX	41084534	G	C	USP9X (Intron)	USP9X:PPI(0.869)		0.656
chrX	40990991	C	G	USP9X (Intron)	USP9X:PPI(0.869)		0.656
chrX	40958292	A	C	USP9X (Intron)	USP9X:PPI(0.869)		0.656
chrX	41048460	T	G	USP9X (Intron)	USP9X:PPI(0.869)		0.656
chrX	40949719	C	G	USP9X (Intron)	USP9X:PPI(0.869)		0.656
chrX	41048752	G	C	USP9X (Intron)	USP9X:PPI(0.869)		0.656
chrX	41056594	A	C	USP9X (Intron)	USP9X:PPI(0.869)		0.656
chrX	40959413	A	C	USP9X (Intron)	USP9X:PPI(0.869)		0.656
chrX	41029995	A	G	USP9X (Intron)	USP9X:PPI(0.869)		0.656
chrX	40962413	C	G	USP9X (Intron)	USP9X:PPI(0.869)		0.656
chrX	40958639	C	T	USP9X (Intron)	USP9X:PPI(0.869)		0.656
chrX	41091872	A	T	USP9X (UTR)	USP9X:PPI(0.869)		1.279
chr11	75715041	C	G	UVRAG (Intron)	UVRAG:PHOS(0.276)PPI(0.142)REG(0.952)		1.043
chr9	35062972	C	T	VCP (Intron)	VCP:PPI(0.961)REG(0.634)		1.691
chr9	35061437	C	T	VCP (Intron)	VCP:PPI(0.961)REG(0.634)		1.506
chr9	35060955	T	C	VCP (Intron)	VCP:PPI(0.961)REG(0.634)		1.069
chr9	35065155	G	A	VCP (Intron)	VCP:PPI(0.961)REG(0.634)		0.883
chr9	35067664	C	T	VCP (Intron)	VCP:PPI(0.961)REG(0.634)		0.883
chr10	114574900	A	G	VTI1A (Intron)	VTI1A:PPI(0.787)REG(0.409)		0.688
chr2	128474631	G	A	WDR33 (Intron)	WDR33:PPI(0.850)		0.802
chr4	1906107	T	C	WHSC1 (Intron)	WHSC1:PPI(0.142)REG(0.634)		1.115
chr4	1929439	T	C	WHSC1 (Intron)	WHSC1:PPI(0.142)REG(0.634)		0.492
chr4	1926787	T	C	WHSC1 (Intron)	WHSC1:PPI(0.142)REG(0.634)		0.307
chr8	38204958	G	C	WHSC1L1 (Intron and Medial)	WHSC1L1:PHOS(0.276)PPI(0.606)		0.466
chr8	38204915	G	C	WHSC1L1 (Intron)	WHSC1L1:PHOS(0.276)PPI(0.606)		0.466
chr8	38172157	C	T	WHSC1L1 (Intron)	WHSC1L1:PHOS(0.276)PPI(0.606)		0.466
chr8	38134101	T	C	WHSC1L1 (Intron)	WHSC1L1:PHOS(0.276)PPI(0.606)		0.280
chr8	38133793	G	A	WHSC1L1 (Intron)	WHSC1L1:PHOS(0.276)PPI(0.606)		0.280
chr12	65514383	C	G	WIF1 (UTR)	WIF1:PHOS(0.920)PPI(0.718)		1.396
chr8	31009416	T	A	WRN (Intron)	WRN:PPI(0.814)REG(0.409)		0.549
chr8	31000310	G	T	WRN (Intron)	WRN:PPI(0.814)REG(0.409)		0.549
chr8	31009416	T	A	WRN (Intron)	WRN:PPI(0.814)REG(0.409)		0.549
chr8	31000310	G	T	WRN (Intron)	WRN:PPI(0.814)REG(0.409)		0.549
chr8	30915924	G	T	WRN (UTR)	WRN:PPI(0.814)REG(0.409)		0.654

chr8	30915924	G	T	WRN (UTR)	WRN:PPI(0.814)REG(0.409)		0.654
chr11	32416875	G	A	WT1 (Intron)	WT1:PHOS(0.509)PPI(0.753)REG(0.634)		0.636
chr11	32417060	C	T	WT1 (Intron)	WT1:PHOS(0.509)PPI(0.753)REG(0.634)		0.451
chr8	87410785	C	A	WWP1 (Intron)	WWP1:PHOS(0.885)PPI(0.860)		1.314
chr8	87470148	A	G	WWP1 (Intron)	WWP1:PHOS(0.885)PPI(0.860)		1.314
chr8	87386396	C	A	WWP1 (Intron)	WWP1:PHOS(0.885)PPI(0.860)		0.691
chr16	69967086	C	G	WWP2 (Intron)	WWP2:PPI(0.850)REG(0.880)		1.488
chr16	69959226	C	G	WWP2 (UTR)	WWP2:PPI(0.850)REG(0.880)		0.865
chr3	149375137	G	C	WWTR1 (UTR)	WWTR1:PHOS(0.709)PPI(0.641)		1.188
chr19	7688614	T	C	XAB2 (Intron)	XAB2:PPI(0.893)REG(0.830)		1.517
chr19	7687408	C	G	XAB2 (Intron)	XAB2:PPI(0.893)REG(0.830)		0.709
chr3	14208754	C	T	XPC (Intron)	XPC:PPI(0.897)REG(0.830)		1.341
chr3	14201364	G	A	XPC (Intron)	XPC:PPI(0.897)REG(0.830)		0.718
chr3	14208754	C	T	XPC (Intron)	XPC:PPI(0.897)REG(0.830)		1.341
chr3	14201364	G	A	XPC (Intron)	XPC:PPI(0.897)REG(0.830)		0.718
chr2	61715464	C	G	XPO1 (Intron)	XPO1:PHOS(0.775)PPI(0.993)REG(0.634)		1.602
chr2	61713179	G	C	XPO1 (Intron)	XPO1:PHOS(0.775)PPI(0.993)REG(0.634)		1.165
chr2	61710085	T	G	XPO1 (Intron)	XPO1:PHOS(0.775)PPI(0.993)REG(0.634)		1.165
chr2	61720953	G	T	XPO1 (Intron)	XPO1:PHOS(0.775)PPI(0.993)REG(0.634)		1.165
chr2	61727127	G	C	XPO1 (Intron)	XPO1:PHOS(0.775)PPI(0.993)REG(0.634)		0.980
chr2	61719942	A	G	XPO1 (Intron)	XPO1:PHOS(0.775)PPI(0.993)REG(0.634)		0.980
chr2	61760841	T	G	XPO1 (Intron)	XPO1:PHOS(0.775)PPI(0.993)REG(0.634)		0.980
chr2	61728951	G	T	XPO1 (Intron)	XPO1:PHOS(0.775)PPI(0.993)REG(0.634)		0.980
chr19	44056946	C	T	XRCC1 (Intron)	XRCC1:PHOS(0.895)PPI(0.641)		1.522
chr5	82576463	G	A	XRCC4 (Intron)	XRCC4:PHOS(0.959)PPI(0.729)REG(0.985)		0.955
chr5	82576463	G	A	XRCC4 (Intron)	XRCC4:PHOS(0.959)PPI(0.729)REG(0.985)		0.955
chr2	216987004	T	G	XRCC5 (Intron)	XRCC5:PPI(0.938)REG(0.634)		1.443
chr2	216990626	T	A	XRCC5 (Intron)	XRCC5:PPI(0.938)REG(0.634)		1.443
chr2	217069163	A	C	XRCC5 (Intron)	XRCC5:PPI(0.938)REG(0.634)		0.820
chr22	42046907	G	T	XRCC6 (Intron)	XRCC6:PHOS(0.276)PPI(0.993)		1.165

chr17	1248847	G	C	YWHAE (Intron)	YWHAE:PHOS(0.585)PPI(0.993)REG(0.634)		1.165
chr14	100728628	T	C	YY1 (Intron)	YY1:PHOS(0.540)PPI(0.798)REG(0.998)		1.618
chr14	100728628	T	C	YY1 (Intron)	YY1:PHOS(0.540)PPI(0.798)REG(0.998)		1.618
chr16	3338576	G	T	ZNF263 (Intron and Promoter)	ZNF263:PHOS(0.566)PPI(0.583)REG(0.997)		1.800
chr19	58717967	C	G	ZNF274 (Intron)	ZNF274:PPI(0.236)REG(0.990)		0.970
chr19	52472427	G	A	ZNF350 (Intron)	ZNF350:PPI(0.408)REG(0.761)		0.463
chr12	6781703	C	T	ZNF384 (Intron)	ZNF384:PHOS(0.276)PPI(0.540)REG(0.909)		1.369
chr18	22930917	A	C	ZNF521 (UTR)	ZNF521:PHOS(0.276)PPI(0.583)		1.068
chr1	78031572	C	G	ZZZ3 (Intron)	ZZZ3:PHOS(0.276)PPI(0.641)REG(0.986)		0.958
chr1	78044442	C	T	ZZZ3 (Intron)	ZZZ3:PHOS(0.276)PPI(0.641)REG(0.986)		0.958

Abbreviations: MOTIFBR = motif-breaking analysis; MOTIFG = motif-gaining analysis; PHOS = phosphorylation network; PPI = protein protein interaction network; REG = regulatory network; TF = transcription factor; UTR = untranslated region.
