# Supplementary material for: An integrative bioinformatics approach reveals coding and non-coding gene variants associated with gene expression profiles and outcome in breast cancer molecular subtypes
Source: Br J Cancer. 2018 Mar 21;118(8):1107–14. doi: 10.1038/s41416-018-0030-0 (PMC5931099; doi:10.1038/s41416-018-0030-0)
Supplement: Supplementary file 7 — Supplementary Table 6 [file 41416_2018_30_MOESM7_ESM.pdf]

**Supplementary Table 6. Prognostic value of genetic alterations in coding and non-coding regions in breast cancer subtypes**	
	**Univariate analysis**
**Gene**	***P*-value**
**ER-positive/HER2-negative breast cancers (*n* = 467)**	
**Variants in coding regions**	
ABCA13	0.95
CDH1	0.55
MAP3K1	0.43
MUC16	0.63
NEB	0.28
TAB3	0.64
TP53	0.34
**Variants in non-coding regions**	
AAK1	0.48
CA5A	0.31
CRTC3	0.21
CTNNA2	0.89
DOCK2	0.65
FAM118A	0.41
FASTKD1	0.68
HDLBP	0.81
HUS1	0.96
PDZD7	0.96
PPP1R12A	0.29
RYR3	0.53
STAG2	0.51
TMEM50A	0.45
TTC27	0.87
**ER-negative/HER2-negative breast cancers (*n* = 185)**	
**Variants in coding regions**	
MUC12	0.38
RYR2	0.14
**Variants in non-coding regions**	
CROCC	0.22
