# Supplementary material for: An integrative bioinformatics approach reveals coding and non-coding gene variants associated with gene expression profiles and outcome in breast cancer molecular subtypes
Source: Br J Cancer. 2018 Mar 21;118(8):1107–14. doi: 10.1038/s41416-018-0030-0 (PMC5931099; doi:10.1038/s41416-018-0030-0)
Supplement: Supplementary file 8 — Supplementary Table 7 [file 41416_2018_30_MOESM8_ESM.pdf]

Supplementary Table 7. Genes enclosed in the prognostic signatures related to variants in coding regions

ABCA13 signature ER+HER2-

Gene	*P*-value	Fold change
CYBA	2.96E-04	2.047
CST1	7.80E-04	2.425
EDEM2	5.72E-04	1.205
FKBP11	2.33E-04	1.633
GJC2	7.80E-04	1.798
KDSR	3.68E-04	0.759
LMAN2	7.91E-04	1.256
OSBPL1A	3.22E-04	0.594
PEX12	6.93E-04	0.718
RNASET2	1.52E-04	1.391
TFPT	2.33E-04	1.558
TGFB1	6.19E-04	1.430
THUMPD1	8.56E-04	0.774
ZNF787	9.67E-04	1.414

CDH1 signature ER+HER2-

Gene	*P* -value	Fold change
CLDN15	1.45E-09	2.292
KANK3	7.84E-08	2.500
PSMD14	1.38E-07	0.647
MAP2K7	1.59E-07	1.489
PVRIG	1.92E-07	2.219
NBEAL2	2.46E-07	1.540
RASGRP2	3.58E-07	3.378
ZNF276	5.76E-07	1.686
SFRS16	6.58E-07	1.965
AGER	8.33E-07	2.350
CDH1	9.96E-07	0.245
PSMD12	1.07E-06	0.638
GRAP	1.20E-06	1.917
SFRS5	1.24E-06	1.495
DDX1	1.24E-06	0.757
MLL4	1.32E-06	1.437
PPM1F	2.02E-06	1.448
ZBTB48	2.30E-06	1.374
DOT1L	2.35E-06	1.521
ARHGEF18	2.53E-06	1.511
HSP90B1	2.56E-06	0.700
DOCK6	2.75E-06	1.405
KIAA1279	2.80E-06	0.740
TXNDC9	2.92E-06	0.804
ITPKB	3.12E-06	1.313
RCN2	3.25E-06	0.754
CRTC1	3.31E-06	1.560
HMHA1	3.53E-06	1.750
RAB1A	3.85E-06	0.823
LTBP4	4.12E-06	2.265
MFNG	4.32E-06	2.259
PSMD11	4.37E-06	0.688
PFN2	4.37E-06	0.456
HDAC7	4.57E-06	1.477
NISCH	4.65E-06	1.394
VPS26A	4.78E-06	0.781
RTN3	5.20E-06	0.722
CDC27	5.24E-06	0.722
CCT2	5.39E-06	0.687
PTCH2	5.80E-06	2.902
NOTCH4	5.87E-06	1.878
DDX52	6.15E-06	0.728
FLT4	6.24E-06	2.017
MTCH2	6.24E-06	0.699
PHF1	6.33E-06	1.392
PRKRA	6.42E-06	0.806
RPN2	6.56E-06	0.689
PDIA6	6.59E-06	0.754

CDH1 signature ER+HER2-

LAT	6.80E-06	2.729
SETD4	6.87E-06	1.246
TAL1	6.97E-06	1.937
CIC	6.97E-06	1.280
TJAP1	7.20E-06	1.345
USP20	8.23E-06	1.476
EIF5B	8.29E-06	0.678
LYL1	8.53E-06	2.728
RALGDS	8.53E-06	1.462
P4HA1	8.87E-06	0.691
SMAD6	9.08E-06	1.915
GPD2	1.03E-05	0.531
HSPA8	1.06E-05	0.627
DARC	1.06E-05	3.146
TNFRSF25	1.07E-05	2.515
PSMD1	1.07E-05	0.757
MEOX1	1.09E-05	2.844
TYK2	1.13E-05	1.440
INPP5B	1.14E-05	1.386
CCDC130	1.15E-05	1.547
SIN3B	1.17E-05	1.491
ZAP70	1.21E-05	3.641
PKD1	1.22E-05	1.359
ARHGEF15	1.27E-05	1.861
MAST3	1.31E-05	1.504
BTN2A1	1.38E-05	1.286
CSE1L	1.38E-05	0.687
ARHGEF1	1.47E-05	2.000
B3GALNT1	1.50E-05	0.628
GNG7	1.58E-05	1.747
ABCD4	1.74E-05	1.300
TSC22D4	1.75E-05	1.467
C19orf6	1.75E-05	1.656
PRKD2	1.76E-05	1.395
ANXA7	1.78E-05	0.838
KIAA0391	1.81E-05	0.798
TMED2	1.83E-05	0.696
GCLM	1.84E-05	0.714
MGAT3	1.88E-05	2.166
GPR132	1.88E-05	2.315
MIER2	1.97E-05	1.253
SSB	1.97E-05	0.818
GRAP2	2.05E-05	2.512
NSF	2.07E-05	0.672
NRD1	2.17E-05	0.825
YWHAB	2.25E-05	0.808
GLTSCR1	2.25E-05	1.510
PTPRCAP	2.31E-05	3.195
DNM2	2.34E-05	1.236
FBXO22	2.51E-05	0.773
ZMPSTE24	2.52E-05	0.759
SH3BP2	2.60E-05	1.287

MAP3K1 signature ER+HER2+

Gene	*P*-value	Fold change
ACSM3	1.89E-04	1.687
ALG3	3.72E-04	0.749
AP1S1	4.14E-05	0.686
ARF1	3.00E-04	0.741
ARGLU1	8.56E-04	1.473
ARPC5L	7.72E-04	0.772
ASL	9.13E-04	0.773
BCL10	9.36E-04	1.154
BLNK	4.45E-04	1.402
C17orf48	1.23E-04	1.251
C17orf68	2.87E-04	1.437
C17orf85	5.20E-04	1.220
C20orf20	5.53E-04	0.772
CDT1	5.27E-04	0.515
CEP68	4.49E-04	1.299
CLK1	8.99E-04	1.349
CLK4	6.44E-05	1.359
CNPY2	4.02E-05	0.739
COPG	3.40E-05	0.738
CPA3	6.26E-05	2.839
CRELD2	1.40E-04	0.611
CTSA	6.12E-04	0.722
DCUN1D3	1.17E-04	1.353
DDX56	6.84E-04	0.825
DHCR7	2.85E-05	0.605
ECE2	7.07E-04	0.707
EDEM2	5.35E-05	0.801
EFEMP1	4.00E-04	1.917
ELMO3	1.51E-04	0.684
ENTPD6	3.87E-04	0.692
ERGIC3	6.54E-04	0.847
FDPS	2.71E-04	0.742
FLAD1	2.40E-04	0.747
GUSB	5.11E-04	0.760
HAX1	4.37E-04	0.728
HEATR2	7.59E-04	0.854
HEXA	7.95E-04	0.818
HIST1H2BC	6.41E-04	0.334
HIST1H2BK	6.54E-04	0.642
JAK1	2.86E-04	1.451
JTB	3.87E-04	0.758
KIAA0430	5.06E-05	1.305
KITLG	6.06E-05	2.077
KL	6.74E-04	1.981
LGALS3BP	8.94E-04	0.611
LLGL2	4.14E-04	0.657
LSM14B	9.09E-04	0.854
LSR	4.45E-04	0.741

MAP3K1 signature ER+HER2+

MAP3K1	1.75E-04	1.804
MAPK8IP2	8.37E-04	0.497
METT10D	5.61E-04	1.213
MOCS3	8.65E-04	0.805
MOSC1	1.39E-04	0.438
NDP	6.22E-04	8.059
NEU1	5.13E-04	0.783
NUDCD3	9.41E-04	0.854
PKIA	7.40E-04	2.405
PLOD3	1.55E-04	0.711
PODXL2	8.40E-04	0.527
PRDM2	4.98E-04	1.294
PSMA7	2.94E-04	0.780
PSMB5	1.72E-04	0.781
PSMD2	1.56E-04	0.848
PTPN21	3.22E-04	1.675
PYCR1	9.09E-04	0.664
RAB31	2.01E-04	1.994
RAI14	7.55E-05	1.412
RAI2	3.90E-05	1.949
RGNEF	8.00E-04	1.475
RLF	4.78E-04	1.329
RNF187	5.06E-04	0.775
RPN1	1.74E-05	0.778
RUSC1	5.36E-04	0.684
SCAMP3	1.05E-04	0.689
SEC61A1	9.96E-04	0.875
SLC14A1	5.85E-04	1.924
SMG1	8.47E-04	1.431
SMG6	4.11E-05	1.243
SNX29	5.26E-05	1.380
SOCS2	2.91E-05	2.457
SRPRB	7.95E-04	0.790
SYNGR2	7.05E-04	0.767
TAOK3	3.11E-04	1.223
THUMPD1	7.34E-04	1.255
TM9SF1	2.63E-04	0.794
TMEM208	5.36E-04	0.751
TNFRSF11B	3.74E-06	3.179
TOR3A	5.26E-04	0.728
TRIM13	2.27E-04	1.246
VTCN1	2.21E-04	4.048
ZNF292	2.21E-04	1.401
ZNF83	6.27E-04	1.432

MUC16 signature ER+HER2-

Gene	*P*-value	Fold change
BCL7A	3.50E-04	0.869
DHCR24	9.12E-04	0.668
LRRC31	8.04E-04	5.406
PAIP2B	5.19E-04	0.663
PHLDA1	5.59E-04	0.707
TRAFD1	1.18E-04	0.826
ZNF202	8.74E-04	1.219

NEB signature ER+HER2-

Gene	*P*-value	Fold change
AP4M1	3.98E-04	1.381
BMS1	7.06E-04	0.850
CCDC47	9.19E-04	0.717
GTF2E2	5.36E-04	1.169
LOC92973	7.82E-04	2.099
MOSPD3	3.93E-04	1.344
NETO2	9.36E-04	0.525
SP2	8.78E-04	0.865

TAB3 signature ER+HER2-

Gene	*P* -value	Fold change
R3HDM1	2.50E-06	1.423
TFG	2.66E-06	1.367
ZNF219	1.34E-05	0.548
DEF6	1.48E-05	0.665
CUL1	1.71E-05	1.246
USP39	1.83E-05	1.217
ZNF143	1.99E-05	1.138
P4HA1	2.16E-05	1.393
KCMF1	2.17E-05	1.272
KIAA1279	2.26E-05	1.253
MINPP1	2.96E-05	1.307
HERC4	3.02E-05	1.256
C1orf115	3.57E-05	0.409
PTCD3	3.84E-05	1.259
RING1	4.07E-05	0.823
SLC6A12	4.25E-05	2.036
GCDH	4.68E-05	0.614
SNX24	5.38E-05	1.344
ZBTB17	5.48E-05	0.730
RHOBTB2	5.52E-05	0.646
RIPK2	5.70E-05	1.465
NCDN	5.81E-05	0.745
CLDN5	6.46E-05	0.507
BIN1	6.54E-05	0.552
NCKAP1	6.62E-05	1.316
ARHGEF18	6.64E-05	0.815
CLIP1	6.90E-05	1.230
MPHOSPH10	7.20E-05	1.171
EMID1	7.76E-05	0.302
GADD45G	7.85E-05	0.588
B9D2	8.09E-05	0.686
CROCC	8.24E-05	0.607
TNXB	8.49E-05	0.427
PTK2B	8.56E-05	0.707
GPATCH3	8.77E-05	0.764
PRPF40A	9.19E-05	1.260
BRF1	9.50E-05	0.755
ECD	1.04E-04	1.143
VILL	1.06E-04	0.615
ALPL	1.23E-04	0.522
LTBP4	1.29E-04	0.714
CLEC3B	1.30E-04	0.596
USP20	1.31E-04	0.822
SEMA3G	1.32E-04	0.432
TRIOBP	1.32E-04	0.759
KANK3	1.33E-04	0.667
DNM1L	1.36E-04	1.231
APBA3	1.41E-04	0.699

TAB3 signature ER+HER2-

LSM14A	1.42E-04	1.174
LDHA	1.46E-04	1.488
CFD	1.51E-04	0.400
SAFB	1.53E-04	0.880
SNX2	1.63E-04	1.276
UBTF	1.64E-04	0.886
KPNA1	1.93E-04	1.258
C21orf2	1.93E-04	0.754
HNRNPM	2.07E-04	0.881
RAMP3	2.16E-04	0.542
NRXN2	2.18E-04	0.490
CCS	2.18E-04	0.661
ECHDC2	2.22E-04	0.637
MAGEH1	2.32E-04	0.753
RRP1	2.33E-04	0.832
RASIP1	2.38E-04	0.643
ZBTB22	2.43E-04	0.781
NCOA6	2.45E-04	1.243
MED21	2.50E-04	1.140
TFPT	2.52E-04	0.683
NOSIP	2.57E-04	0.720
BCAM	2.57E-04	0.509
CCDC85B	2.59E-04	0.579
MRPL19	2.61E-04	1.221
OSGIN1	2.70E-04	0.510
CHMP6	2.72E-04	0.731
PICALM	2.78E-04	1.203
CDK10	2.89E-04	0.700
MED16	2.89E-04	0.663
INTS1	2.90E-04	0.739
TRPM4	3.02E-04	0.623
ZBTB48	3.05E-04	0.700
AZI1	3.08E-04	0.703
MEOX1	3.19E-04	0.340
HDAC7	3.20E-04	0.826
TNFRSF14	3.31E-04	0.697
EFNB1	3.37E-04	0.757
EIF3J	3.39E-04	1.145
XPO1	3.41E-04	1.276
PIN1	3.50E-04	0.736
WASL	3.52E-04	1.214
GLRX3	3.53E-04	1.237
TYK2	3.53E-04	0.863
ATP13A2	3.55E-04	0.670
TMEM160	3.69E-04	0.671
SFRS16	3.74E-04	0.718
EIF4G2	3.75E-04	1.204
MGAT3	3.78E-04	0.560
ST7	3.78E-04	1.229
CCHCR1	3.85E-04	0.720
MXD4	3.85E-04	0.723
SPG21	3.86E-04	1.161

TP53 signature ER+HER2-

Gene	*P*-value	Fold change
TUBA1C	3.51E-16	1.601
PSMB2	2.57E-14	1.367
KIF2C	4.99E-14	2.291
EDA2R	6.06E-14	0.472
MYBL2	8.79E-14	2.864
CDC20	1.08E-13	2.136
CCNG1	2.24E-13	0.658
DDB2	8.89E-13	0.682
CBX2	1.44E-12	4.120
ENO1	4.00E-12	1.490
RFC4	5.21E-12	1.491
ANKRA2	5.92E-12	0.747
C1orf135	7.43E-12	2.164
CDCA8	7.94E-12	1.725
DDX27	8.44E-12	1.388
CCT5	9.70E-12	1.529
XPC	1.28E-11	0.783
CENPN	1.40E-11	1.663
RAD54L	1.43E-11	2.000
UBE2C	1.55E-11	2.163
NUP93	1.84E-11	1.480
PLK1	3.22E-11	2.196
TUBB	3.30E-11	1.365
ORC1L	5.18E-11	2.100
EIF4G1	5.25E-11	1.269
C14orf45	5.62E-11	0.579
PSMD2	5.83E-11	1.301
LRP8	5.88E-11	2.009
VAMP2	6.62E-11	0.659
TPX2	8.91E-11	1.983
LRRC48	1.10E-10	0.424
APH1B	1.10E-10	0.622
AURKA	1.13E-10	2.192
MED8	1.42E-10	1.188
STIP1	1.44E-10	1.438
NRD1	1.49E-10	1.256
ALG3	1.56E-10	1.381
E2F2	1.60E-10	2.124
ALS2CR8	1.81E-10	0.654
G6PD	1.89E-10	1.451
CENPA	1.94E-10	1.904
ECE2	2.07E-10	1.510
POLI	2.11E-10	0.649
YARS	2.11E-10	1.360
CCT6A	2.18E-10	1.295
KIF4A	2.23E-10	1.934
FOXM1	2.25E-10	1.974
CIRBP	2.45E-10	0.720

TP53 signature ER+HER2-

SHMT2	2.62E-10	1.375
CDC25A	2.70E-10	2.122
GPI	2.81E-10	1.366
PRDX1	3.04E-10	1.480
EZH1	3.24E-10	0.739
SNRPA1	3.43E-10	1.406
OIP5	3.76E-10	1.692
KIAA0141	4.12E-10	0.806
CCNB2	4.25E-10	1.929
TRIP13	4.43E-10	2.125
SRM	4.55E-10	1.406
GARS	4.60E-10	1.400
POP1	5.16E-10	1.612
MAP2K4	5.27E-10	0.733
ORC6L	5.63E-10	2.156
C17orf48	6.44E-10	0.721
CTSA	7.68E-10	1.315
CDCA3	8.84E-10	1.692
SRD5A1	9.46E-10	1.616
NCAPH	1.03E-09	1.880
TIMM8A	1.05E-09	1.384
CDK4	1.18E-09	1.230
PSMD7	1.34E-09	1.364
NOC2L	1.46E-09	1.454
KIF23	1.51E-09	1.713
CHEK2	1.52E-09	1.412
EIF2S2	1.57E-09	1.305
CEP55	1.69E-09	1.879
STIL	1.78E-09	1.696
BIRC5	1.79E-09	1.960
RPP25	1.88E-09	1.916
MCM2	1.89E-09	1.614
TROAP	1.94E-09	1.946
C20orf20	1.95E-09	1.378
POLQ	2.04E-09	2.028
DNMT3B	2.09E-09	1.841
PKMYT1	2.12E-09	2.096
IDH2	2.22E-09	1.487
DEPDC1	2.32E-09	2.131
PIR	2.53E-09	1.940
SLC7A5	2.59E-09	2.485
SHCBP1	2.62E-09	1.801
HHAT	2.73E-09	0.675
PGK1	2.79E-09	1.474
PHF7	2.79E-09	0.610
PSMA7	2.93E-09	1.374
MCM6	2.95E-09	1.428
ERCC6L	3.27E-09	1.684
PTPRT	3.45E-09	0.222
CENPI	3.60E-09	1.943
C17orf68	3.63E-09	0.727
FRY	3.63E-09	0.696

MUC12 signature ER-HER2-

Gene	*P* -value	Fold change
ANXA2	5.20E-04	0.507
ANXA2P1	7.84E-04	0.575
AP4E1	1.14E-04	0.640
CLEC5A	1.25E-04	0.257
CNPY3	3.44E-04	1.504
DIO2	7.84E-04	0.311
DTWD1	8.92E-04	0.778
KIAA0913	8.47E-04	1.546
MAPK6	5.29E-04	0.632
OSR2	3.98E-04	0.327
PNMA3	1.90E-04	4.681

RYR2 signature ER-HER2-

Gene	*P* -value	Fold change
ALCAM	8.22E-04	0.339
BTN3A2	6.63E-04	1.906
HLA-B	6.40E-04	1.783
LMF1	4.27E-04	0.506
PDGFD	5.53E-04	0.428
SOBP	5.14E-04	0.351
STAT1	7.94E-04	2.518
TAP1	3.54E-04	2.235
TAP2	8.67E-04	1.833
WARS	5.90E-04	2.583
