# Supplementary material for: An integrative bioinformatics approach reveals coding and non-coding gene variants associated with gene expression profiles and outcome in breast cancer molecular subtypes
Source: Br J Cancer. 2018 Mar 21;118(8):1107–14. doi: 10.1038/s41416-018-0030-0 (PMC5931099; doi:10.1038/s41416-018-0030-0)
Supplement: Supplementary file 9 — Supplementary Table 8 [file 41416_2018_30_MOESM9_ESM.pdf]

Supplementary Table 8. Genes enclosed in the prognostic signatures related to variants in non-coding regions

AAK1 signature ER+HER2-

Gene	*P*-value	Fold change
ANAPC10	1.30E-04	1.251
ARHGEF18	9.66E-05	0.808
ARL6IP4	6.17E-04	0.703
ARMC8	8.94E-04	1.162
ASCC1	2.02E-05	1.275
ASMTL	3.76E-04	0.721
ATAD2	3.34E-04	1.726
BIN1	5.44E-04	0.645
BIN3	5.18E-04	0.724
C17orf59	8.96E-04	0.663
C21orf2	6.47E-05	0.737
CARD10	3.57E-04	0.688
CCDC25	5.85E-04	0.728
CCRN4L	5.63E-05	1.432
CENPQ	2.10E-04	1.363
CHMP7	1.67E-04	0.750
CLDN5	4.56E-04	0.419
CLN8	2.00E-05	0.634
COX7A1	9.24E-04	0.510
CROCC	3.20E-05	0.547
CTDP1	1.33E-05	0.734
DDX52	7.49E-04	1.315
DNAJC9	9.64E-04	1.272
DPM1	6.13E-04	1.301
EIF2S1	4.26E-04	1.157
ELMO2	4.68E-04	1.274
ELOVL6	6.83E-04	1.626
EMILIN1	2.28E-04	0.488
EPHX2	7.96E-04	0.591
FBXL8	2.23E-05	0.546
FKBP2	5.26E-04	0.569
FLT4	5.14E-04	0.671
GDPD5	5.17E-04	0.713
GLO1	9.43E-04	1.520
INF2	1.68E-05	0.605
KHDRBS1	6.37E-04	1.099
KIF15	2.57E-04	1.859
KIF18A	3.76E-04	1.639
MAD1L1	6.93E-04	0.774
MAFK	2.85E-04	0.671
MCM6	7.92E-04	1.329
MLYCD	5.46E-04	0.805
MPG	7.46E-05	0.637
MXD4	5.63E-04	0.749
NOC3L	6.47E-05	1.239
NOL11	6.63E-04	1.270

AAK1 signature ER+HER2-

NUDT18	2.67E-06	0.483
P4HA1	7.00E-04	1.349
PBXIP1	3.06E-04	0.654
PDLIM2	4.36E-04	0.687
PLD2	3.33E-04	0.745
POLR2D	7.58E-04	1.344
POLR2K	8.70E-04	1.487
POLR3D	8.22E-04	0.801
PRMT2	4.31E-04	0.804
PSMD12	1.03E-04	1.325
PTK2B	5.88E-04	0.716
RCN2	8.04E-04	1.178
RHOBTB2	1.95E-05	0.578
RPS3	9.47E-04	0.754
RRBP1	3.48E-04	0.708
SLC38A10	2.82E-04	0.665
SMNDC1	2.56E-04	1.254
SORBS3	3.22E-04	0.712
STK11	3.17E-04	0.804
TFG	9.82E-04	1.205
THUMPD2	3.54E-04	1.210
TLK2	4.17E-04	1.251
TRADD	3.71E-04	0.704
TRIOBP	1.63E-04	0.726
TRPM4	2.32E-04	0.604
TUBGCP5	3.27E-04	1.152
UBE2W	3.94E-04	1.456
ZBTB7B	9.29E-04	0.681
ZNF143	2.18E-06	1.199
ZNF219	1.69E-04	0.561

CA5A signature ER+HER2-

Gene	*P*-value	Fold change
ABAT	6.83E-04	1.658
CCDC25	6.41E-04	1.435
DDX19A	4.31E-05	0.816
INTS9	3.91E-04	1.464
IQCK	5.20E-04	1.462
KCNAB2	5.21E-04	0.485
LMAN2L	8.70E-04	1.336
ORAI3	8.40E-04	1.459
POLR2C	5.63E-04	0.796
RHBDF1	9.65E-04	1.472
SF3B3	2.63E-04	0.779
STC2	9.97E-04	3.302
SYK	5.37E-04	0.563

CRTC3 signature ER+HER2-

Gene	*P* -value	Fold change
AMDHD2	2.34E-05	0.725
ANAPC2	3.71E-05	0.796
APBA3	6.21E-06	0.707
ARFGAP1	1.55E-05	0.788
ARFRP1	8.04E-07	0.750
ARHGEF18	2.44E-05	0.801
ARL6IP4	1.18E-06	0.717
ARMC6	2.95E-05	0.772
ASPSCR1	5.94E-07	0.710
ATAD3A	2.13E-06	0.661
ATXN10	2.03E-05	1.272
AURKAIP1	1.50E-06	0.664
BBC3	7.99E-06	0.617
C19orf60	1.93E-05	0.590
CASKIN2	2.11E-05	0.764
CCDC9	4.46E-08	0.570
CCDC94	2.60E-05	0.781
CDC34	2.14E-06	0.695
CDC37	2.72E-07	0.740
CHMP6	9.03E-06	0.826
CROCC	2.41E-06	0.531
CSNK1G3	7.87E-07	1.334
CTDP1	3.87E-07	0.702
DAPK3	1.81E-05	0.753
DAZAP1	1.25E-05	0.794
DDX54	1.90E-06	0.806
DRAP1	1.65E-05	0.683
DUS1L	3.81E-06	0.700
E4F1	2.40E-05	0.721
EHBP1L1	2.33E-05	0.624
EHD1	3.11E-05	0.761
EIF3G	1.03E-05	0.787
EPN1	2.52E-06	0.765
ESRRA	6.96E-06	0.738
FBXW11	2.44E-05	1.239
FKBP2	1.79E-06	0.541
FKBP8	6.74E-06	0.725
GADD45GIP1	1.93E-05	0.701
GDPD5	3.84E-05	0.668
GIPC1	4.00E-06	0.712
GLTSCR2	3.41E-05	0.661
HGS	1.45E-06	0.761
HIP1R	5.76E-06	0.662
ICAM3	8.28E-07	0.707
INF2	3.29E-06	0.635
IRF3	9.65E-07	0.672
KIAA1279	2.68E-06	1.275
KIF1C	2.68E-05	0.668

CRTC3 signature ER+HER2-

KRI1	6.49E-06	0.737
MAD1L1	3.63E-05	0.764
MAFK	9.30E-06	0.665
MAP3K11	3.12E-05	0.743
MAPK9	3.39E-06	1.257
MRPL12	1.17E-05	0.745
NARFL	3.72E-05	0.737
NCKAP1	2.98E-05	1.273
NFKB2	2.22E-05	0.711
NOS3	1.49E-05	0.659
NOSIP	1.04E-05	0.723
NR1H2	2.56E-05	0.743
NT5C	1.09E-06	0.692
NUBP2	2.75E-05	0.681
OGFR	2.34E-06	0.651
PIN1	3.11E-05	0.745
PPAN	1.06E-07	0.586
PPP3CB	9.41E-06	1.225
PRPF6	4.68E-06	0.762
PUS1	5.04E-06	0.722
RHOT2	2.78E-05	0.705
RPS3	3.80E-06	0.727
RRP1	9.86E-09	0.698
RRP9	2.99E-06	0.723
SAC3D1	2.99E-05	0.693
SAFB	1.14E-07	0.783
SAFB2	3.71E-06	0.796
SART1	3.51E-07	0.600
SERPINF2	3.63E-05	0.500
SIRT7	3.14E-06	0.705
SLC12A9	2.14E-06	0.722
SLC38A10	6.87E-07	0.631
SNCG	2.23E-05	0.303
SNX2	8.67E-06	1.262
SUPT5H	4.55E-06	0.784
TAF1B	1.94E-05	1.206
TBC1D19	8.65E-06	1.322
TIMM44	8.40E-06	0.760
TNIP2	6.24E-06	0.834
TRADD	1.52E-05	0.727
TRAF2	1.70E-05	0.751
TSC22D4	9.44E-06	0.682
TSR2	1.83E-06	0.789
WDR4	2.40E-05	0.784
ZBTB17	7.92E-06	0.728
ZCCHC4	3.52E-05	1.217
ZGPAT	1.84E-07	0.611
ZNF143	9.41E-06	1.126
ZNF205	3.34E-05	0.671
ZNF219	1.17E-06	0.554
ZNF593	6.70E-06	0.722
ZNF787	8.32E-08	0.640

CTNNA2 signature ER+HER2-

Gene	*P*-value	Fold change
AP4M1	9.07E-04	1.196
ATP5I	7.02E-04	1.380
BET1L	5.40E-05	1.248
C11orf24	2.99E-04	1.418
GEMIN8	7.39E-04	1.290
IFNAR1	5.31E-04	0.842
IFNAR2	8.87E-04	0.736
KDELR1	9.85E-04	1.167
MAP3K4	2.04E-04	0.839
MGST3	3.72E-04	1.459
MOSPD3	9.98E-04	1.249
MRPL17	2.17E-04	1.264
OSTM1	4.16E-05	0.728
PSENEN	1.03E-04	1.415
RAB3D	8.62E-04	1.404
RNGTT	6.88E-04	0.757
RPLP2	3.47E-04	1.422
RRAGD	9.68E-04	0.657
SHARPIN	3.60E-04	1.487
TAF10	2.28E-04	1.478
ZC3H3	4.67E-04	1.381

DOCK2 signature ER+HER2-

**Gene**	***P*-value**	**Fold change**
NEIL1	8.08E-04	0.647
PCYT1A	6.04E-04	1.230
PDK3	5.27E-05	1.351

FAM118A signature ER+HER2-

Gene	*P*-value	Fold change
ABI2	2.90E-04	1.250
ARFGAP1	2.62E-04	0.801
ARFRP1	9.22E-05	0.780
ARHGEF5	3.66E-04	0.668
ARL5A	1.65E-04	1.205
ARL6IP4	9.53E-05	0.722
ASPSCR1	1.40E-04	0.738
ATAD3A	2.18E-05	0.662
AURKAIP1	6.37E-05	0.674
AXIN1	1.52E-04	0.694
AZI2	1.43E-04	1.244
BANP	5.59E-04	0.791
BBC3	3.11E-04	0.554
CALM1	5.88E-04	1.253
CCDC9	2.28E-05	0.572
CDC37	1.21E-04	0.760
CENPQ	3.97E-04	1.289
CNTLN	2.16E-04	1.602
COPS2	1.22E-04	1.138
CPSF3L	4.21E-04	0.746
CROCC	4.17E-04	0.627
CTDP1	1.67E-04	0.739
DAPK3	6.16E-04	0.787
DDX54	1.93E-04	0.810
DUS1L	3.56E-04	0.711
E4F1	1.82E-04	0.792
EIF2B4	1.86E-04	0.836
EIF3G	2.84E-04	0.814
EPN1	2.15E-04	0.748
ERBB2	8.60E-05	0.698
FAM149B1	2.34E-04	1.259
FKBP2	3.81E-04	0.569
GCDH	2.66E-04	0.823
GLTPD1	4.82E-04	0.740
GNB1L	4.30E-04	0.720
GPATCH3	3.25E-04	0.812
GPN3	3.23E-04	1.189
HCFC2	1.15E-04	1.332
HNRNPM	1.73E-04	0.836
ICAM3	4.95E-04	0.745
IRF3	2.83E-04	0.771
ITCH	2.78E-05	1.265
KIAA1279	5.35E-04	1.246
KRI1	2.65E-04	0.790
LAS1L	6.08E-04	0.809
LONP1	5.52E-04	0.806
MAPK14	5.18E-04	1.132
MPG	5.92E-04	0.732

FAM118A signature ER+HER2-

NARFL	3.71E-04	0.720
NCKAP1	3.37E-06	1.341
NFKB2	3.56E-04	0.779
NOC2L	1.64E-04	0.825
NUBP2	2.52E-04	0.662
OGFR	5.58E-05	0.659
PAF1	2.93E-04	0.815
PJA2	3.65E-04	1.341
PPAN	1.43E-05	0.610
PPM1G	4.83E-04	0.840
PPP3CB	3.23E-05	1.266
PRKD2	2.50E-04	0.771
PRPF6	2.67E-04	0.776
PSIP1	4.04E-04	1.373
PTPN11	1.21E-04	1.289
PUS1	7.36E-05	0.689
RAB11FIP2	3.46E-04	1.341
RHOT2	2.83E-04	0.715
RPS3	5.27E-04	0.754
RRP1	1.28E-06	0.713
RRP9	2.31E-05	0.731
SAFB	1.12E-05	0.780
SAFB2	2.97E-04	0.809
SCRN3	2.01E-04	1.246
SH2D3A	4.57E-04	0.717
SIRT7	1.99E-04	0.708
SLC12A9	3.21E-04	0.754
SLC35A5	5.87E-04	1.287
SMPD2	3.21E-05	0.695
SNAPC4	4.41E-04	0.809
STX4	3.34E-04	0.700
SUPT5H	7.54E-05	0.838
TBC1D19	2.11E-04	1.312
TBL3	4.19E-04	0.755
TCF12	8.42E-05	1.274
TCOF1	1.17E-04	0.761
TM9SF3	4.98E-04	1.282
TOMM40	6.36E-04	0.787
TRAPPC6A	1.93E-04	0.741
TRMT1	3.13E-04	0.757
TSC22D4	4.75E-04	0.683
TSR2	2.88E-04	0.813
TSSC4	9.22E-05	0.767
TUFM	1.81E-04	0.771
ZBTB17	2.76E-04	0.784
ZBTB48	5.04E-04	0.728
ZGPAT	1.55E-05	0.657
ZNF205	3.17E-04	0.651
ZNF212	1.84E-04	0.792
ZNF394	3.20E-04	0.863
ZNF593	1.07E-04	0.739
ZNF787	8.29E-05	0.682

FASTKD1 signature ER+HER2-

Gene	*P*-value	Fold change
ARHGEF18	3.50E-05	0.785
ASCC1	4.75E-05	1.242
ATP5C1	7.51E-04	1.281
CCDC9	2.34E-04	0.665
CHMP7	1.38E-04	0.736
CLDN5	3.17E-04	0.440
CMAS	9.74E-04	1.246
CROCC	4.30E-04	0.573
DDX1	8.63E-04	1.148
DNAJB6	8.46E-04	1.291
ECD	2.59E-04	1.155
EML3	5.21E-04	0.654
EPHX1	1.58E-04	0.643
FAM46A	2.35E-04	1.600
GBE1	4.75E-04	1.300
GLTSCR2	7.40E-04	0.619
HERC4	5.39E-04	1.164
HNRNPM	5.54E-04	0.826
HPRT1	4.43E-04	1.489
IGFBP4	7.61E-04	0.690
INF2	1.05E-04	0.594
IRF3	7.87E-04	0.704
KIAA1279	3.70E-04	1.270
LTBP4	5.09E-04	0.746
MRPS35	8.61E-04	1.192
NARS	8.21E-04	1.236
P4HA1	2.52E-05	1.369
PFDN4	3.58E-04	1.315
PIGP	4.39E-04	1.315
PPP1R3D	4.30E-04	1.463
PTCD3	9.40E-04	1.136
PTK2B	2.83E-04	0.675
PVRIG	9.99E-04	0.625
RHOBTB2	4.01E-05	0.648
SAFB	6.60E-05	0.789
SAFB2	2.55E-04	0.785
SEH1L	5.56E-04	1.155
SEMA3G	5.39E-04	0.506
STARD3	4.44E-04	0.791
TBKBP1	4.13E-04	0.615
TFG	3.32E-04	1.431
TMEM160	3.40E-04	0.665
TRIOBP	8.39E-04	0.745
TRPM4	7.92E-04	0.521
TSG101	9.43E-04	1.210
YWHAE	2.33E-04	1.248
ZBTB17	3.34E-04	0.684
ZNF219	4.39E-04	0.485

HDLBP signature ER+HER2-

Gene	*P*-value	Fold change
CIB1	6.33E-04	0.761
CTSD	9.36E-04	0.636
EDF1	9.02E-04	0.785
MAN1B1	7.12E-04	0.753
NR1H3	5.39E-04	0.724
PMPCA	9.39E-04	0.782
PPIB	9.93E-04	0.749
RRP1	2.78E-04	0.720
YIPF2	9.02E-04	0.774
ZNF136	3.68E-04	1.275

HUS1 signature ER+HER2-

Gene	*P*-value	Fold change
KIN	7.74E-04	1.123
PPL	9.10E-04	0.612
SSB	9.12E-04	1.165
ZNF143	9.45E-04	1.133

PDZD7 signature ER+HER2-

Gene	*P* -value	Fold change
FGF2	9.23E-04	0.528
GALNT14	9.68E-04	3.674
PTGDS	9.90E-04	0.356

PPP1R12A signature ER+HER2-

Gene	*P* -value	Fold change
ACVRL1	4.44E-04	0.653
B3GALNT1	1.37E-04	1.731
CABYR	3.15E-05	2.590
CD97	7.73E-04	0.628
CENPQ	7.53E-04	1.345
CHST2	5.81E-04	0.663
CLEC1A	6.87E-04	0.652
EMILIN1	8.63E-04	0.426
ERG	7.20E-04	0.514
GGT5	3.90E-04	0.601
GRAP	3.50E-04	0.669
GSN	4.31E-04	0.609
HOXD9	5.86E-04	0.562
LSP1	5.39E-04	0.532
NRXN2	6.44E-04	0.408
PLCG2	5.36E-04	0.595
PTGIR	5.53E-04	0.532
RAD1	3.48E-04	1.259
RMI1	3.99E-04	1.529
RPL11	4.03E-04	0.736
SPATA2	5.54E-04	1.377
SPTBN5	3.53E-04	0.420
TNIP1	1.35E-04	0.768
TNIP2	2.98E-04	0.823
TPT1	4.98E-04	0.676
UPP1	9.22E-04	0.679

RYR3 signature ER+HER2-

Gene	*P*-value	Fold change
AAAS	1.42E-04	1.277
AES	9.91E-04	1.306
AFG3L2	2.24E-04	0.737
ATOX1	7.41E-04	1.380
BCL3	3.21E-04	1.360
C19orf24	4.68E-04	1.567
C2orf44	4.18E-04	0.815
CASK	9.19E-04	0.725
CD63	7.16E-04	1.317
CNPY2	9.14E-04	1.400
CREB1	8.39E-04	0.759
DPM2	4.78E-04	1.385
GPR137	5.40E-04	1.539
HIGD2A	5.87E-04	1.388
HTATSF1	1.81E-04	0.839
KDELR1	2.62E-04	1.349
MED1	6.88E-04	0.769
MLF2	5.57E-05	1.366
NAB2	9.91E-04	1.310
NAPA	5.00E-04	1.391
NCL	7.35E-04	0.837
NOC3L	7.49E-05	0.725
PIK3C3	6.63E-04	0.737
PSENEN	6.97E-04	1.421
RAB11FIP2	6.50E-04	0.726
RBCK1	9.40E-04	1.343
RNMT	1.61E-04	0.798
SCAND1	8.26E-04	1.653
SERTAD3	3.16E-04	1.270
SH3BGRL3	6.98E-04	1.482
SSSCA1	9.42E-04	1.505
TAF10	1.07E-04	1.267
TRIM44	8.06E-04	0.695
UBR5	9.91E-04	0.728
UQCRQ	4.84E-04	1.407
UTP20	4.75E-04	0.646
VPS4B	2.31E-04	0.826
WBP1	5.70E-05	1.362
WDR3	9.61E-05	0.821
YIPF2	1.93E-04	1.543
ZNF124	9.37E-04	0.727

STAG2 signature ER+HER2-

Gene	*P*-value	Fold change
BCAR3	1.57E-04	1.758
CLCA2	6.59E-04	4.127
F3	1.64E-04	2.160
GADD45A	4.75E-04	1.643
TMPRSS3	3.41E-04	3.143

TMEM50A signature ER+HER2-

Gene	*P* -value	Fold change
AMDHD2	2.97E-04	0.697
AMPD2	3.01E-04	0.802
ANAPC2	3.92E-04	0.810
APOE	2.57E-04	0.641
ARFRP1	2.79E-04	0.815
ARL6IP4	1.40E-04	0.730
ASPSCR1	1.47E-04	0.715
ATAD3A	1.41E-04	0.690
AURKAIP1	6.98E-05	0.679
BIN1	2.45E-06	0.484
BRF1	1.48E-04	0.771
BUB3	1.58E-04	1.246
CADM4	1.01E-04	0.656
CASK	1.99E-04	1.334
CCDC9	1.81E-05	0.590
CCS	3.52E-04	0.726
CD79B	1.61E-05	0.388
CDC37	1.53E-05	0.781
CENPQ	1.68E-04	1.316
CHMP6	3.68E-04	0.826
CSNK1G3	1.80E-04	1.225
CUL1	1.25E-04	1.157
DDIT4	3.32E-04	0.578
DDX54	1.73E-04	0.787
DUS1L	1.04E-04	0.749
DYNLT3	2.52E-04	1.337
ECHDC2	6.73E-05	0.723
EEF1D	1.39E-04	0.736
EHBP1L1	5.91E-05	0.634
EIF3G	6.80E-05	0.799
ERP29	3.38E-04	0.739
EVPL	1.50E-04	0.592
FKBP2	2.09E-05	0.680
FKBP8	1.83E-04	0.778
GADD45GIP1	1.26E-04	0.681
GLTSCR2	6.28E-05	0.749
HIP1R	3.81E-04	0.724
ICAM3	3.20E-05	0.750
IFT52	1.83E-04	1.179
IRF3	3.90E-04	0.723
KIAA1279	5.60E-06	1.255
KIF1C	3.48E-05	0.649
KLHDC4	1.49E-04	0.734
KRI1	1.60E-05	0.821
LSP1	1.18E-04	0.508
NARFL	2.42E-04	0.783
NECAP1	2.23E-04	1.301
NFKB2	1.11E-04	0.777

TMEM50A signature ER+HER2-

NT5C	1.05E-05	0.737
P4HA1	7.58E-05	1.353
PACSIN3	2.23E-04	0.659
PAF1	2.59E-04	0.814
PBXIP1	6.63E-05	0.687
PIGF	1.58E-04	1.164
PLCG2	3.91E-04	0.693
PLEKHA5	3.57E-04	1.235
PLEKHH3	1.60E-04	0.758
PPAN	3.25E-06	0.606
PPP3CB	2.07E-05	1.215
PSTPIP1	3.48E-04	0.539
PTPRCAP	3.38E-04	0.461
PUS1	2.76E-04	0.766
RAB11A	3.50E-04	1.180
RAB28	1.54E-04	1.127
RAD1	1.09E-04	1.195
RHOT2	2.88E-04	0.721
RPS3	1.02E-05	0.741
RRBP1	4.71E-05	0.709
RRP1	3.63E-06	0.786
SAC3D1	3.18E-04	0.730
SAFB	3.67E-05	0.789
SAFB2	4.70E-05	0.798
SAR1A	3.27E-04	1.114
SART1	7.25E-05	0.614
SCRN1	6.26E-05	1.574
SEMA3G	1.53E-04	0.537
SFXN1	2.63E-04	1.451
SLC35A5	3.21E-04	1.243
SLC38A10	2.55E-05	0.648
SNCG	1.96E-04	0.323
SNX2	3.70E-04	1.140
SNX24	3.33E-04	1.281
ST14	1.34E-04	0.693
SUPT5H	3.75E-04	0.822
TBL3	2.58E-04	0.793
TMED1	2.95E-04	0.782
TMEM209	1.32E-04	1.219
TNIP2	7.58E-05	0.834
TRPM4	2.91E-04	0.634
U2AF1	6.63E-05	0.828
UBQLN2	3.26E-04	1.169
VPS26A	2.73E-04	1.095
ZBTB17	1.87E-04	0.799
ZBTB7B	1.72E-04	0.743
ZFP30	1.91E-04	1.213
ZGPAT	6.73E-05	0.693
ZNF143	1.29E-04	1.119
ZNF219	3.06E-04	0.586
ZNF593	1.10E-04	0.742
ZNF787	1.41E-04	0.691

TTC27 signature ER+HER2-

Gene	*P*-value	Fold change
ALDH1A1	9.59E-04	0.472
ARMC6	1.55E-04	0.748
ASPSCR1	2.80E-04	0.669
BRD9	2.75E-04	0.811
CDC37	9.15E-04	0.780
CNOT4	4.43E-04	1.197
CUL1	1.02E-04	1.237
HPRT1	9.57E-04	1.379
IMPACT	6.86E-04	1.466
KIAA1279	2.06E-04	1.246
POT1	8.33E-04	1.339
RAB11A	2.40E-04	1.278
RPS3	5.43E-04	0.756
SF3B5	3.81E-04	0.812
USP47	8.74E-04	1.155
WASL	1.47E-04	1.261
YWHAE	8.29E-04	1.211
ZNF143	2.69E-05	1.138
ZNF287	1.67E-04	1.294

CROCC signature ER-HER2-

Gene	*P* -value	Fold change
HIC1	3.95E-04	1.842
RAD52	5.09E-04	0.752
TULP3	7.69E-04	0.698
