# Supplementary material for: An integrative bioinformatics approach reveals coding and non-coding gene variants associated with gene expression profiles and outcome in breast cancer molecular subtypes
Source: Br J Cancer. 2018 Mar 21;118(8):1107–14. doi: 10.1038/s41416-018-0030-0 (PMC5931099; doi:10.1038/s41416-018-0030-0)
Supplement: Supplementary file 10 — Supplementary Table 9 [file 41416_2018_30_MOESM10_ESM.pdf]

Supplementary Table 9. Validation of the variants-related transcriptional signatures associated with overall survival in breast cancer patients from the Metabric cohort

	Univariate analysis (validation cohort)	
**Gene**	***P*-value**	**HR**
**ER-positive/HER2-negative breast cancers (*n* = 1 386)**		
**Variants in coding regions**		
ABCA13	4.7E-03	1.33
CDH1[*]	2.6E-02	0.78
MAP3K1[*]	2.2E-09	0.52
MUC16	8.9E-02	0.82
NEB	1.5E-02	0.78
TAB3	3.0E-04	1.52
TP53[*]	1.1E-11	2.07
**Variants in non-coding regions**		
AAK1	2.0E-06	1.62
CA5A	7.9E-10	0.54
CRTC3[*]	5.2E-03	0.75
CTNNA2	9.2E-02	1.2
DOCK2	9.3E-02	1.19
FAM118A	1.1E-02	0.76
FASTKD1	2.0E-02	1.32
HDLBP	2.1E-01	1.15
HUS1	7.5E-07	1.65
PDZD7	2.0E-03	1.36
PPP1R12A	8.5E-02	1.19
RYR3	2.3E-02	1.26
STAG2[*]	1.7E-01	0.86
TMEM50A	2.3E-01	0.87
TTC27	6.4E-03	0.76
**ER-negative/HER2-negative breast cancers (*n* = 331)**		
**Variants in coding regions**		
MUC12	4.9E-02	0.65
RYR2	4.8E-02	0.7
**Variants in non-coding regions**		
CROCC	1.0E-01	0.74

Abbreviations: HR = hazard ratio.
